# Antimicrobial, Structural, Optical, and Redox Profiling of 7*H*‑Benzo[*c*]carbazol-10-ol Derivatives: An Integrated Experimental and Computational Study

**DOI:** 10.1021/acsomega.5c08465

**Published:** 2025-11-21

**Authors:** Mohamed S. H. Salem, Manar El Samak, Yasmine M. Abdel Aziz, Mohamed H. Aboutaleb, Sharvari Patil, Tin Zar Aye, Tarek S. Ibrahim, Shinobu Takizawa

**Affiliations:** † 13013 SANKEN, The University of Osaka, 8-1 Mihogaoka, Ibaraki-shi, Osaka 567-0047, Japan; ‡ Pharmaceutical Organic Chemistry Department, Faculty of Pharmacy, 68831 Suez Canal University, Ismailia 41522, Egypt; § Department of Microbiology and Immunology, Faculty of Pharmacy, 68831 Suez Canal University, Ismailia 41522, Egypt; ∥ Department of Pharmaceutical Chemistry, Faculty of Pharmacy, Horus University-Egypt, New Damietta 34518, Egypt; ⊥ Department of Pharmaceutical Chemistry, Faculty of Pharmacy, 108781 King Abdulaziz University, Jeddah 21589, Saudi Arabia; # Department of Pharmaceutical Organic Chemistry, Faculty of Pharmacy, Zagazig University, Zagazig 44519, Egypt

## Abstract

Carbazoles represent a versatile class of heterocycles with wide-ranging applications in medicinal and materials chemistry. However, their functionalized derivatives remain underexplored, largely due to challenges in synthesis and derivatization. This study investigates, for the first time, the integrated antimicrobial, photophysical, and redox properties of structurally novel 7*H*-benzo­[*c*]­carbazol-10-ol derivatives. These derivatives, distinct from parent carbazoles, were synthesized via a one-pot annulation and C–C coupling approach, enabling access to hydroxylated and naphthyl-substituted scaffolds with enhanced bioactivity and optoelectronic features. The target compounds were initially screened for their *in vitro* antimicrobial activity using the agar well diffusion assay. Compounds **3a** and **3c** exhibited superior activity over amoxicillin and ceftazidime against *P. aeruginosa* with (MIC = 8 μg/mL). Scanning electron microscopy (SEM) and molecular docking studies provided insights into the mode of antibacterial action and morphological changes. Moreover, these compounds exhibited unique redox and UV/vis absorption behavior, characterized by high absorption coefficients and distinct electronic transitions, as demonstrated by time-dependent density functional theory (TD-DFT) calculations. This dual profiling underscores their potential as antimicrobial agents and sustainable organophotocatalysts, offering new horizons in both medicinal and materials chemistry.

## Graphical abstract

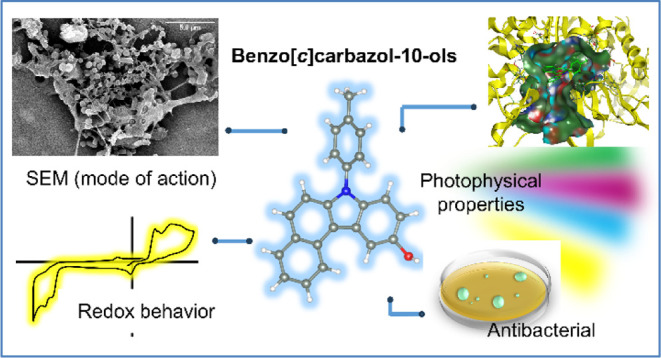

## 1 Introduction

Carbazoles are a versatile class of heterocyclic compounds with a rich history and broad applicability in both medicinal chemistry and materials science.^1–7^ The core structure of carbazoles consists of a tricyclic system, formed by two benzene rings fused to a nitrogen-containing five-membered ring (Figure 1). This highly conjugated π-system endows carbazoles with exceptional electronic, optical, and chemical properties, making them a key scaffold in the development of bioactive compounds and advanced materials.^8–10^ The parent compound, 9*H*-carbazole, was first isolated from coal tar in 1872 by Graebe and Glaser.^11^ Since then, carbazoles have evolved from chemical curiosities into indispensable molecules, influencing drug discovery, electronics, and energy materials.^12,13^ Notably, naturally occurring carbazole-containing molecules such as murrayanine, clausenal, clausenine, olivacin, and others exemplify the biological significance of these compounds (Figure 1A). For instance, murrayanine exhibits potent antimicrobial activity,^14,15^ while olivacin has demonstrated antiviral effects.^16^ These natural products highlight the therapeutic potential of carbazole derivatives, particularly in addressing infections.^17^

**Figure 1:**
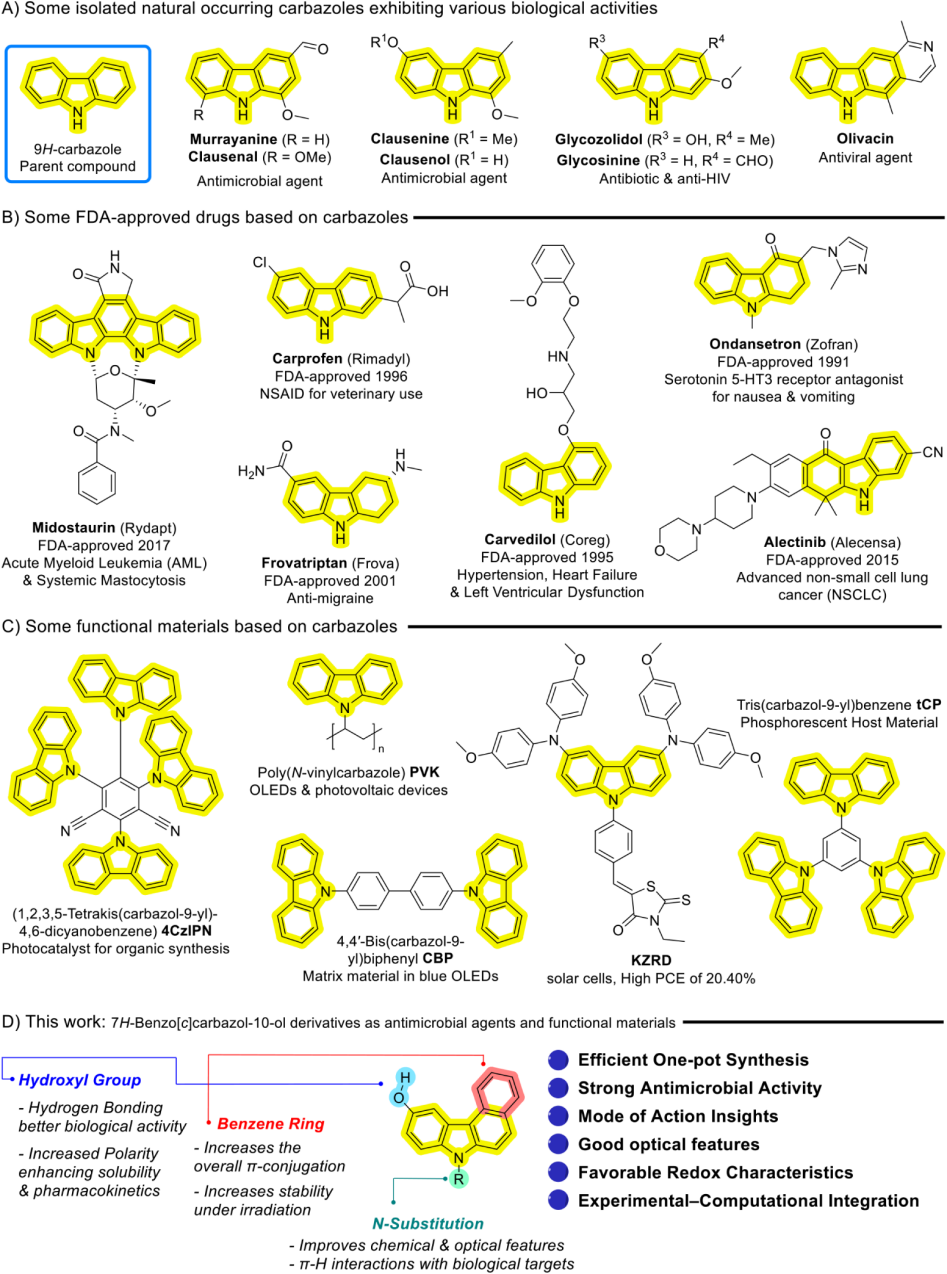
Medicinal and material-based applications of carbazole derivatives.

In addition to naturally occurring compounds, several carbazole-based drugs have received FDA approval for clinical use. Midostaurin (Rydapt), approved in 2017 for treating acute myeloid leukemia (AML) and systemic mastocytosis,^18^ and carvedilol (Coreg), approved in 1995 for hypertension and heart failure, are prime examples of the medicinal applications of carbazoles.^19^ Other notable FDA-approved drugs include ondansetron (Zofran) for nausea and vomiting,^20^ frovatriptan (Frova) for migraines,^21^ and alectinib (Alecensa) for advanced nonsmall cell lung cancer (NSCLC) (Figure 1B).^22^ These examples underscore the diverse therapeutic roles that carbazole derivatives can play. The medicinal potential of carbazole derivatives is further highlighted by their wide range of bioactivities.^6,23^ Numerous carbazole-based molecules have been developed as novel therapeutic agents,^24–28^ with a particular focus on antimicrobial applications.^29–31^ For example, *N*-substituted carbazoles, carbazole chalcones, and halogenated carbazole alkaloids have shown exceptional efficacy against resistant bacterial strains, including methicillin-resistant *Staphylococcus aureus* (MRSA).^32,33^ The antimicrobial action of carbazoles is attributed to their ability to interact with bacterial cells through various mechanisms such as DNA binding, disruption of membrane integrity, and interference with membrane-associated proteins.^34–37^ These properties make carbazoles invaluable tools in addressing the growing global challenge of antimicrobial resistance.

Carbazoles are also integral to materials science due to their exceptional electronic and optical properties.^38–44^ Poly­(*N*-vinylcarbazole) (PVK) is widely used in organic light-emitting diodes (OLEDs) and photovoltaic devices due to its excellent charge transport capabilities.^45,46^ Similarly, 4,4′-bis­(carbazol-9-yl)­biphenyl (CBP) is a commonly used matrix material in blue OLEDs.^47^ KZRD has achieved a high-power conversion efficiency (PCE) of 20.40% in solar cells, demonstrating the potential of carbazole derivatives in energy applications.^48^ Due to their favorable redox behavior, carbazoles have also been applied as photocatalysts; for example, 4CzIPN serves as an effective photocatalyst for organic synthesis (Figure 1C).^49^

Among the diverse derivatives of carbazoles, benzo­[*c*]­carbazoles have emerged as a particularly promising subclass due to their enhanced electronic properties. Recent studies indicate that the addition of an extra benzene ring can significantly improve their optical absorption and electron transport capabilities.^50^ The added hydroxyl group has the potential to act as a hydrogen bond donor (HBD), improving interactions with various biological targets. Despite some recent promising studies,^51^ benzo­[*c*]­carbazole derivatives remain largely unexplored, particularly regarding their potential as dual-purpose agents in bioactivity and materials science, due to challenges with their synthesis and derivatization.^52–54^ To address this gap, and encouraged by our previous work utilizing a one-pot synthetic approach to access these scaffolds,^55^ we report the synthesis of a series of 7*H*-benzo­[*c*]­carbazol-10-ol derivatives, including three novel compounds, via efficient one-pot and electrochemical/vanadium-mediated protocols (Figure 1D).^55,56^ We further present a comprehensive evaluation of their antimicrobial activity, photophysical behavior, and redox characteristics. Notably, this is the first report to integrate structure–activity relationships, SEM imaging, TD-DFT, and electrochemical redox profiling for this scaffold class. These findings position our compounds as promising candidates for both antibacterial drug development and organic photocatalysis, introducing a versatile platform for future applications.

## 2 Results and Discussion

### 2.1 Synthesis of 7*H*-benzo­[*c*]­carbazol-10-ol Derivatives

The synthesis of target carbazole derivatives was carried out as illustrated in Scheme 1A–D. Leveraging our expertise in carbazole chemistry and established synthetic methodologies primarily developed by our group,^55^ we have explored the reactivity of different carbazoles in the preparation of various functionalized molecules.^56–63^ Applying this knowledge, we aimed to synthesize a series of 7*H*-benzo­[*c*]­carbazol-10-ol derivatives, including three newly designed and some previously reported compounds by our group, to investigate their antimicrobial profile and optoelectronic features for some potential applications in materials science. Starting with the acid-mediated annulation of commercially available *N*-aryl-2-naphthylamines **1** and *p*-benzoquinone **2**, the 7*H*-benzo­[*c*]­carbazol-10-ol derivatives **3a**–**3e** were synthesized in good yields (Scheme 1A). This tandem process, involving sequential Michael addition followed by ring closure, was optimized to yield this series of substituted carbazoles **3a**–**3e** with distinctive substitution patterns. Further C–C bond coupling using either electrochemistry (**method A**)^61^ or vanadium chemistry (**method B**)^58,64^ afforded diol derivatives, featuring 2-naphthol substituents at position 11, **6a** and **6b** isolated in yields of 65–84% (Scheme 1A). Both methods demonstrated distinct and complementary advantages in terms of green, mild conditions, selectivity, and synthetic potential.^65^ The electrochemical approach (**method A**) provided milder, metal-free conditions and leveraged the differential redox potentials of the coupling partners to enable chemoselective heterocoupling. On the other hand, the vanadium-based method (**method B**) afforded higher yields and holds promise for enantioselective synthesis.

**Scheme 1:**
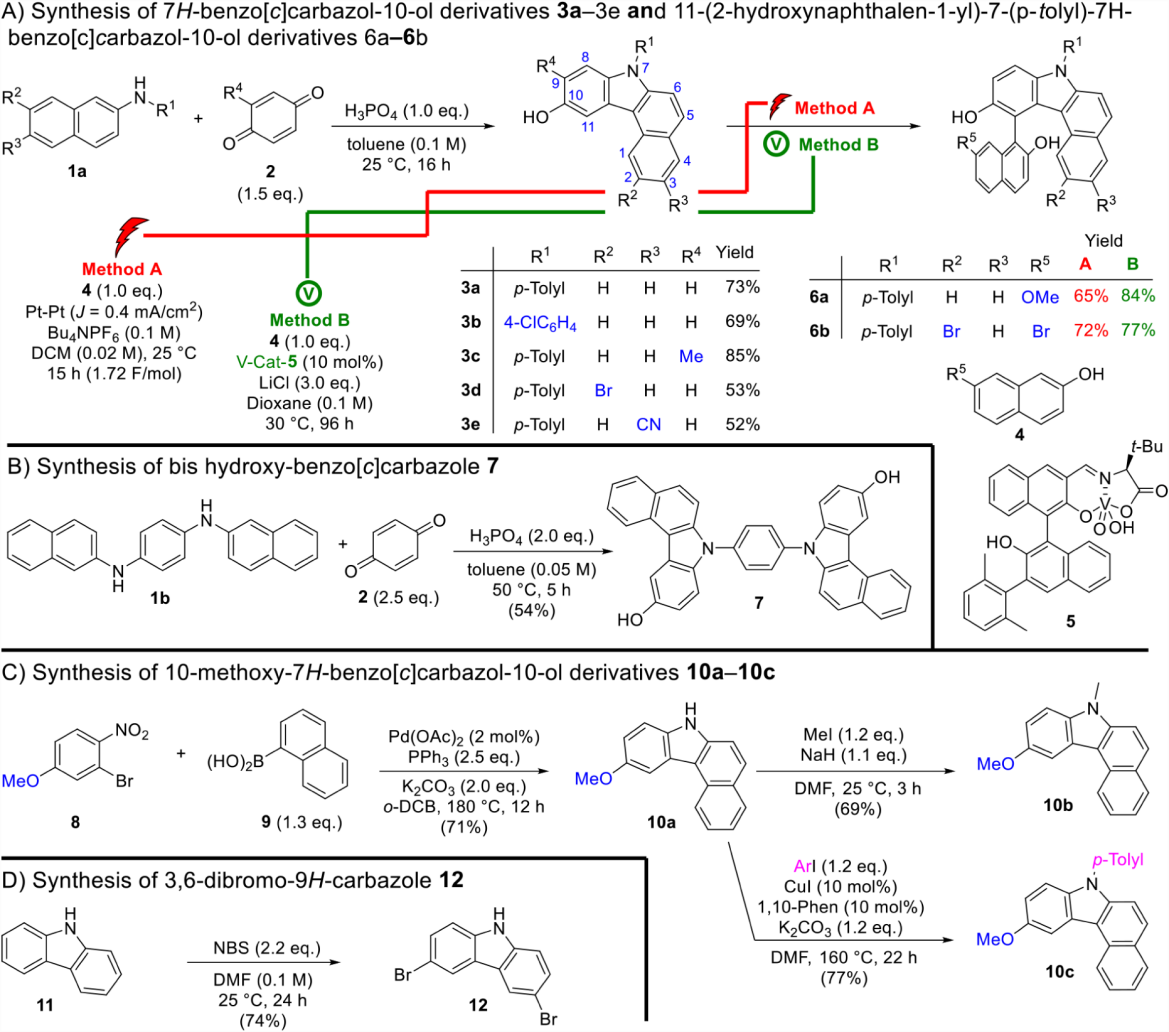
Synthesis of 7*H*-Benzo­[*c*]­carbazol-10-ols and Dibromo-9*H*-carbazole

In a similar approach with fine-tuned conditions (Scheme 1B), *N* ^1^,*N* ^4^-di­(naphthalen-2-yl)­benzene-1,4-diamine **1b** underwent a sequential reaction with **2** to yield 7,7’-(1,4-phenylene)­bis­(7*H*-benzo­[*c*]­carbazol-10-ol) **7** in 54% yield.^56,60^ Structural diversity was further expanded through the synthesis of methoxy-substituted carbazoles **10a**–**10c** via Suzuki–Miyaura coupling followed by cyclization (Scheme 1C).^59,61^ The reaction of 2-bromo-4-methoxy-1-nitrobenzene **8** with naphthalen-1-boronic acid **9** in the presence of Pd­(OAc)_2_ as a catalyst in *ortho*-dichlorobenzene (*o*-DCB) at 180 °C afforded 10-methoxy-7*H*-benzo­[*c*]­carbazole **10a** in 71% yield. Subsequent *N*-methylation or *N*-arylation using alkyl or aryl iodides yielded compounds **10b** and **10c** in 69% and 77% yields, respectively. Finally, 3,6-dibromocarbazole **12** was synthesized from the parent 9*H*-carbazole **11** using *N*-bromosuccinimide (NBS). These compounds, **11** and **12**, were used to investigate the effect of the additional benzene moiety on the antimicrobial activity, photophysical properties, optical characteristics, and redox behavior (Scheme 1D).

### 2.2 Antibacterial Activity of Carbazole Derivatives

The antimicrobial activity of the synthesized compounds was initially assessed using the agar well diffusion assay, which revealed varying degrees of antibacterial efficacy against standard pathogenic strains.^66,67^ Gram-positive bacteria, such as *Staphylococcus aureus*, are characterized by a thick peptidoglycan layer in their cell walls, rendering them more susceptible to agents that target cell wall synthesis.^68^ Conversely, Gram-negative bacteria, such as *Pseudomonas aeruginosa*, possess an outer membrane that confers additional protection, contributing to their heightened resistance to many antibiotics.^69^ The inhibition zone diameters for the tested compounds are shown in Table 1, illustrating their antibacterial profiles. Compounds **3a**–**3e** (except **3d**) and **7**, which are typical 7*H*-benzo­[*c*]­carbazol-10-ols, demonstrated good antibacterial activity against *S. aureus* ATCC-6538 and *P. aeruginosa* ATCC-9027, with inhibition zones up to 17 mm for **3e**. These results indicate that the hydroxyl group at the 10-position may enhance the compounds’ capacity to interact with bacterial membranes. This observation is further supported by comparing these results to those of their methoxy analogs, **10a**–**10c** (see the Supporting Information for assay images). More details on the antimicrobial effects of our carbazole derivatives against other Gram-negative bacteria (*Klebsiella pneumoniae* and *Escherichia coli*) and fungus *Candida albicans* can be found in the Supporting Information.

**Table 1:** Inhibition Zone Diameters Showing Antibacterial and Antifungal Activities of Carbazoles^a^

	Inhibition zone diameter (mm)	
Compound number	*S. aureus* ATCC 6538	*P. Aeruginosa* ATCC 9027
**3a**	8	11
**3b**	14	10
**3c**	10	10
**3d**	–	–
**3e**	17	17
**6a**	–	–
**6b**	–	–
**7**	15	14
**10a**	–	13
**10b**	–	–
**10c**	–	–
**11**	–	–
**12**	8	14

a (−) indicates no activity.

Following the initial agar well diffusion screen, we quantified antibacterial potency by determining MIC values for the active carbazoles (Table 2). Replicate microdilution assays (*n* = 2) gave the same MIC for each active carbazole. The compounds exhibiting activity against *P. aeruginosa* ATCC-9027 generally demonstrated MIC values ranging from 8 to 32 μg/mL, with **3a** and **3c** showing the lowest MIC of 8 μg/mL, indicating enhanced antibacterial potency. These values outperform ceftazidime (MIC > 128 μg/mL) and amoxicillin (MIC 16 μg/mL) in this bacterial strain, emphasizing the potential of this scaffold as an alternative antimicrobial agent following further optimization. Additionally, compounds **3a** and **3c** displayed MIC values of 8 μg/mL against *S. aureus* ATCC-6538, reinforcing their effectiveness against Gram-positive bacteria as well. For **3a**, the minimum bactericidal concentration (MBC) equaled the MIC (8 μg/mL) against both *P. aeruginosa* ATCC-9027 and *S. aureus* ATCC-6538 (MBC/MIC = 1), indicating a bactericidal effect by the ≤4 threshold.^70^ Compound **12** exhibited MIC values exceeding 128 μg/mL against both *P. aeruginosa* and *S. aureus*, suggesting that the lack of key structural features, such as hydroxyl groups and the extra benzene moiety, may diminish its efficacy. Compared to most of the previously reported carbazole derivatives, including *N*-arylcarbazole chalcones and halogenated analogs, the hydroxylated benzo­[*c*]­carbazoles (**3a**, **3c**) demonstrate markedly superior or comparable antibacterial potency with 2–8-fold against *S. aureus* and *P. aeruginosa*.^32,71,72^ This enhanced activity may be attributed to the extended conjugation and presence of a free hydroxyl group at position 10, which improves binding interactions with target bacterial proteins. These findings underscore the selective antibacterial activities of these compounds, which are influenced by their structural characteristics, such as hydroxyl groups, aromaticity, and the additional benzene moiety. Further investigation of the antimicrobial efficacy against Gram-negative bacteria, including *Klebsiella pneumoniae* and *Escherichia coli*, as well as the antifungal activity against *Candida albicans* ATCC-10231, demonstrated a more selective antibacterial effect (see the Supporting Information).

**Table 2:** Minimum Inhibitory Concentration (MIC) (μg/mL) of Carbazoles with Antibacterial Activity against Standard Pathogenic Bacteria^b^

	Minimum Inhibitory Concentration (MIC) (μg/mL)^a^	
Compound number	*S. aureus* ATCC-6538	*P. aeruginosa* ATCC-9027
**3a**	8	8
**3b**	32	16
**3c**	8	8
**3e**	16	16
**6b**	–	–
**7**	64	64
**10a**	–	>128
**12**	>128	>128
**Amoxicillin**	<0.25	16
**Ceftazidime**	–	>128
**Imipenem/cilastatin**	–	1

a MICs (μg/mL) are from two independent assays; identical results were obtained in both runs.

b (−) indicates that the compound was not tested for MIC because it did not show antimicrobial activity against the illustrated bacteria in the well diffusion assay.

### 2.3 Scanning Electron Microscopy

Scanning electron microscopy (SEM) was used to systematically investigate the morphological changes in *S. aureus* and *P. aeruginosa* before and after treatment with compounds **3a** and **3c**, the most potent agents based on their MIC values (Table 2).^73^ Untreated *P. aeruginosa* (ATCC-9027) exhibited a regular rod-shaped morphology (Figure 2A). Following treatment with MIC concentrations (8 μg/mL) of **3a** and **3c**, cells displayed significant structural alterations, including reduced cell size, shortened length, and evidence of surface damage (Figure 2B,C). For *S. aureus* (ATCC-6538), SEM images of untreated cells (Figure 2D) revealed their characteristic spherical shape. Treatment with compound **3a** resulted in substantial morphological changes, including cell wall distortion, blebbing, and debris formation, indicative of a bactericidal effect (Figure 2E). This observation aligns with the MBC data for **3a** (MBC = MIC = 8 μg/mL; MBC/MIC = 1) in both strains. In contrast, cells treated with **3c** showed a reduction in cell numbers but retained their overall morphology, suggesting a bacteriostatic effect where cell wall integrity was largely preserved (Figure 2F). These observations can suggest different modes of action for the tested compounds: **3a** exerts a bactericidal effect through direct cell wall rupture and cytoplasmic leakage, while **3c** inhibits bacterial proliferation without inducing structural collapse. Despite the structural similarity of the two compounds, the SEM images suggest that the methyl group in **3c**, located near the active site, might slightly suppress its bactericidal effect, potentially contributing to the observed bacteriostatic behavior. These insights can provide a foundation for further exploration of their potential as targeted antibacterial agents.

**Figure 2:**
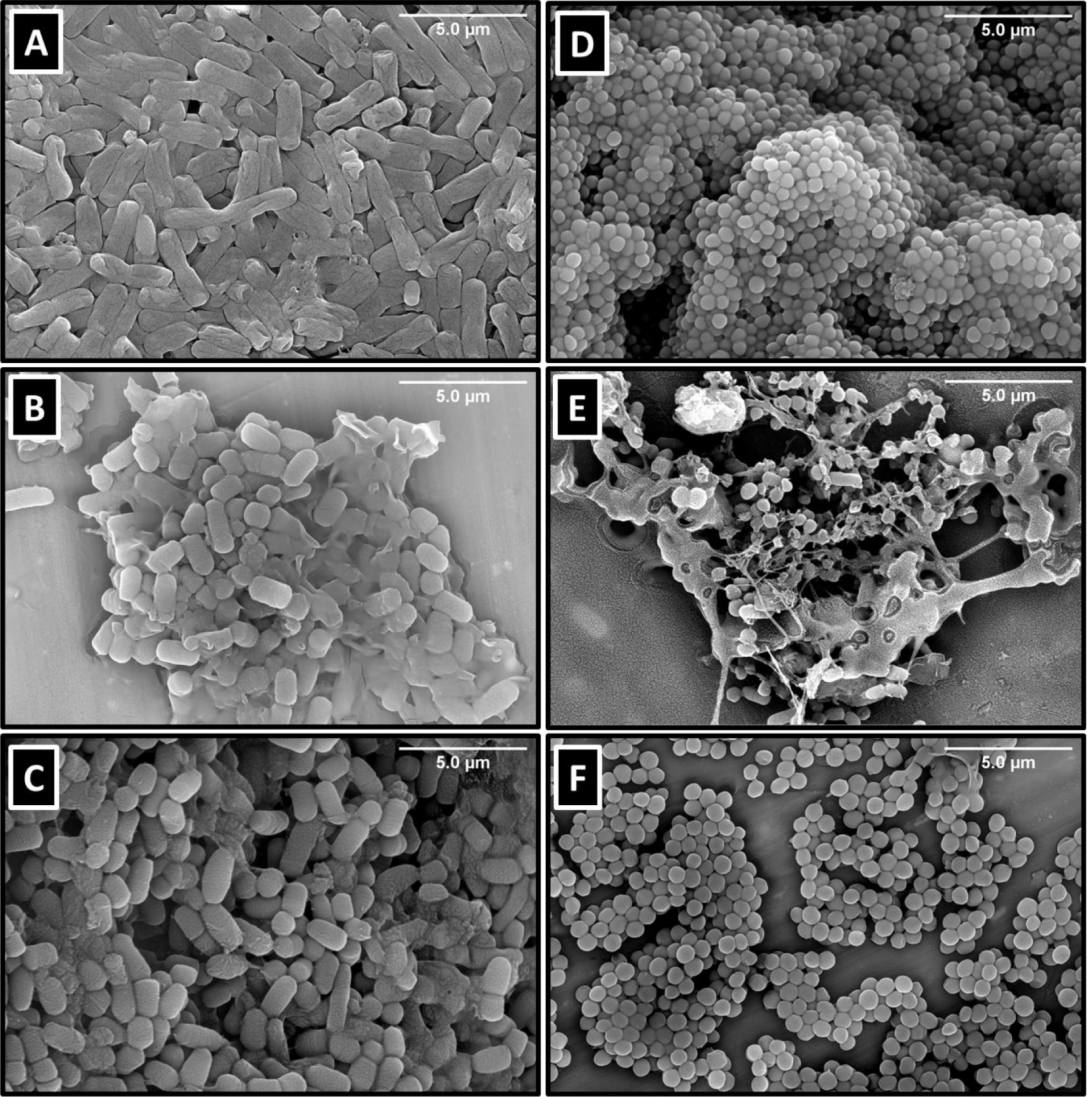
Scanning electron microscope (SEM) photomicrographs of *P. aeruginosa* and *S. aureus*. (**A**) Untreated *P. aeruginosa*. (**B**) *P. aeruginosa* after treatment with compound **3a** (8 μg/mL). (**C**) *P. aeruginosa* after treatment with compound **3c** (8 μg/mL). (**D**) Untreated *S. aureus*. (**E**) *S. aureus* after treatment with compound **3a** (8 μg/mL). (**F**) *S. aureus* after treatment with compound **3c** (8 μg/mL).

### 2.4 Molecular Modeling

#### 2.4.1 Molecular Docking

To gain deeper insights into the importance of some key structural features, such as the hydroxyl group, a preliminary molecular docking study of some analogous carbazole derivatives **3a** and **10c** in addition to ceftazidime and amoxicillin as reference antibiotics was conducted with penicillin-binding proteins (PBPs) from both *S. aureus* and *P. aeruginosa* using the Molecular Operating Environment (MOE) platform. MOE employs a force-field-based approach and scoring functions to evaluate ligand–protein binding affinities.^74^ The crystal structures of PBP3 from *S. aureus* (PDB ID: 3VSL) and *P. aeruginosa* (PDB ID: 3PBO) were retrieved from the Protein Data Bank.^75,76^ Docking protocol validation was initially performed by redocking the cocrystallized ligands into the active sites of their respective receptors.^77,78^ The docked poses successfully reproduced the binding conformations of the crystallographic ligands, maintaining the critical interactions. This confirmed the reliability of the docking workflow, as reflected by the low RMSD values of 1.74 Å and 1.69 Å for PDB IDs 3VSL and 3PBO, respectively (see Figures S9 and S10). The energy-minimized structures of carbazole derivatives **3a** and **10c** and the reference antibiotics ceftazidime and amoxicillin were docked into their active sites.

Docking analysis against penicillin-binding proteins (PBPs) 3VSL and 3PBO provided insights into the differential activity of compounds **3a** and **10c** compared to standard β-lactam antibiotics. As expected, the reference ligands ceftazidime and amoxicillin showed the strongest binding scores (−8.89 to −9.32 for ceftazidime; −6.61 to −6.95 for amoxicillin in 3VSL and 3PBO, respectively), forming multiple hydrogen bonds and ionic interactions with catalytically relevant residues such as Ser392, Ser448, Lys395, Thr487, and Asn351 (see Figures S11–S14). These results confirm the reliability of the docking protocol and reflect the well-established binding of β-lactam antibiotics within the active site of PBPs (Table 3).

**Table 3:** Docking Score of Compounds **3a**, **10c**, and the Reference Antibiotics Amoxicillin and Ceftazidime against the Penicillin Binding Protein (PDB ID: 3VSL and 3PBO)

	Docking score		H-bond (residues)		Hydrophobic interactions	
Compound	3VSL	3PBO	3VSL	3PBO	3VSL	3PBO
**3a**	–6.27	–6.03	Ser392	Ser294	Ser448, Thr603, Pro660	Phe533, Thr487, Ala488, Arg489, Tyr532
**10c**	–6.5	–6.54	None	None	Tyr430, Thr603, Pro660	Ser 349, Asn 351,Thr487, Ala488
Ceftazidime	–8.89	–9.32	Ser392, Ser448, Thr621, Lys395 (ionic)	Thr487, Glu291, Ser334, Asn351, Lys297, Val333, Arg489 (ionic)	None	None
Amoxicillin	–6.61	–6.95	Ser392, Thr603, Thr619, Ser429, Thr621, Lys395 (ionic)	Ser349, Ser485, Thr487, Ser349, Thr487, Asn351, Lys484 (ionic)	None	None

Compound **3a**, which demonstrated measurable antibacterial activity *in vitro*, exhibited docking scores (−6.27 and −6.03 in *3VSL* and *3PBO*, respectively) comparable to amoxicillin. Importantly, **3a** established hydrogen bonding with Ser392 in *3VSL* and Ser294 in *3PBO*, residues critical for acylation of the catalytic serine, as well as hydrophobic interactions with Thr603, Pro660, and Tyr532. The presence of these stabilizing contacts likely contributes to the observed biological activity, suggesting that **3a** may mimic the binding orientation of β-lactam antibiotics despite its distinct scaffold. The hydroxyl substitution in **3a** appears to play a key role in hydrogen bond formation and target engagement (Figure 3A,B).

**Figure 3:**
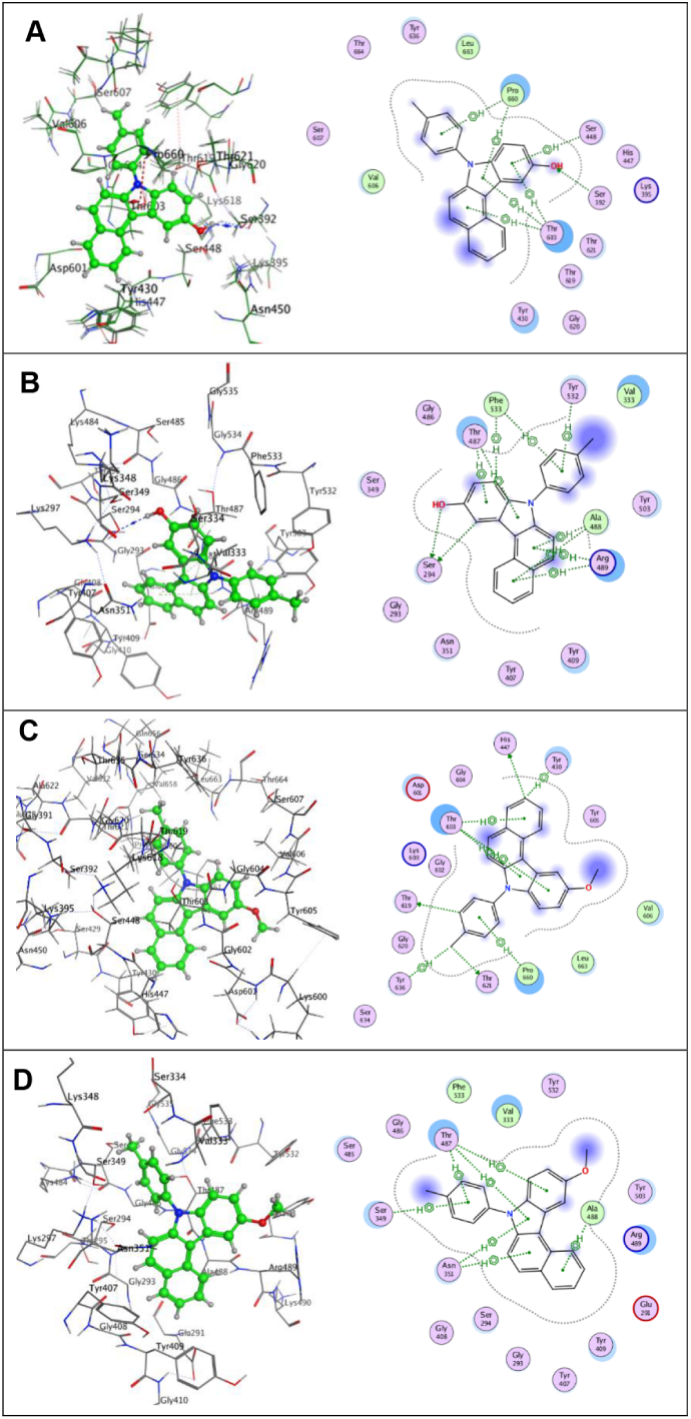
Molecular docking studies of 7*H*-benzo­[*c*]­carbazol-10-ol derivatives. (**A**) 3D and 2D conformations of compound **3a** docked in the *S. aureus* PBP3 active site (PDB ID: 3VSL). (**B**) 3D and 2D conformations of **3a** docked in the *P. aeruginosa* PBP3 active site (PDB ID: 3PBO). (**C**) 3D and 2D conformations of compound **10c** docked in the *S. aureus* PBP3 active site (PDB ID: 3VSL). (**D**) 3D and 2D conformations of compound **10c** docked in the *P. aeruginosa* PBP3 active site (PDB ID: 3PBO).

In contrast, compound **10c**, which lacked antibacterial activity, produced similar docking scores (−6.50 and −6.54 in *3VSL* and *3PBO*, respectively) but failed to establish hydrogen bonds with active site residues. Instead, its binding was dominated by weaker hydrophobic interactions with residues such as Tyr430, Pro660, and Ala488, with no engagement of the catalytic serine. The absence of strong polar contacts likely explains the poor experimental activity of **10c**, highlighting that docking scores alone are insufficient to predict biological efficacy without considering interaction quality and positioning within the binding pocket (Figure 3C,D).

These findings demonstrate that compound **3a** achieves activity by forming stabilizing hydrogen bonds with catalytically relevant residues resembling β-lactams, while compound **10c**, despite having similar docking energies, lacks such interactions and is biologically inactive. The docking outcomes thus correlate well with experimental observations and provide a structural rationale for the activity differences observed. The pharmacological and bioavailability profiles of **3a** and **10c** were further investigated computationally, revealing a low BBB penetration but a higher probability of GI absorption, outperforming amoxicillin (see the Supporting Information).

#### 2.4.2 Molecular Dynamics Simulation

A molecular dynamics simulation was conducted for the active compound **3a** in the crystal structures of PBPs from *S. aureus* (PDB ID: 3VSL). The simulation began with the best-docked poses of **3a** from the docking study using it as the initial frame (frame-0). A 100 ns molecular dynamics simulation was performed in an explicit hydration environment for all structures. System stability and molecular trajectories were evaluated based on the root-mean-square deviation (RMSD) of protein Cα-atoms and ligand heavy atoms. Figure 4A presents the RMSD values over simulation time. The Cα RMSD of the protein backbone fluctuated initially within the first 10 ns, indicating the system’s equilibration phase. After about 20 ns, the protein stabilized, with RMSD values fluctuating between 6–9 Å, reflecting moderate conformational flexibility throughout the simulation. This degree of fluctuation is consistent with the intrinsic plasticity of PBPs, which undergo conformational changes to accommodate ligands and catalytic processes. The ligand RMSD followed a similar trend, stabilizing after the initial equilibration and maintaining values in the 5–7 Å range for most of the trajectory. The ligand RMSD did not show any large drifts beyond 9 Å, suggesting that compound **3a** remained stably associated within the active site during the entire 100 ns production run. The comparable fluctuation profiles of the protein and ligand RMSDs further confirm that the ligand’s movement was coordinated with the protein’s conformational adjustments rather than reflecting dissociation.

**Figure 4:**
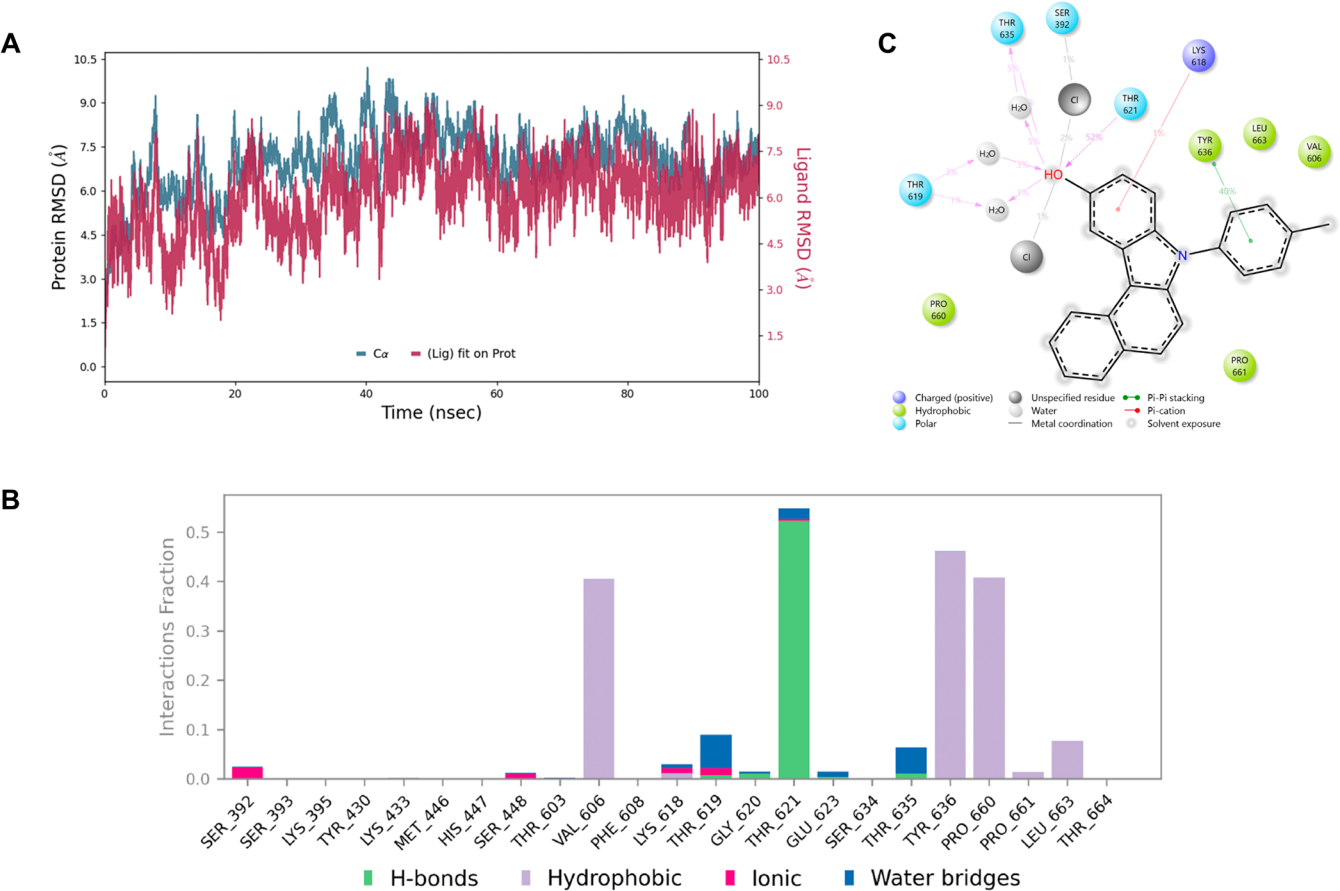
100 ns simulation interaction diagram panel for compound **3a** and protein (PDB ID: 3VSL) . (A) RMSD values for the PBP protein Cα-atoms (blue) and ligand **3a** heavy atoms (red). (B) Fractions of interaction between **3a** and PBPs active site residues. (**C**) 2D interaction diagram of **3a** within the PBP active site.

The molecular dynamics simulation interaction timeline indicates that compound **3a** maintained a well-defined network of contacts in the PBP active site over the 100 ns trajectory. Hydrogen bonding with Thr621 was the most predominant for more than 52% of the simulation time that acts as the primary anchor for the ligand. Additionally, lower-frequency H-bonds through water bridges were also observed with **Ser392**, **Thr619,** and **Thr635**, suggesting intermittent engagement of the catalytic region. Hydrophobic contacts forming a complementary clamp around **3a** were also observed, mostly with Tyr636, Pro660, and Val606 for 46%, 40.8%, and 40.5% of the simulation time, respectively. Hydrophobic contacts were also observed to a lesser extent with Pro661 and Leu663. This hydrophobic shell stabilizes the aromatic core of **3a** and helps limit large ligand displacement. Ionic interactions were also sporadic with Lys618 and Ser448, but their presence indicates favorable electrostatic affinity near the pocket.

In conclusion, the most predominant Thr621 H-bond, in addition to the hydrophobic shell provided by Tyr636, Pro660, and Val606, and the water-mediated contacts with Thr619 and Ser392 established an interaction network that accounts for the stable binding of **3a** in the PBP active site and is consistent with its demonstrated antibacterial activity. The relatively weak and intermittent interaction with the catalytic Ser392 indicates that the potency of this scaffold is driven less by a classical β-lactam-like acylation mechanism and more by the combined effects of anchoring at Thr621 and hydrophobic stabilization. This interaction pattern helps explain why close analogs lacking these persistent polar and nonpolar contacts such as compound **10c** fail to translate docking scores into activity (Figure 4B,C).

### 2.5 Photophysical Properties

Carbazole derivatives have garnered significant attention in material-based applications, particularly in the development of organic semiconductors, photonic devices, and light-emitting materials, due to their good optical and electronic properties.^1–3^ Their strong UV/vis absorption makes them ideal candidates for exploring structure–property relationships critical to advancing these technologies. Here, we focus on 7*H*-benzo­[*c*]­carbazol-10-ol derivatives that have not been previously investigated, aiming to elucidate their optical behavior and understand the key molecular features that drive their photophysical properties. The carbazole derivatives synthesized in this study (Scheme 1A–C) exhibit remarkable absorption upon photoirradiation, attributed to their rigid molecular scaffold. The UV/vis absorption spectra of these derivatives were recorded in chloroform (concentration = 5.0 × 10^–5^ M), displaying consistent absorption patterns (Figure 5A–D). Most *7H*-benzo­[*c*]­carbazole derivatives **3a**–**3e** exhibited an absorption onset around 400 nm, with two main characteristic peaks: approximately 375 and 335 nm. Among them, derivative **3a** showed its highest absorbance peak at 378 nm (absorption coefficient ε = 5.14 × 10^3^ M^–1^ cm^–1^), corresponding to an optical bandgap of *E*_g_ = 3.17 eV (Figure 5E).^79^

**Figure 5:**
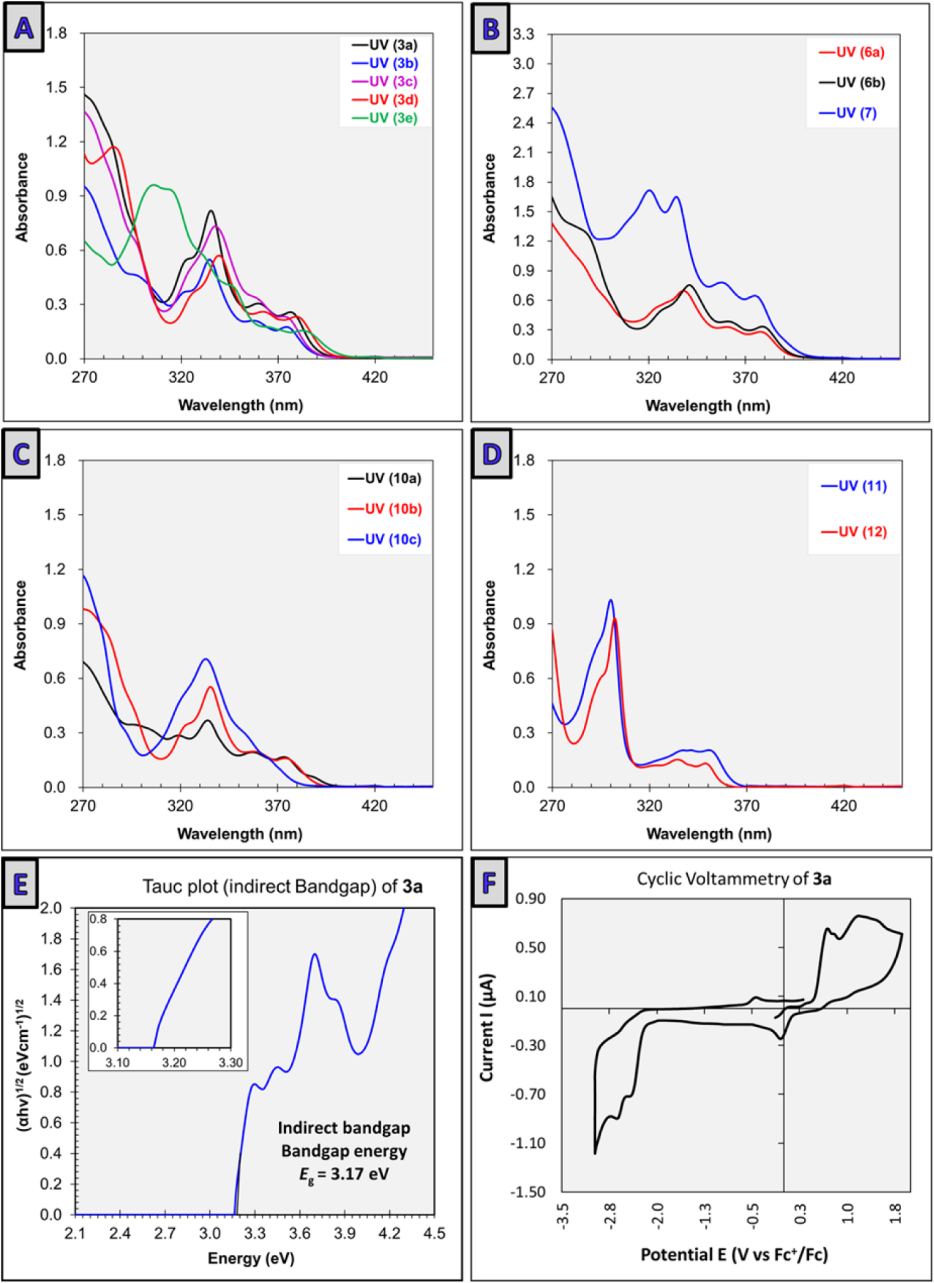
Photophysical and redox behavior of carbazole derivatives. (A) UV/vis absorption spectra of **3a**–**3e** in chloroform (50 μM). (B) UV/vis absorption spectra of **6a**, **6b,** and **7** in chloroform (50 μM). (C) UV/vis absorption spectra of **10a**–**10c** in chloroform (50 μM). (D) UV/vis absorption spectra of **11** and **12** in chloroform (50 μM). (E) Tauc plot of compound **3a**. (F) The cyclic voltammetry profile of **3a** in MeCN with *n*-Bu_4_NPF_6_ (0.1 M) using Fc/Fc^+^ as the internal reference.

Time-dependent density functional theory (TD-DFT) calculations of **3a** at the B3LYP/6-311+G­(d,p)/PCM = chloroform and B3LYP/6-311G/PCM = chloroform levels of theory indicate that the low-energy absorption band at 378 nm is primarily due to the HOMO → LUMO transition. The band at 337 nm can be attributed to the HOMO–1 → LUMO transition (see the Supporting Information for more details). Molecular orbital calculations revealed that the highest occupied molecular orbital (HOMO) is localized across the entire scaffold, whereas the lowest unoccupied molecular orbital (LUMO) is primarily localized over the carbazole moiety, with no contribution from the *N*-aryl substitution (Figure 6A).

**Figure 6:**
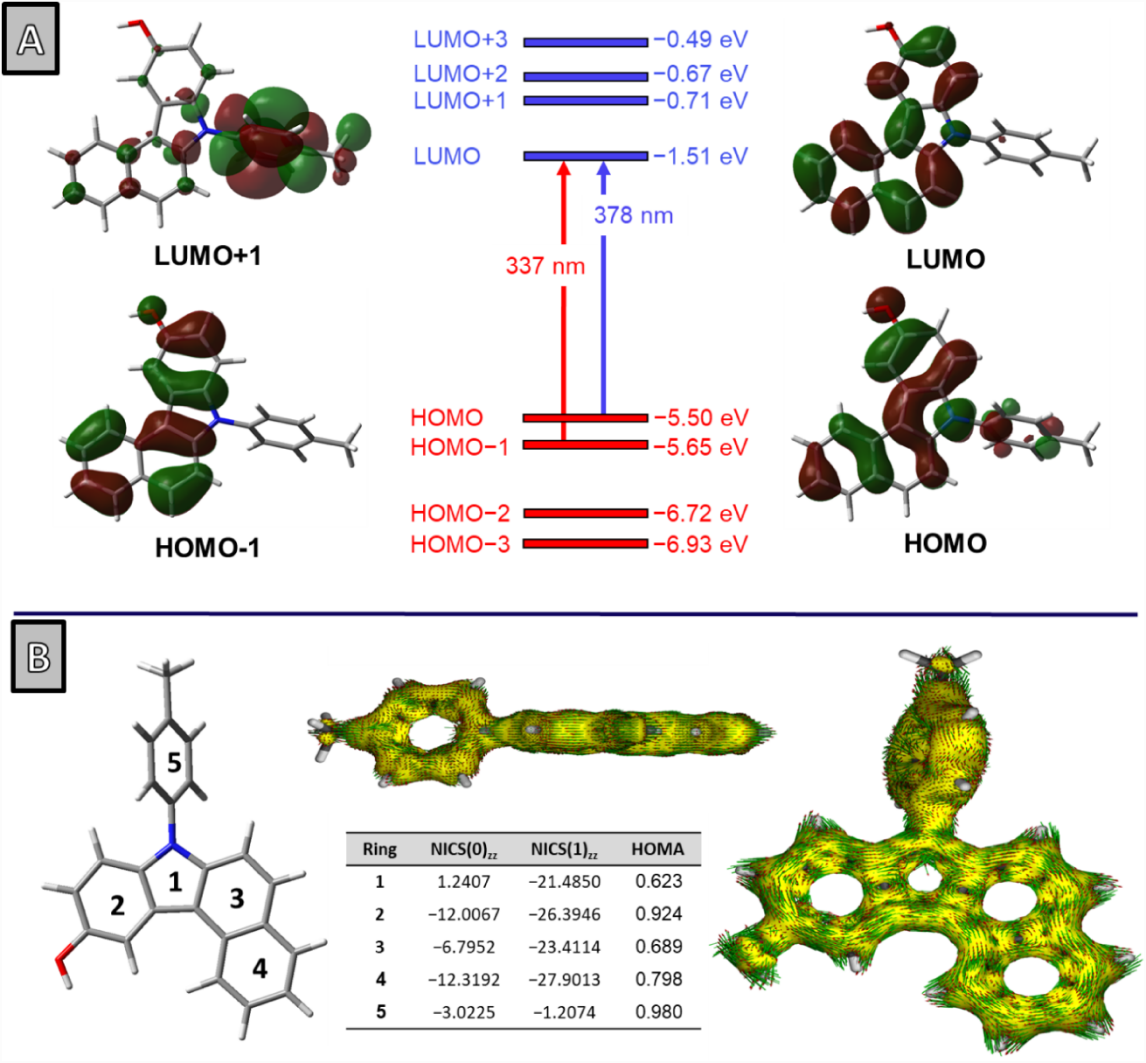
Electronic transitions and aromaticity of **3a**. (A) Frontier Kohn–Sham molecular orbital calculations and TD-DFT calculated electronic transitions at the B3LYP/6-311+G­(d,p)/PCM = chloroform level of theory. (B) AICD plots, NICS, and HOMA values of **3a** calculated at the same level of theory (isosurface value: 0.05).

Interestingly, 11-(2-hydroxynaphthalen-1-yl)-7-(*p*-tolyl)-7*H*-benzo­[*c*]­carbazol-10-ol derivatives **6a** and **6b** and bis-hydroxy-benzo­[*c*]­carbazole **7** displayed absorption onsets similar to derivatives **3a**–**3e**, starting around 400 nm. These compounds exhibited comparable characteristic peaks but showed higher absorption coefficients (Figure 5B). Compounds **10a**–**10c** exhibited similar absorption patterns but with lower absorption coefficients (Figure 5C). On the other hand, 9*H*-carbazoles **11** and **12** showed absorption onsets starting around 365 nm rather than 400 nm, reflecting the loss of the π-extended conjugated system (Figure 5D).

Conventional 9*H*-carbazole-based chromophores typically exhibit UV absorption in the range of 327–337 nm, with occasional extension up to 350 nm, primarily attributed to π → π* electronic transitions.^80^ In contrast, established OLED materials such as 4,4’-bis­(carbazol-9-yl)­biphenyl (CBP) display absorption peaks at 293, 317, and 340 nm, with the lowest-energy absorption edge around 340 nm.^81^ In comparison, 7*H*-benzo­[*c*]­carbazol-10-ol derivatives in the current study exhibit absorption onsets near or beyond 370 nm, indicating a pronounced red shift. This shift, along with their notably higher molar absorption coefficients, highlights the critical role of the fused benzene ring and hydroxyl substitution in extending conjugation and enhancing optical performance relative to both parent carbazoles and conventional OLED scaffolds. Practically, the red-shifted onsets (∼370–400 nm) and elevated ε enable efficient UV/near-visible light harvesting compatible with photopatterning and blue/near-UV emitter/host architectures. Consistent with TD-DFT analysis (Figure 6A), a HOMO delocalized across the framework and a LUMO centered on the carbazole core suggest limited intramolecular charge transfer, favoring photostability and hole-transport character; together with the measured optical gap for **3a** (*E*_g_ = 3.17 eV), these attributes place the series in a practically useful spectral/electronic window for organic optoelectronics.^82^

### 2.6 Aromatic Behavior

Subsequently, we employed nucleus-independent chemical shifts (NICSs), proposed by Schleyer and colleagues,^83,84^ as a magnetic indicator, and the harmonic oscillator model of aromaticity (HOMA) as a geometrical index,^85,86^ to examine how the additional benzene moiety (Ring 4), hydroxyl substitution, and *N*-substitution (Ring 5) influence aromaticity in a comparative context. As shown in Figure 6B, compound **3a** features five rings, each exhibiting distinct aromatic behavior. Rings 2 and 4 display the strongest aromaticity, with highly negative NICS(1)_zz_ values (−26.39 and −27.90, respectively) and high HOMA scores (0.924 and 0.798), indicating significant π-electron delocalization. The presence of an OH group at Ring 2 slightly reduces its aromaticity compared to the nonhydroxylated system (NICS(1)_zz_ = −26.39 vs −28.31), suggesting that resonance donation from the hydroxyl group counteracts the minor inductive electron-withdrawing effects. Ring 3 exhibits moderate aromaticity, with NICS(1)_zz_ = −23.41, which is less negative than in the system lacking Ring 4 (NICS(1)_zz_ = −27.37). This indicates that Ring 4 disrupts the delocalization in Ring 3, possibly due to electronic effects on the conjugated framework. Conversely, when Ring 4 is absent, Ring 3 exhibits enhanced aromaticity (NICS(1)_zz_ = −27.37 and HOMA = 0.929), reinforcing that its interaction with Ring 4 perturbs the delocalization to some extent. Ring 1 (pyrrole) shows weaker aromatic stabilization, as indicated by its NICS(0)_zz_ value of +1.24, which suggests structural distortion due to the presence of the *N*-substituent (Ring 5).

Analyzing the isosurfaces from the anisotropy of the induced current density (AICD) plots of **3a** provides further insight into their electronic delocalization.^87^ When a magnetic field is applied perpendicular to the molecular plane of **3a** (along the +*z*-axis, upward), conjugation through the radial C–C bonds is diminished, revealing diatropic currents (clockwise) in the outer rim of the carbazole scaffold (Figure 6B).

### 2.7 Redox Behavior

The electrochemical properties of our carbazole derivatives were investigated using cyclic voltammetry (CV) and DFT calculations, revealing remarkable redox behavior that underscores their chemical stability and redox versatility. Notably, irreversible redox peaks were observed in both the anodic and cathodic regions, indicating hindered interconversion between the neutral forms and their corresponding anion/cation pairs (Figure 5F).^88^ The irreversibility observed in the anodic region can be attributed to the high acidity (p*K*_a_ ∼ −5.15, DFT-calculated; see the Supporting Information) of the generated radical cation [**3a**]^•+^ upon oxidation, which facilitates the release of a proton. To quantify the redox potentials, CV measurements were conducted in acetonitrile (MeCN) with 0.1 M tetrabutylammonium hexafluorophosphate (Bu_4_NPF_6_) as the supporting electrolyte. Ferrocene/ferrocenium (Fc/Fc^+^) was employed as an internal reference. Under these conditions, compound **3a** exhibited an oxidation potential of 0.64 V vs Fc/Fc^+^, which corresponds to 1.02 V vs the saturated calomel electrode (SCE). Similarly, the reduction potential was measured to be −2.37 and −2.64 V vs Fc/Fc^+^, which corresponds to −1.99 and −2.26 V vs SCE (Figure 5F).

To extend the study of redox potentials of various carbazole derivatives in both their ground and excited states, we employed DFT calculations adopting the computational protocol established by Nicewicz and coworkers.^89^ Redox potentials were calculated using the B3LYP functional in conjunction with the 6-311+G­(d,p) basis set and the CPCM continuum solvation model for acetonitrile. All computations were carried out with Gaussian 09 and 16, beginning with geometry optimizations followed by frequency analyses to confirm the absence of imaginary frequencies, thereby validating the optimized structures as true minima.^90^ Additionally, the frequency calculations provided free energies at 298 K, which were used to derive solution-phase redox potentials. Solution-phase Gibbs free energies were referenced to SCE. This was achieved by subtracting the absolute potential of the standard hydrogen electrode (SHE, 4.281 V) and the conversion factor for SHE to SCE in acetonitrile (0.141 V). The redox potentials were calculated using the following formula (eq 1):

1 $$E_{1/2}^{o,calc}=-\frac{\left(G_{298}\left[\mathrm{reduced}\right]-G_{298}\left[\mathrm{oxidized}\right]\right)}{n_{e}F}-E_{1/2}^{o,SHE}+E_{1/2}^{o,SCE}$$

where *n*_e_ is the number of electrons transferred (*n*_e_ = 1), F is the Faraday constant (23.06 kcal mol^–1^ V^–1^), *E*_1/2_^o,SHE^ is the absolute value for SHE (4.281 V), *E*_1/2_^o,SCE^ is the potential of the SCE relative to SHE in acetonitrile (−0.141 V), and *G* oxidized and *G* reduced are the Gibbs free energies in acetonitrile as obtained from DFT calculations.

The redox characteristics of carbazole derivatives demonstrate their significant potential as organophotocatalysts in photoredox catalysis.^91^ These compounds exhibit remarkably negative reduction potentials, with ground-state values up to −2.37 V vs SCE and excited-state potentials spanning −1.58 to −2.16 V vs SCE (Table 4). The redox potentials of **3a**–**3c** fall within the range of highly reducing photocatalysts, with a reducing power more than 4CzIPN (*E*_1/2_* ≈ −1.21 V) and eosin Y (*E*_1/2_* ≈ −1.06 V),^92,93^ and more negative ground-state reduction potentials (e.g., −2.31 V for **3a**), suggesting enhanced electron-acceptor capability. Their excited-state oxidative potentials (0.70–0.93 V vs SCE) further confirm their effectiveness as photoreductants under mild conditions. The fused benzene moiety in our scaffold significantly modulates redox behavior, decreasing oxidation potentials by 0.15–0.25 V and increasing reduction potentials by 0.3–0.5 V compared to nonfused analogs (see the Supporting Information). This enhanced redox activity stems from improved charge delocalization across the extended π-system. While the 10-OH group shows modest electronic effects (Δ*E* ^1/2^ < 0.1 V vs nonhydroxylated analogs), it provides crucial hydrogen-bonding sites that may influence catalytic activity through substrate interactions. Notably, the combination of benzannulation and hydroxylation yields unique redox profiles unattainable with simpler carbazole architectures. The most active derivatives surpass common transition-metal photocatalysts in reducing power while maintaining oxidative capability sufficient for diverse radical transformations. These advantageous redox characteristics, combined with the inherent benefits of organic photocatalysts, including low toxicity, cost efficiency, and structural tunability, position our derivatives as sustainable, metal-free alternatives with customizable redox behavior. Their balanced redox profiles suggest particular utility in challenging reductive transformations requiring strong reductants, while the modular scaffold allows for further optimization through targeted substitution.^94^

**Table 4:** DFT Calculation of the Redox Potentials of Carbazole Derivatives

Compound number	Oxid. Pot. ground state *E* ^1/2^(A^•+^/A)	Red. Pot. ground state *E* ^1/2^(A/A^•–^)	Oxid. Pot. excited state *E* ^1/2^(A*/A^•–^)	Red. Pot. excited state *E* ^1/2^(A^•+^/A*)
**3a**	1.02	–2.31	0.80	–2.09
**3b**	1.08	–2.28	0.86	–2.06
**3c**	0.97	–2.37	0.74	–2.15
**3d**	1.09	–2.18	0.86	–1.96
**3e**	1.16	–1.80	0.93	–1.58
**6a**	1.05	–2.28	0.80	–2.02
**6b**	1.19	–2.16	0.85	–1.83
**7**	1.04	–2.28	0.85	–2.09
**10a**	0.99	–2.36	0.79	–2.16
**10b**	0.89	–2.33	0.70	–2.14
**10c**	0.96	–2.34	0.76	–2.14
**12**	1.39	–2.31	1.15	–2.08

The redox profiles, aromaticity maps (NICS, HOMA), and AICD currents show that o/m/p π-extension systematically tunes delocalization across rings 2–4. This conjugation control is directly relevant to excited-state electron-transfer propensity: more delocalized members facilitate SET under near-UV excitation, a property leveraged in photoredox processes and consistent with the bactericidal profile of **3a** (MBC/MIC = 1). In a medicinal context, increased planarity/delocalization supports π-surface interactions and membrane insertion, while the phenolic −OH modulates H-bonding/recognition, aligning the electronic structure with the observed antibacterial activity.

## 3 Experimental Section

### 3.1 General Experimental Details

^1^H and ^13^C NMR spectra were recorded using JEOL JMN ECS400 FT NMR, JNM ECA600 FT NMR, or Bruker AVANCE II instruments (^1^H NMR: 400 or 600 MHz; ^13^C NMR: 101 or 151 MHz). The ^1^H NMR spectra are reported as follows: chemical shifts in ppm downfield of tetramethylsilane (TMS), referenced to the residual solvent peak (CDCl_3_ at 7.26 ppm or (CD_3_)_2_CO at 2.05 ppm), along with integration, multiplicities (s = singlet, d = doublet, t = triplet, q = quartet, m = multiplet), and coupling constants (Hz). The ^13^C NMR spectra are reported in ppm relative to the central line of the triplet for CDCl_3_ at 77.16 ppm or the central line of the septet for (CD_3_)_2_CO at 29.84 ppm. APCI-MS spectra were obtained using a JMS-T100LC (JEOL). FT-IR spectra were recorded on a JASCO FT/IR4100 system. Thin-layer chromatography (TLC) of reaction mixtures was performed on Merck silica gel 60 F254 TLC plates, visualized under UV light. Column chromatography was conducted on Kanto Silica Gel 60 (63–210 μm). UV/vis absorption spectra were recorded on a JASCO V-670 spectrophotometer. Cyclic voltammetry (CV) was performed using a BAS CV-620C voltammetric analyzer with a platinum disk as the counter electrode and an Ag/AgNO_3_ reference electrode at a scan rate of 100 mV s^–1^. Commercially available organic and inorganic reagents were used without further purification. Electro-oxidation experiments were conducted using platinum plate electrodes (1.3 × 1.5 cm^2^) connected to a DC power supply (KIKUSUI PMX 35-1A). Scanning electron microscopy (SEM) was performed on a Quanta FEG 250 system (Thermo Fisher Scientific).

### 3.2 Synthetic Procedures

#### 3.2.1 Synthesis of 7*H*-Benzo­[*c*]­carbazol-10-ol Derivatives **3a**–**3e**

To a solution of *N*-aryl-2-naphthylamines **1a** and *p*-benzoquinone **2** (1.5 equiv) in dry toluene (0.1 M), orthophosphoric acid (1.0 equiv) was added dropwise. The reaction mixture was stirred at 25 °C for 16 h under a nitrogen atmosphere until completion. Afterward, the reaction was quenched with water and extracted with EtOAc, and the combined organic layers were dried over Na_2_SO_4_ and concentrated under reduced pressure. The crude product was purified by column chromatography (SiO_2_, EtOAc/*n*-hexane), yielding 7*H*-benzo­[*c*]­carbazol-10-ol derivatives **3a**–**3e** in good yields, as illustrated in Scheme 1A.

##### 3.2.1.1 7-(*p*-Tolyl)-7*H*-benzo­[*c*]­carbazol-10-ol **3a**

White solid, ^1^H NMR (400 MHz, CDCl_3_): δ 8.72 (d, *J* = 8.2 Hz, 1H), 8.07 (d, *J* = 2.7 Hz, 1H), 7.99 (d, *J* = 7.8 Hz, 1H), 7.80 (d, *J* = 8.7 Hz, 1H), 7.7 (td, *J* = 7.5, 0.9 Hz, 1H), 7.52 (d, *J* = 9.2 Hz, 1H), 7.47 (ddd, *J* = 7.8, 7.3, 0.9 Hz, 1H), 7.40–7.45 (m, 4H), 7.35 (d, *J* = 8.7 Hz, 1H), 6.99 (dd, *J* = 8.9, 2.5 Hz, 1H), 4.90 (s, 1H), 2.51 (s, 3H).^61^

##### 3.2.1.2 7-(4-Chlorophenyl)-7*H*-benzo­[*c*]­carbazol-10-ol **3b**

White solid, ^1^H NMR (400 MHz, CDCl_3_): δ 8.70 (d, *J* = 8.2 Hz, 1H), 8.06 (d, *J* = 1.4 Hz, 1H), 7.99 (d, *J* = 7.8 Hz, 1H), 7.82 (d, *J* = 9.2 Hz, 1H), 7.71 (t, *J* = 7.6 Hz, 1H), 7.59 (d, *J* = 8.2 Hz, 2H), 7.48–7.50 (m, 4H), 7.32 (d, *J* = 8.7 Hz, 1H), 7.00 (dd, *J* = 8.7, 2.3 Hz, 1H), 4.94 (s, 1H).^61^

##### 3.2.1.3 9-Methyl-7-(*p*-tolyl)-7*H*-benzo­[*c*]­carbazol-10-ol **3c**

White solid, ^1^H NMR (400 MHz, CDCl_3_): δ 8.70 (d, *J* = 8.2 Hz, 1H), 8.01 (s, 1H), 7.98 (d, *J* = 8.2 Hz, 1H), 7.78 (d, *J* = 8.7 Hz, 1H), 7.69 (t, *J* = 7.6 Hz, 1H), 7.50 (d, *J* = 8.7 Hz, 1H), 7.44–7.48 (m, 5H), 7.23 (s, 1H), 4.79 (s, 1H), 2.52 (s, 3H), 2.43 (s, 3H).^61^

##### 3.2.1.4 2-Bromo-7-(*p*-tolyl)-7*H*-benzo­[*c*]­carbazol-10-ol **3d**

Yellow solid, ^1^H NMR (400 MHz, CDCl_3_) δ 8.82 (d, *J* = 1.8 Hz, 1H), 8.00 (d, *J* = 2.3 Hz, 1H), 7.83 (d, *J* = 8.7 Hz, 1H), 7.74 (d, *J* = 9.2 Hz, 1H), 7.54 (dd, *J* = 8.5, 2.1 Hz, 1H), 7.50 (d, *J* = 9.2 Hz, 1H), 7.42 (s, 4H), 7.34 (d, *J* = 8.7 Hz, 1H), 7.01 (dd, *J* = 8.9, 2.5 Hz, 1H), 4.88 (s, 1H), 2.51 (s, 3H); ^13^C NMR (101 MHz, CDCl_3_) δ 150.39, 139.84, 138.12, 135.69, 134.60, 131.10, 130.81, 130.69, 127.72, 127.55, 127.19, 126.27, 125.52, 123.96, 121.43, 114.05, 113.98, 112.43, 111.35, 107.09, 21.42; HRMS (APCI): calcd for C_23_H_17_BrNO^+^: *m*/*z* 402.0488 [M + H]^+^, found 402.0494; IR (KBr): 3411, 3074, 3022, 2987, 1681, 1503, 1469, 1169, 804, 766 cm^–1^.

##### 3.2.1.5 10-Hydroxy-7-(*p*-tolyl)-7*H*-benzo­[*c*]­carbazole-3-carbonitrile **3e**

Yellow solid, ^1^H NMR (600 MHz, (CD_3_)_2_CO): δ 8.87 (d, *J* = 8.2 Hz, 1H), 8.54 (s, 1H), 8.34 (s, 1H), 8.09 (s, 1H), 8.00 (d, *J* = 8.9 Hz, 1H), 7.96 (d, *J* = 8.2 Hz, 1H), 7.64 (d, *J* = 8.9 Hz, 1H), 7.51–7.58 (m, 4H), 7.34 (d, *J* = 8.9 Hz, 1H), 7.11 (d, *J* = 8.2 Hz, 1H), 2.51 (s, 3H).^61^

#### 3.2.2 Synthesis of 11-(2-Hydroxynaphthalen-1-yl)-7-(*p*-tolyl)-7*H*-benzo­[*c*]­carbazol-10-ol Derivatives **6a** and **6b**

##### 3.2.2.1 Method A

A solution of benzo­[*c*]­carbazol-10-ol derivatives **3**, 2-naphthol derivatives **4** (1.0 equiv), and tetrabutylammonium hexafluorophosphate (0.1 M) in CH_2_Cl_2_ (0.02 M) was transferred into an undivided electrolysis cell equipped with two Pt electrodes (1.3 × 1.5 cm^2^) connected to a DC power supply. At room temperature, a constant current electrolysis with a current density of 0.4 mA/cm^2^ was performed. After 15 h (1.72 F/mol) of stirring, the electrolysis was stopped. The crude products were purified by column chromatography (SiO_2_, EtOAc/*n*-hexane), affording the desired 11-(2-hydroxynaphthalen-1-yl)-7-(*p*-tolyl)-7*H*-benzo­[*c*]­carbazol-10-ol **6** as yellow solids in 65–72% yields, along with minor traces of the corresponding helicenes and dehydrohelicenes.^59^

##### 3.2.2.2 Method B

A reaction vessel was charged with benzo­[*c*]­carbazol-10-ol derivatives **3**, 2-naphthol derivatives **4** (1.0 equiv), mononuclear vanadium catalyst *rac*-**5** (10 mol %),^58^ LiCl (3.0 equiv), and dioxane (0.1 M) under air at 30 °C. The reaction mixture was stirred for 96 h and then filtered through a short pad of silica gel, and the solvent was evaporated *in vacuo*. The resulting residue was purified by column chromatography (SiO_2_, EtOAc/*n*-hexane), yielding the desired diols **6** in 77–84% yields.^61^

##### 3.2.2.3 11-(2-Hydroxy-7-methoxynaphthalen-1-yl)-7-(*p*-tolyl)-7*H*-benzo­[*c*]­carbazol-10-ol **6a**

^1^H NMR (600 MHz, CDCl_3_): δ 8.01 (d, *J* = 8.9 Hz, 1H), 7.88 (d, *J* = 8.9 Hz, 1H), 7.76 (d, *J* = 6.9 Hz, 1H), 7.72 (d, *J* = 8.9 Hz, 1H), 7.52 (d, *J* = 9.3 Hz, 1H), 7.47–7.51 (m, 4H), 7.43 (d, *J* = 8.9 Hz, 1H), 7.29 (d, *J* = 8.9 Hz, 1H), 7.20 (d, *J* = 8.9 Hz, 1H), 7.14 (ddd, *J* = 7.6, 6.9, 1.4 Hz, 1H), 7.06 (dd, *J* = 8.9, 2.7 Hz, 1H), 6.88 (d, *J* = 2.7 Hz, 1H), 6.76 (d, *J* = 8.9 Hz, 1H), 6.67 (ddd, *J* = 7.6, 6.9, 1.4 Hz, 1H), 5.25 (s, 1H), 4.95 (s, 1H), 3.61 (s, 3H), 2.55 (s, 3H).^61^

##### 3.2.2.4 2-Bromo-11-(7-bromo-2-hydroxynaphthalen-1-yl)-7-(*p*-tolyl)-7*H*-benzo­[*c*]­carbazol-10-ol **6b**

^1^H NMR (400 MHz, CDCl_3_) δ 8.13 (d, *J* = 8.7 Hz, 1H), 7.86 (d, *J* = 8.7 Hz, 1H), 7.66–7.68 (m, 2H), 7.61 (d, *J* = 8.7 Hz, 1H), 7.41–7.54 (m, 8H), 7.30 (d, *J* = 8.7 Hz, 1H), 7.24 (dd, *J* = 8.7, 1.8 Hz, 1H), 6.86 (d, *J* = 1.4 Hz, 1H), 5.29 (s, 1H), 4.75 (s, 1H), 2.55 (s, 3H); ^13^C NMR (101 MHz, CDCl_3_) δ 154.02, 149.46, 140.76, 138.88, 136.51, 134.81, 134.11, 133.03, 130.90, 130.65, 130.44, 128.39, 128.36, 128.04, 128.01, 127.49, 126.83, 126.14, 123.32, 122.73, 120.51, 118.36, 114.87, 114.72, 114.17, 113.45, 112.29, 109.16, 21.50 (Two carbons overlapped); DEPT-135 NMR (101 MHz, CDCl_3_) δ 133.04, 130.90, 130.65, 130.45, 128.39, 128.02, 127.49, 126.83, 126.15, 118.36, 114.88, 113.45, 112.29, 21.50 (One carbon overlapped); HRMS (APCI): calcd for C_33_H_22_Br_2_NO_2_^+^: *m*/*z* 622.0012 [M + H]^+^, found 622.0017; IR (KBr): 3477, 3354, 3022, 3012, 2890, 1654, 1521, 1302, 1212, 809 cm^–1^.

#### 3.2.3 Synthesis of 7,7’-(1,4-phenylene)­bis­(7*H*-benzo­[*c*]­carbazol-10-ol) **7**

To a solution of *N* ^1^,*N* ^4^-di­(naphthalen-2-yl)­benzene-1,4-diamine **1b** and *p*-benzoquinone **2** (2.5 equiv) in dry toluene (0.05 M), orthophosphoric acid (2.0 equiv) was added dropwise. The reaction mixture was stirred at 50 °C for 5 h under a nitrogen atmosphere until completion. The reaction was then quenched with water and extracted with EtOAc, and the combined organic extracts were dried over Na_2_SO_4_ and concentrated under reduced pressure. The crude product was purified by column chromatography (SiO_2_, EtOAc/*n*-hexane), affording 7,7’-(1,4-phenylene)­bis­(7*H*-benzo­[*c*]­carbazol-10-ol) **7** as a white solid in 54% yield.^1^H NMR (600 MHz, (CD_3_)_2_CO): δ 8.80 (d, *J* = 8.2 Hz, 2H), 8.31 (s, 2H), 8.17 (d, *J* = 2.1 Hz, 2H), 8.07 (d, *J* = 8.2 Hz, 2H), 7.92–7.96 (m, 6H), 7.75–7.78 (m, 4H), 7.58 (d, *J* = 8.9 Hz, 2H), 7.50 (dd, *J* = 8.3, 6.9 Hz, 2H), 7.14 (dd, *J* = 8.6, 2.4 Hz, 2H).^56^

#### 3.2.4 Synthesis of 10-Methoxy-7*H*-benzo­[*c*]­carbazol-10-ol Derivatives **10a**–**10c**

##### 3.2.4.1 10-Methoxy-7*H*-benzo­[*c*]­carbazole **10a**

A mixture of 2-bromo-4-methoxy-1-nitrobenzene **8**, naphthalen-1-boronic acid **9** (1.3 equiv), Pd­(OAc)_2_ (2.0 mol %), PPh_3_ (2.5 equiv), and K_2_CO_3_ (2.0 equiv) in *o*-DCB (0.5 M) was heated to 180 °C. The reaction mixture was stirred at this temperature for 12 h, then filtered through Celite, and concentrated *in vacuo*. The resulting residue was purified by column chromatography (SiO_2_, EtOAc/*n*-hexane), yielding 10-methoxy-7*H*-benzo­[*c*]­carbazole **10a** as a deep green solid in 71% yield.^1^H NMR (400 MHz, CDCl_3_): δ 8.72 (d, *J* = 8.55 Hz, 1H), 8.35 (s, 1H), 8.00–8.05 (m, 2H), 7.86 (d, *J* = 8.55 Hz, 1H), 7.73 (td, *J* = 7.71, 1.26 Hz, 1H), 7.64 (d, *J* = 8.55 Hz, 1H), 7.46–7.52 (m, 2H), 7.15 (dd, *J* = 8.81, 2.27 Hz, 1H), 4.04 (s, 3H).^95^

##### 3.2.4.2 10-Methoxy-7-methyl-7*H*-benzo­[*c*]­carbazole **10b**

A mixture of compound **10a**, iodomethane (1.2 equiv), and NaH (1.1 equiv) in DMF (0.2 M) was stirred at 25 °C for 3 h. After the reaction was complete, the mixture was filtered through Celite and extracted with EtOAc. The combined organic layers were dried over anhydrous Na_2_SO_4_, filtered, and concentrated under reduced pressure. The resulting residue was purified by column chromatography (SiO_2_, EtOAc/*n*-hexane), yielding 10-methoxy-7-methyl-7*H*-benzo­[*c*]­carbazole **10b** as a yellow solid in 69% yield.^1^H NMR (400 MHz, CDCl_3_) δ 8.71 (d, *J* = 8.3 Hz, 1H), 8.08–7.96 (m, 2H), 7.88 (d, *J* = 8.9 Hz, 1H), 7.70 (t, *J* = 7.3 Hz, 1H), 7.62 (d, *J* = 8.9 Hz, 1H), 7.50–7.41 (m, 2H), 7.17 (dd, *J* = 8.8, 2.3 Hz, 1H), 4.03 (s, 3H), 3.96 (s, 3H).^96^

##### 3.2.4.3 10-Methoxy-7-(*p*-tolyl)-7*H*-benzo­[*c*]­carbazole **10c**

A mixture of compound **10a**, 4-iodotoluene (1.2 equiv), CuI (10 mol %), 1,10-phenanthroline (10 mol %), and K_2_CO_3_ (1.2 equiv) in DMF (0.2 M) was stirred at 160 °C for 22 h. After cooling, the reaction mixture was filtered through Celite and extracted with EtOAc. The combined organic layers were dried over anhydrous Na_2_SO_4_, filtered, and concentrated *in vacuo*. The residue was purified by column chromatography (SiO_2_, EtOAc/*n*-hexane), affording 10-methoxy-7-(*p*-tolyl)-7*H*-benzo­[*c*]­carbazole **10c** as a white solid in 77% yield. ^1^H NMR (600 MHz, CDCl_3_) δ 8.76 (d, *J* = 8.2 Hz, 1H), 8.09 (d, *J* = 2.7 Hz, 1H), 7.99 (d, *J* = 7.6 Hz, 1H), 7.80 (d, *J* = 8.2 Hz, 1H), 7.73 (td, *J* = 7.6, 1.4 Hz, 1H), 7.53 (d, *J* = 8.9 Hz, 1H), 7.48 (t, *J* = 7.6 Hz, 1H), 7.42–7.46 (m, 4H), 7.39 (d, *J* = 8.9 Hz, 1H), 7.09 (dd, *J* = 8.8, 2.9 Hz, 1H), 4.04 (s, 3H), 2.51 (s, 3H). ^13^C NMR (151 MHz, CDCl_3_) δ 154.78, 139.35, 137.87, 135.59, 134.92, 130.64, 130.06, 129.35, 127.59, 127.39, 127.04, 124.20, 123.17, 123.04, 115.14, 113.55, 112.06, 111.15, 105.23, 99.34, 56.47, 21.43. HRMS (APCI): calcd for C_24_H_20_NO^+^: *m*/*z* 338.1539 [M + H]^+^, found 338.1541; IR (KBr): 3032, 2956, 2876, 1724, 1604, 1570, 1420, 1405, 1219, 715 cm^–1^.

#### 3.2.5 Synthesis of 3,6-Dibromo-9*H*-carbazole **12**

To a stirred solution of 9*H*-carbazole **11** in DMF (0.1 M), NBS (2.2 equiv) was gradually added at room temperature. Following the addition, the mixture was stirred at room temperature for 24 h. The resulting solution was then poured into 250 mL of cold water and extracted with DCM (3 × 200 mL). The combined organic layers were dried over anhydrous Na_2_SO_4_ and concentrated *in vacuo*. After purification by column chromatography (SiO_2_, EtOAc/*n*-hexane), a white solid of **12** was obtained in 74% yield.^1^H NMR (400 MHz, (CD_3_)_2_CO): δ 10.68 (s, 1H), 8.36 (d, *J* = 1.4 Hz, 2H), 7.49–7.56 (m, 4H).^97^

### 3.3 Screening the Compounds for Antimicrobial Activity

Antimicrobial activity was evaluated using the agar well diffusion method against a panel of standard pathogenic microorganisms, including Gram-positive bacteria (*S. aureus* ATCC-6538), Gram-negative bacteria (*E. coli* ATCC-12345, *P. aeruginosa* ATCC-9027, *K. pneumoniae* ATCC-33495), and the yeast (*C. albicans* ATCC-10231). Microbial inocula were prepared to achieve a turbidity equivalent to 0.5 McFarland standard (approximately 1.5 × 10^8^ CFU/mL) and uniformly spread onto the surface of Mueller–Hinton agar plates using sterile cotton swabs. The swabbing was conducted in three directions, each rotated by 60°, to ensure even distribution of the microbial lawn. Agar wells, each with a diameter of 6 mm, were created in the inoculated plates and subsequently loaded with 80 μL of test solutions (1 mg/mL concentration of each compound). A negative control consisting of the same volume of dimethyl sulfoxide (DMSO) was included to confirm the absence of antimicrobial effects from the solvent. The plates were incubated at 37 °C for 24 h for bacterial cultures and 48 h for yeast cultures. Postincubation, the diameters of the inhibition zones around the wells were measured to assess the antimicrobial efficacy of the compounds under investigation (see the Supporting Information).

### 3.4 Determination of the Minimum Inhibitory Concentrations (MICs)

The MICs of compounds exhibiting antibacterial activity, defined by an inhibition zone diameter of ≥7 mm, were evaluated using a modified broth microdilution technique following Clinical and Laboratory Standards Institute (CLSI) guidelines with minor modifications.^98^ Stock solutions of the carbazoles were initially prepared at a concentration of 2560 μg/mL. From these, working solutions were generated by diluting 100 μL of stock solution into 900 μL of sterile nutrient broth. A microbial inoculum was prepared to match the turbidity of a 0.5 McFarland standard and subsequently diluted 1:100 in sterile nutrient broth to achieve a final density of 10^5^–10^6^ CFU/mL. The broth microdilution assay was performed in U-bottom 96-well microtiter plates. 2-fold serial dilutions of the compounds were prepared by mixing 100 μL of each working solution with an equal volume of nutrient broth, covering a concentration range of 0.25–128 μg/mL. Each well was then inoculated with 100 μL of the prepared microbial suspension. Sterility controls (nutrient broth without inoculum) and growth controls (inoculated wells without test compounds) were included in each assay. Amoxicillin and imipenem/cilastatin served as reference standards for comparison. The microtiter plates were incubated at 37 °C for 18 h, after which bacterial growth was assessed visually by checking for turbidity in the wells. The MIC was defined as the lowest concentration of a compound that completely inhibited visible growth (i.e., no turbidity). MICs were determined in two independent experiments; both runs produced identical values (no interassay variability).

### 3.5 Determination of the Minimum Bactericidal Concentrations (MBCs)

For minimum bactericidal concentration (MBC) determination of compound **3a**, 10 μL of the suspension from the well represents the MIC value, from the MIC experiment, and four wells above this value were spread on agar plates. The plates were incubated at 37 °C for 24 h. The MBC is the lowest dilution that prevents the growth of the microorganism on the agar plates. The MBC/MIC ratio was also calculated to determine the bacteriostatic or bactericidal effect of compound **3a**.

### 3.6 Scanning Electron Microscopy (SEM)

The morphological alterations in *S. aureus* (ATCC-6538) and *P. aeruginosa* (ATCC-9027) before and after exposure to the compounds **3a** and **3c**—identified as having the lowest MIC—were analyzed using SEM. Bacterial cultures were initiated by inoculating nutrient broth with *S. aureus* and *P. aeruginosa*, followed by overnight incubation at 37 °C. The resulting suspensions were standardized to a turbidity equivalent to a 0.5 McFarland standard. Subsequently, aliquots were treated with compounds **3a** and **3c** at their respective MICs for 24 h. Untreated bacterial suspensions adjusted to the same turbidity were incubated under identical conditions to serve as negative controls. Post-treatment, bacterial cells were collected by centrifugation at 6000 rpm for 10 min at 4 °C. The cell pellets were then fixed overnight in 4% glutaraldehyde at 4 °C to preserve cellular structures. After fixation, the cells were rinsed twice with phosphate-buffered saline (PBS, pH 7.4) to remove residual fixative. Dehydration was carried out sequentially using an ascending ethanol series (30%, 50%, 70%, 80%, 90%, and absolute ethanol). Finally, the processed samples were mounted and subjected to SEM imaging to visualize and compare the morphological features of treated and untreated bacterial cells, providing insight into the structural effects of compounds **3a** and **3c**.

### 3.7 Molecular Modeling

#### 3.7.1 Docking Study

Using ChemDraw, the carbazole derivatives (**3a** and **10c**) were manually drawn, converted into 3D structures, and energy-minimized within the MOE platform (Molecular Operating Environment, Chemical Computing Group Inc.). The crystal structures of penicillin-binding proteins (PBPs) from *S. aureus* (PBP3, PDB ID: 3VSL) and *P. aeruginosa* (PBP3, PDB ID: 3PBO) were retrieved from the Protein Data Bank. Protein structures were prepared using the Protonate3D module, followed by potential fixing, structure correction, energy minimization, and removal of cocrystallized ligands and water molecules. Docking simulations were carried out using MOE’s Dock module, applying the Triangle Matcher placement method and London dG scoring function, followed by pose refinement using the GBVI/WSA dG rescoring method. Validation of the docking protocol confirmed the reliability of the docking setup, as redocking of the cocrystallized ligands into the respective active sites produced binding modes consistent with their crystallographic positions. The minimized carbazole derivatives were docked into the active sites of both PBPs, and the best-ranked poses were further analyzed.

#### 3.7.2 Molecular Dynamics Simulation

Molecular dynamics (MD) simulations for compound **3a** in complex with PBPs (PDB ID: 3VSL) were initiated from its most favorable docked pose. All-atom simulations were performed using the OPLS_2005 force field throughout the calculations. The protein–ligand complex was embedded in an orthorhombic periodic box with a 10 Å buffer and solvated with explicit TIP3P water molecules. System neutrality was achieved by adding appropriate counterions, followed by the inclusion of 0.15 M salt to mimic physiological conditions. Prior to the production run, the system underwent a series of energy minimizations and short equilibration phases under both NVT and NPT ensembles, with 12 ps runs applied to gradually relax the system. The final production simulation was conducted for 100 ns under the NPT ensemble, with temperature and pressure maintained at 300 K and 1 atm, respectively. The integration time step was set at 10 ps, applying a 9 Å cutoff for short-range van der Waals interactions, while long-range electrostatics were computed using the particle mesh Ewald (PME) method. Resulting trajectories were analyzed and visualized in Maestro using the Desmond interaction diagram tools.

### 3.8 DFT Calculations

DFT calculations were conducted using the Gaussian 09 and Gaussian 16 software suites.^90,99^ Molecular geometries of the model compounds were optimized employing the B3LYP functional with 6-311G, 6-31+G­(d,p), and 6-311+G­(d,p) basis sets. Frequency calculations confirmed all optimized geometries as true minima on the potential energy surface. Optimization procedures adhered to the default convergence criteria outlined in the Gaussian program documentation.^100^ Time-Dependent DFT (TD-DFT) computations were carried out at the B3LYP/6-311G and B3LYP/6-311+G­(d,p) levels, incorporating solvent effects using the Polarizable Continuum Model (PCM) with chloroform as the solvent. Structural optimizations were performed without imposing any symmetry constraints. The nucleus-independent chemical shift (NICS) indices were calculated in the center of each ring NICS(0) and 1 Å above/below the center NICS(1) within gauge-independent atomic orbital (GIAO) approximation at the B3LYP/6-311+G­(d,p) level of theory. For the anisotropy of the induced current density (AICD) simulations, the AICD-3.0.4 software was used. Additional computational methodologies and parameters can be found in the Supporting Information.

## 4 Conclusion

In this study, a series of 7*H*-benzo­[*c*]­carbazol-10-ol derivatives were designed and synthesized through an efficient one-pot approach, featuring various substituents that modulate their biological activity and photophysical properties. These compounds demonstrated selective antimicrobial activity against *S. aureus* and *P. aeruginosa*, with **3a** and **3c** outperforming standard references (MIC = 8 μg/mL). SEM analysis confirmed cell wall damage, blebbing, and cytoplasmic leakage, indicating a possible bactericidal effect through inhibition of cell wall synthesis, which was further confirmed by MBC analysis. Molecular docking with PBPs highlighted the role of hydroxyl groups in stabilizing the ligand within the active site. Additionally, their optical and redox properties, including favorable HOMO–LUMO transitions and excited-state redox potentials, suggest their applicability as efficient metal-free photocatalysts. To the best of our knowledge, this is the first comprehensive antimicrobial, structural, optical, and redox profiling of this scaffold class. These dual-function materials represent a unique contribution to both antimicrobial research and sustainable materials chemistry, opening new avenues for multifunctional heterocyclic design.

## Supplementary Material



## Notes

^1^H and ^13^C NMR spectra for all compounds; agar-well diffusion assay results against *S. aureus*, *C. albicans*, *E. coli*, *K. pneumoniae*, and *P. aeruginosa* (Figure S1); antibacterial/antifungal assay tables (S1–S2); SEM images (Figures S2–S7); bioavailability prediction (Table S3, Figure S8); additional docking studies (Figures S9–S14); frontier molecular orbitals of **3a** (Tables S4–S5); simulated UV–Vis spectra (Figure S15); TD-DFT results of electronic transitions (Tables S6–S28); DFT-calculated redox potentials of carbazoles (Tables S29–S30); aromaticity comparisons; Cartesian coordinates (PDF)

## Abbreviations

SEM: Scanning electron microscopy
*P. aeruginosa*: *Pseudomonas aeruginosa*
*S. aureus*: *Staphylococcus aureus*
*K. pneumoniae*: *Klebsiella pneumoniae*
*E. coli*: *Escherichia coli*
*C. albicans*: *Candida albicans*
MIC: Minimum inhibitory concentration
MOE: Molecular operating environment
PBP: Penicillin-binding protein
PDB: Protein data bank
NBS: *N*-Bromosuccinimide
Pd­(OAc)_2_: Palladium­(II) acetate
*o*-DCB: *o*-Dichlorobenzene
ε: Molar absorption coefficient
CV: Cyclic voltammetry
DFT: Density functional theory
MeCN: Acetonitrile
Bu_4_NPF_6_: Tetrabutylammonium hexafluorophosphate
Fc/Fc^+^: Ferrocene/ferrocenium
SCE: Saturated calomel electrode
SHE: Standard hydrogen electrode
B3LYP: A functional used in DFT calculations
6–31+G­(d,p): A specific basis set for DFT calculations
CPCM: Conductor-like polarizable continuum model
NMR: Nuclear magnetic resonance
APCI-MS: Atmospheric pressure chemical ionization mass spectrometry
FT-IR: Fourier transform infrared spectroscopy
TLC: Thin-layer chromatography
SiO_2_: Silica gel
EtOAc: Ethyl acetate
DC: Direct current
MHz: Megahertz
ppm: Parts per million
NICS: Nucleus-independent chemical shift
AICD: Anisotropy of the induced current density
HOMA: Harmonic oscillator model of aromaticity
