# Supplementary material for: Antimicrobial, Structural, Optical, and Redox Profiling of 7H‑Benzo[c]carbazol-10-ol Derivatives: An Integrated Experimental and Computational Study
Source: ACS Omega. 2025 Nov 21;10(48):59183–99. doi: 10.1021/acsomega.5c08465 (PMC12771225; doi:10.1021/acsomega.5c08465)
Supplement: Supplementary file 1 [file ao5c08465_si_001.pdf]

Supporting Information

Antimicrobial, Structural, Optical, and Redox Profiling of 7*H*-Benzo[*c*]carbazol-10-ol Derivatives: An Integrated Experimental and Computational Study

Mohamed S. H. Salem [1,2,*], Manar El Samak [3], Yasmine M. Abdel Aziz [2], Mohamed H. Aboutaleb [4], Sharvari Patil [1], Tin Zar Aye [1], Tarek S. Ibrahim [5,6] and Shinobu Takizawa [1,*]

[1] SANKEN, The University of Osaka, 8−1 Mihogaoka, Ibaraki, Osaka 567−0047, JAPAN
[2] Pharmaceutical Organic Chemistry Department, Faculty of Pharmacy, Suez Canal University, Ismailia 41522, Egypt
[3] Department of Microbiology and Immunology, Faculty of Pharmacy, Suez Canal University, Ismailia 41522, Egypt
[4] Department of Pharmaceutical Chemistry, Faculty of Pharmacy, Horus University-Egypt, New Damietta, Egypt
[5] Department of Pharmaceutical Chemistry, Faculty of Pharmacy, King Abdulaziz University, Jeddah 21589, Saudi Arabia
[6] Department of Pharmaceutical Organic Chemistry, Faculty of Pharmacy, Zagazig University, Zagazig 44519, Egypt
* Correspondence: mohamedsalem43@sanken.osaka-u.ac.jp, taki@sanken.osaka-u.ac.jp; Tel.: (+81−6−6879−8469)

Contents

1. **Supplementary Note 1:** *Antibacterial and antifungal studies of carbazole derivatives* 2
2. **Supplementary Note 2:** *Scanning electron microscopy (SEM) study* 4
3. **Supplementary Note 3:** *Bioavailability prediction* 10
3. **Supplementary Note 4:** *Molecular Docking* 12
4. **Supplementary Note 5:** *Molecular orbitals of **3a*** 15
5. **Supplementary Note 6:** *Simulated UV-Vis spectra* 17
6. **Supplementary Note 7:** *Summary of the TD-DFT calculation results* 21
7. **Supplementary Note 8:** *Theoretical prediction of the pKa values of **3a** and its radical cation [**3a**]$^{•+}$* 47
8. **Supplementary Note 9:** *DFT−calculated redox behavior of reported carbazole derivatives* 48
9. **Supplementary Note 10:** *Comparative analysis of the aromaticity of various carbazoles* 51
10. **Supplementary Note 11:** *Cartesian coordinates* 55
11. **Supplementary Note 12:** *NMR Spectra* 237
12. **Supplementary Note 13:** *References* 242

1. Supplementary Note 1: *Antibacterial and antifungal studies of carbazole derivatives*

Table S1. Inhibition zone diameters showing antibacterial and antifungal activities of carbazoles.

Compound number	Inhibition zone diameter (mm)				
	S. aureus ATCC 6538	*P. Aeruginosa* ATCC 9027	*K. Pneumoniae* ATCC 33495	*E. coli* ATCC 12345	*C. albicans* ATCC 10231
**3a**	8	11	-	-	11
**3b**	14	10	-	-	-
**3c**	10	10	-	-	-
**3d**	-	-	-	-	-
**3e**	17	17	-	-	-
**6a**	-	-	-	-	-
**6b**	-	-	10	10	12
**7**	15	14	-	-	-
**10a**	-	13	-	-	18
**10b**	-	-	-	-	17
**10c**	-	-	-	-	12
**11**	-	-	-	-	16
**12**	8	14	-	-	-

(-) indicates no activity.

Table S2. Minimum inhibitory concentration MIC (μg/ml) of carbazoles with antibacterial activity against standard pathogenic bacteria.

Compound number	Minimum Inhibitory Concentration (MIC) (μg/ml)			
	S. aureus ATCC 6538	*P. Aeruginosa* ATCC 9027	*K. Pneumoniae* ATCC 33495	*E. coli* ATCC 12345
**3a**	8	8	-	-
**3b**	32	16	-	-
**3c**	8	8	-	-
**3e**	16	16	-	-
**6b**	-	-	>128	>128
**7**	64	64	-	-
**10a**	-	>128	-	-
**12**	>128	>128	-	-
**Amoxicillin**	<0.25	16	-	-
**Ceftazidime**	-	>128	-	-
**Imipenem/ cilastatin**	-	1	-	-

(-) indicates that the compound was not tested for MIC because it did not show antimicrobial activity against the illustrated bacteria in the well diffusion assay.

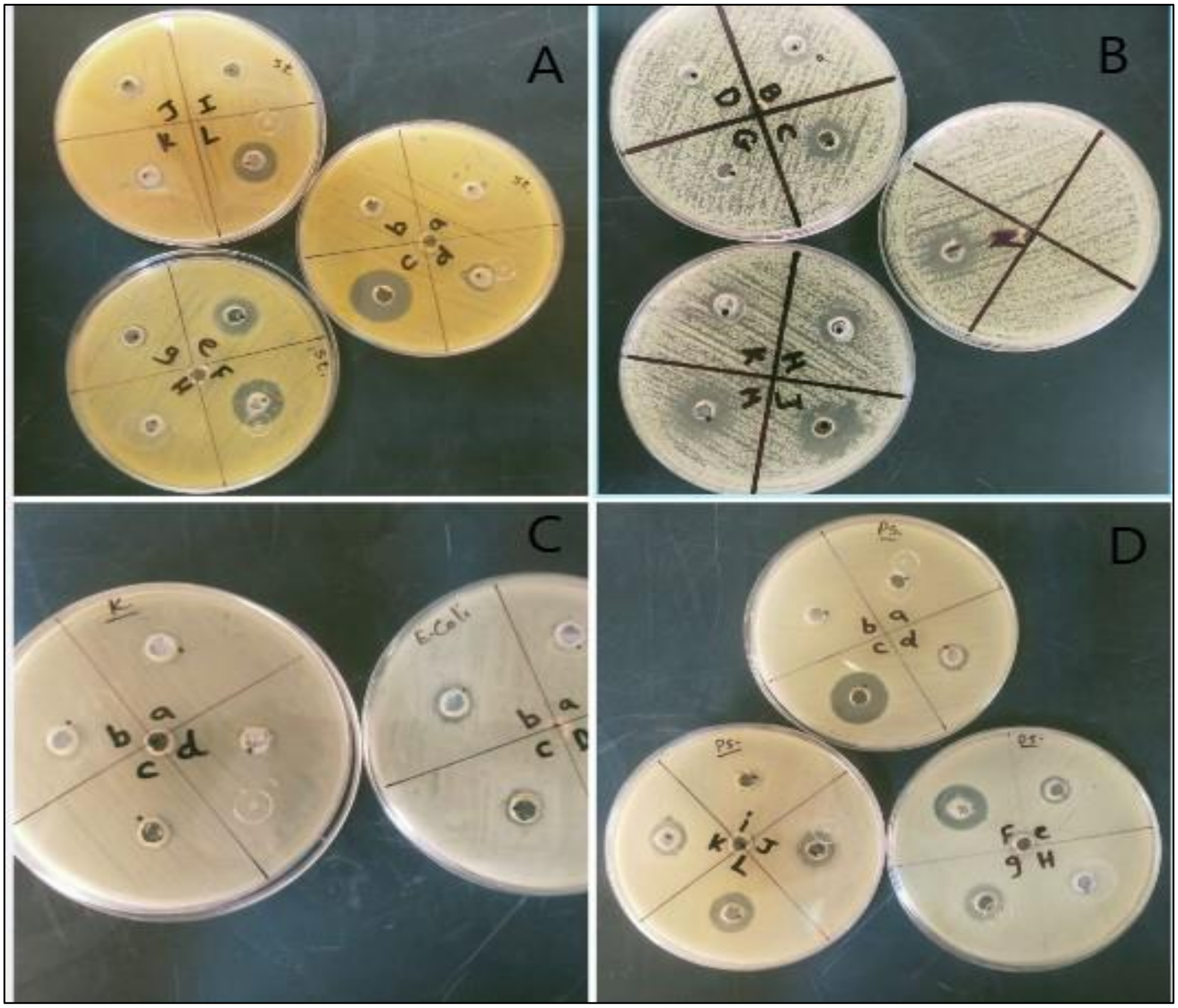

Figure S1. (A) Compounds showing antibacterial activity against *Staphylococcus aureus* ATCC 6538. (B) Compounds showing antifungal activity against *Candida albicans* ATCC 10231. (C) Compounds showing antibacterial activity against *Escherichia coli* ATCC 12345 (E. coli) and *Klebsiella Pneumoniae* ATCC 33495 (K). (D) Compounds showing antibacterial activity against *Pseudomonas Aeruginosa* ATCC 9027.

3a	3b	3c	3d	3e	6a	6b	7	10a	10b	10c	11	12
k	e	d	a	f	i	b	l	j	n	k	m	g

2. **Supplementary Note 2:** *Scanning electron microscopy (SEM) study*

Figure S2. Untreated *S. aureus*

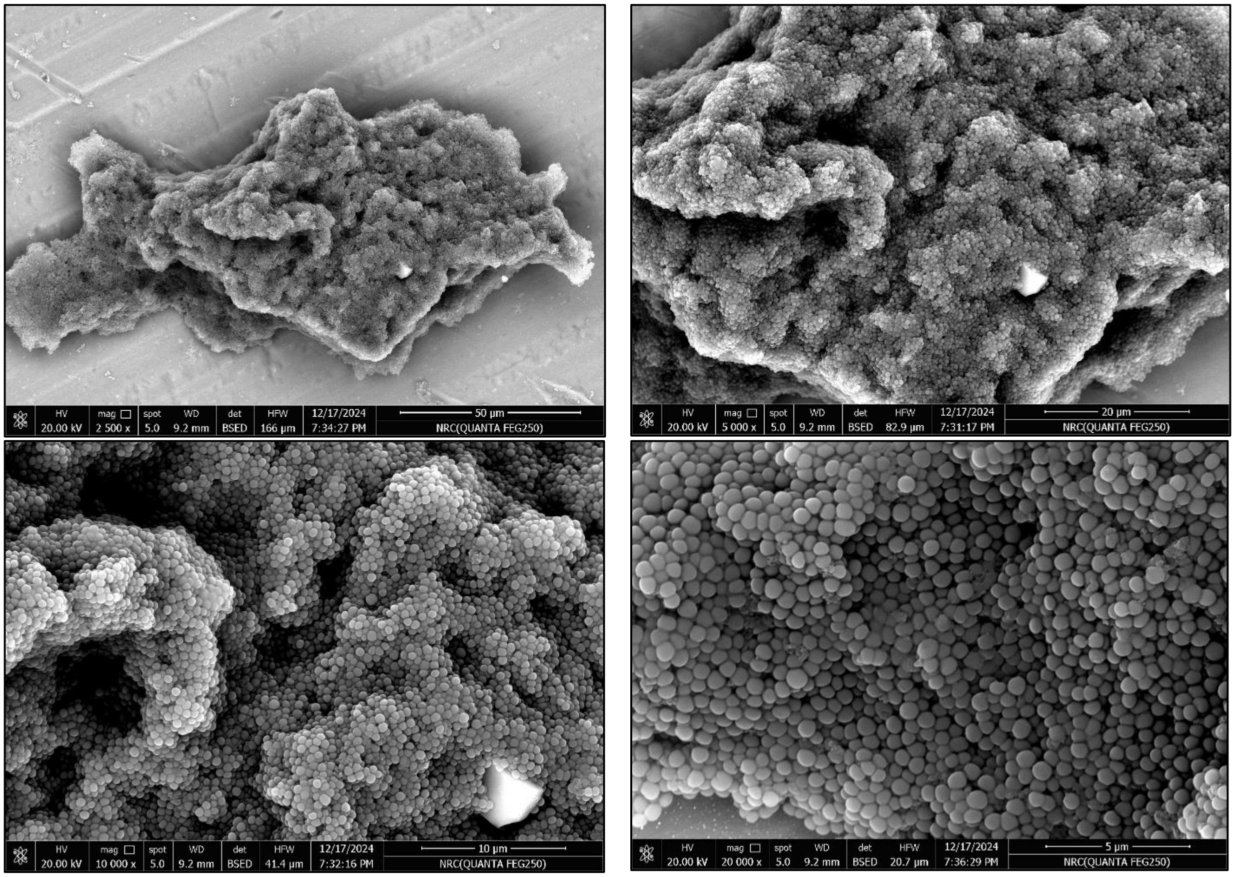

Figure S3. *S. aureus* after treatment with compound **3a** (8 µg/ml)

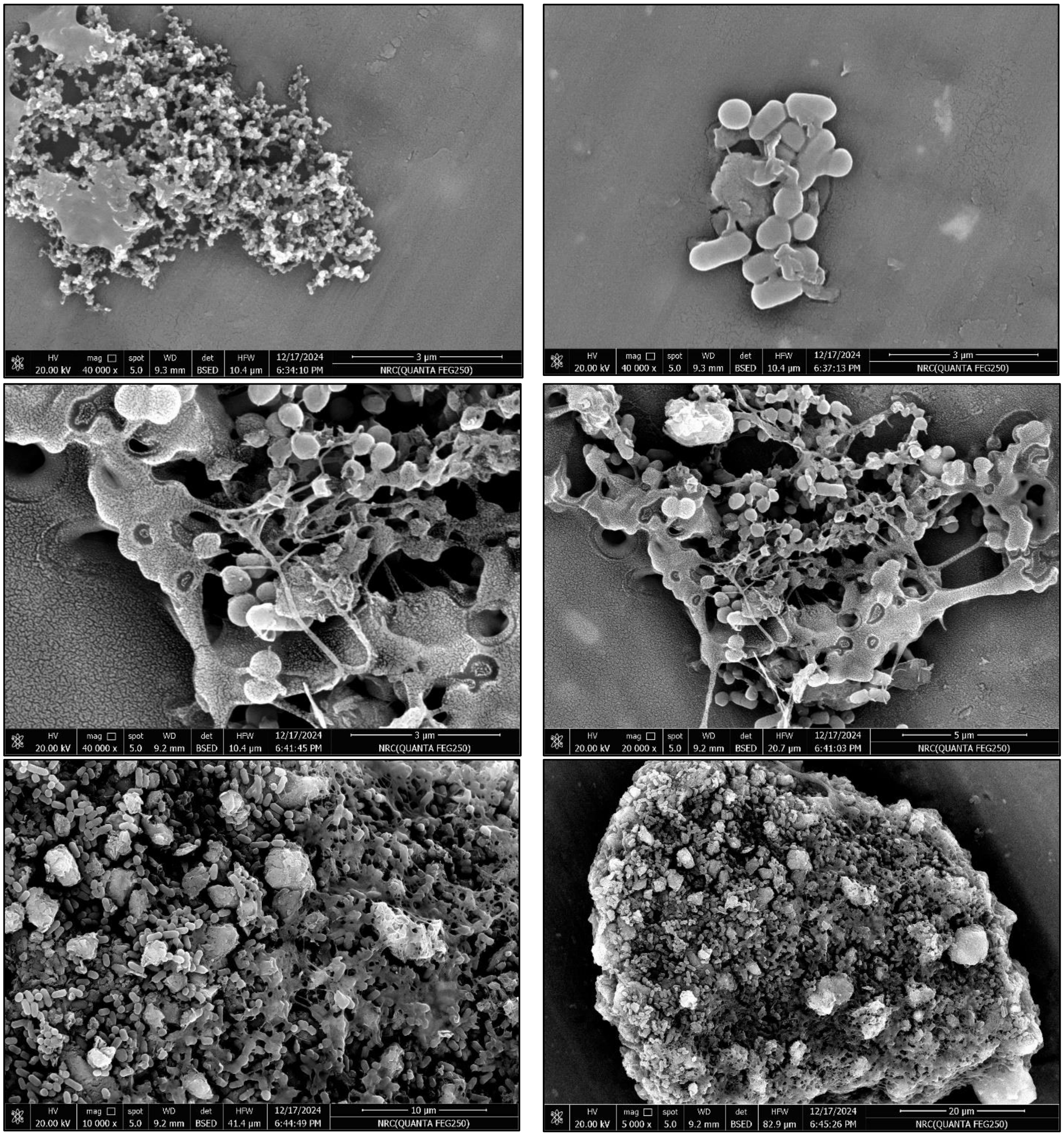

Figure S4. *S. aureus* after treatment with compound **3c** (8 µg/ml).

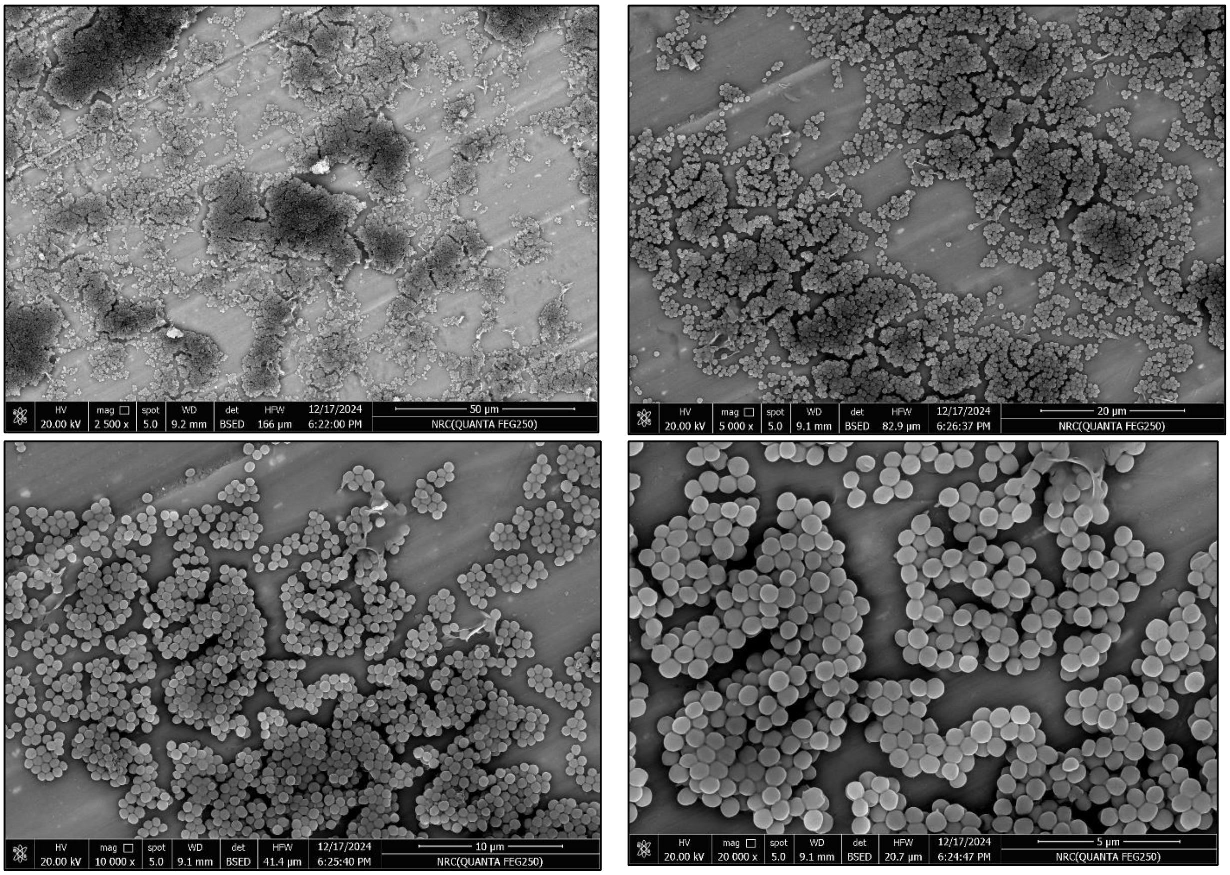

Figure S5. Untreated *P. aeruginosa*

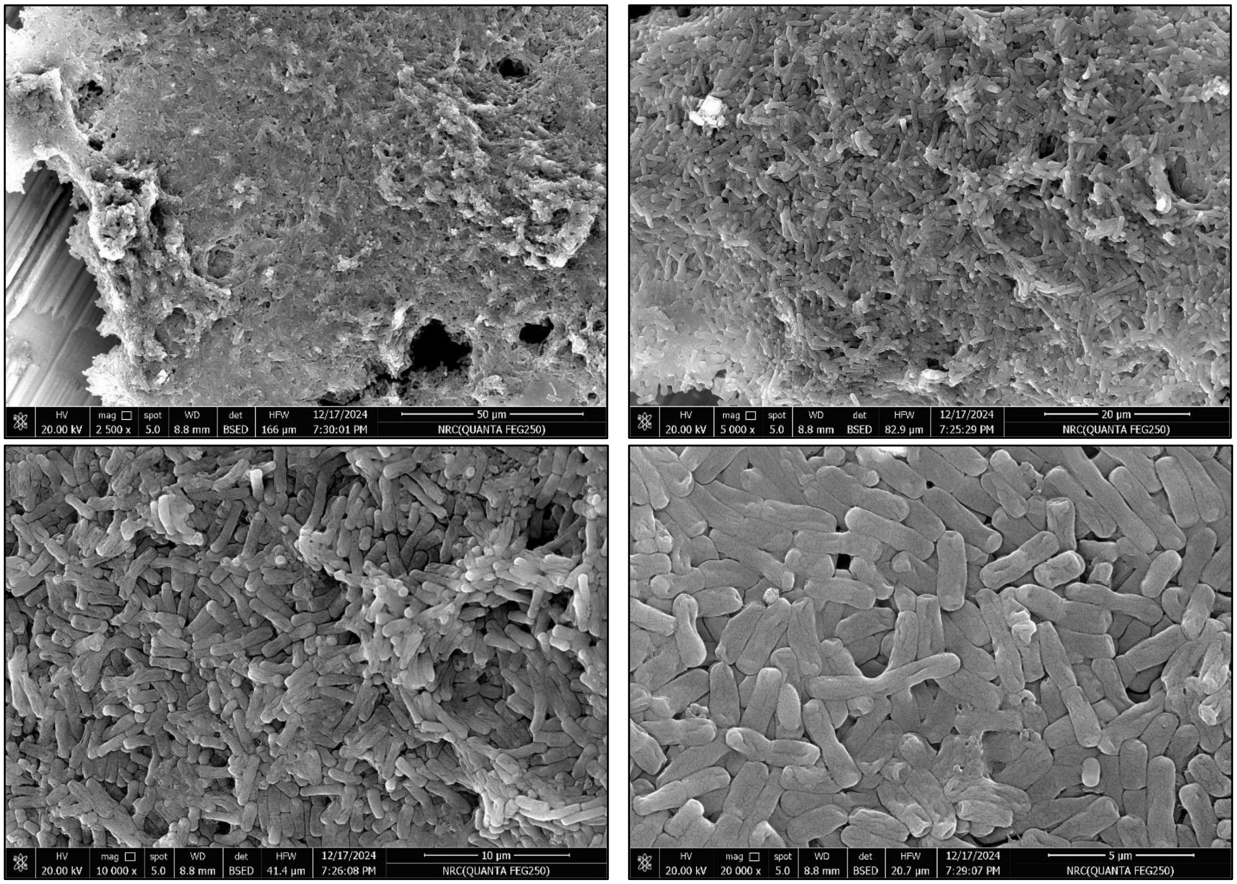

Figure S6. *P. aeruginosa*. after treatment with compound **3a** (8 µg/ml).

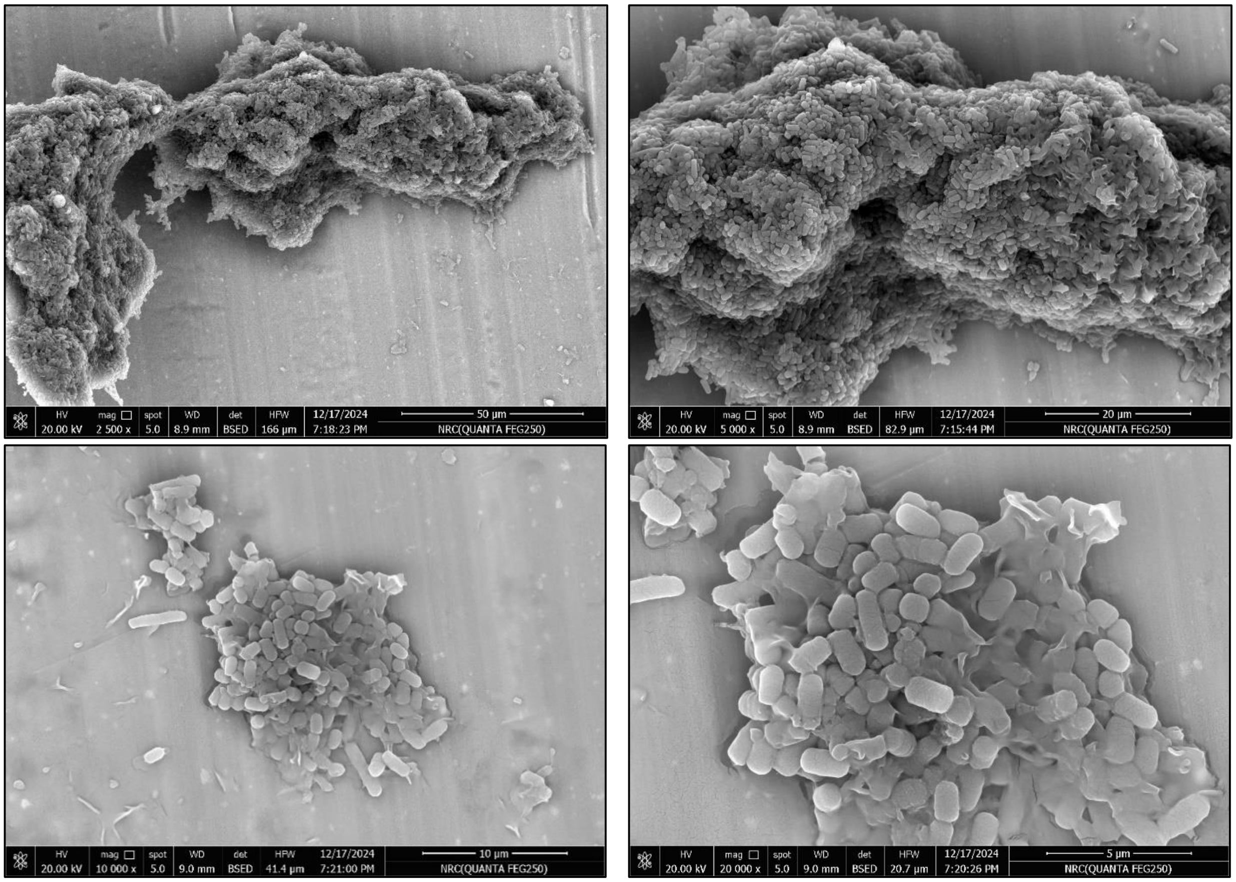

Figure S7. *P. aeruginosa*. after treatment with compound **3c** (8 μg/ml).

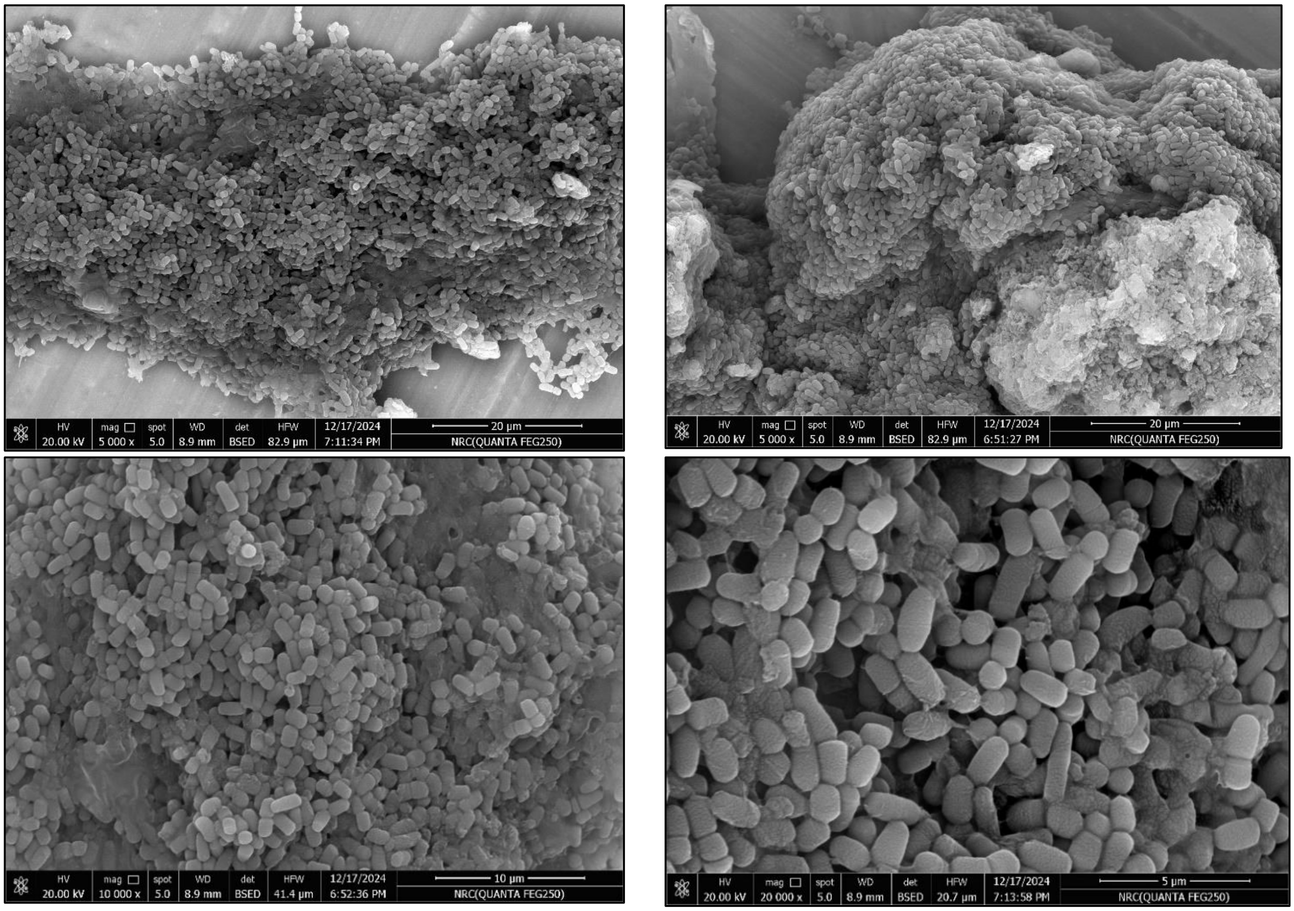

3. Supplementary Note 3: *Bioavailability prediction*

The intricate pharmacological behavior of molecules within biological systems is governed by a dynamic interplay of factors, including bioactivity, transport dynamics, protein interactions, and diverse reactivity profiles. To predict and elucidate these behaviors for 7*H*-benzo[*c*]carbazol-10-ol derivatives **3a** and **3c**, as well as the antibiotic amoxicillin, the SwissADME computational platform was employed with targeted modifications to integrate their distinct physicochemical properties [1]. Critical descriptors, such as the number and spatial configuration of heavy atoms, topological polar surface area (TPSA), molar refractivity, aqueous solubility (S), and lipophilicity indices, were rigorously evaluated to characterize their molecular landscapes [2]. Additionally, pharmacokinetic parameters, including skin permeability (Log Kp), blood-brain barrier (BBB) penetration potential, P-glycoprotein substrate affinity (P-gp substrate), and gastrointestinal (GI) absorption efficiency, were comprehensively assessed (**Table S3**).

Table S3. Predicted physicochemical variables of carbazole derivatives **3a** and **3c** and **Amoxicillin**.

physicochemical variables	3a	3c	Amoxicillin
Molecular weight (g/mol)	323.39	337.41	365.40
Heavy atoms	25	26	25
Aromatic heavy atoms	23	23	6
Rotatable bonds	1	1	5
Fraction Csp^3	0.04	0.08	0.44
H-bond acceptors	1	1	6
H-bond donors	1	1	4
Log S	−6.40	−6.69	−0.7
XLogP3	6.26	6.62	−1.99
Molar Refractivity	105.28	110.24	94.59
TPSA (Å^2)	25.16	25.16	158.26
Log Kp (skin permeation) (cm/s)	−3.83	−3.66	−9.94
GI absorption	High	High	Low
P-gp substrate	Yes	Yes	No
BBB permeant	No	No	No

The BOILED-Egg model was utilized to visualize and predict the compounds' brain and intestinal permeability profiles, offering a nuanced understanding of their pharmacokinetic behavior [3].The BOILED-Egg model classifies molecular behavior into two primary zones: the yellow region, indicative of a high probability of BBB penetration, and the white region, which signifies a strong likelihood of GI absorption. Importantly, these zones are

not mutually exclusive, allowing for overlapping predictions. Notably, the 7*H*-benzo[*c*]carbazol-10-ol derivatives **3a** and **3c** exhibited a low likelihood of BBB penetration but demonstrated a markedly higher probability of GI absorption, outperforming amoxicillin in this respect (**Figure S8**). This enhanced GI absorption potential underscores their promising bioavailability in gastrointestinal contexts. Furthermore, the model incorporates P-glycoprotein (P-gp) activity, which is critical for drug efflux mechanisms. Compounds predicted to be actively effluxed by P-gp (PGP+) are represented by blue dots, while red dots signify molecules that are not P-gp substrates. Derivatives **3a** and **3c** were predicted to be actively effluxed by P-gp, indicating their susceptibility to P-gp-mediated transport, whereas amoxicillin was classified as a non-substrate, suggesting distinct transport and bioavailability profiles (**Figure S8**).

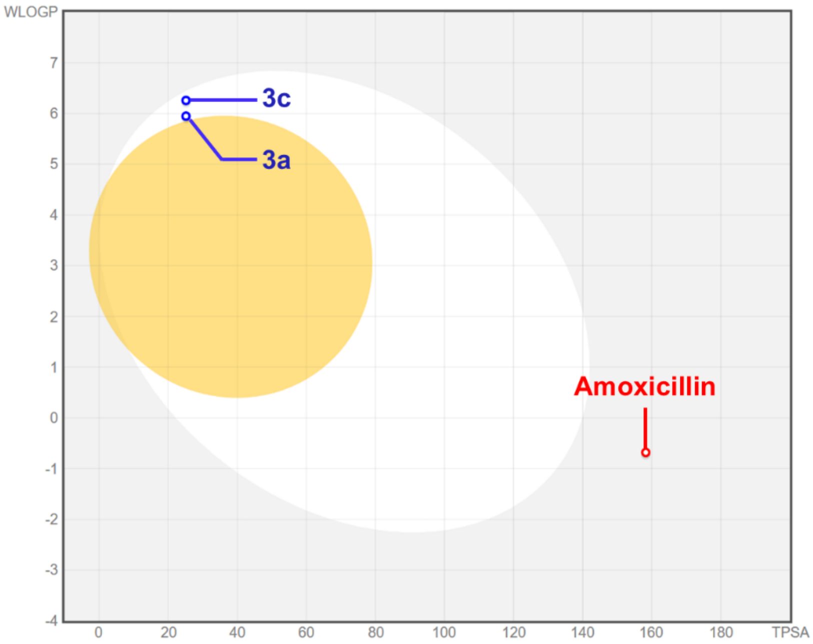

Figure S8. BOILED-Egg model of **3a**, **3c**, and amoxicillin.

3. Supplementary Note 4: *Molecular Docking*

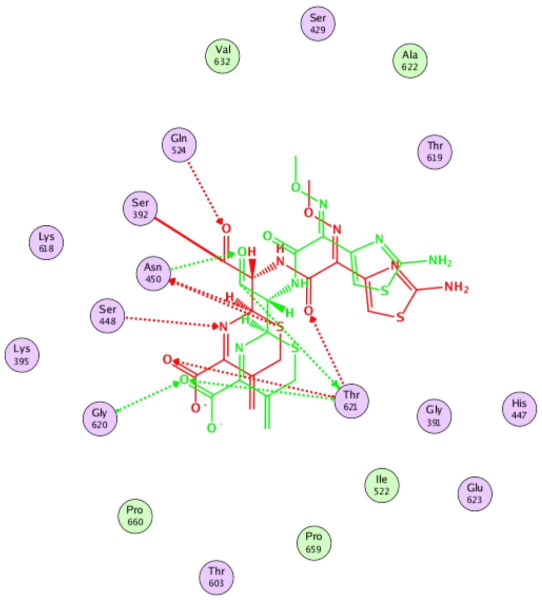

Figure S9. The co-crystallized ligand (red) overlayed to the docked pose (green) in *S. aureus* PBP3 active site (PDB ID: 3VSL) with RMSD of 1.74 Å.

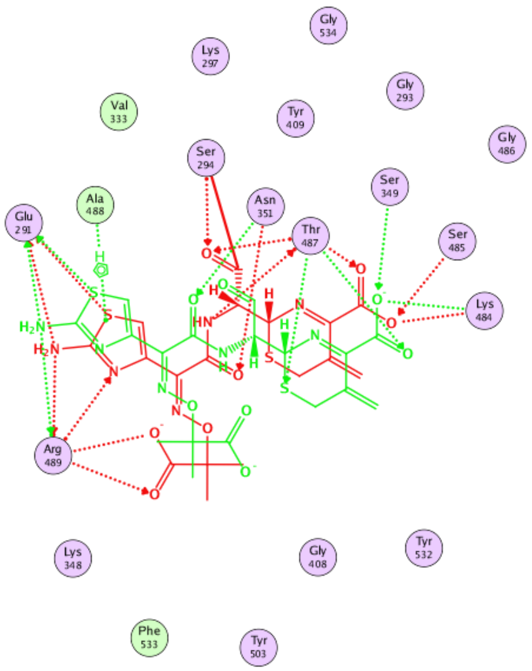

Figure S10. The co-crystallized ligand (red) overlayed to the docked pose (green) in the *P. aeruginosa* PBP3 active site (PDB ID: 3PBO) with RMSD of 1.69 Å.

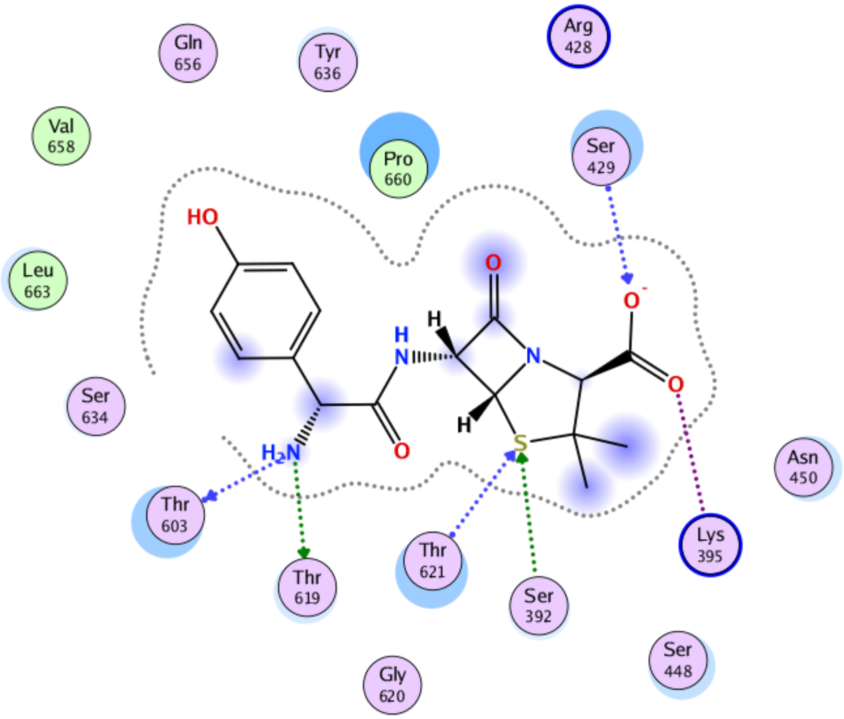

Figure S11. The best docking pose of Amoxicillin in *S. aureus* PBP3 active site (PDB ID: 3VSL).

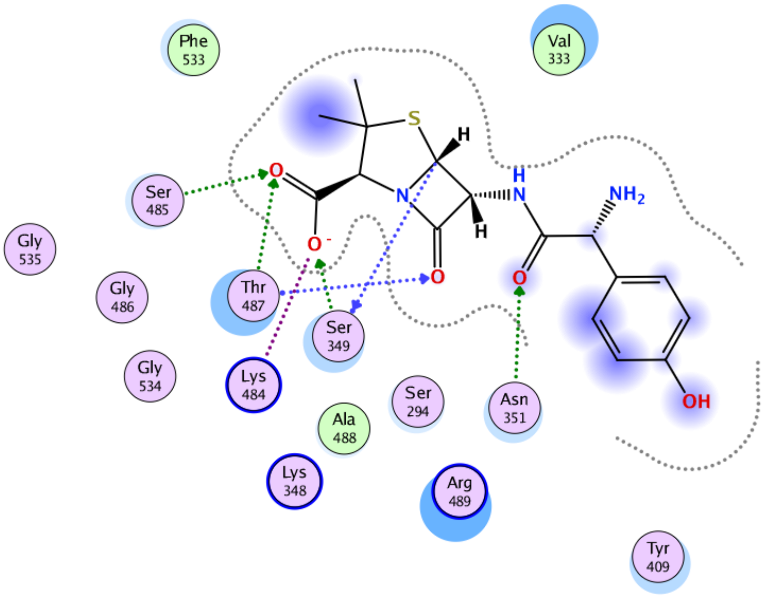

Figure S12. The best docking pose of Amoxicillin in *P. aeruginosa* PBP3 active site (PDB ID: 3PBO).

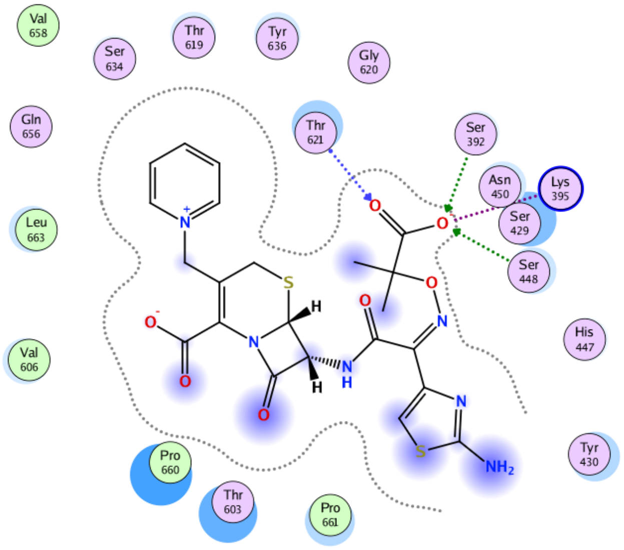

Figure S13. The best docking pose of Ceftazidime in *S. aureus* PBP3 active site (PDB ID: 3VSL).

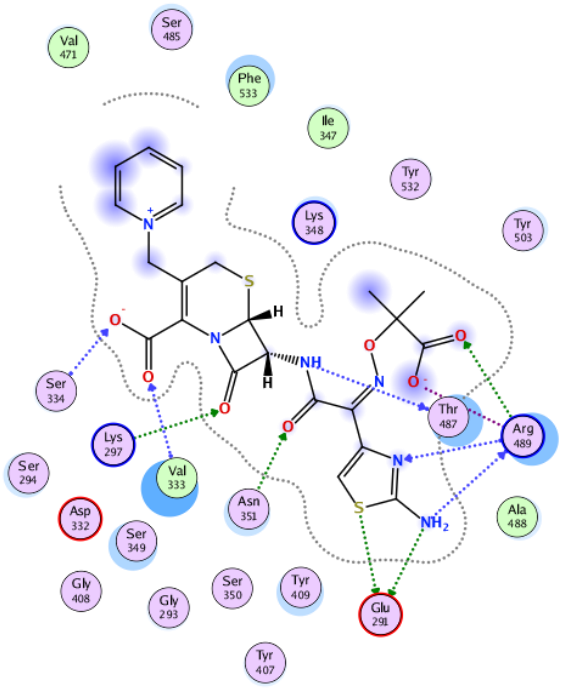

Figure S14. The best docking pose of Ceftazidime in *P. aeruginosa* PBP3 active site (PDB ID: 3PBO).

4. Supplementary Note 5: *Molecular orbitals of **3a***

Table S4. Selected molecular orbitals of **3a** optimized in the ground state S_0 calculated at B3LYP/6-311+G(d,p) level of theory (isosurface value = 0.02 a.u.).

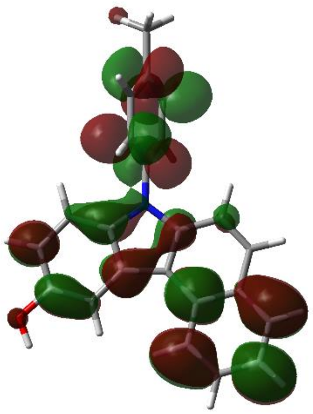	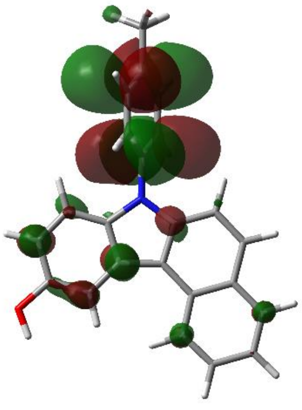	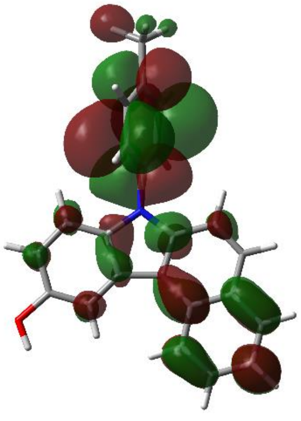
LUMO+3 (−0.49 eV)	LUMO+2 (−0.67 eV)	LUMO+1 (−0.71 eV)
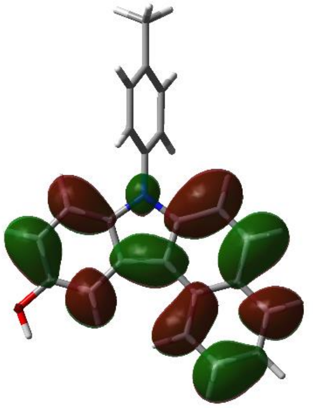	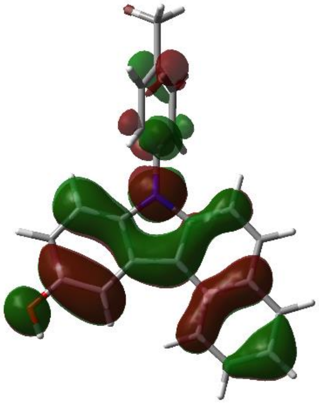	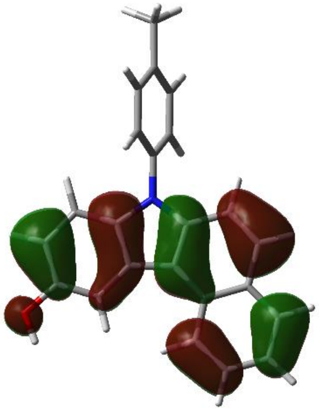
LUMO (−1.51 eV)	HOMO (−5.50 eV)	HOMO−1 (−5.65 eV)
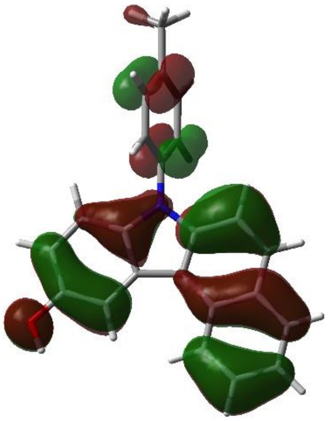	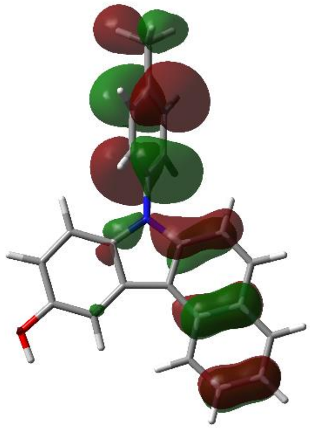	
HOMO−2 (−6.72 eV)	HOMO−3 (−6.93 eV)	

Table S5. Selected molecular orbitals of **3a** optimized in the ground state S_0 calculated at B3LYP/6-311G level of theory (isosurface value = 0.02 a.u.).

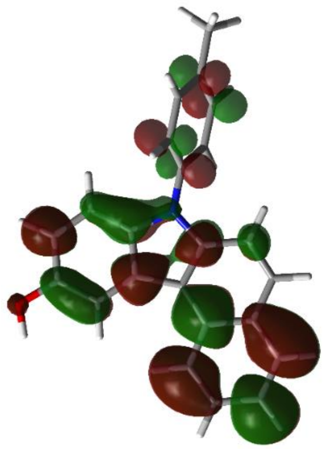<br>LUMO+3 (−0.25 eV)	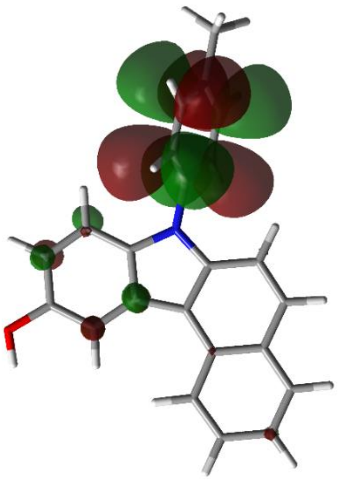<br>LUMO+2 (−0.63 eV)	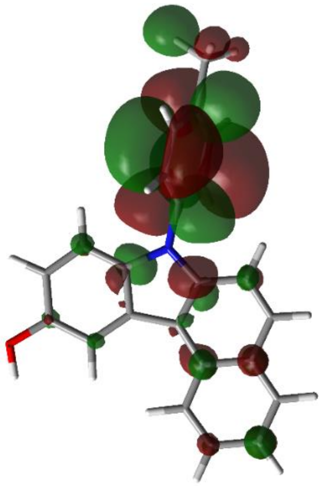<br>LUMO+1 (−0.67 eV)
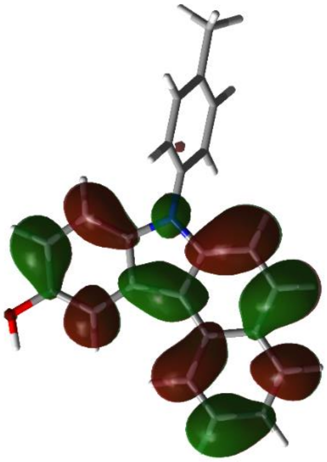<br>LUMO (−1.31 eV)	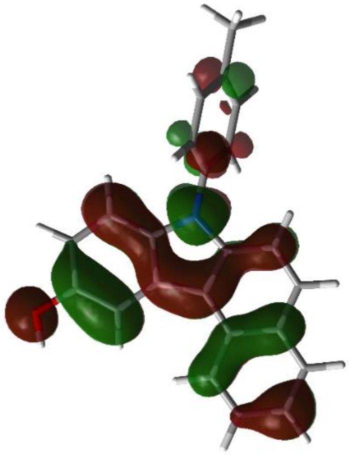<br>HOMO (−5.43 eV)	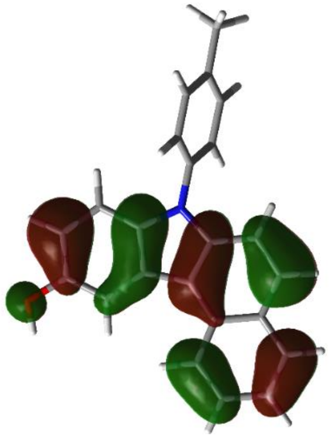<br>HOMO−1 (−5.57 eV)
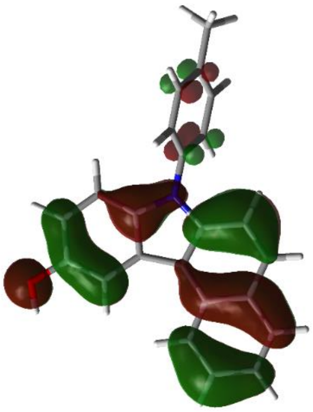<br>HOMO−2 (−6.66 eV)	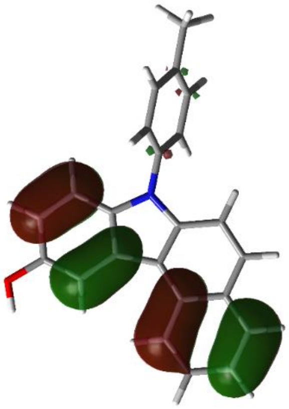<br>HOMO−3 (−7.00 eV)	

5. **Supplementary Note 6:** *Simulated UV-Vis spectra*

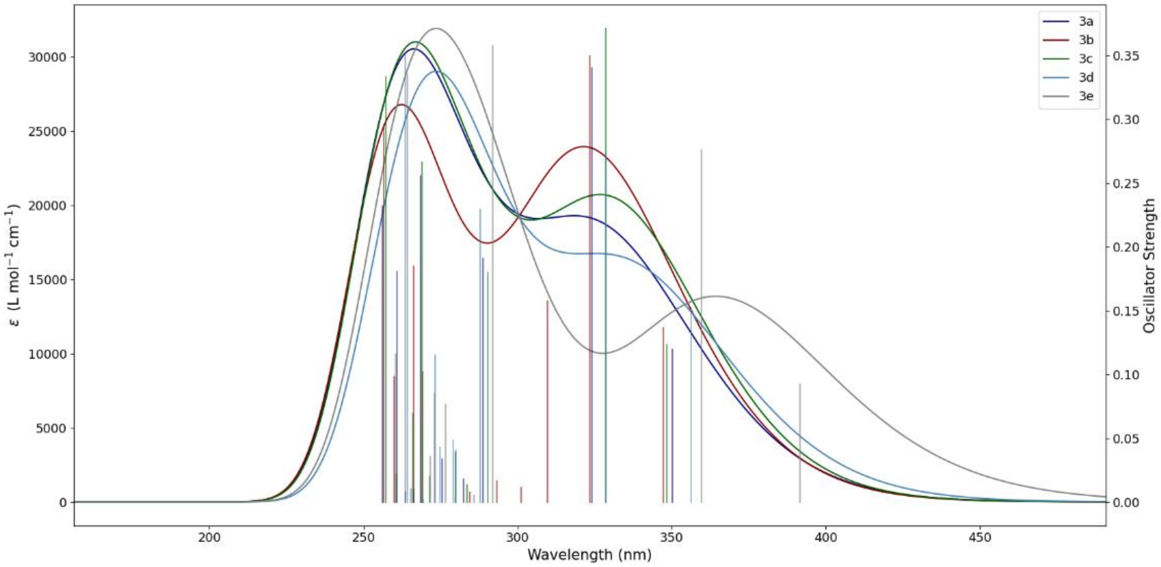

Figure S15a. Simulated UV-Vis absorption spectra of **3a**–**3e** at B3LYP/6-311G/PCM = chloroform level of theory.

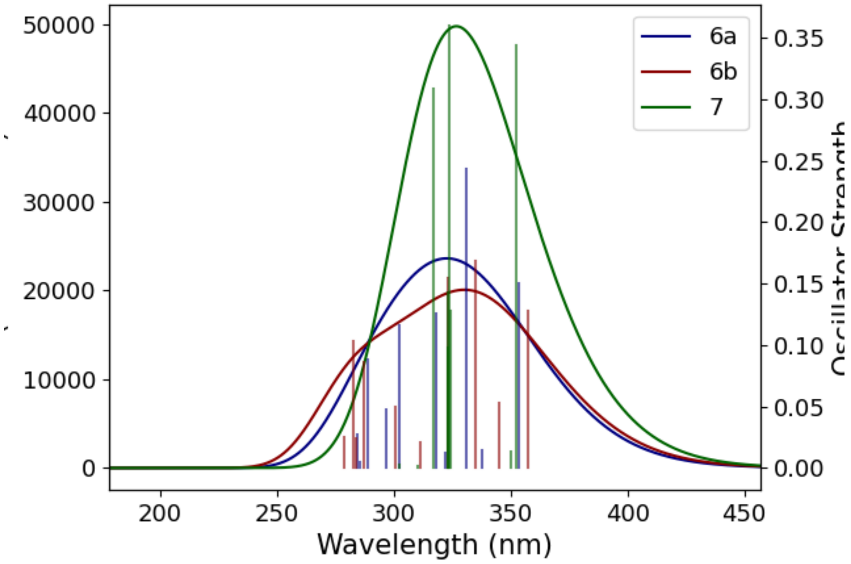

Figure S15b. Simulated UV-Vis absorption spectra of **6a**, **6b**, and **7** at B3LYP/6-311G/PCM = chloroform level of theory.

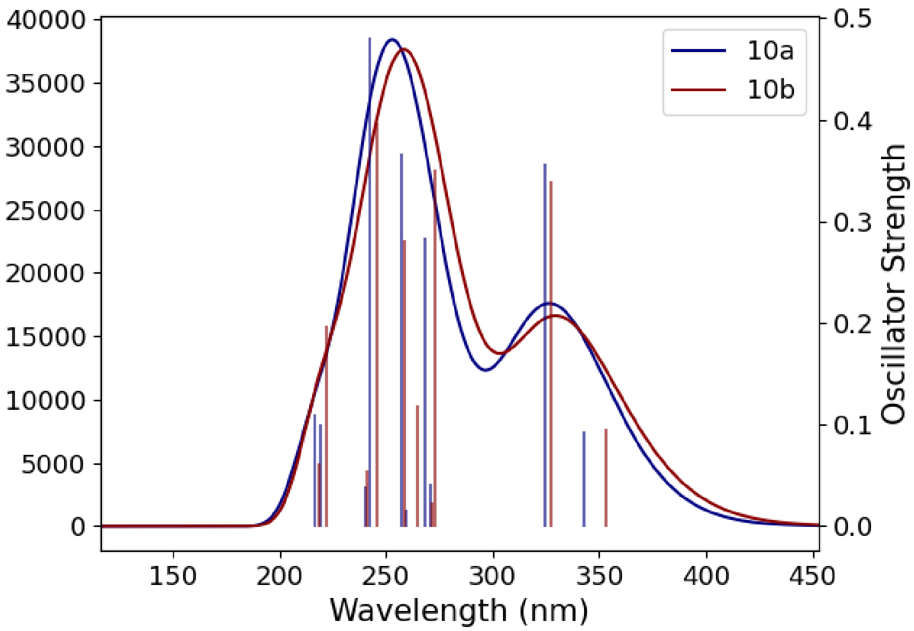

Figure S15c. Simulated UV-Vis absorption spectra of **10a** and **10b** at B3LYP/6-311G/PCM = chloroform level of theory.

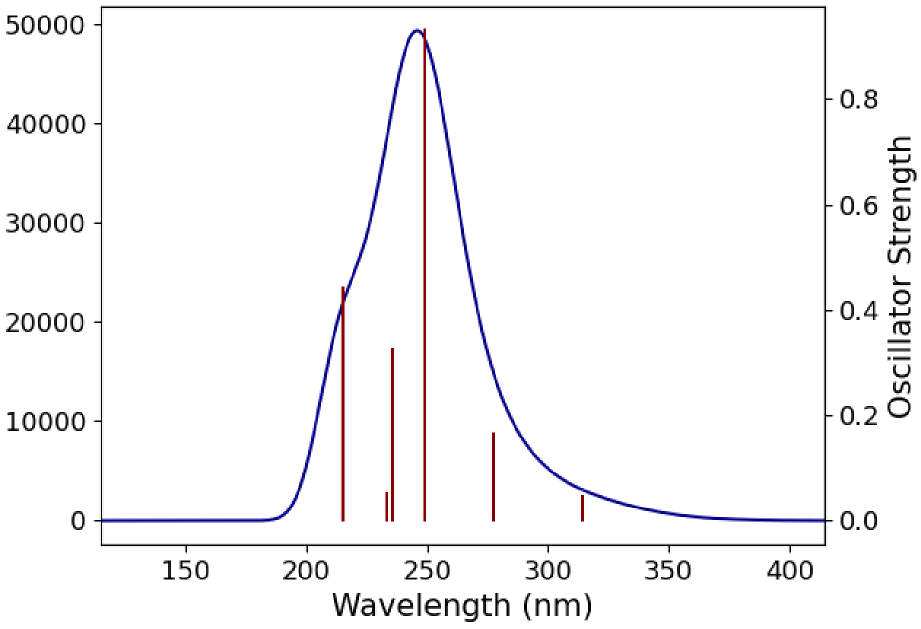

Figure S15d. Simulated UV-Vis absorption spectra of **12** at B3LYP/6-311G/PCM = chloroform level of theory.

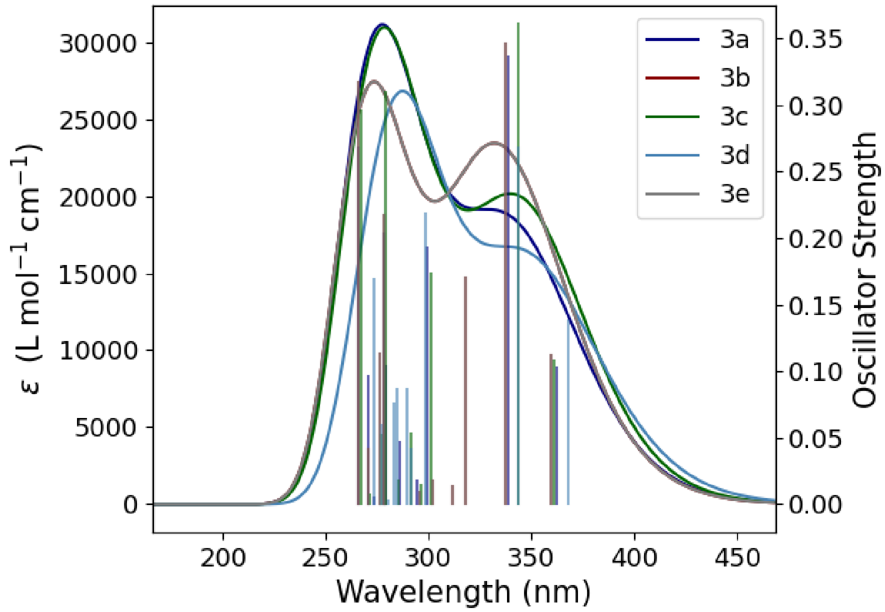

Figure S15e. Simulated UV-Vis absorption spectra of **3a**–**3e** at B3LYP/6-311+G(d,p)/PCM = chloroform level of theory.

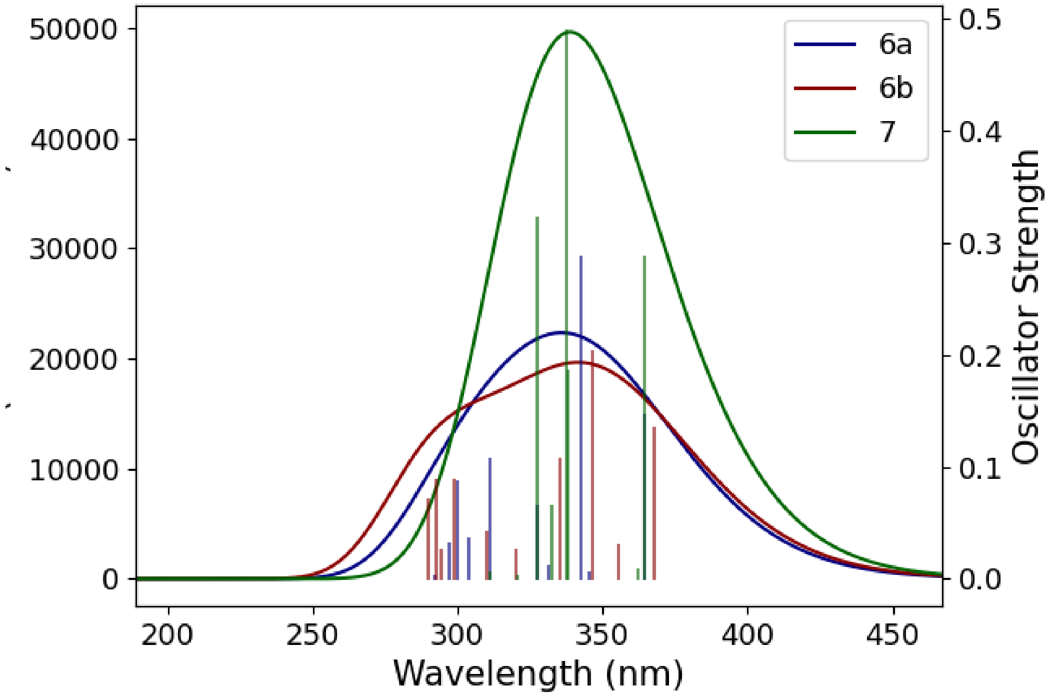

Figure S15f. Simulated UV-Vis absorption spectra of **6a**, **6b**, and **7** at B3LYP/6-311+G(d,p)/PCM = chloroform level of theory.

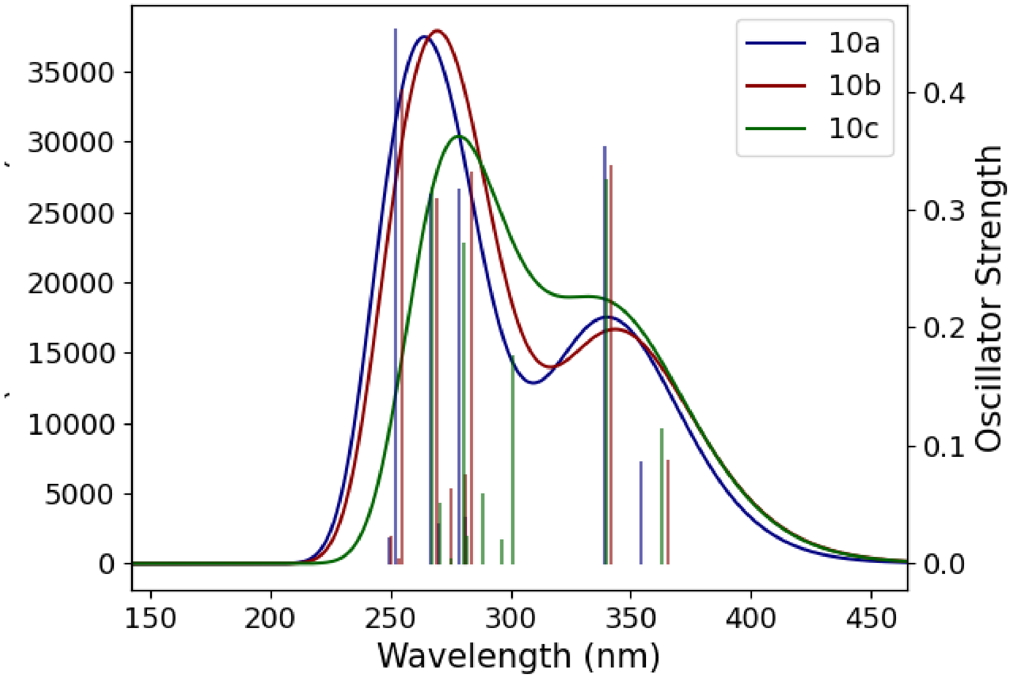

Figure S15g. Simulated UV-Vis absorption spectra of **10a** and **10b** at B3LYP/6-311+G(d,p)/PCM = chloroform level of theory.

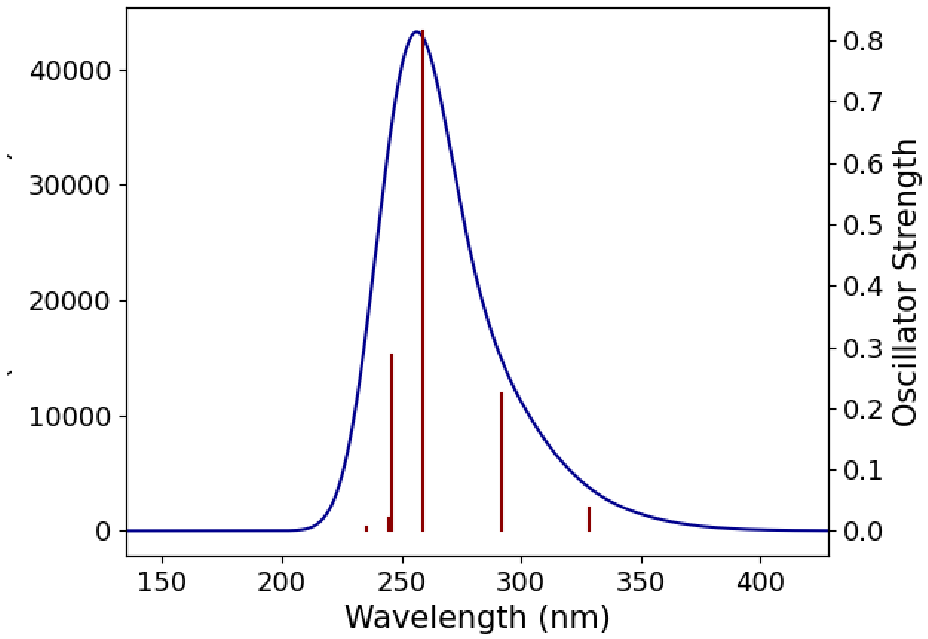

Figure S15h. Simulated UV-Vis absorption spectra of **12** at B3LYP/6-311+G(d,p)/PCM = chloroform level of theory.

6. Supplementary Note 7: *Summary of the TD-DFT calculation results*

Table S6. Summary of the TD-DFT calculation results of **3a** (S_0 state geometry) at B3LYP/6-311+G(d,p)/ PCM = chloroform level of theory.

Excited states	Energy (eV)	Wavelength (nm)	Oscillator strength	Major contributions		
S1	3.4181	362.73	0.1027	HOMO	LUMO	95
S2	3.6624	338.53	0.3362	HOMO-1	LUMO	93
				HOMO	LUMO+3	3
S3	4.1449	299.13	0.1924	HOMO	LUMO+1	90
				HOMO	LUMO+2	6
S4	4.2076	294.66	0.0177	HOMO	LUMO+1	5
				HOMO	LUMO+2	90
				HOMO	LUMO+3	2
S5	4.3332	286.12	0.0465	HOMO-3	LUMO	2
				HOMO-2	LUMO	12
				HOMO-1	LUMO+1	75
S6	4.4375	279.4	0.1038	HOMO-1	LUMO+2	78
				HOMO	LUMO+3	13
				HOMO	LUMO+4	4
S7	4.4527	278.44	0.2027	HOMO-2	LUMO	4
				HOMO-1	LUMO	3
				HOMO-1	LUMO+2	14
				HOMO	LUMO+3	72
S8	4.5334	273.49	0.0045	HOMO-3	LUMO	3
				HOMO-2	LUMO	15
				HOMO-1	LUMO+1	15
				HOMO-1	LUMO+3	46
				HOMO-1	LUMO+4	7
				HOMO	LUMO+3	4
				HOMO	LUMO+4	5
S9	4.5797	270.73	0.0956	HOMO-1	LUMO+2	4
				HOMO-1	LUMO+3	15
				HOMO-1	LUMO+4	5
				HOMO	LUMO+4	69

S10	4.6626	265.91	0.2679	HOMO-2	LUMO	20
				HOMO-1	LUMO+3	21
				HOMO-1	LUMO+4	39
				HOMO	LUMO+4	14

S_1~S_{10}

Table S7. Summary of the TD-DFT calculation results of **3a** (S_0 state geometry) at B3LYP/6-311G/PCM = chloroform level of theory.

Excited states	Energy (eV)	Wavelength (nm)	Oscillator strength	Major contributions		
S1	3.5391	350.33	0.1196	HOMO	LUMO	95
S2	3.8255	324.1	0.3404	HOMO−1	LUMO	93
				HOMO	LUMO+3	3
S3	4.2911	288.94	0.1907	HOMO	LUMO+1	95
				HOMO	LUMO+2	2
S4	4.3887	282.51	0.0176	HOMO	LUMO+2	93
				HOMO	LUMO+3	3
S5	4.4991	275.57	0.0333	HOMO−3	LUMO	3
				HOMO−2	LUMO	17
				HOMO−1	LUMO+1	69
				HOMO−1	LUMO+3	2
				HOMO	LUMO+4	2
S6	4.6152	268.64	0.2553	HOMO−2	LUMO	6
				HOMO−1	LUMO	3
				HOMO−1	LUMO+2	3
				HOMO−1	LUMO+3	2
				HOMO	LUMO+3	76
				HOMO	LUMO+4	3
S7	4.6539	266.41	0.0094	HOMO−1	LUMO+2	91
				HOMO	LUMO+3	4
S8	4.7038	263.58	0.0073	HOMO−3	LUMO	2
				HOMO−2	LUMO	16
				HOMO−1	LUMO+1	23
				HOMO−1	LUMO+3	32
				HOMO−1	LUMO+4	9
				HOMO	LUMO+3	5

				HOMO	LUMO+4	8
S9	4.7514	260.94	0.1802	HOMO−1	LUMO+2	4
				HOMO−1	LUMO+3	11
				HOMO	LUMO+3	4
				HOMO	LUMO+4	72
S10	4.8414	256.09	0.2319	HOMO−4	LUMO	5
				HOMO−2	LUMO	17
				HOMO−1	LUMO+3	33
				HOMO−1	LUMO+4	36
				HOMO	LUMO+4	5

S_1~S_{10}

Table S8. Summary of the TD-DFT calculation results of **3b** (S_0 state geometry) at B3LYP/6-311+G(d,p)/ PCM = chloroform level of theory.

Excited states	Energy (eV)	Wavelength (nm)	Oscillator strength	Major contributions		
S1	3.4474	359.65	0.1124	HOMO-1	LUMO+3	2
				HOMO	LUMO	95
S2	3.6717	337.67	0.3455	HOMO-1	LUMO	93
				HOMO	LUMO+3	4
S3	3.9017	317.77	0.1695	HOMO	LUMO+1	98
S4	3.9787	311.62	0.0129	HOMO	LUMO+2	97
S5	4.1033	302.15	0.0176	HOMO-2	LUMO	2
				HOMO-1	LUMO+1	95
S6	4.1942	295.61	0.0087	HOMO-1	LUMO+2	99
S7	4.4542	278.35	0.2168	HOMO-2	LUMO	27
				HOMO-1	LUMO+3	27
				HOMO-1	LUMO+4	4
				HOMO	LUMO+3	34
S8	4.4876	276.28	0.1135	HOMO-2	LUMO	7
				HOMO-1	LUMO	2
				HOMO-1	LUMO+3	19
				HOMO	LUMO+3	55
				HOMO	LUMO+4	8

S9	4.5853	270.4	0.0408	HOMO-1	LUMO+3	22
				HOMO-1	LUMO+4	11
				HOMO	LUMO+4	55
				HOMO	LUMO+5	3
S10	4.6575	266.2	0.3168	HOMO-2	LUMO	16
				HOMO-1	LUMO+3	13
				HOMO-1	LUMO+4	32
				HOMO-1	LUMO+5	3
				HOMO	LUMO+3	3
				HOMO	LUMO+4	28

S_1~S_{10}

Table S9. Summary of the TD-DFT calculation results of **3b** (S_0 state geometry) at B3LYP/6-311G/PCM = chloroform level of theory.

Excited states	Energy (eV)	Wavelength (nm)	Oscillator strength	Major contributions		
S1	3.5688	347.42	0.1365	HOMO−1	LUMO+3	3
				HOMO	LUMO	95
S2	3.8336	323.42	0.3498	HOMO−1	LUMO	93
				HOMO	LUMO+3	4
S3	4.0015	309.84	0.1574	HOMO	LUMO+1	99
S4	4.117	301.15	0.011	HOMO	LUMO+2	98
S5	4.2278	293.26	0.0161	HOMO−2	LUMO	2
				HOMO−1	LUMO+1	95
S6	4.3585	284.46	0.0072	HOMO−1	LUMO+2	99
S7	4.606	269.18	0.1023	HOMO−3	LUMO	2
				HOMO−2	LUMO	40
				HOMO−1	LUMO+3	27
				HOMO−1	LUMO+4	6
				HOMO	LUMO	2
				HOMO	LUMO+3	17
S8	4.6551	266.34	0.1847	HOMO−2	LUMO	3
				HOMO−1	LUMO	3
				HOMO−1	LUMO+3	8
				HOMO	LUMO+3	70

				HOMO	LUMO+4	9
S9	4.7669	260.09	0.0984	HOMO−1	LUMO+3	15
				HOMO−1	LUMO+4	6
				HOMO	LUMO+3	4
				HOMO	LUMO+4	69
S10	4.8328	256.54	0.2958	HOMO−3	LUMO	5
				HOMO−2	LUMO	14
				HOMO−1	LUMO+3	23
				HOMO−1	LUMO+4	36
				HOMO	LUMO+3	3
				HOMO	LUMO+4	15

S_1~S_{10}

Table S10. Summary of the TD-DFT calculation results of **3c** (S_0 state geometry) at B3LYP/6-311+G(d,p)/ PCM = chloroform level of theory.

Excited states	Energy (eV)	Wavelength (nm)	Oscillator strength	Major contributions		
S1	3.4354	360.9	0.1079	HOMO-1	LUMO+3	2
				HOMO	LUMO	95
S2	3.609	343.54	0.3605	HOMO-1	LUMO	94
				HOMO	LUMO+3	3
S3	4.1168	301.17	0.1734	HOMO	LUMO+1	95
				HOMO	LUMO+2	2
S4	4.1836	296.36	0.0139	HOMO	LUMO+1	2
				HOMO	LUMO+2	94
S5	4.2538	291.46	0.0525	HOMO-2	LUMO	6
				HOMO-1	LUMO+1	87
S6	4.3492	285.07	0.0172	HOMO-1	LUMO+2	97
S7	4.4462	278.85	0.3093	HOMO-2	LUMO	11
				HOMO-1	LUMO+3	20
				HOMO-1	LUMO+4	3
				HOMO	LUMO+3	57
S8	4.4801	276.74	0.0522	HOMO-2	LUMO	5
				HOMO-1	LUMO+1	4

				HOMO-1	LUMO+3	49
				HOMO	LUMO+3	33
S9	4.568	271.42	0.0071	HOMO-2	LUMO	2
				HOMO-1	LUMO+3	4
				HOMO-1	LUMO+4	19
				HOMO	LUMO+4	66
S10	4.6338	267.56	0.2955	HOMO-2	LUMO	12
				HOMO-1	LUMO+3	7
				HOMO-1	LUMO+4	47
				HOMO	LUMO+3	3
				HOMO	LUMO+4	26

S_1~S_{10}

Table S11. Summary of the TD-DFT calculation results of **3c** (S_0 state geometry) at B3LYP/6-311G/PCM = chloroform level of theory.

Excited states	Energy (eV)	Wavelength (nm)	Oscillator strength	Major contributions		
S1	3.5586	348.41	0.1234	HOMO−1	LUMO+3	3
				HOMO	LUMO	95
S2	3.7728	328.63	0.3713	HOMO−1	LUMO	94
				HOMO	LUMO+3	3
S3	4.2709	290.3	0.1796	HOMO	LUMO+1	96
S4	4.3714	283.62	0.0134	HOMO	LUMO+2	95
S5	4.4302	279.86	0.0388	HOMO−3	LUMO	3
				HOMO−2	LUMO	7
				HOMO−1	LUMO+1	84
S6	4.5652	271.59	0.0199	HOMO−1	LUMO+2	98
S7	4.609	269.01	0.2666	HOMO−2	LUMO	13
				HOMO−1	LUMO+3	15
				HOMO−1	LUMO+4	4
				HOMO	LUMO+3	59
S8	4.6585	266.15	0.0695	HOMO−3	LUMO	4
				HOMO−2	LUMO	8
				HOMO−1	LUMO+1	7
				HOMO−1	LUMO+3	45

				HOMO	LUMO+3	29
S9	4.7539	260.8	0.0214	HOMO−1	LUMO+3	4
				HOMO−1	LUMO+4	12
				HOMO	LUMO+4	74
S10	4.8166	257.41	0.3331	HOMO−4	LUMO	2
				HOMO−2	LUMO	16
				HOMO−1	LUMO+3	12
				HOMO−1	LUMO+4	44
				HOMO	LUMO+3	4
				HOMO	LUMO+4	17

S_1~S_{10}

Table S12. Summary of the TD-DFT calculation results of **3d** (S_0 state geometry) at B3LYP/6-311+G(d,p)/ PCM = chloroform level of theory.

Excited states	Energy (eV)	Wavelength (nm)	Oscillator strength	Major contributions		
S1	3.3676	368.17	0.1376	HOMO-1	LUMO+1	2
				HOMO	LUMO	95
S2	3.6104	343.41	0.2674	HOMO-1	LUMO	92
				HOMO	LUMO+1	4
S3	4.1557	298.35	0.2179	HOMO-1	LUMO	2
				HOMO	LUMO+1	82
				HOMO	LUMO+2	8
				HOMO	LUMO+4	6
S4	4.2505	291.69	0.0417	HOMO-1	LUMO+1	5
				HOMO	LUMO+1	5
				HOMO	LUMO+2	76
				HOMO	LUMO+3	6
S5	4.2784	289.79	0.0858	HOMO-2	LUMO	24
				HOMO-1	LUMO+1	57
				HOMO	LUMO+2	5
				HOMO	LUMO+4	3
S6	4.3573	284.55	0.0865	HOMO-1	LUMO+3	15
				HOMO	LUMO+1	3
				HOMO	LUMO+2	6
				HOMO	LUMO+3	52

				HOMO	LUMO+4	17
S7	4.3752	283.38	0.0752	HOMO-1	LUMO+2	4
				HOMO-1	LUMO+3	17
				HOMO	LUMO+1	4
				HOMO	LUMO+3	8
				HOMO	LUMO+4	61
S8	4.4205	280.48	0.0027	HOMO-2	LUMO+3	4
				HOMO-1	LUMO+2	12
				HOMO-1	LUMO+3	43
				HOMO	LUMO+2	2
				HOMO	LUMO+3	29
				HOMO	LUMO+4	6
S9	4.4744	277.09	0.0591	HOMO-2	LUMO	6
				HOMO-1	LUMO+1	8
				HOMO-1	LUMO+2	49
				HOMO-1	LUMO+3	9
				HOMO-1	LUMO+5	8
				HOMO	LUMO+5	15
S10	4.5317	273.59	0.1692	HOMO-2	LUMO	20
				HOMO-1	LUMO+1	16
				HOMO-1	LUMO+4	32
				HOMO-1	LUMO+5	6
				HOMO	LUMO+5	21

S_1~S_{10}

Table S13. Summary of the TD-DFT calculation results of **3d** (S_0 state geometry) at B3LYP/6-311G/PCM = chloroform level of theory.

Excited states	Energy (eV)	Wavelength (nm)	Oscillator strength	Major contributions		
S1	3.4799	356.28	0.1522	HOMO−1	LUMO+1	2
				HOMO	LUMO	95
S2	3.7715	328.74	0.2734	HOMO−1	LUMO	92
				HOMO	LUMO+1	4
S3	4.3065	287.9	0.2292	HOMO	LUMO+1	81
				HOMO	LUMO+2	10
				HOMO	LUMO+4	5

S4	4.4258	280.14	0.0407	HOMO−2	LUMO	14
				HOMO−1	LUMO+1	25
				HOMO	LUMO+1	4
				HOMO	LUMO+2	43
				HOMO	LUMO+3	4
S5	4.4403	279.23	0.0481	HOMO−2	LUMO	20
				HOMO−1	LUMO+1	28
				HOMO	LUMO+2	32
				HOMO	LUMO+3	6
				HOMO	LUMO+4	4
S6	4.5098	274.92	0.0427	HOMO−1	LUMO+2	3
				HOMO−1	LUMO+3	16
				HOMO	LUMO+2	8
				HOMO	LUMO+3	65
				HOMO	LUMO+4	3
S7	4.5375	273.24	0.115	HOMO−1	LUMO+3	6
				HOMO	LUMO+1	5
				HOMO	LUMO+4	77
S8	4.5992	269.58	0.0015	HOMO−2	LUMO+3	3
				HOMO−1	LUMO+2	15
				HOMO−1	LUMO+3	49
				HOMO	LUMO+2	2
				HOMO	LUMO+3	20
				HOMO	LUMO+4	4
S9	4.6698	265.5	0.0101	HOMO−1	LUMO+1	2
				HOMO−1	LUMO+2	31
				HOMO−1	LUMO+3	7
				HOMO−1	LUMO+4	2
				HOMO−1	LUMO+5	5
				HOMO	LUMO+5	47
S10	4.7043	263.56	0.3501	HOMO−2	LUMO	27
				HOMO−1	LUMO+1	29
				HOMO−1	LUMO+4	16
				HOMO−1	LUMO+5	12
				HOMO	LUMO+4	2
				HOMO	LUMO+5	9

S_1~S_{10}

Table S14. Summary of the TD-DFT calculation results of **3e** (S_0 state geometry) at B3LYP/6-311+G(d,p)/ PCM = chloroform level of theory.

Excited states	Energy (eV)	Wavelength (nm)	Oscillator strength	Major contributions		
S1	3.0426	407.5	0.0835	HOMO	LUMO	97
S2	3.2817	377.8	0.2719	HOMO-1	LUMO	95
S3	4.1122	301.51	0.3301	HOMO	LUMO+1	88
				HOMO	LUMO+3	5
S4	4.1856	296.21	0.014	HOMO-5	LUMO	3
				HOMO-3	LUMO	12
				HOMO-2	LUMO	26
				HOMO-1	LUMO+1	41
				HOMO	LUMO+2	14
S5	4.2561	291.31	0.0043	HOMO-3	LUMO	3
				HOMO-2	LUMO	3
				HOMO-1	LUMO+1	7
				HOMO	LUMO+2	78
				HOMO	LUMO+4	2
S6	4.3289	286.41	0.0459	HOMO-1	LUMO+2	5
				HOMO	LUMO+1	3
				HOMO	LUMO+2	2
				HOMO	LUMO+3	83
S7	4.3711	283.64	0.0834	HOMO-3	LUMO	22
				HOMO-2	LUMO	48
				HOMO-1	LUMO+1	8
				HOMO	LUMO+3	2
				HOMO	LUMO+4	16
S8	4.3975	281.94	0.0218	HOMO-3	LUMO	5
				HOMO-2	LUMO	12
				HOMO	LUMO+4	75
S9	4.4987	275.6	0.4142	HOMO-5	LUMO	5
				HOMO-1	LUMO+2	75
				HOMO	LUMO+1	4
				HOMO	LUMO+3	5

				HOMO	LUMO+4	2
S10	4.5872	270.28	0.1235	HOMO-5	LUMO	7
				HOMO-4	LUMO	5
				HOMO-3	LUMO	14
				HOMO-1	LUMO+1	16
				HOMO-1	LUMO+3	47
				HOMO-1	LUMO+4	6

S_1~S_{10}

Table S15. Summary of the TD-DFT calculation results of **3e** (S_0 state geometry) at B3LYP/6-311G/PCM = chloroform level of theory.

Excited states	Energy (eV)	Wavelength (nm)	Oscillator strength	Major contributions		
S1	3.1647	391.77	0.0922	HOMO	LUMO	97
S2	3.4454	359.85	0.2764	HOMO−1	LUMO	95
				HOMO	LUMO+1	2
S3	4.2469	291.94	0.3579	HOMO	LUMO+1	88
				HOMO	LUMO+3	6
S4	4.3365	285.91	0.005	HOMO−5	LUMO	3
				HOMO−3	LUMO	8
				HOMO−2	LUMO	33
				HOMO−1	LUMO+1	40
				HOMO	LUMO+2	10
S5	4.4293	279.92	0.0025	HOMO−3	LUMO	2
				HOMO−2	LUMO	2
				HOMO−1	LUMO+1	5
				HOMO	LUMO+2	78
				HOMO	LUMO+3	5
S6	4.4804	276.73	0.0764	HOMO−1	LUMO+2	3
				HOMO	LUMO+1	4
				HOMO	LUMO+2	6
				HOMO	LUMO+3	82
S7	4.5418	272.99	0.0851	HOMO−3	LUMO	26
				HOMO−2	LUMO	35
				HOMO−1	LUMO+1	9
				HOMO	LUMO+4	25

S8	4.5629	271.72	0.0357	HOMO−3	LUMO	10
				HOMO−2	LUMO	15
				HOMO	LUMO+4	66
S9	4.6893	264.4	0.3356	HOMO−5	LUMO	13
				HOMO−4	LUMO	4
				HOMO−1	LUMO+2	67
				HOMO−1	LUMO+4	2
				HOMO	LUMO+1	3
				HOMO	LUMO+3	3
S10	4.7621	260.36	0.1155	HOMO−5	LUMO	5
				HOMO−4	LUMO	3
				HOMO−3	LUMO	11
				HOMO−1	LUMO+1	14
				HOMO−1	LUMO+3	56
				HOMO−1	LUMO+4	4

S_1~S_{10}

Table S16. Summary of the TD-DFT calculation results of **6a** (S_0 state geometry) at B3LYP/6-311+G(d,p)/ PCM = chloroform level of theory.

Excited states	Energy (eV)	Wavelength (nm)	Oscillator strength	Major contributions		
S1	3.4008	364.58	0.1457	HOMO	LUMO	96
S2	3.5887	345.48	0.0055	HOMO	LUMO+1	98
S3	3.6218	342.33	0.2874	HOMO-1	LUMO	93
S4	3.7411	331.41	0.0107	HOMO-2	LUMO	91
				HOMO-1	LUMO+1	4
S5	3.7837	327.68	0.0646	HOMO-2	LUMO	3
				HOMO-1	LUMO+1	93
S6	3.9832	311.27	0.1073	HOMO-3	LUMO	2
				HOMO-2	LUMO+1	89
S7	4.0784	304	0.0352	HOMO-3	LUMO	38
				HOMO-3	LUMO+1	27
				HOMO-2	LUMO+1	3
				HOMO-2	LUMO+2	2
				HOMO-2	LUMO+4	10

				HOMO-2	LUMO+5	5
				HOMO-2	LUMO+6	3
				HOMO	LUMO+2	6
				HOMO	LUMO+4	3
S8	4.1322	300.05	0.087	HOMO	LUMO+3	97
S9	4.1702	297.31	0.0309	HOMO-3	LUMO	4
				HOMO	LUMO+2	86
				HOMO	LUMO+4	2
S10	4.2468	291.95	0.0022	HOMO-3	LUMO	50
				HOMO-3	LUMO+1	40
				HOMO-2	LUMO+4	2
				HOMO	LUMO+4	2

S_1~S_{10}

Table S17. Summary of the TD-DFT calculation results of **6a** (S_0 state geometry) at B3LYP/6-311G/PCM = chloroform level of theory.

Excited states	Energy (eV)	Wavelength (nm)	Oscillator strength	Major contributions		
S1	3.5082	353.41	0.1502	HOMO	LUMO	96
S2	3.6703	337.81	0.0143	HOMO	LUMO+1	97
S3	3.7468	330.91	0.2426	HOMO−1	LUMO	91
				HOMO−1	LUMO+1	3
S4	3.8488	322.14	0.0127	HOMO−2	LUMO	87
				HOMO−2	LUMO+1	4
				HOMO−1	LUMO+1	6
S5	3.8987	318.01	0.1254	HOMO−2	LUMO	4
				HOMO−1	LUMO	3
				HOMO−1	LUMO+1	88
S6	4.0985	302.51	0.1159	HOMO−3	LUMO	10
				HOMO−3	LUMO+1	3
				HOMO−2	LUMO	4
				HOMO−2	LUMO+1	74
S7	4.1788	296.7	0.0473	HOMO−3	LUMO	39
				HOMO−3	LUMO+1	20
				HOMO−2	LUMO+1	16

				HOMO−2	LUMO+3	2
				HOMO−2	LUMO+4	9
				HOMO−2	LUMO+5	3
				HOMO−2	LUMO+6	3
				HOMO	LUMO+3	3
				HOMO	LUMO+4	2
S8	4.2897	289.03	0.0879	HOMO	LUMO+2	79
				HOMO	LUMO+3	18
S9	4.3402	285.66	0.0046	HOMO−3	LUMO	41
				HOMO−3	LUMO+1	38
				HOMO	LUMO+2	3
				HOMO	LUMO+3	12
S10	4.3627	284.19	0.0274	HOMO−3	LUMO	3
				HOMO−3	LUMO+1	11
				HOMO	LUMO+2	12
				HOMO	LUMO+3	62
				HOMO	LUMO+4	3

S_1~S_{10}

Table S18. Summary of the TD-DFT calculation results of **6b** (S_0 state geometry) at B3LYP/6-311+G(d,p)/ PCM = chloroform level of theory.

Excited states	Energy (eV)	Wavelength (nm)	Oscillator strength	Major contributions		
S1	3.3701	367.89	0.1338	HOMO	LUMO	94
S2	3.4866	355.6	0.0293	HOMO	LUMO+1	96
S3	3.5809	346.23	0.2026	HOMO-1	LUMO	92
S4	3.6962	335.44	0.107	HOMO-1	LUMO+1	94
S5	3.8758	319.89	0.0249	HOMO-2	LUMO	90
				HOMO-2	LUMO+1	2
				HOMO	LUMO+2	3
S6	3.9953	310.33	0.0408	HOMO-3	LUMO	2
				HOMO-3	LUMO+1	3
				HOMO-2	LUMO+1	77
				HOMO	LUMO+2	8

S7	4.1459	299.05	0.0876	HOMO-2	LUMO	3
				HOMO-2	LUMO+1	7
				HOMO	LUMO+2	81
S8	4.2116	294.39	0.0259	HOMO-3	LUMO	7
				HOMO-3	LUMO+1	4
				HOMO-2	LUMO+1	4
				HOMO-2	LUMO+2	2
				HOMO	LUMO+3	70
				HOMO	LUMO+4	7
S9	4.2375	292.59	0.0877	HOMO-3	LUMO	25
				HOMO-3	LUMO+1	11
				HOMO-2	LUMO+1	5
				HOMO-2	LUMO+2	8
				HOMO-2	LUMO+3	5
				HOMO-1	LUMO+2	4
				HOMO	LUMO+3	3
				HOMO	LUMO+4	29
S10	4.2797	289.7	0.0697	HOMO-4	LUMO	13
				HOMO-3	LUMO+1	2
				HOMO-1	LUMO+2	64
				HOMO-1	LUMO+3	4

S_1~S_{10}

Table S19. Summary of the TD-DFT calculation results of **6b** (S_0 state geometry) at B3LYP/6-311G/PCM = chloroform level of theory.

Excited states	Energy (eV)	Wavelength (nm)	Oscillator strength	Major contributions		
S1	3.4681	357.5	0.1275	HOMO	LUMO	94
S2	3.5929	345.08	0.0524	HOMO	LUMO+1	95
S3	3.7024	334.87	0.1688	HOMO−1	LUMO	91
				HOMO−1	LUMO+1	4
S4	3.8352	323.28	0.1539	HOMO−1	LUMO	3
				HOMO−1	LUMO+1	90
S5	3.9838	311.22	0.0211	HOMO−2	LUMO	87
				HOMO−2	LUMO+1	6

S6	4.1222	300.77	0.0496	HOMO−3	LUMO	4
				HOMO−3	LUMO+1	5
				HOMO−2	LUMO+1	71
				HOMO−2	LUMO+2	3
				HOMO	LUMO+2	7
S7	4.3144	287.37	0.0863	HOMO−2	LUMO	3
				HOMO−2	LUMO+1	5
				HOMO	LUMO+2	82
				HOMO	LUMO+5	2
S8	4.3653	284.02	0.024	HOMO−3	LUMO	26
				HOMO−3	LUMO+1	9
				HOMO−2	LUMO	3
				HOMO−2	LUMO+1	10
				HOMO−2	LUMO+2	7
				HOMO	LUMO+3	39
S9	4.385	282.75	0.1032	HOMO−3	LUMO	15
				HOMO−3	LUMO+1	4
				HOMO−2	LUMO+1	2
				HOMO−2	LUMO+2	4
				HOMO−2	LUMO+3	3
				HOMO	LUMO+2	3
				HOMO	LUMO+3	36
				HOMO	LUMO+4	25
S10	4.4447	278.95	0.0255	HOMO−4	LUMO	36
				HOMO−4	LUMO+1	4
				HOMO−1	LUMO+2	31
				HOMO−1	LUMO+3	9
				HOMO−1	LUMO+5	4

S_1~S_{10}

Table S20. Summary of the TD-DFT calculation results of **7** (S_0 state geometry) at B3LYP/6-311+G(d,p)/ PCM = chloroform level of theory.

Excited states	Energy (eV)	Wavelength (nm)	Oscillator strength	Major contributions		
S1	3.4017	364.47	0.2871	HOMO-1	LUMO+1	19
				HOMO	LUMO	77
S2	3.4213	362.39	0.0076	HOMO-1	LUMO	19
				HOMO	LUMO+1	76

S3	3.6656	338.24	0.1854	HOMO-3	LUMO+1	46
				HOMO-2	LUMO	47
				HOMO	LUMO+5	2
S4	3.673	337.56	0.4885	HOMO-3	LUMO	47
				HOMO-2	LUMO+1	46
				HOMO	LUMO+4	3
S5	3.7299	332.4	0.0644	HOMO-1	LUMO+1	57
				HOMO	LUMO	14
				HOMO	LUMO+2	27
S6	3.7395	331.55	0.0012	HOMO-1	LUMO	77
				HOMO	LUMO+1	21
S7	3.7868	327.41	0.3218	HOMO-1	LUMO+1	20
				HOMO	LUMO	7
				HOMO	LUMO+2	71
S8	3.8665	320.66	0.0017	HOMO-3	LUMO+1	50
				HOMO-2	LUMO	49
S9	3.8667	320.64	0.0002	HOMO-3	LUMO	50
				HOMO-2	LUMO+1	49
S10	3.985	311.13	0.0044	HOMO-1	LUMO+2	95

S_1~S_{10}

Table S21. Summary of the TD-DFT calculation results of **7** (S_0 state geometry) at B3LYP/6-311G/PCM = chloroform level of theory.

Excited states	Energy (eV)	Wavelength (nm)	Oscillator strength	Major contributions		
S1	3.5167	352.55	0.3438	HOMO−1	LUMO+1	18
				HOMO	LUMO	77
S2	3.5438	349.86	0.013	HOMO−1	LUMO	19
				HOMO	LUMO+1	76
S3	3.8242	324.21	0.1276	HOMO−3	LUMO+1	32
				HOMO−2	LUMO	35
				HOMO−1	LUMO+1	20
				HOMO	LUMO	5
				HOMO	LUMO+2	3

S4	3.8315	323.59	0.3592	HOMO−3	LUMO	31
				HOMO−2	LUMO+1	29
				HOMO−1	LUMO	29
				HOMO	LUMO+1	7
S5	3.8349	323.31	0.0982	HOMO−3	LUMO+1	13
				HOMO−2	LUMO	13
				HOMO−1	LUMO+1	45
				HOMO	LUMO	10
				HOMO	LUMO+2	16
S6	3.8399	322.89	0.1421	HOMO−3	LUMO	17
				HOMO−2	LUMO+1	17
				HOMO−1	LUMO	48
				HOMO	LUMO+1	13
S7	3.9117	316.95	0.3086	HOMO−1	LUMO+1	14
				HOMO	LUMO	6
				HOMO	LUMO+2	78
S8	3.9966	310.22	0.0019	HOMO−3	LUMO+1	50
				HOMO−2	LUMO	48
S9	3.997	310.19	0.0005	HOMO−3	LUMO	49
				HOMO−2	LUMO+1	49
S10	4.0979	302.56	0.0027	HOMO−1	LUMO+2	96

S_1~S_{10}

Table S22. Summary of the TD-DFT calculation results of **10a** (S_0 state geometry) at B3LYP/6-311+G(d,p)/ PCM = chloroform level of theory.

Excited states	Energy (eV)	Wavelength (nm)	Oscillator strength	Major contributions		
S1	3.5007	354.17	0.0847	HOMO-1	LUMO	8
				HOMO-1	LUMO+1	3
				HOMO	LUMO	87
S2	3.6587	338.87	0.3522	HOMO-1	LUMO	86
				HOMO	LUMO	8
				HOMO	LUMO+1	4
S3	4.411	281.08	0.0385	HOMO-2	LUMO	25
				HOMO-1	LUMO+1	44

				HOMO-1	LUMO+2	3
				HOMO	LUMO+1	19
S4	4.4533	278.41	0.3166	HOMO-2	LUMO	6
				HOMO-1	LUMO+1	14
				HOMO	LUMO+1	73
S5	4.5982	269.64	0.0322	HOMO-2	LUMO	13
				HOMO-1	LUMO+1	19
				HOMO-1	LUMO+2	55
				HOMO	LUMO+2	8
S6	4.6476	266.77	0.3115	HOMO-2	LUMO	5
				HOMO-1	LUMO+1	4
				HOMO-1	LUMO+2	2
				HOMO	LUMO+2	83
S7	4.9174	252.13	0.4513	HOMO-3	LUMO	5
				HOMO-2	LUMO	35
				HOMO-1	LUMO+1	12
				HOMO-1	LUMO+2	24
				HOMO-1	LUMO+4	11
				HOMO	LUMO+4	7
S8	4.9737	249.28	0.0213	HOMO-3	LUMO	84
				HOMO-2	LUMO	2
				HOMO-2	LUMO+1	7
				HOMO	LUMO+4	3
S9	4.9957	248.18	0.0002	HOMO	LUMO+3	90
				HOMO	LUMO+5	4
				HOMO	LUMO+6	4
S10	5.1032	242.96	0.0004	HOMO-1	LUMO+3	94
				HOMO-1	LUMO+5	2

S_1~S_{10}

Table S23. Summary of the TD-DFT calculation results of **10a** (S_0 state geometry) at B3LYP/6-311G/PCM = chloroform level of theory.

Excited states	Energy (eV)	Wavelength (nm)	Oscillator strength	Major contributions		
S1	3.613	343.16	0.0922	HOMO−1	LUMO	2
				HOMO−1	LUMO+1	4

				HOMO	LUMO	92
S2	3.8218	324.42	0.3553	HOMO−1	LUMO	91
				HOMO	LUMO	3
				HOMO	LUMO+1	4
S3	4.5776	270.85	0.0402	HOMO−3	LUMO	3
				HOMO−2	LUMO	39
				HOMO−1	LUMO+1	45
				HOMO−1	LUMO+2	5
				HOMO	LUMO	2
S4	4.6179	268.49	0.2829	HOMO−1	LUMO	3
				HOMO	LUMO+1	89
				HOMO	LUMO+2	3
S5	4.7808	259.34	0.0146	HOMO−2	LUMO	3
				HOMO−1	LUMO+1	14
				HOMO−1	LUMO+2	29
				HOMO	LUMO+2	46
S6	4.819	257.28	0.3656	HOMO−2	LUMO	10
				HOMO−1	LUMO+1	15
				HOMO−1	LUMO+2	24
				HOMO	LUMO+1	2
				HOMO	LUMO+2	44
S7	5.1127	242.5	0.4786	HOMO−3	LUMO	5
				HOMO−2	LUMO	32
				HOMO−1	LUMO+1	17
				HOMO−1	LUMO+2	25
				HOMO−1	LUMO+3	6
				HOMO	LUMO+3	8
S8	5.1572	240.41	0.0383	HOMO−3	LUMO	81
				HOMO−2	LUMO	2
				HOMO−2	LUMO+1	8
				HOMO−1	LUMO+1	2
				HOMO−1	LUMO+2	2
S9	5.6493	219.47	0.0994	HOMO−3	LUMO+2	3
				HOMO−2	LUMO+1	34
				HOMO−1	LUMO+3	4
				HOMO	LUMO+3	48

				HOMO	LUMO+4	3
S10	5.72	216.75	0.1086	HOMO−4	LUMO	7
				HOMO−2	LUMO	5
				HOMO−2	LUMO+1	3
				HOMO−1	LUMO+3	77
				HOMO	LUMO+2	2

S_1~S_{10}

Table S24. Summary of the TD-DFT calculation results of **10b** (S_0 state geometry) at B3LYP/6-311+G(d,p)/ PCM = chloroform level of theory.

Excited states	Energy (eV)	Wavelength (nm)	Oscillator strength	Major contributions		
S1	3.3901	365.73	0.0871	HOMO-1	LUMO+1	3
				HOMO	LUMO	95
S2	3.6254	341.99	0.3368	HOMO-1	LUMO	93
				HOMO	LUMO+1	4
S3	4.373	283.52	0.3316	HOMO-1	LUMO	4
				HOMO	LUMO+1	89
				HOMO	LUMO+2	3
S4	4.4059	281.4	0.0738	HOMO-2	LUMO	30
				HOMO-1	LUMO+1	55
				HOMO-1	LUMO+2	4
				HOMO	LUMO	2
				HOMO	LUMO+2	2
S5	4.5096	274.93	0.0628	HOMO-1	LUMO+1	12
				HOMO-1	LUMO+2	13
				HOMO	LUMO+1	2
				HOMO	LUMO+2	67
S6	4.6016	269.44	0.3081	HOMO-2	LUMO	16
				HOMO-1	LUMO+1	14
				HOMO-1	LUMO+2	42
				HOMO	LUMO+2	23
S7	4.8661	254.79	0.4003	HOMO-3	LUMO	2
				HOMO-2	LUMO	37
				HOMO-1	LUMO+1	12
				HOMO-1	LUMO+2	25

				HOMO-1	LUMO+5	5
				HOMO	LUMO+5	12
S8	4.8914	253.48	0.0028	HOMO	LUMO+3	94
				HOMO	LUMO+10	2
S9	4.9565	250.14	0.022	HOMO-3	LUMO	87
				HOMO-2	LUMO+1	6
				HOMO	LUMO+5	2
S10	5.0532	245.36	0	HOMO-1	LUMO+3	95
				HOMO-1	LUMO+10	2

S_1~S_{10}

Table S25. Summary of the TD-DFT calculation results of **10b** (S_0 state geometry) at B3LYP/6-311G/PCM = chloroform level of theory.

Excited states	Energy (eV)	Wavelength (nm)	Oscillator strength	Major contributions		
S1	3.5085	353.38	0.0952	HOMO−1	LUMO+1	3
				HOMO	LUMO	95
S2	3.7889	327.23	0.3386	HOMO−1	LUMO	93
				HOMO	LUMO+1	5
S3	4.5377	273.23	0.3498	HOMO−2	LUMO	6
				HOMO−1	LUMO	3
				HOMO−1	LUMO+1	2
				HOMO	LUMO+1	84
				HOMO	LUMO+2	2
S4	4.5676	271.44	0.0225	HOMO−2	LUMO	35
				HOMO−1	LUMO+1	40
				HOMO−1	LUMO+2	5
				HOMO−1	LUMO+3	2
				HOMO	LUMO+1	5
				HOMO	LUMO+2	5
S5	4.6826	264.78	0.1174	HOMO−1	LUMO+1	10
				HOMO−1	LUMO+2	5
				HOMO	LUMO+1	3
				HOMO	LUMO+2	77
S6	4.7882	258.94	0.2801	HOMO−3	LUMO	3
				HOMO−2	LUMO	14

				HOMO−1	LUMO+1	23
				HOMO−1	LUMO+2	46
				HOMO	LUMO+2	10
S7	5.0512	245.46	0.3958	HOMO−2	LUMO	33
				HOMO−1	LUMO+1	15
				HOMO−1	LUMO+2	26
				HOMO−1	LUMO+3	4
				HOMO	LUMO+3	13
S8	5.1395	241.24	0.0526	HOMO−3	LUMO	85
				HOMO−2	LUMO+1	6
				HOMO−1	LUMO+1	4
S9	5.5838	222.04	0.1955	HOMO−2	LUMO+1	29
				HOMO	LUMO+3	56
				HOMO	LUMO+4	3
S10	5.6706	218.64	0.061	HOMO−4	LUMO	7
				HOMO−2	LUMO	5
				HOMO−1	LUMO+3	80

S_1~S_{10}

Table S26. Summary of the TD-DFT calculation results of **10c** (S_0 state geometry) at B3LYP/6-311+G(d,p)/ PCM = chloroform level of theory.

Excited states	Energy (eV)	Wavelength (nm)	Oscillator strength	Major contributions		
S1	3.4187	362.67	0.1136	HOMO	LUMO	94
S2	3.6482	339.85	0.3244	HOMO-1	LUMO	92
				HOMO	LUMO+3	3
S3	4.1241	300.63	0.1754	HOMO	LUMO+1	92
				HOMO	LUMO+2	5
S4	4.1859	296.2	0.02	HOMO	LUMO+1	4
				HOMO	LUMO+2	92
S5	4.3019	288.21	0.0585	HOMO-2	LUMO	11
				HOMO-1	LUMO+1	78
S6	4.4043	281.51	0.0216	HOMO-1	LUMO+2	95

S7	4.4244	280.23	0.2699	HOMO-2	LUMO	4
				HOMO-1	LUMO	3
				HOMO-1	LUMO+3	2
				HOMO	LUMO+3	86
S8	4.5012	275.45	0.0037	HOMO-3	LUMO	2
				HOMO-2	LUMO	16
				HOMO-1	LUMO+1	12
				HOMO-1	LUMO+3	57
				HOMO-1	LUMO+4	4
				HOMO	LUMO+3	4
S9	4.585	270.41	0.0508	HOMO-1	LUMO+2	2
				HOMO-1	LUMO+3	5
				HOMO-1	LUMO+4	15
				HOMO	LUMO+4	68
S10	4.6423	267.07	0.3172	HOMO-2	LUMO	19
				HOMO-1	LUMO+3	21
				HOMO-1	LUMO+4	32
				HOMO	LUMO+4	23

S_1~S_{10}

Table S27. Summary of the TD-DFT calculation results of **12** (S_0 state geometry) at B3LYP/6-311+G(d,p)/ PCM = chloroform level of theory.

Excited states	Energy (eV)	Wavelength (nm)	Oscillator strength	Major contributions		
S1	3.7765	328.3	0.0365	HOMO-1	LUMO+3	3
				HOMO	LUMO	95
S2	4.249	291.79	0.2231	HOMO-1	LUMO	84
				HOMO	LUMO+3	12
S3	4.4712	277.3	0	HOMO-2	LUMO+2	4
				HOMO-1	LUMO+2	4
				HOMO	LUMO+1	90
S4	4.5203	274.28	0.0001	HOMO-2	LUMO+1	5
				HOMO-1	LUMO+1	7
				HOMO	LUMO+2	87
S5	4.7921	258.73	0.814	HOMO-2	LUMO	4
				HOMO-1	LUMO	8

				HOMO-1	LUMO+4	4
				HOMO	LUMO+3	81
S6	4.9461	250.67	0.0003	HOMO-1	LUMO+1	90
				HOMO	LUMO+2	7
S7	5.0002	247.96	0	HOMO-1	LUMO+2	92
				HOMO	LUMO+1	4
S8	5.0412	245.94	0.2868	HOMO-2	LUMO	86
				HOMO-1	LUMO	4
				HOMO-1	LUMO+4	4
				HOMO	LUMO+3	4
S9	5.062	244.93	0.0215	HOMO-1	LUMO+3	54
				HOMO	LUMO+4	41
S10	5.2596	235.73	0.0058	HOMO	LUMO+5	91
				HOMO	LUMO+8	5

S_1~S_{10}

Table S28. Summary of the TD-DFT calculation results of **12** (S_0 state geometry) at B3LYP/6-311G/PCM = chloroform level of theory.

Excited states	Energy (eV)	Wavelength (nm)	Oscillator strength	Major contributions		
S1	3.9431	314.44	0.0447	HOMO−1	LUMO+2	4
				HOMO	LUMO	94
S2	4.465	277.68	0.1635	HOMO−1	LUMO	78
				HOMO	LUMO+2	17
S3	4.6497	266.65	0	HOMO−2	LUMO+3	4
				HOMO−1	LUMO+3	3
				HOMO	LUMO+1	92
S4	4.6997	263.81	0.0007	HOMO−2	LUMO+1	4
				HOMO−1	LUMO+1	5
				HOMO	LUMO+3	90
S5	4.9746	249.23	0.9298	HOMO−2	LUMO	5
				HOMO−1	LUMO	11
				HOMO−1	LUMO+4	4
				HOMO	LUMO+2	77

S6	5.1391	241.26	0	HOMO−1	LUMO+1	92
				HOMO	LUMO+3	5
S7	5.1918	238.81	0	HOMO−3	LUMO+1	2
				HOMO−1	LUMO+3	94
				HOMO	LUMO+1	3
S8	5.2568	235.85	0.3247	HOMO−2	LUMO	85
				HOMO−1	LUMO	6
				HOMO−1	LUMO+4	4
				HOMO	LUMO+2	3
S9	5.3109	233.45	0.0504	HOMO−1	LUMO+2	67
				HOMO	LUMO	3
				HOMO	LUMO+4	26
S10	5.7612	215.21	0.4407	HOMO−1	LUMO	3
				HOMO−1	LUMO+4	14
				HOMO	LUMO+5	76

S_1~S_{10}

7. Supplementary Note 8: *Theoretical prediction of the pKa values of **3a** and its radical cation [**3a**]$^{\bullet+}$*

$$pKa = \frac{G\ sol\ (H^{+}) + G\ sol\ (conjugate\ base^{-}) - G\ sol\ (Acid)}{2.3\ RT}$$

We optimized the structures of the substrate **3a** and its radical cation **3a**$^{\bullet+}$ (representing the acidic forms) and the neutral radical **3a**$^{\bullet}$ and anion **3a**$^{-}$ (representing the conjugate basic forms) in MeCN using the B3LYP/6-311+G(d,p) level of theory. Additionally, we accounted for entropy and enthalpy corrections and optimized the solvated proton. Using these refined energy values, we applied the previously established equation to obtain the final results.

T(K)	298	
R (kcal/Kmol)	0.001987	
Etotal, H (kcal/mol)	−0.42899	−269.1950859
Molecule	$E_{(total)}$ Hartree	$E_{(total)}$ kcal/mol
**3a-OH**	−1016.6804	−637976.1011
**3a-O**$^{-}$	−1016.2095	−637680.6071
**[3a-OH]**$^{\bullet+}$	−1016.4784	−637849.3443
**3a-O**$^{\bullet}$	−1016.0606	−637587.171
pKa (on MeCN)	*ΔG* (kcal/mol)	*pKa*
**[3a-OH]**$^{\bullet+}$	−7.02	−5.15
**3a-OH**	26.30	19.29

8. Supplementary Note 9: *DFT–calculated redox behavior of reported carbazole derivatives*

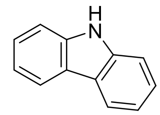

11

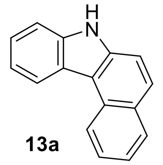

13a

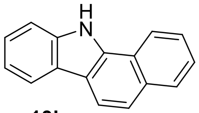

13b

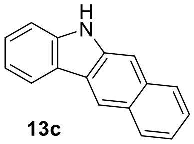

13c

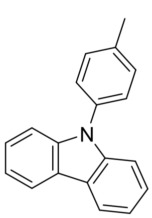

13d

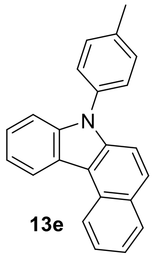

13e

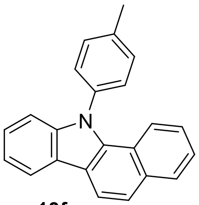

13f

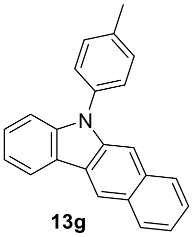

13g

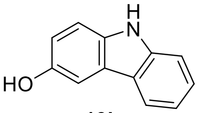

13h

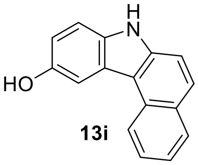

13i

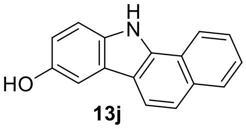

13j

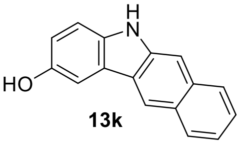

13k

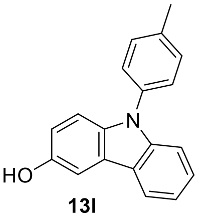

13l

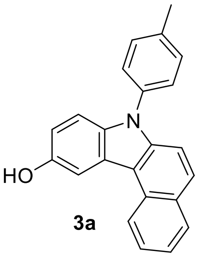

3a

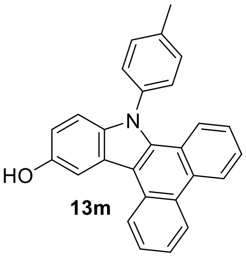

13m

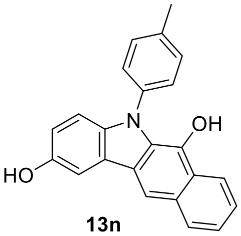

13n

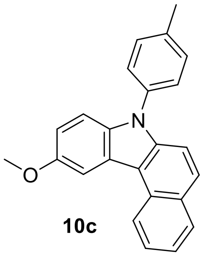

10c

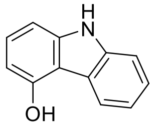

13o

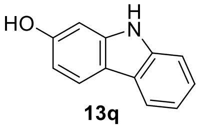

13q

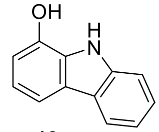

13s

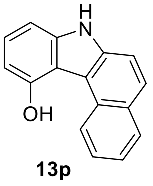

13p

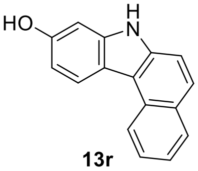

13r

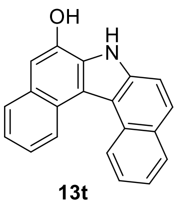

13t

Table S29. DFT−calculated redox behavior of previously reported carbazole derivatives

Compound number	DFT calculation of the redox potentials of carbazoles			
	Reduction Pot. ground state $E^{1/2}$(A/A•−)	Oxidation Pot. ground state $E^{1/2}$(A•+/A)	Oxidation Pot. excited state $E^{1/2}$(A*/A•−)	Reduction Pot. excited state $E^{1/2}$(A•+/A*)
**11**	−2.67	1.28	0.99	−2.38
**13a**	−2.36	1.10	0.89	−2.15
**13b**	−2.50	1.06	0.84	−2.29
**13c**	−2.23	0.95	0.75	−2.03
**13d**	−2.66	1.19	0.89	−2.36
**13e**	−2.34	1.09	0.87	−2.13
**13f**	−2.53	0.99	0.79	−2.33
**13g**	−2.24	0.91	0.71	−2.04
**13h**	−2.61	1.02	0.79	−2.38
**13i**	−2.32	1.07	0.86	−2.11
**13j**	−2.52	0.99	0.75	−2.29
**13k**	−2.19	0.78	0.61	−2.02
**13l**	−2.60	0.96	0.73	−2.36
**3a**	−2.31	1.02	0.80	−2.09
**13m**	−2.38	0.99	0.75	−2.14
**13n**	−2.27	0.62	0.51	−2.16
**10c**	−2.34	0.96	0.76	−2.14
**13o**	−2.74	1.16	0.91	−2.49
**13p**	−2.36	1.02	0.79	−2.13
**13q**	−2.77	1.17	0.96	−2.56
**13r**	−2.40	0.90	0.73	−2.24
**13s**	−2.69	1.12	0.89	−2.46
**13t**	−2.33	0.94	0.72	−2.11

Calculated at B3LYP/6-311+G(d,p)/PCM = MeCN level of theory.

Table S30. Predicted physicochemical variables of carbazole derivatives at different level of theory

Compound number	DFT calculation of the redox potentials of carbazoles			
	Oxid Pot. ground state $E^{1/2}(A^{\bullet+}/A)$	Red. Pot. ground state $E^{1/2}(A/A^{\bullet-})$	Oxid Pot. excited state $E^{1/2}(A^{*}/A^{\bullet-})$	Red. Pot. excited state $E^{1/2}(A^{\bullet+}/A^{*})$
**3a**	0.96	−2.38	0.75	−2.17
**3b**	1.02	−2.36	0.80	−2.14
**3c**	0.87	−2.42	0.71	−2.25
**3d**	1.04	−2.26	0.81	−2.03
**3e**	1.10	−1.92	0.81	−1.64
**6a**	0.98	−2.32	0.80	−2.13
**6b**	1.09	−2.17	0.79	−1.88
**7**	0.98	−2.35	0.79	−2.16
**10a**	0.94	−2.43	0.73	−2.23
**10b**	0.84	−2.40	0.65	−2.21
**10c**	0.91	−2.40	0.70	−2.20
**12**	1.33	−2.40	1.09	−2.16

Calculated at B3LYP/6-31+G(d,p)/PCM = MeCN level of theory.

9. Supplementary Note 10: *Comparative analysis of the aromaticity of various carbazoles*

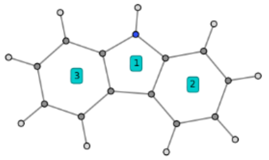

Ring	NICS(0)$_{iso}$	NICS(0)$_{zz}$	NICS(1)$_{iso}$	NICS(1)$_{zz}$	NICS(−1)$_{iso}$	NICS(−1)$_{zz}$	HOMA
**1**	−9.2680	2.3769	−8.3058	−21.2713	−8.3058	−21.2713	0.599
**2**	−9.5036	−13.5742	−10.5941	−28.6978	−10.5941	−28.6978	0.929
**3**	−9.5036	−13.5742	−10.5941	−28.6978	−10.5941	−28.6978	0.929

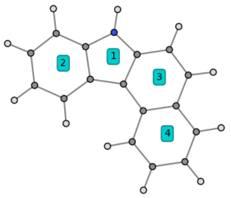

Ring	NICS(0)$_{iso}$	NICS(0)$_{zz}$	NICS(1)$_{iso}$	NICS(1)$_{zz}$	NICS(−1)$_{iso}$	NICS(−1)$_{zz}$	HOMA
**1**	−10.2603	0.6836	−9.0433	−22.6875	−9.0433	−22.6875	0.624
**2**	−9.7833	−14.3840	−10.8597	−29.0086	−10.8597	−29.0085	0.909
**3**	−8.4550	−7.8633	−9.8058	−24.5392	−9.8058	−24.5392	0.691
**4**	−8.5686	−12.5324	−10.5031	−28.1649	−10.5031	−28.1649	0.795

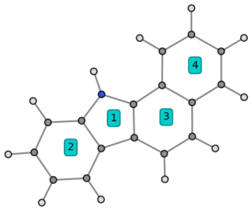

Ring	NICS(0)$_{iso}$	NICS(0)$_{zz}$	NICS(1)$_{iso}$	NICS(1)$_{zz}$	NICS(−1)$_{iso}$	NICS(−1)$_{zz}$	HOMA
**1**	−9.6546	1.9231	−8.7879	−22.1434	−8.7879	−22.1434	0.699
**2**	−9.5337	−13.4338	−10.6141	−28.5846	−10.6141	−28.5846	0.925
**3**	−8.5102	−8.0939	−9.9570	−25.0374	−9.9570	−25.0374	0.720
**4**	−8.3940	−11.6975	−10.2828	−27.6125	−10.2828	−27.6125	0.801

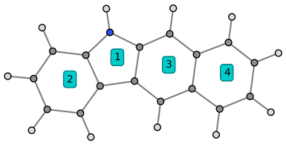

Ring	NICS(0)$_{iso}$	NICS(0)$_{zz}$	NICS(1)$_{iso}$	NICS(1)$_{zz}$	NICS(−1)$_{iso}$	NICS(−1)$_{zz}$	HOMA
**1**	−7.5029	7.9210	−6.9279	−16.7371	−6.9279	−16.7371	0.500
**2**	−9.1571	−12.4209	−10.3219	−27.7162	−10.3219	−27.7162	0.939
**3**	−10.8565	−14.7769	−11.7160	−30.5639	−11.7160	−30.5639	0.692
**4**	−8.3262	−11.9120	−10.3679	−27.7407	−10.3679	−27.7407	0.702

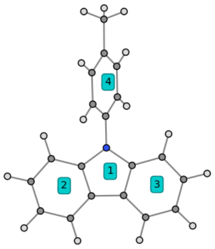

Ring	NICS(0)$_{iso}$	NICS(0)$_{zz}$	NICS(1)$_{iso}$	NICS(1)$_{zz}$	NICS(−1)$_{iso}$	NICS(−1)$_{zz}$	HOMA
**1**	−8.4890	3.3173	−7.8878	−19.8017	−7.8954	−19.8168	0.581
**2**	−9.2729	−12.8398	−10.3105	−27.7872	−10.4938	−28.4435	0.935
**3**	−9.2692	−12.8476	−10.4834	−28.4393	−10.3081	−27.7971	0.935
**4**	−7.5291	−3.4239	−8.8578	−2.4979	−8.9704	−2.5683	0.980

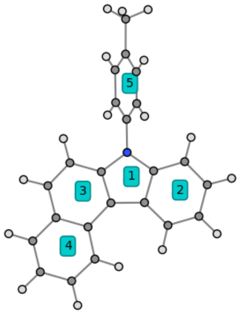

Ring	NICS(0)$_{iso}$	NICS(0)$_{zz}$	NICS(1)$_{iso}$	NICS(1)$_{zz}$	NICS(−1)$_{iso}$	NICS(−1)$_{zz}$	HOMA
**1**	−9.5122	1.1561	−8.4690	−21.5062	−8.4325	−21.4253	0.608
**2**	−9.5903	−13.7561	−10.4630	−28.3131	−10.5898	−28.7800	0.916
**3**	−8.2486	−7.4366	−9.7432	−24.4676	−9.5626	−23.9827	0.699
**4**	−8.4670	−12.3901	−10.3894	−27.9727	−10.3730	−27.9647	0.795
**5**	−7.4852	−2.7404	−8.9884	−0.5807	−8.8564	−0.5208	0.980

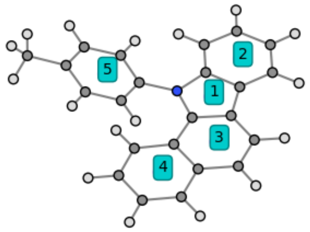

Ring	NICS(0)iso	NICS(0)zz	NICS(1)iso	NICS(1)zz	NICS(−1)iso	NICS(−1)zz	HOMA
1	−9.3679	0.4602	−8.3834	−21.9151	−8.3837	−21.9155	0.652
2	−9.5742	−13.5155	−10.5599	−28.5934	−10.5601	−28.5938	0.938
3	−8.4589	−8.0923	−9.7771	−24.7050	−9.7771	−24.7050	0.676
4	−8.6231	−11.8976	−10.4928	−27.6801	−10.4928	−27.6801	0.776
5	−7.4705	−1.6881	−9.8108	3.2406	−9.8082	2.9057	0.981

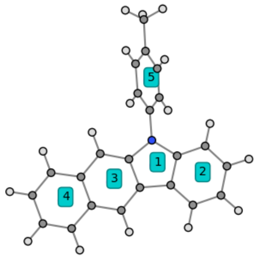

Ring	NICS(0)iso	NICS(0)zz	NICS(1)iso	NICS(1)zz	NICS(−1)iso	NICS(−1)zz	HOMA
1	−6.8255	8.4736	−6.5855	−15.6197	−6.6233	−15.6274	0.485
2	−8.9802	−11.8731	−10.2482	−27.4634	−10.0809	−27.0768	0.945
3	−10.6312	−14.1898	−11.4923	−29.9044	−11.6759	−30.1066	0.705
4	−8.1625	−11.2375	−10.2030	−27.1930	−10.2655	−27.5218	0.709
5	−7.4045	−2.9686	−8.8992	−1.5125	−8.7644	−1.4869	0.980

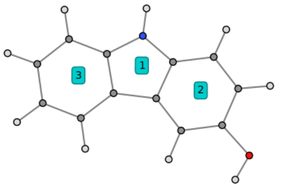

Ring	NICS(0)iso	NICS(0)zz	NICS(1)iso	NICS(1)zz	NICS(−1)iso	NICS(−1)zz	HOMA
1	−9.5607	2.2841	−8.4126	−21.4220	−8.4125	−21.4220	0.611
2	−10.5273	−12.0257	−10.2901	−26.4935	−10.2900	−26.4933	0.937
3	−9.4472	−13.1578	−10.5523	−28.3009	−10.5523	−28.3008	0.922

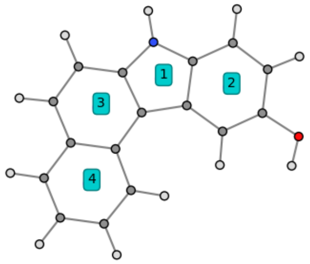

Ring	NICS(0)iso	NICS(0)zz	NICS(1)iso	NICS(1)zz	NICS(−1)iso	NICS(−1)zz	HOMA
1	−10.5544	0.5269	−9.0461	−22.7616	−9.0461	−22.7617	0.641
2	−10.7294	−12.6547	−10.4866	−26.6637	−10.4866	−26.6638	0.916
3	−8.3547	−7.3528	−9.6659	−24.0069	−9.6659	−24.0070	0.681
4	−8.6114	−12.4947	−10.5194	−28.1375	−10.5193	−28.1373	0.798

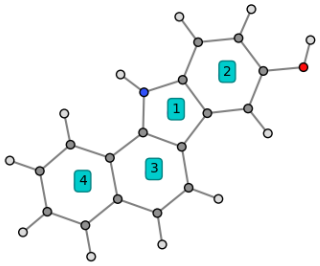

Ring	NICS(0)iso	NICS(0)zz	NICS(1)iso	NICS(1)zz	NICS(−1)iso	NICS(−1)zz	HOMA
1	−9.5584	2.7467	−8.6806	−21.6064	−8.6806	−21.6063	0.675
2	−10.6981	−12.2766	−10.4247	−26.5937	−10.4249	−26.5942	0.931
3	−8.4042	−7.6420	−9.8325	−24.5864	−9.8325	−24.5864	0.715
4	−8.3572	−11.5205	−10.2704	−27.5439	−10.2704	−27.5438	0.805

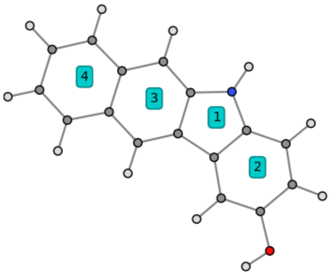

Ring	NICS(0)iso	NICS(0)zz	NICS(1)iso	NICS(1)zz	NICS(−1)iso	NICS(−1)zz	HOMA
1	−7.9110	7.3932	−7.1249	−17.1848	−7.1249	−17.1848	0.510
2	−10.2310	−11.1890	−10.1173	−25.7485	−10.1173	−25.7486	0.949
3	−10.9344	−14.7328	−11.7326	−30.4390	−11.7326	−30.4390	0.690
4	−8.2647	−11.6091	−10.3119	−27.4763	−10.3119	−27.4763	0.696

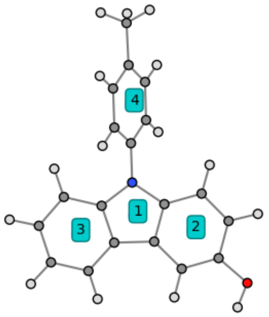

Ring	NICS(0)$_{iso}$	NICS(0)$_{zz}$	NICS(1)$_{iso}$	NICS(1)$_{zz}$	NICS(−1)$_{iso}$	NICS(−1)$_{zz}$	HOMA
1	−8.7602	3.3751	−8.0539	−19.9448	−7.9804	−19.8323	0.5920
2	−10.2530	−11.2105	−10.2253	−26.1579	−10.0262	−25.5149	0.944
3	−9.2054	−12.2831	−10.2030	−27.3662	−10.4031	−28.0040	0.929
4	−7.6025	−3.6690	−8.9348	−3.1562	−9.0643	−3.3402	0.979

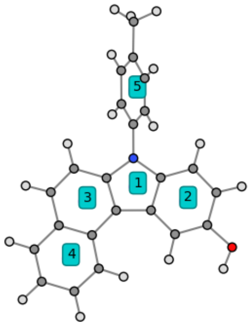

Ring	NICS(0)$_{iso}$	NICS(0)$_{zz}$	NICS(1)$_{iso}$	NICS(1)$_{zz}$	NICS(−1)$_{iso}$	NICS(−1)$_{zz}$	HOMA
1	−9.7658	1.2407	−8.5344	−21.4850	−8.5686	−21.5141	0.623
2	−10.5019	−12.0067	−10.1967	−26.3946	−10.0582	−25.9476	0.924
3	−8.0905	−6.7952	−9.4006	−23.4114	−9.5982	−23.8830	0.689
4	−8.4857	−12.3192	−10.3706	−27.9013	−10.3833	−27.9477	0.798
5	−7.5565	−3.0225	−8.8869	−1.2074	−9.0423	−1.3657	0.980

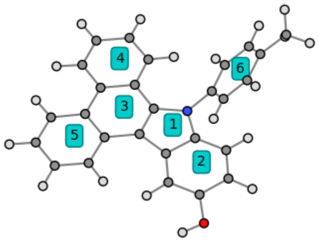

Ring	NICS(0)$_{iso}$	NICS(0)$_{zz}$	NICS(1)$_{iso}$	NICS(1)$_{zz}$	NICS(−1)$_{iso}$	NICS(−1)$_{zz}$	HOMA
1	−9.4079	2.0333	−8.0523	−20.5322	−8.0590	−20.5456	0.648
2	−10.3305	−11.2452	−10.0247	−25.5675	−10.0358	−25.5838	0.929
3	−4.8723	4.6978	−6.8632	−14.4751	−6.8558	−14.4609	0.353
4	−8.1461	−9.8449	−10.1635	−26.3023	−10.1484	−26.2457	0.854
5	−7.9225	−10.1123	−9.9603	−26.4677	−9.9571	−26.4817	0.867
6	−7.4936	−1.6622	−8.8468	4.0078	−8.8461	3.6805	0.981

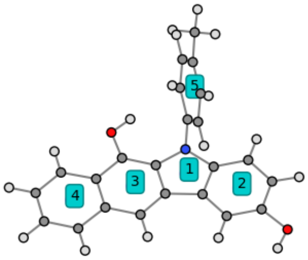

Ring	NICS(0)$_{iso}$	NICS(0)$_{zz}$	NICS(1)$_{iso}$	NICS(1)$_{zz}$	NICS(−1)$_{iso}$	NICS(−1)$_{zz}$	HOMA
1	−7.6370	7.9308	−6.5198	−15.4313	−6.6363	−15.6497	0.495
2	−10.0984	−10.3527	−9.9031	−24.8945	−10.0585	−25.2540	0.957
3	−12.2157	−14.4880	−11.8175	−29.2200	−11.5654	−28.6179	0.717
4	−8.2758	−10.7682	−10.1841	−26.9623	−10.1469	−26.8947	0.711
5	−7.3777	−2.4623	−8.8620	1.0143	−8.9481	0.6848	0.975

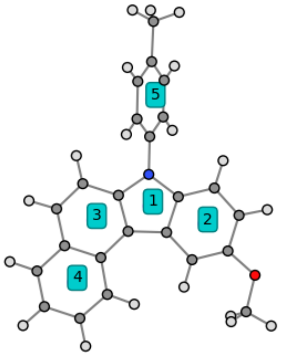

Ring	NICS(0)$_{iso}$	NICS(0)$_{zz}$	NICS(1)$_{iso}$	NICS(1)$_{zz}$	NICS(−1)$_{iso}$	NICS(−1)$_{zz}$	HOMA
1	−9.9104	0.9373	−8.7220	−21.8183	−8.7194	−21.8043	0.629
2	−10.1434	−11.1755	−10.0439	−25.7418	−10.2287	−26.1949	0.914
3	−8.1213	−6.7774	−9.6062	−23.8596	−9.4137	−23.4096	0.687
4	−8.4730	−12.2843	−10.3834	−27.9678	−10.3501	−27.8712	0.798
5	−7.5303	−2.9675	−9.0039	−1.3499	−8.8916	−1.3561	0.980

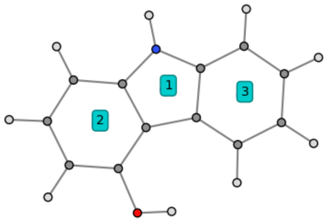

Ring	NICS(0)$_{iso}$	NICS(0)$_{zz}$	NICS(1)$_{iso}$	NICS(1)$_{zz}$	NICS(−1)$_{iso}$	NICS(−1)$_{zz}$	HOMA
1	−9.8974	2.6897	−8.2464	−20.6239	−8.2456	−20.6238	0.574
2	−10.7996	−12.8022	−10.4221	−26.6937	−10.4226	−26.6948	0.926
3	−9.8626	−14.2214	−10.7667	−28.9579	−10.7650	−28.9536	0.923

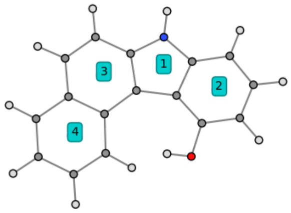

Ring	NICS(0)$_{iso}$	NICS(0)$_{zz}$	NICS(1)$_{iso}$	NICS(1)$_{zz}$	NICS(−1)$_{iso}$	NICS(−1)$_{zz}$	HOMA
1	−10.5322	1.3442	−8.9155	−22.2335	−8.4726	−21.6556	0.564
2	−10.5919	−12.6806	−10.7902	−27.2868	−9.9507	−25.2675	0.897
3	−8.4181	−8.0327	−9.3772	−24.2925	−10.0651	−25.0089	0.663
4	−8.3452	−11.8731	−9.7612	−25.6208	−10.8782	−28.6147	0.784

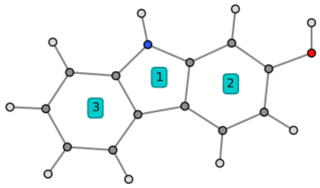

Ring	NICS(0)$_{iso}$	NICS(0)$_{zz}$	NICS(1)$_{iso}$	NICS(1)$_{zz}$	NICS(−1)$_{iso}$	NICS(−1)$_{zz}$	HOMA
1	−9.0302	3.7801	−8.0030	−20.1961	−8.0030	−20.1961	0.603
2	−9.9484	−10.5889	−9.8207	−25.1978	−9.8208	−25.1980	0.931
3	−9.5872	−13.5158	−10.5742	−28.5804	−10.5742	−28.5804	0.931

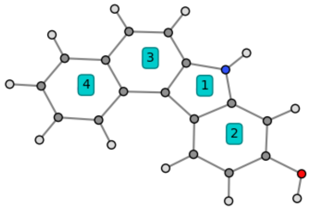

Ring	NICS(0)$_{iso}$	NICS(0)$_{zz}$	NICS(1)$_{iso}$	NICS(1)$_{zz}$	NICS(−1)$_{iso}$	NICS(−1)$_{zz}$	HOMA
1	−9.7148	2.8474	−8.5809	−21.0273	−8.5809	−21.0273	0.621
2	−10.5830	−12.0535	−10.3724	−26.0206	−10.3723	−26.0203	0.912
3	−8.7500	−8.4450	−9.9839	−24.9823	−9.9840	−24.9824	0.702
4	−8.5072	−12.2278	−10.4623	−27.9607	−10.4624	−27.9607	0.787

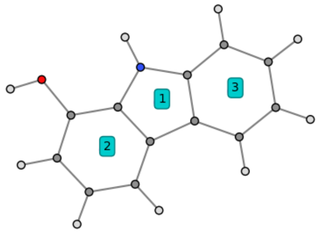

Ring	NICS(0)$_{iso}$	NICS(0)$_{zz}$	NICS(1)$_{iso}$	NICS(1)$_{zz}$	NICS(−1)$_{iso}$	NICS(−1)$_{zz}$	HOMA
1	−9.5598	2.2735	−8.3808	−21.5257	−8.3809	−21.5259	0.613
2	−11.0353	−13.2632	−10.6840	−27.3982	−10.6842	−27.3987	0.936
3	−9.4732	−13.3470	−10.5556	−28.5191	−10.5556	−28.5191	0.925

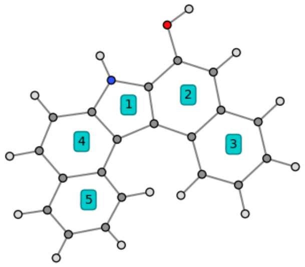

Ring	NICS(0)$_{iso}$	NICS(0)$_{zz}$	NICS(1)$_{iso}$	NICS(1)$_{zz}$	NICS(−1)$_{iso}$	NICS(−1)$_{zz}$	HOMA
1	−11.1936	−1.1506	−9.3020	−24.2378	−9.2894	−24.0068	0.632
2	−9.2898	−6.2029	−9.8948	−22.1378	−8.9528	−21.4567	0.618
3	−8.4459	−11.5597	−10.8968	−27.9241	−9.5064	−24.0291	0.798
4	−7.9000	−6.4707	−8.8670	−22.4462	−9.8894	−23.0876	0.624
5	−8.4392	−11.7950	−9.5629	−23.6473	−11.0173	−28.1570	0.791

10. Supplementary Note 11: *Cartesian coordinates*

3a (ground state) b3lyp/6-311+g(d,p) scrf=(cpcm,solvent=acetonitrile)

```
# opt freq b3lyp/6-311+g(d,p)
scrf=(cpcm,solvent=acetonitrile)
```

Charge = 0, Multiplicity = 1, Point group = C1

Electronic Energy = -1016.964331 Hartree

Number of imaginary frequencies = 0

Sum of electronic and zero-point Energies = -1016.630836 Hartree

Sum of electronic and thermal Energies = -1016.611216 Hartree

Sum of electronic and thermal Enthalpies = -1016.610271 Hartree

Sum of electronic and thermal Free Energies = -1016.680371 Hartree

	Cartesian Coordinates				Cartesian Coordinates		
	X	***Y***	***Z***		***X***	***Y***	***Z***
C	-0.264170	3.747721	0.090012	C	1.140644	3.698151	0.081498
C	1.820650	2.487133	0.050477	C	1.084742	1.287981	0.022080
C	-0.333678	1.359694	0.022315	C	-1.011677	2.578713	0.061536
C	1.426756	-0.118721	-0.000925	C	0.207610	-0.824767	-0.013707
N	-0.853121	0.066776	0.001264	C	2.659287	-0.846683	-0.021889
C	2.589123	-2.280169	-0.061619	C	1.319463	-2.935558	-0.083050
C	0.141929	-2.235302	-0.060587	C	3.942971	-0.243996	-0.006383
C	5.093026	-1.006482	-0.028130	C	5.021278	-2.414289	-0.066840
C	3.789862	-3.032497	-0.083087	C	-2.240823	-0.274058	-0.001290
C	-3.034341	0.028630	-1.109080	C	-4.387508	-0.300421	-1.104878
C	-4.974920	-0.944679	-0.008924	C	-4.161236	-1.249141	1.088833
C	-2.809595	-0.911996	1.101841	O	1.795764	4.909350	0.108139
C	-6.446801	-1.277537	-0.001990	H	-0.753756	4.713694	0.119341
H	2.902990	2.489642	0.050313	H	-2.093703	2.618802	0.069464
H	1.300820	-4.019375	-0.119583	H	-0.813130	-2.744973	-0.079857
H	4.031690	0.832678	0.024339	H	6.060635	-0.517199	-0.014951
H	5.930715	-3.003786	-0.083822	H	3.722270	-4.115232	-0.113213
H	-2.590450	0.512394	-1.971331	H	-4.992823	-0.062102	-1.973142
H	-4.589651	-1.751226	1.949850	H	-2.195621	-1.142578	1.964675
H	2.749725	4.767800	0.099136	H	-6.810966	-1.489300	-1.009633
H	-6.655657	-2.143578	0.629409	H	-7.032899	-0.438244	0.387767

3b (ground state) b3lyp/6-311+g(d,p) scrf=(cpcm,solvent=acetonitrile)

```
# opt freq b3lyp/6-311+g(d,p)
scrf=(cpcm,solvent=acetonitrile)
```

Charge = 0, Multiplicity = 1, Point group = C1

Electronic Energy = -1437.258629 Hartree

Number of imaginary frequencies = 0

Sum of electronic and zero-point Energies = -1436.961693 Hartree

Sum of electronic and thermal Energies = -1436.942790 Hartree

Sum of electronic and thermal Enthalpies = -1436.941846 Hartree

Sum of electronic and thermal Free Energies = -1437.009960 Hartree

	**Cartesian Coordinates**				**Cartesian Coordinates**		
	X	***Y***	***Z***		***X***	***Y***	***Z***
C	-0.251921	3.800909	0.100562	C	-1.651267	3.670856	0.090214
C	-2.260422	2.422621	0.055574	C	-1.456755	1.268329	0.023024
C	-0.045534	1.421627	0.022398	C	0.561544	2.676916	0.068530
C	-1.718971	-0.155882	-0.001581	C	-0.463664	-0.793353	-0.015318
N	0.546438	0.158290	-0.000679	C	-2.909551	-0.950563	-0.024831
C	-2.760342	-2.377769	-0.067955	C	-1.456798	-2.961752	-0.092374
C	-0.319628	-2.197537	-0.068335	C	-4.224407	-0.419461	-0.008558
C	-5.330414	-1.244283	-0.031991	C	-5.181089	-2.645938	-0.073468
C	-3.917707	-3.195354	-0.091040	C	1.947319	-0.102966	-0.001749
C	2.536780	-0.767642	1.075687	C	3.904390	-1.029728	1.075007
C	4.673210	-0.605330	-0.004030	C	4.104449	0.066203	-1.081807
C	2.733597	0.309725	-1.079663	O	-2.374498	4.841716	0.119950
Cl	6.406458	-0.925133	-0.005745	H	0.181442	4.793159	0.134640
H	-3.341001	2.362766	0.056240	H	1.639174	2.780962	0.079846
H	-1.377834	-4.042636	-0.133312	H	0.661182	-2.655277	-0.092181
H	-4.372786	0.650493	0.024268	H	-6.323539	-0.809271	-0.018045
H	-6.056712	-3.284506	-0.091700	H	-3.790453	-4.272558	-0.123628
H	1.929290	-1.075670	1.917801	H	4.363587	-1.546130	1.907809
H	4.716133	0.384942	-1.915687	H	2.274484	0.815517	-1.920175
H	-3.318751	4.645943	0.107161				

3c (ground state) b3lyp/6-311+g(d,p) scrf=(cpcm,solvent=acetonitrile)

```
# opt freq b3lyp/6-311+g(d,p)
scrf=(cpcm,solvent=acetonitrile)
```

Charge = 0, Multiplicity = 1, Point group = C1

Electronic Energy = -1056.291712 Hartree

Number of imaginary frequencies = 0

Sum of electronic and zero-point Energies = -1055.930814 Hartree

Sum of electronic and thermal Energies = -1055.909465 Hartree

Sum of electronic and thermal Enthalpies = -1055.908520 Hartree

Sum of electronic and thermal Free Energies = -1055.982392 Hartree

	Cartesian Coordinates				Cartesian Coordinates		
	X	*Y*	*Z*		*X*	*Y*	*Z*
C	0.445737	3.510358	0.064454	C	-0.969155	3.525468	0.056784
C	-1.722714	2.360167	0.032805	C	-1.069464	1.114705	0.009725
C	0.347976	1.104482	0.008544	C	1.097708	2.281142	0.041268
C	-1.494818	-0.269117	-0.005109	C	-0.319584	-1.046200	-0.014610
N	0.791882	-0.217115	-0.005864	C	-2.767562	-0.922876	-0.021421
C	-2.783338	-2.358445	-0.053021	C	-1.555225	-3.088265	-0.071456
C	-0.338028	-2.457922	-0.053795	C	-4.012662	-0.243515	-0.008846
C	-5.206590	-0.935076	-0.025514	C	-5.219541	-2.345175	-0.056109
C	-4.027771	-3.036700	-0.069526	C	2.156580	-0.639432	-0.005490
C	2.687299	-1.301017	1.102983	C	4.018126	-1.712495	1.095647
C	4.848929	-1.464092	-0.003612	C	4.302503	-0.788287	-1.101604
C	2.969497	-0.384746	-1.111183	O	-1.561404	4.770188	0.077344
C	6.283345	-1.932987	-0.015287	C	1.213941	4.806202	0.097561
H	-2.802966	2.435667	0.034355	H	2.180749	2.247058	0.048192
H	-1.600514	-4.171534	-0.101805	H	0.585164	-3.023504	-0.070791
H	-4.034791	0.836913	0.015451	H	-6.143073	-0.388486	-0.014723
H	-6.162898	-2.878899	-0.069189	H	-4.026005	-4.121705	-0.093498
H	2.061475	-1.485755	1.968354	H	4.418226	-2.225021	1.964154
H	4.924691	-0.579441	-1.965523	H	2.557774	0.126358	-1.973624
H	-2.520871	4.674174	0.068415	H	6.696924	-1.972252	0.994654
H	6.359347	-2.939957	-0.439681	H	6.911830	-1.275683	-0.619894
H	0.980740	5.429706	-0.770987	H	0.962469	5.395822	0.984387
H	2.288654	4.617794	0.104926				

3d (ground state) b3lyp/6-311+g(d,p) scrf=(cpcm,solvent=acetonitrile)

```
# opt freq b3lyp/6-311+g(d,p) scrf=(cpcm,solvent=acetonitrile)
```

Charge = 0, Multiplicity = 1, Point group = C1

Electronic Energy = -3590.507622 Hartree

Number of imaginary frequencies = 0

Sum of electronic and zero-point Energies = -3590.184491 Hartree

Sum of electronic and thermal Energies = -3590.163265 Hartree

Sum of electronic and thermal Enthalpies = -3590.162321 Hartree

Sum of electronic and thermal Free Energies = -3590.237311 Hartree

	Cartesian Coordinates				Cartesian Coordinates		
	X	*Y*	*Z*		*X*	*Y*	*Z*
C	-1.449493	3.753997	0.086884	C	-0.043882	3.724194	0.078659
C	0.653008	2.522786	0.048855	C	-0.066635	1.313962	0.021614

C	-1.485436	1.365935	0.022022	C	-2.180744	2.575182	0.059632
C	0.292839	-0.088162	-0.000980	C	-0.917492	-0.811000	-0.013748
N	-1.987645	0.065924	0.001251	C	1.530996	-0.803164	-0.020802
C	1.478904	-2.236791	-0.059432	C	0.218962	-2.908933	-0.080806
C	-0.966171	-2.222161	-0.059231	C	2.803763	-0.176845	-0.004518
C	3.948016	-0.940236	-0.025202	C	3.916739	-2.345697	-0.062913
C	2.688985	-2.970720	-0.079360	C	-3.371616	-0.293150	-0.001284
C	-4.162882	-0.021134	-1.118238	C	-5.511539	-0.368351	-1.114159
C	-6.095522	-0.999556	-0.008845	C	-5.283508	-1.272430	1.098507
C	-3.936466	-0.917634	1.111295	Br	5.661855	-0.061297	-0.001247
O	0.593362	4.944007	0.104147	C	-7.562777	-1.352049	-0.002240
H	-1.952153	4.713222	0.115019	H	1.735164	2.542300	0.048579
H	-3.263196	2.599774	0.067141	H	0.214208	-3.992611	-0.116354
H	-1.915245	-2.742677	-0.078132	H	2.882359	0.898685	0.025613
H	4.834188	-2.918735	-0.078626	H	2.642187	-4.054087	-0.108693
H	-3.721020	0.452863	-1.986920	H	-6.115795	-0.154438	-1.989432
H	-5.709593	-1.763837	1.966765	H	-3.323259	-1.124121	1.980752
H	1.549427	4.817125	0.094903	H	-7.922187	-1.575560	-1.008982
H	-7.761202	-2.216354	0.634908	H	-8.160508	-0.517952	0.380910

3e (ground state) b3lyp/6-311+g(d,p) scrf=(cpcm,solvent=acetonitrile)

```
# opt freq b3lyp/6-311+g(d,p)
scrf=(cpcm,solvent=acetonitrile)
```

Charge = 0, Multiplicity = 1, Point group = C1

Electronic Energy = -1109.236764 Hartree

Number of imaginary frequencies = 0

Sum of electronic and zero-point Energies = -1108.904635 Hartree

Sum of electronic and thermal Energies = -1108.883172 Hartree

Sum of electronic and thermal Enthalpies = -1108.882228 Hartree

Sum of electronic and thermal Free Energies = -1108.956851 Hartree

	**Cartesian Coordinates**				**Cartesian Coordinates**		
	X	***Y***	***Z***		***X***	***Y***	***Z***
C	-0.736257	3.749871	0.084573	C	0.669829	3.706746	0.075564
C	1.354818	2.499148	0.046217	C	0.622627	1.297481	0.020612
C	-0.795620	1.362988	0.021926	C	-1.479311	2.579379	0.058685
C	0.967206	-0.107348	-0.001485	C	-0.250576	-0.817721	-0.013553
N	-1.311258	0.068816	0.001797	C	2.196874	-0.837646	-0.020902
C	2.130809	-2.272759	-0.058759	C	0.864636	-2.929978	-0.079455
C	-0.312016	-2.227713	-0.058100	C	3.471964	-0.230926	-0.004977
C	4.628611	-0.998892	-0.025107	C	4.562920	-2.414918	-0.062390

C	3.332016	-3.024087	-0.078344	C	-2.699423	-0.276570	-0.000667
C	-3.483634	-0.011110	-1.123945	C	-4.835611	-0.344967	-1.119809
C	-5.429329	-0.955521	-0.008061	C	-4.623885	-1.221847	1.105675
C	-3.273365	-0.880595	1.118338	O	1.317015	4.920389	0.099613
C	-6.900043	-1.293024	-0.001706	C	5.903027	-0.352244	-0.007594
N	6.938570	0.163764	0.006271	H	-1.228982	4.714231	0.112228
H	2.437064	2.508124	0.044786	H	-2.561420	2.614475	0.066651
H	0.846416	-4.013254	-0.114165	H	-1.266734	-2.738067	-0.076445
H	3.564239	0.844167	0.024643	H	5.474308	-2.998475	-0.077919
H	3.267707	-4.106155	-0.106968	H	-3.034071	0.447267	-1.997040
H	-5.434939	-0.136615	-1.999762	H	-5.057755	-1.697436	1.978839
H	-2.664871	-1.081639	1.992382	H	2.272156	4.786288	0.090883
H	-7.258814	-1.524624	-1.006822	H	-7.109084	-2.147629	0.645038
H	-7.490111	-0.448319	0.369883				

6a (ground state) b3lyp/6-311+g(d,p) scrf=(cpcm,solvent=acetonitrile)

```
# opt freq b3lyp/6-311+g(d,p)
scrf=(cpcm,solvent=acetonitrile)
```

Charge = 0, Multiplicity = 1, Point group = C1

Electronic Energy = -1591.553763 Hartree

Number of imaginary frequencies = 0

Sum of electronic and zero-point Energies = -1591.057003 Hartree

Sum of electronic and thermal Energies = -1591.026077 Hartree

Sum of electronic and thermal Enthalpies = -1591.025133 Hartree

Sum of electronic and thermal Free Energies = -1591.122126 Hartree

	**Cartesian Coordinates**				**Cartesian Coordinates**		
	X	***Y***	***Z***		***X***	***Y***	***Z***
C	-0.745590	-2.997523	1.198149	C	0.498093	-2.360860	1.318328
C	0.699009	-1.009455	0.989834	C	-0.421613	-0.259900	0.511967
C	-1.675777	-0.938164	0.448131	C	-1.845904	-2.284429	0.766558
C	-0.689357	1.109777	0.058016	C	-2.077895	1.167312	-0.202038
N	-2.659945	-0.061483	0.020580	C	0.079632	2.309304	-0.180797
C	-0.624327	3.498850	-0.582509	C	-2.038327	3.480047	-0.774018
C	-2.762517	2.334976	-0.604931	C	1.489696	2.405143	-0.083575
C	2.162434	3.586327	-0.324129	C	1.463031	4.753947	-0.683783
C	0.093488	4.698846	-0.813431	C	-4.043696	-0.383146	-0.155655
C	-4.875102	-0.528234	0.955319	C	-6.218669	-0.849069	0.778315
C	-6.760817	-1.021006	-0.501622	C	-5.910465	-0.871549	-1.603732
C	-4.562786	-0.559570	-1.438072	O	1.518745	-3.146467	1.794708
C	-8.225381	-1.333704	-0.687105	C	2.080559	-0.486080	1.231645

C	3.145473	-0.701215	0.290143	C	4.463468	-0.235287	0.607355
C	4.686738	0.423239	1.845792	C	3.667612	0.614559	2.740026
C	2.364825	0.151577	2.430464	C	2.933170	-1.341787	-0.951251
C	3.973515	-1.529711	-1.844494	C	5.278523	-1.076418	-1.531160
C	5.502781	-0.443635	-0.329143	O	1.411043	0.380671	3.377837
O	3.657798	-2.161599	-3.011658	C	4.684282	-2.383350	-3.983153
H	-0.824933	-4.044360	1.464206	H	-2.817511	-2.754867	0.684186
H	-2.532142	4.397984	-1.073451	H	-3.832868	2.312800	-0.766225
H	2.058183	1.529646	0.175753	H	3.243310	3.609208	-0.239421
H	1.997655	5.678971	-0.866666	H	-0.464706	5.582013	-1.106824
H	-4.469149	-0.390863	1.950821	H	-6.855226	-0.963855	1.649258
H	-6.303472	-1.008216	-2.605572	H	-3.912401	-0.456949	-2.298978
H	2.324980	-2.614718	1.858670	H	-8.608598	-1.944602	0.133112
H	-8.817938	-0.412862	-0.713177	H	-8.402885	-1.864104	-1.624819
H	5.685901	0.775060	2.079212	H	3.830102	1.113833	3.687492
H	1.945616	-1.695437	-1.220325	H	6.097325	-1.219015	-2.222511
H	6.500613	-0.092739	-0.088157	H	0.564560	0.024109	3.069721
H	4.197463	-2.892120	-4.812661	H	5.108054	-1.438702	-4.334814
H	5.478362	-3.019528	-3.582663				

6b (ground state) b3lyp/6-311+g(d,p) scrf=(cpcm,solvent=acetonitrile)

```
# opt freq b3lyp/6-311+g(d,p)
scrf=(cpcm,solvent=acetonitrile)
```

Charge = 0, Multiplicity = 1, Point group = C1

Electronic Energy = -6624.081214 Hartree

Number of imaginary frequencies = 0

Sum of electronic and zero-point Energies = -6623.637324 Hartree

Sum of electronic and thermal Energies = -6623.605901 Hartree

Sum of electronic and thermal Enthalpies = -6623.604957 Hartree

Sum of electronic and thermal Free Energies = -6623.706485 Hartree

	Cartesian Coordinates				Cartesian Coordinates		
	X	***Y***	***Z***		***X***	***Y***	***Z***
C	-1.778878	-2.808722	1.799143	C	-0.482786	-2.282632	1.900390
C	-0.124795	-1.028316	1.375167	C	-1.135641	-0.264579	0.711341
C	-2.449169	-0.820994	0.676467	C	-2.775852	-2.074075	1.191260
C	-1.239065	1.031759	0.031686	C	-2.603445	1.180003	-0.314209
N	-3.316638	0.065514	0.060579	C	-0.342756	2.093563	-0.359572
C	-0.905856	3.261707	-0.984007	C	-2.305026	3.351045	-1.245063
C	-3.148775	2.324051	-0.935965	C	1.067553	2.057435	-0.212138
C	1.845274	3.116364	-0.623136	C	1.303071	4.276744	-1.198170

C	-0.060608	4.328189	-1.371972	C	-4.719422	-0.145670	-0.142266
C	-5.617468	0.079388	0.900755	C	-6.979082	-0.134325	0.699719
C	-7.469810	-0.567189	-0.538435	C	-6.551560	-0.789402	-1.572021
C	-5.187119	-0.584325	-1.380644	O	0.417380	-3.074440	2.568563
C	-8.949359	-0.763943	-0.760021	C	1.285204	-0.602175	1.642021
C	2.386265	-1.103727	0.870401	C	3.725851	-0.768813	1.245417
C	3.935645	0.063169	2.377009	C	2.881017	0.542964	3.105309
C	1.555228	0.203591	2.740663	C	2.185149	-1.922891	-0.273802
C	3.268204	-2.380142	-0.982486	C	4.595230	-2.068160	-0.621875
C	4.806535	-1.271076	0.478806	Br	2.978662	-3.485193	-2.531193
O	0.572016	0.709197	3.534234	Br	3.759767	3.017650	-0.429708
H	-1.979914	-3.784595	2.223407	H	-3.787140	-2.454413	1.122774
H	-2.687431	4.250335	-1.714484	H	-4.208458	2.375739	-1.151250
H	1.536967	1.187340	0.211819	H	1.939296	5.096095	-1.504853
H	-0.508995	5.203689	-1.829095	H	-5.249421	0.418237	1.862236
H	-7.669515	0.039773	1.518111	H	-6.905580	-1.132693	-2.538250
H	-4.484616	-0.766522	-2.185621	H	1.270500	-2.622050	2.622387
H	-9.454206	-1.056868	0.162865	H	-9.415260	0.164278	-1.107989
H	-9.139914	-1.528620	-1.515864	H	4.950718	0.318298	2.659887
H	3.033219	1.177932	3.969635	H	1.178648	-2.177541	-0.577422
H	5.425743	-2.447266	-1.202287	H	5.819304	-1.015914	0.770995
H	-0.290156	0.409258	3.209461				

7 (ground state) b3lyp/6-311+g(d,p) scrf=(cpcm,solvent=acetonitrile)

```
# opt freq b3lyp/6-311+g(d,p)
scrf=(cpcm,solvent=acetonitrile)
```

Charge = 0, Multiplicity = 1, Point group = C1

Electronic Energy = -1722.957748 Hartree

Number of imaginary frequencies = 0

Sum of electronic and zero-point Energies = -1722.445060 Hartree

Sum of electronic and thermal Energies = -1722.413806 Hartree

Sum of electronic and thermal Enthalpies = -1722.412862 Hartree

Sum of electronic and thermal Free Energies = -1722.509799 Hartree

	Cartesian Coordinates				Cartesian Coordinates		
	X	*Y*	*Z*		*X*	*Y*	*Z*
C	4.188625	-3.352626	1.754474	C	5.547732	-3.011709	1.646353
C	5.948491	-1.818325	1.058553	C	4.970575	-0.932792	0.569515
C	3.602925	-1.290607	0.698210	C	3.206116	-2.495431	1.279190
C	5.001655	0.351998	-0.098394	C	3.661657	0.708451	-0.342534
N	2.816958	-0.282699	0.137820	C	6.048758	1.241348	-0.501037

C	5.673441	2.472107	-1.137470	C	4.293960	2.780018	-1.344814
C	3.294717	1.925429	-0.958978	C	7.430496	0.985288	-0.310815
C	8.389700	1.886943	-0.724368	C	8.018370	3.094634	-1.350712
C	6.684356	3.375532	-1.550189	C	1.393754	-0.280673	0.067551
C	0.636313	-0.274041	1.241068	C	-0.753191	-0.289987	1.173735
C	-1.393754	-0.280673	-0.067551	C	-0.636313	-0.274043	-1.241068
C	0.753191	-0.289989	-1.173735	O	6.448963	-3.922993	2.148486
N	-2.816958	-0.282698	-0.137820	C	-4.188625	-3.352626	-1.754473
C	-5.547732	-3.011709	-1.646352	C	-5.948491	-1.818326	-1.058553
C	-4.970575	-0.932792	-0.569515	C	-3.602925	-1.290607	-0.698210
C	-3.206116	-2.495431	-1.279190	C	-5.001655	0.351998	0.098394
C	-3.661657	0.708451	0.342534	C	-6.048758	1.241348	0.501037
C	-5.673441	2.472108	1.137470	C	-4.293960	2.780018	1.344814
C	-3.294717	1.925430	0.958977	C	-7.430495	0.985288	0.310815
C	-8.389700	1.886943	0.724368	C	-8.018370	3.094634	1.350712
C	-6.684356	3.375532	1.550188	O	-6.448963	-3.922993	-2.148485
H	3.920319	-4.296276	2.213893	H	7.005118	-1.595653	0.985076
H	2.160423	-2.763777	1.360808	H	4.042705	3.721832	-1.820380
H	2.253724	2.176009	-1.118704	H	7.747397	0.068511	0.165419
H	9.438847	1.663449	-0.565913	H	8.779999	3.795860	-1.671709
H	6.386489	4.302028	-2.030270	H	1.133682	-0.261229	2.203080
H	-1.341107	-0.304640	2.083238	H	-1.133682	-0.261233	-2.203080
H	1.341107	-0.304645	-2.083238	H	7.348462	-3.596613	2.027977
H	-3.920320	-4.296277	-2.213892	H	-7.005118	-1.595653	-0.985075
H	-2.160424	-2.763777	-1.360808	H	-4.042705	3.721832	1.820379
H	-2.253724	2.176010	1.118703	H	-7.747397	0.068511	-0.165419
H	-9.438847	1.663449	0.565914	H	-8.779999	3.795860	1.671709
H	-6.386488	4.302028	2.030270	H	-7.348463	-3.596613	-2.027976

10a (ground state) b3lyp/6-311+g(d,p) scrf=(cpcm,solvent=acetonitrile)

```
# opt freq b3lyp/6-311+g(d,p)
scrf=(cpcm,solvent=acetonitrile)
```

Charge = 0, Multiplicity = 1, Point group = C1

Electronic Energy = -785.843493 Hartree

Number of imaginary frequencies = 0

Sum of electronic and zero-point Energies = -785.588906 Hartree

Sum of electronic and thermal Energies = -785.574543 Hartree

Sum of electronic and thermal Enthalpies = -785.573599 Hartree

Sum of electronic and thermal Free Energies = -785.630005 Hartree

Cartesian Coordinates	Cartesian Coordinates

	X	Y	Z		X	Y	Z
C	-3.635402	-0.926506	-0.000004	C	-3.153524	0.400230	-0.000033
C	-1.788902	0.668298	-0.000026	C	-0.881016	-0.411279	0.000018
C	-1.390750	-1.735877	0.000032	C	-2.762347	-2.001063	0.000025
C	0.564211	-0.527406	0.000019	C	0.851093	-1.907978	0.000022
N	-0.326398	-2.619108	0.000040	C	1.647303	0.409332	0.000015
C	2.984928	-0.114225	-0.000033	C	3.205492	-1.527277	-0.000048
C	2.167083	-2.420826	-0.000013	C	1.484507	1.817928	0.000072
C	2.573409	2.665957	0.000056	C	3.885435	2.149674	-0.000015
C	4.079837	0.785261	-0.000053	O	-4.128289	1.366973	-0.000083
C	-3.726883	2.735547	0.000011	H	-4.707036	-1.085951	-0.000007
H	-1.435734	1.687397	-0.000066	H	-3.137275	-3.017877	0.000042
H	-0.400128	-3.624287	0.000026	H	4.228386	-1.887824	-0.000085
H	2.343348	-3.490234	-0.000014	H	0.491006	2.243923	0.000144
H	2.417280	3.739019	0.000104	H	4.734094	2.823990	-0.000032
H	5.085446	0.377148	-0.000096	H	-4.647435	3.316297	0.000045
H	-3.143858	2.978756	0.893915	H	-3.143850	2.978880	-0.893855

10b (ground state) b3lyp/6-311+g(d,p) scrf=(cpcm,solvent=acetonitrile)

```
# opt freq b3lyp/6-311+g(d,p)
scrf=(cpcm,solvent=acetonitrile)
```

Charge = 0, Multiplicity = 1, Point group = C1

Electronic Energy = -825.160100 Hartree

Number of imaginary frequencies = 0

Sum of electronic and zero-point Energies = -824.877900 Hartree

Sum of electronic and thermal Energies = -824.862605 Hartree

Sum of electronic and thermal Enthalpies = -824.861661 Hartree

Sum of electronic and thermal Free Energies = -824.920233 Hartree

	Cartesian Coordinates				Cartesian Coordinates		
	X	Y	Z		X	Y	Z
C	3.625249	0.541575	0.000142	C	3.093219	-0.764830	0.000125
C	1.718670	-0.979592	-0.000010	C	0.853392	0.133376	-0.000161
C	1.411393	1.438327	-0.000284	C	2.793514	1.649325	-0.000020
C	-0.583003	0.307018	-0.000152	C	-0.814261	1.700918	-0.000184
N	0.390792	2.374407	-0.000711	C	-1.696567	-0.592072	-0.000112
C	-3.015986	-0.025814	0.000193	C	-3.185114	1.392814	0.000498
C	-2.116969	2.251173	0.000362	C	-1.581537	-2.005964	-0.000363
C	-2.698163	-2.816868	-0.000328	C	-3.992705	-2.257451	-0.000025
C	-4.141009	-0.887412	0.000237	C	0.597865	3.812979	0.000032
O	4.029220	-1.769235	0.000351	C	3.574443	-3.121026	0.000156

H	4.702155	0.660424	0.000365	H	1.327672	-1.984749	0.000082
H	3.212968	2.648020	0.000158	H	-4.194121	1.790696	0.000928
H	-2.274912	3.322255	0.000849	H	-0.603389	-2.465636	-0.000631
H	-2.577775	-3.894560	-0.000546	H	-4.863274	-2.903217	-0.000012
H	-5.132158	-0.445261	0.000469	H	-0.362644	4.321996	-0.011246
H	1.143393	4.123569	0.894953	H	1.162005	4.120649	-0.884173
H	4.471549	-3.737388	0.000192	H	2.982217	-3.341411	0.893930
H	2.982415	-3.341212	-0.893796				

10c (ground state) b3lyp/6-311+g(d,p) scrf=(cpcm,solvent=acetonitrile)

```
# opt freq b3lyp/6-311+g(d,p)
scrf=(cpcm,solvent=acetonitrile)
```

Charge = 0, Multiplicity = 1, Point group = C1

Electronic Energy = -1056.271556 Hartree

Number of imaginary frequencies = 0

Sum of electronic and zero-point Energies = -1055.909742 Hartree

Sum of electronic and thermal Energies = -1055.888765 Hartree

Sum of electronic and thermal Enthalpies = -1055.887820 Hartree

Sum of electronic and thermal Free Energies = -1055.961092 Hartree

	Cartesian Coordinates				Cartesian Coordinates		
	X	***Y***	***Z***		***X***	***Y***	***Z***
C	0.134956	3.540353	0.072605	C	1.521397	3.279806	0.062689
C	2.004477	1.975903	0.034970	C	1.085095	0.906821	0.011653
C	-0.303183	1.194262	0.013391	C	-0.784598	2.505420	0.049287
C	1.207048	-0.536079	-0.006174	C	-0.106256	-1.046934	-0.013945
N	-1.016656	-0.002179	-0.001353	C	2.312407	-1.446094	-0.026346
C	2.021911	-2.851928	-0.059055	C	0.666495	-3.304292	-0.075486
C	-0.388742	-2.430533	-0.054695	C	3.673922	-1.049100	-0.016897
C	4.692669	-1.979787	-0.037423	C	4.404403	-3.359787	-0.068657
C	3.092271	-3.780445	-0.079181	C	-2.440174	-0.124705	-0.001034
C	-3.180539	0.302142	-1.104848	C	-4.568201	0.185218	-1.097887
C	-5.245243	-0.366783	-0.003218	C	-4.485336	-0.798422	1.090400
C	-3.097887	-0.673167	1.100791	C	-6.750838	-0.469058	0.006845
O	2.318670	4.396521	0.085024	C	3.734208	4.220940	0.080221
H	-0.196641	4.571425	0.099432	H	3.067014	1.791217	0.033606
H	-1.847211	2.713603	0.058840	H	0.481085	-4.372492	-0.107057
H	-1.411076	-2.786742	-0.070510	H	3.927698	0.001079	0.007956
H	5.724180	-1.645470	-0.029081	H	5.211832	-4.082785	-0.084585
H	2.858345	-4.839887	-0.103857	H	-2.669714	0.716075	-1.966395
H	-5.131532	0.518560	-1.963123	H	-4.983664	-1.232816	1.950604

H	-2.524849	-0.999512	1.960851	H	-7.145378	-0.624524	-0.999659
H	-7.089353	-1.291245	0.640637	H	-7.200019	0.451371	0.395282
H	4.158818	5.222921	0.099525	H	4.068937	3.667356	0.963156
H	4.067206	3.702445	-0.824385				

12 (ground state) b3lyp/6-311+g(d,p) scrf=(cpcm,solvent=acetonitrile)

```
# opt freq b3lyp/6-311+g(d,p)
scrf=(cpcm,solvent=acetonitrile)
```

Charge = 0, Multiplicity = 1, Point group = C1

Electronic Energy = -5664.694080 Hartree

Number of imaginary frequencies = 0

Sum of electronic and zero-point Energies = -5664.538828 Hartree

Sum of electronic and thermal Energies = -5664.526788 Hartree

Sum of electronic and thermal Enthalpies = -5664.525844 Hartree

Sum of electronic and thermal Free Energies = -5664.579279 Hartree

	**Cartesian Coordinates**				**Cartesian Coordinates**		
	X	***Y***	***Z***		***X***	***Y***	***Z***
C	-1.128589	1.958701	0.000003	C	-0.723841	0.597809	0.000004
C	0.723841	0.597809	0.000001	C	1.128589	1.958701	-0.000002
N	-0.000000	2.756618	0.000000	C	-2.477203	2.319498	0.000003
C	-3.426292	1.303623	0.000002	C	-3.022015	-0.040015	0.000001
C	-1.688119	-0.416849	0.000003	C	1.688119	-0.416849	0.000003
C	3.022015	-0.040015	-0.000001	C	3.426292	1.303623	-0.000004
C	2.477203	2.319498	-0.000005	Br	-4.380197	-1.409048	-0.000002
Br	4.380197	-1.409049	0.000001	H	-0.000000	3.765097	-0.000003
H	-2.785039	3.358156	0.000002	H	-4.479541	1.552056	0.000001
H	-1.402125	-1.460834	0.000002	H	1.402125	-1.460834	0.000005
H	4.479541	1.552056	-0.000007	H	2.785039	3.358156	-0.000007

3a (ground state) b3lyp/6-311g

opt freq b3lyp/6-311g

Charge = 0, Multiplicity = 1, Point group = C1

Electronic Energy = −1016.665128 Hartree

Number of imaginary frequencies = 0

Sum of electronic and zero-point Energies = −1016.328808 Hartree

Sum of electronic and thermal Energies = −1016.309508 Hartree

Sum of electronic and thermal Enthalpies = −1016.308564 Hartree

Sum of electronic and thermal Free Energies = −1016.378351 Hartree

**Atoms**	**Cartesian Coordinates**	**Atoms**	**Cartesian Coordinates**

	X	Y	Z		X	Y	Z
C	−0.253897	3.757567	0.099196	C	1.149979	3.695547	0.087208
C	1.827865	2.481964	0.052646	C	1.087665	1.284226	0.022395
C	−0.333675	1.366537	0.024091	C	−1.007584	2.590195	0.069649
C	1.427895	−0.127240	−0.002098	C	0.208630	−0.837802	−0.015962
N	−0.860591	0.064212	0.001295	C	2.662955	−0.852212	−0.023718
C	2.596800	−2.290196	−0.067571	C	1.326800	−2.948038	−0.092878
C	0.145503	−2.248184	−0.069310	C	3.947289	−0.245156	−0.005692
C	5.101449	−1.003955	−0.027882	C	5.033730	−2.414160	−0.070247
C	3.803397	−3.037798	−0.089400	C	−2.250451	−0.274490	−0.001491
C	−3.061027	0.084840	−1.084657	C	−4.417315	−0.240707	−1.080277
C	−4.994058	−0.940465	−0.008673	C	−4.165470	−1.302970	1.064028
C	−2.811011	−0.967375	1.077108	O	1.816019	4.928524	0.116153
C	−6.469002	−1.271576	−0.001243	H	−0.724156	4.728963	0.134221
H	2.908609	2.476847	0.052002	H	−2.087040	2.632421	0.085408
H	1.311244	−4.029833	−0.135502	H	−0.807658	−2.756129	−0.096360
H	4.027325	0.829955	0.028126	H	6.066411	−0.514159	−0.012507
H	5.943856	−2.998598	−0.087526	H	3.740564	−4.118814	−0.122369
H	−2.623074	0.604479	−1.925528	H	−5.033045	0.041443	−1.925089
H	−4.585647	−1.844045	1.902441	H	−2.187128	−1.233593	1.919041
H	2.780756	4.814621	0.107053	H	−6.840266	−1.461827	−1.009359
H	−6.676697	−2.154335	0.604620	H	−7.056868	−0.446403	0.411704

3a (ground state) b3lyp/6-31+g(d,p) scrf=(cpcm,solvent=acetonitrile)

opt freq b3lyp/6-31+g(d,p) scrf=(cpcm,solvent=acetonitrile)

Charge = 0, Multiplicity = 1, Point group = C1

Electronic Energy = −1016.772935 Hartree

Number of imaginary frequencies = 0

Sum of electronic and zero-point Energies = −1016.438336 Hartree

Sum of electronic and thermal Energies = −1016.418729 Hartree

Sum of electronic and thermal Enthalpies = −1016.417785 Hartree

Sum of electronic and thermal Free Energies = −1016.488251 Hartree

Atoms	Cartesian Coordinates			Atoms	Cartesian Coordinates		
	X	Y	Z		X	Y	Z
C	−0.268568	3.753920	0.092031	C	1.139389	3.704654	0.083024
C	1.822899	2.491833	0.050707	C	1.086215	1.289959	0.021672
C	−0.334765	1.360722	0.021998	C	−1.016525	2.581125	0.062985
C	1.429154	−0.117935	−0.002271	C	0.207972	−0.825447	−0.015927
N	−0.854482	0.066482	−0.000466	C	2.664123	−0.846546	−0.022665

C	2.593963	−2.283011	−0.063351	C	1.322372	−2.940006	−0.086224
C	0.141251	−2.238416	−0.063906	C	3.949625	−0.241956	−0.005439
C	5.103258	−1.005928	−0.026414	C	5.031885	−2.416764	−0.066118
C	3.797026	−3.036551	−0.083894	C	−2.241893	−0.275009	−0.001886
C	−3.040261	0.036284	−1.108628	C	−4.396481	−0.294087	−1.103026
C	−4.982340	−0.948524	−0.007516	C	−4.163643	−1.261588	1.088389
C	−2.808709	−0.922651	1.101005	O	1.793650	4.918310	0.110049
C	−6.455891	−1.281987	−0.000138	H	−0.760056	4.720921	0.122415
H	2.906998	2.494330	0.050253	H	−2.100448	2.619567	0.071077
H	1.303757	−4.025705	−0.123522	H	−0.815281	−2.749127	−0.083865
H	4.037340	0.836608	0.026247	H	6.072006	−0.514682	−0.011830
H	5.942780	−3.007618	−0.082482	H	3.729332	−4.121223	−0.114595
H	−2.597811	0.527618	−1.969732	H	−5.005586	−0.048547	−1.969052
H	−4.590495	−1.771472	1.947990	H	−2.191218	−1.159756	1.961934
H	2.751381	4.780568	0.100451	H	−6.813477	−1.529129	−1.004296
H	−6.670015	−2.127554	0.659723	H	−7.047378	−0.429193	0.355703

3b (ground state) b3lyp/6-31+g(d,p) scrf=(cpcm,solvent=acetonitrile)

opt freq b3lyp/6-31+g(d,p) scrf=(cpcm,solvent=acetonitrile)

Charge = 0, Multiplicity = 1, Point group = C1

Electronic Energy = −1437.045453 Hartree

Number of imaginary frequencies = 0

Sum of electronic and zero-point Energies = −1436.747529 Hartree

Sum of electronic and thermal Energies = −1436.728661 Hartree

Sum of electronic and thermal Enthalpies = −1436.727717 Hartree

Sum of electronic and thermal Free Energies = −1436.795736 Hartree

Atoms	Cartesian Coordinates			Atoms	Cartesian Coordinates		
	X	*Y*	*Z*		*X*	*Y*	*Z*
C	−0.248587	3.807534	0.102104	C	−1.651062	3.677369	0.091843
C	−2.263447	2.427081	0.055967	C	−1.458693	1.270225	0.022511
C	−0.044887	1.422888	0.021431	C	0.565674	2.679904	0.068962
C	−1.721627	−0.155261	−0.003072	C	−0.464128	−0.793960	−0.018148
N	0.547562	0.158277	−0.003396	C	−2.914593	−0.950768	−0.025336
C	−2.765202	−2.380901	−0.069923	C	−1.459612	−2.966308	−0.096394
C	−0.318949	−2.200548	−0.072606	C	−4.231392	−0.417918	−0.006626
C	−5.340849	−1.244488	−0.029016	C	−5.191704	−2.649171	−0.071952
C	−3.924773	−3.199907	−0.091739	C	1.948252	−0.103503	−0.003237
C	2.536390	−0.778204	1.073426	C	3.907211	−1.041733	1.073699
C	4.681276	−0.608021	−0.003015	C	4.113289	0.074068	−1.079682

C	2.739042	0.318215	−1.079050	O	−2.373883	4.850537	0.122508
Cl	6.414267	−0.928411	−0.003127	H	0.186492	4.800971	0.137063
H	−3.345779	2.367149	0.056580	H	1.645279	2.782637	0.079961
H	−1.380548	−4.049052	−0.138370	H	0.663361	−2.659292	−0.097601
H	−4.378963	0.653944	0.027556	H	−6.335228	−0.807632	−0.013075
H	−6.068698	−3.289188	−0.089349	H	−3.797240	−4.279017	−0.125285
H	1.925519	−1.093112	1.912978	H	4.364712	−1.566187	1.904992
H	4.727944	0.400417	−1.911015	H	2.280994	0.831639	−1.917963
H	−3.322032	4.657922	0.109510				

3c (ground state) b3lyp/6-31+g(d,p) scrf=(cpcm,solvent=acetonitrile)

opt freq b3lyp/6-31+g(d,p) scrf=(cpcm,solvent=acetonitrile)

Charge = 0, Multiplicity = 1, Point group = C1

Electronic Energy = −1056.093617 Hartree

Number of imaginary frequencies = 0

Sum of electronic and zero-point Energies = −1055.731473 Hartree

Sum of electronic and thermal Energies = −1055.710159 Hartree

Sum of electronic and thermal Enthalpies = −1055.709215 Hartree

Sum of electronic and thermal Free Energies = −1055.783328 Hartree

Atoms	Cartesian Coordinates			Atoms	Cartesian Coordinates		
	X	*Y*	*Z*		*X*	*Y*	*Z*
C	0.452455	3.515158	0.065402	C	−0.965322	3.531824	0.058320
C	−1.723469	2.365592	0.033099	C	−1.070567	1.116881	0.008539
C	0.349405	1.104557	0.006259	C	1.103638	2.281770	0.040089
C	−1.497784	−0.267788	−0.007577	C	−0.321073	−1.047212	−0.019281
N	0.792646	−0.218614	−0.010710	C	−2.773474	−0.921163	−0.022206
C	−2.790448	−2.359662	−0.054980	C	−1.560980	−3.092047	−0.076322
C	−0.339682	−2.461459	−0.059961	C	−4.019729	−0.238885	−0.006398
C	−5.217824	−0.930849	−0.021250	C	−5.232365	−2.343918	−0.053208
C	−4.037755	−3.038088	−0.069559	C	2.156817	−0.642186	−0.007907
C	2.683638	−1.313871	1.101069	C	4.017425	−1.727216	1.095886
C	4.855280	−1.470380	−0.000700	C	4.312529	−0.784305	−1.098557
C	2.976314	−0.379270	−1.111312	O	−1.555626	4.779411	0.080382
C	6.290973	−1.941080	−0.009569	C	1.223540	4.810935	0.099671
H	−2.805405	2.442027	0.035126	H	2.188438	2.245495	0.046030
H	−1.607191	−4.177161	−0.107631	H	0.584508	−3.028818	−0.078758
H	−4.039958	0.843384	0.019152	H	−6.154923	−0.381558	−0.007957
H	−6.177647	−2.878237	−0.064873	H	−4.036840	−4.125029	−0.094389
H	2.052804	−1.504980	1.963794	H	4.414267	−2.247927	1.963428

H	4.939892	−0.568583	−1.959438	H	2.567699	0.139512	−1.973029
H	−2.518631	4.687391	0.072258	H	6.705648	−1.974793	1.002239
H	6.367703	−2.952348	−0.428247	H	6.921561	−1.286879	−0.618725
H	0.990402	5.438203	−0.768537	H	0.975115	5.401045	0.989355
H	2.299787	4.620235	0.104846				

3d (ground state) b3lyp/6-31+g(d,p) scrf=(cpcm,solvent=acetonitrile)

opt freq b3lyp/6-31+g(d,p) scrf=(cpcm,solvent=acetonitrile)

Charge = 0, Multiplicity = 1, Point group = C1

Electronic Energy = −3587.901683 Hartree

Number of imaginary frequencies = 0

Sum of electronic and zero-point Energies = −3587.576804 Hartree

Sum of electronic and thermal Energies = −3587.555753 Hartree

Sum of electronic and thermal Enthalpies = −3587.554809 Hartree

Sum of electronic and thermal Free Energies = −3587.630148 Hartree

Atoms	Cartesian Coordinates			Atoms	Cartesian Coordinates		
	X	*Y*	*Z*		*X*	*Y*	*Z*
C	−1.452039	3.759205	0.089209	C	−0.043290	3.729095	0.080664
C	0.656493	2.525628	0.049806	C	−0.064735	1.314668	0.021818
C	−1.486027	1.366096	0.022047	C	−2.184409	2.577019	0.061349
C	0.295046	−0.088510	−0.001247	C	−0.917133	−0.812687	−0.015013
N	−1.988831	0.064866	0.000079	C	1.535751	−0.803219	−0.021674
C	1.483698	−2.239720	−0.061549	C	0.222240	−2.913831	−0.083847
C	−0.966684	−2.226289	−0.061974	C	2.808732	−0.174025	−0.004307
C	3.956935	−0.937162	−0.025638	C	3.925836	−2.344969	−0.064468
C	2.696403	−2.973741	−0.081415	C	−3.372405	−0.294558	−0.001831
C	−4.169366	−0.009254	−1.116281	C	−5.521311	−0.356537	−1.110902
C	−6.103230	−1.001530	−0.007684	C	−5.285385	−1.287944	1.096125
C	−3.934770	−0.932321	1.108791	Br	5.661054	−0.061633	−0.000776
O	0.593680	4.951063	0.106622	C	−7.572484	−1.353190	−0.000917
H	−1.956122	4.719696	0.118343	H	1.740404	2.543885	0.049581
H	−3.268716	2.600719	0.068977	H	0.217870	−3.999389	−0.120339
H	−1.917115	−2.748010	−0.081680	H	2.886460	0.903250	0.026962
H	4.845217	−2.918725	−0.080349	H	2.650717	−4.059040	−0.111329
H	−3.729373	0.475196	−1.982519	H	−6.130031	−0.131546	−1.982721
H	−5.709489	−1.790060	1.961589	H	−3.317520	−1.148820	1.975308
H	1.553401	4.827589	0.097416	H	−7.922736	−1.620661	−1.002423
H	−7.778873	−2.190690	0.671548	H	−8.175787	−0.502154	0.338886

3e (ground state) b3lyp/6-31+g(d,p) scrf=(cpcm,solvent=acetonitrile)

opt freq b3lyp/6-31+g(d,p) scrf=(cpcm,solvent=acetonitrile)

Charge = 0, Multiplicity = 1, Point group = C1

Electronic Energy = −1109.023567 Hartree

Number of imaginary frequencies = 0

Sum of electronic and zero-point Energies = −1108.690420 Hartree

Sum of electronic and thermal Energies = −1108.668943 Hartree

Sum of electronic and thermal Enthalpies = −1108.667999 Hartree

Sum of electronic and thermal Free Energies = −1108.743161 Hartree

Atoms	Cartesian Coordinates			Atoms	Cartesian Coordinates		
	X	*Y*	*Z*		*X*	*Y*	*Z*
C	−0.742011	3.754623	0.086407	C	0.667218	3.712030	0.077039
C	1.355941	2.502748	0.046369	C	0.623100	1.298282	0.019986
C	−0.797689	1.362581	0.021231	C	−1.485313	2.580216	0.059779
C	0.968664	−0.107704	−0.003330	C	−0.251015	−0.819776	−0.016675
N	−1.313521	0.067104	−0.000759	C	2.200932	−0.838391	−0.022129
C	2.135172	−2.276478	−0.061014	C	0.867213	−2.935589	−0.083343
C	−0.313183	−2.232254	−0.062487	C	3.477547	−0.229289	−0.004591
C	4.637426	−0.999191	−0.024298	C	4.573086	−2.418186	−0.062701
C	3.338796	−3.028892	−0.080056	C	−2.701484	−0.278448	−0.001810
C	−3.490946	−0.002679	−1.123582	C	−4.846385	−0.336515	−1.117482
C	−5.438429	−0.957406	−0.005993	C	−4.627458	−1.234199	1.105527
C	−3.273383	−0.892546	1.117212	O	1.313317	4.928254	0.101740
C	−6.911234	−1.293640	0.002186	C	5.914408	−0.348855	−0.005116
N	6.956526	0.172616	0.010123	H	−1.236852	4.719887	0.115143
H	2.439925	2.511716	0.044712	H	−2.569302	2.613533	0.067795
H	0.849303	−4.020737	−0.118864	H	−1.269199	−2.743912	−0.081776
H	3.569107	0.847606	0.026033	H	5.486235	−3.002494	−0.077903
H	3.274525	−4.112837	−0.109403	H	−3.042938	0.463549	−1.995680
H	−5.449832	−0.119652	−1.994931	H	−5.059693	−1.717920	1.977398
H	−2.661014	−1.101326	1.989082	H	2.272216	4.798262	0.092198
H	−7.269117	−1.540464	−1.001774	H	−7.123376	−2.139870	0.661919
H	−7.503405	−0.442076	0.359818				

6a (ground state) b3lyp/6-31+g(d,p) scrf=(cpcm,solvent=acetonitrile)

opt freq b3lyp/6-31+g(d,p) scrf=(cpcm,solvent=acetonitrile)

Charge = 0, Multiplicity = 1, Point group = C1

Electronic Energy = −1591.245497 Hartree

Number of imaginary frequencies = 0

Sum of electronic and zero-point Energies = −1590.747024 Hartree
Sum of electronic and thermal Energies = −1590.716163 Hartree
Sum of electronic and thermal Enthalpies = −1590.715219 Hartree
Sum of electronic and thermal Free Energies = −1590.811692 Hartree

Atoms	Cartesian Coordinates			Atoms	Cartesian Coordinates		
	X	***Y***	***Z***		***X***	***Y***	***Z***
C	−0.738678	−3.013292	1.159271	C	0.503571	−2.368775	1.286117
C	0.699678	−1.011189	0.966831	C	−0.427109	−0.263650	0.492300
C	−1.681264	−0.946850	0.428020	C	−1.846061	−2.299598	0.735044
C	−0.700990	1.108320	0.046487	C	−2.093734	1.164599	−0.203883
N	−2.671979	−0.069320	0.012189	C	0.066592	2.310617	−0.194995
C	−0.643686	3.504996	−0.581096	C	−2.062187	3.486723	−0.757380
C	−2.785744	2.336174	−0.591442	C	1.480829	2.403751	−0.119346
C	2.153000	3.588927	−0.363232	C	1.447824	4.762428	−0.703938
C	0.072677	4.708494	−0.814288	C	−4.054990	−0.393872	−0.160332
C	−4.872524	−0.596284	0.956949	C	−6.218904	−0.920325	0.782357
C	−6.778860	−1.037372	−0.500275	C	−5.942642	−0.829885	−1.607942
C	−4.591271	−0.515515	−1.445803	O	1.527528	−3.154010	1.761222
C	−8.245408	−1.353324	−0.678935	C	2.074014	−0.475453	1.225897
C	3.158340	−0.689353	0.302320	C	4.469886	−0.208314	0.636862
C	4.668498	0.464377	1.874748	C	3.630693	0.655719	2.753533
C	2.335782	0.175608	2.426876	C	2.971666	−1.345474	−0.938417
C	4.031379	−1.535248	−1.813576	C	5.330341	−1.068014	−1.483546
C	5.528739	−0.418969	−0.281400	O	1.365048	0.404543	3.360229
O	3.738804	−2.183412	−2.980691	C	4.784723	−2.411371	−3.930895
H	−0.812883	−4.064399	1.417139	H	−2.817210	−2.774612	0.650539
H	−2.561051	4.408017	−1.044794	H	−3.859176	2.314290	−0.743739
H	2.052873	1.523612	0.123419	H	3.237061	3.608859	−0.295723
H	1.980900	5.690208	−0.888877	H	−0.490137	5.595303	−1.094838
H	−4.453626	−0.500432	1.954015	H	−6.844042	−1.080348	1.656853
H	−6.349262	−0.922336	−2.611438	H	−3.952207	−0.368736	−2.311171
H	2.336297	−2.621045	1.828452	H	−8.596551	−2.058654	0.080473
H	−8.853934	−0.445270	−0.585625	H	−8.443531	−1.781947	−1.665344
H	5.662360	0.828100	2.120956	H	3.774324	1.165742	3.700357
H	1.989605	−1.709445	−1.220599	H	6.163635	−1.211361	−2.159970
H	6.521038	−0.056409	−0.026617	H	0.522278	0.037363	3.045195
H	4.314848	−2.934571	−4.763623	H	5.209821	−1.466876	−4.287139
H	5.576315	−3.038402	−3.506633				

6b (ground state) b3lyp/6-31+g(d,p) scrf=(cpcm,solvent=acetonitrile)

opt freq b3lyp/6-31+g(d,p) scrf=(cpcm,solvent=acetonitrile)

Charge = 0, Multiplicity = 1, Point group = C1

Electronic Energy = −6618.975346 Hartree

Number of imaginary frequencies = 0

Sum of electronic and zero-point Energies = −6618.528718 Hartree

Sum of electronic and thermal Energies = −6618.497694 Hartree

Sum of electronic and thermal Enthalpies = −6618.496750 Hartree

Sum of electronic and thermal Free Energies = −6618.595389 Hartree

Atoms	Cartesian Coordinates			Atoms	Cartesian Coordinates		
	X	*Y*	*Z*		*X*	*Y*	*Z*
C	−1.764358	−2.961542	1.552684	C	−0.483073	−2.406931	1.719267
C	−0.151390	−1.109243	1.285651	C	−1.173566	−0.336597	0.646507
C	−2.479613	−0.909180	0.569246	C	−2.779023	−2.207677	0.988506
C	−1.290249	0.995142	0.048076	C	−2.659563	1.161472	−0.272772
N	−3.361579	0.011569	0.020450	C	−0.392568	2.070502	−0.298716
C	−0.960038	3.294384	−0.803585	C	−2.368524	3.412573	−1.013365
C	−3.212996	2.358922	−0.784439	C	1.022065	1.989416	−0.231602
C	1.807895	3.067088	−0.580966	C	1.264681	4.287381	−1.022346
C	−0.107866	4.378789	−1.132848	C	−4.760390	−0.200022	−0.197662
C	−5.641936	−0.232650	0.887196	C	−7.004000	−0.449072	0.667696
C	−7.513014	−0.624473	−0.628614	C	−6.611208	−0.587603	−1.704207
C	−5.246107	−0.382057	−1.496684	O	0.425935	−3.213889	2.360255
C	−8.992304	−0.821037	−0.863703	C	1.217870	−0.618516	1.638631
C	2.387859	−1.029082	0.911619	C	3.682150	−0.581970	1.336140
C	3.780307	0.261260	2.476776	C	2.660565	0.648732	3.169126
C	1.380638	0.200023	2.753123	C	2.297203	−1.856009	−0.242519
C	3.442295	−2.218799	−0.913177	C	4.726807	−1.800180	−0.501351
C	4.830374	−0.990944	0.609923	Br	3.301413	−3.345952	−2.451262
O	0.329513	0.625840	3.509851	Br	3.715107	2.886219	−0.508667
H	−1.945600	−3.971893	1.903475	H	−3.780794	−2.609543	0.884969
H	−2.760014	4.352992	−1.390123	H	−4.277640	2.433904	−0.975846
H	1.491696	1.071388	0.077271	H	1.905096	5.121559	−1.284333
H	−0.556367	5.299521	−1.495886	H	−5.262026	−0.090048	1.894197
H	−7.680372	−0.477610	1.517737	H	−6.978564	−0.729244	−2.717132
H	−4.556914	−0.367805	−2.335488	H	1.264835	−2.739361	2.471593
H	−9.479564	−1.264219	0.009421	H	−9.485511	0.138343	−1.063710
H	−9.176185	−1.467613	−1.726910	H	4.760806	0.599873	2.798544
H	2.727638	1.292493	4.039945	H	1.326381	−2.192148	−0.586649
H	5.608538	−2.109522	−1.050405	H	5.808098	−0.652504	0.941152

H	−0.497802	0.262883	3.152029

7 (ground state) b3lyp/6-31+g(d,p) scrf=(cpcm,solvent=acetonitrile)

opt freq b3lyp/6-31+g(d,p) scrf=(cpcm,solvent=acetonitrile)

Charge = 0, Multiplicity = 1, Point group = C1

Electronic Energy = −1722.631314 Hartree

Number of imaginary frequencies = 0

Sum of electronic and zero-point Energies = −1722.116908 Hartree

Sum of electronic and thermal Energies = −1722.085729 Hartree

Sum of electronic and thermal Enthalpies = −1722.084785 Hartree

Sum of electronic and thermal Free Energies = −1722.181470 Hartree

Atoms	Cartesian Coordinates			Atoms	Cartesian Coordinates		
	X	Y	Z		X	Y	Z
C	4.186476	−3.338050	1.800193	C	5.548982	−2.998599	1.689811
C	5.953982	−1.809774	1.088044	C	4.975755	−0.927489	0.586131
C	3.605045	−1.282435	0.717659	C	3.203681	−2.482634	1.312422
C	5.009095	0.350591	−0.097395	C	3.667248	0.706689	−0.346354
N	2.819754	−0.278770	0.145298	C	6.059722	1.234166	−0.511973
C	5.685448	2.460613	−1.163900	C	4.304113	2.769491	−1.374433
C	3.300469	1.919035	−0.977721	C	7.443390	0.976219	−0.319833
C	8.407024	1.873022	−0.746079	C	8.037046	3.076842	−1.387316
C	6.699632	3.358654	−1.589046	C	1.396908	−0.276897	0.071364
C	0.634597	−0.269823	1.246125	C	−0.758120	−0.286677	1.174990
C	−1.396908	−0.276897	−0.071365	C	−0.634597	−0.269826	−1.246125
C	0.758120	−0.286680	−1.174990	O	6.448911	−3.906594	2.204895
N	−2.819754	−0.278770	−0.145299	C	−4.186476	−3.338050	−1.800193
C	−5.548982	−2.998599	−1.689811	C	−5.953982	−1.809774	−1.088044
C	−4.975755	−0.927489	−0.586131	C	−3.605045	−1.282435	−0.717659
C	−3.203681	−2.482634	−1.312422	C	−5.009095	0.350591	0.097395
C	−3.667248	0.706689	0.346353	C	−6.059722	1.234166	0.511973
C	−5.685447	2.460614	1.163900	C	−4.304113	2.769492	1.374432
C	−3.300469	1.919035	0.977720	C	−7.443389	0.976219	0.319833
C	−8.407024	1.873022	0.746079	C	−8.037045	3.076842	1.387316
C	−6.699632	3.358655	1.589045	O	−6.448912	−3.906595	−2.204895
H	3.915458	−4.277641	2.270468	H	7.012503	−1.587976	1.013672
H	2.155596	−2.748517	1.395626	H	4.053708	3.707820	−1.861540
H	2.258186	2.170011	−1.140597	H	7.758620	0.062307	0.167033
H	9.457466	1.647649	−0.585740	H	8.800810	3.774187	−1.718120
H	6.402487	4.281641	−2.080618	H	1.130513	−0.256667	2.211008

H	−1.349602	−0.301492	2.084425	H	−1.130513	−0.256672	−2.211009
H	1.349602	−0.301497	−2.084425	H	7.353225	−3.584823	2.082466
H	−3.915458	−4.277641	−2.270467	H	−7.012503	−1.587976	−1.013671
H	−2.155597	−2.748517	−1.395626	H	−4.053708	3.707821	1.861539
H	−2.258186	2.170012	1.140596	H	−7.758620	0.062307	−0.167033
H	−9.457466	1.647649	0.585741	H	−8.800810	3.774187	1.718120
H	−6.402487	4.281641	2.080618	H	−7.353226	−3.584823	−2.082465

10a (ground state) b3lyp/6-31+g(d,p) scrf=(cpcm,solvent=acetonitrile)

opt freq b3lyp/6-31+g(d,p) scrf=(cpcm,solvent=acetonitrile)

Charge = 0, Multiplicity = 1, Point group = C1

Electronic Energy = −785.693936 Hartree

Number of imaginary frequencies = 0

Sum of electronic and zero-point Energies = −785.438356 Hartree

Sum of electronic and thermal Energies = −785.424035 Hartree

Sum of electronic and thermal Enthalpies = −785.423091 Hartree

Sum of electronic and thermal Free Energies = −785.479380 Hartree

Atoms	Cartesian Coordinates			Atoms	Cartesian Coordinates		
	X	*Y*	*Z*		*X*	*Y*	*Z*
C	−3.642509	−0.927496	−0.000003	C	−3.158697	0.401626	−0.000032
C	−1.790944	0.671276	−0.000024	C	−0.881719	−0.410695	0.000018
C	−1.392772	−1.737757	0.000031	C	−2.767067	−2.004748	0.000024
C	0.564785	−0.527749	0.000020	C	0.851180	−1.911156	0.000022
N	−0.327996	−2.622442	0.000039	C	1.650040	0.410133	0.000016
C	2.990035	−0.115536	−0.000033	C	3.210336	−1.531105	−0.000048
C	2.168730	−2.426834	−0.000013	C	1.487203	1.821260	0.000072
C	2.579364	2.671233	0.000056	C	3.893984	2.153201	−0.000015
C	4.087613	0.784842	−0.000053	O	−4.135437	1.369702	−0.000081
C	−3.733432	2.739037	0.000008	H	−4.715939	−1.087356	−0.000006
H	−1.436635	1.691874	−0.000065	H	−3.142270	−3.023468	0.000041
H	−0.402518	−3.628710	0.000024	H	4.234756	−1.893074	−0.000084
H	2.344762	−3.498153	−0.000015	H	0.491908	2.247714	0.000146
H	2.422656	3.746156	0.000104	H	4.744940	2.827758	−0.000033
H	5.094710	0.375171	−0.000096	H	−4.655662	3.320579	0.000040
H	−3.149015	2.981832	0.895385	H	−3.149008	2.981949	−0.895333

10b (ground state) b3lyp/6-31+g(d,p) scrf=(cpcm,solvent=acetonitrile)

opt freq b3lyp/6-31+g(d,p) scrf=(cpcm,solvent=acetonitrile)

Charge = 0, Multiplicity = 1, Point group = C1

Electronic Energy = −825.003972 Hartree
Number of imaginary frequencies = 0
Sum of electronic and zero-point Energies = −824.720681 Hartree
Sum of electronic and thermal Energies = −824.705426 Hartree
Sum of electronic and thermal Enthalpies = −824.704481 Hartree
Sum of electronic and thermal Free Energies = −824.762943 Hartree

Atoms	Cartesian Coordinates			Atoms	Cartesian Coordinates		
	X	*Y*	*Z*		*X*	*Y*	*Z*
C	3.632217	0.541334	0.000157	C	3.097861	−0.767287	0.000128
C	1.720069	−0.983209	−0.000029	C	0.853784	0.132364	−0.000186
C	1.413456	1.439429	−0.000305	C	2.798379	1.651933	−0.000009
C	−0.583896	0.307513	−0.000166	C	−0.814052	1.704192	−0.000187
N	0.392998	2.377511	−0.000720	C	−1.699835	−0.592510	−0.000124
C	−3.021411	−0.023847	0.000203	C	−3.189822	1.397330	0.000549
C	−2.118261	2.257481	0.000420	C	−1.585027	−2.008933	−0.000399
C	−2.705134	−2.821455	−0.000365	C	−4.002096	−2.259972	−0.000032
C	−4.149288	−0.885996	0.000255	C	0.602995	3.815641	−0.000019
O	4.035499	−1.773310	0.000366	C	3.579796	−3.125743	0.000192
H	4.710949	0.660332	0.000401	H	1.327643	−1.989712	0.000063
H	3.218172	2.652484	0.000186	H	−4.200190	1.796964	0.001018
H	−2.276055	3.330436	0.000953	H	−0.605191	−2.469305	−0.000690
H	−2.584448	−3.901049	−0.000605	H	−4.875079	−2.905766	−0.000017
H	−5.141784	−0.442056	0.000510	H	−0.357838	4.327534	−0.011196
H	1.150573	4.125706	0.896142	H	1.168949	4.122702	−0.885612
H	4.478408	−3.743164	0.000245	H	2.986139	−3.345475	0.895443
H	2.986350	−3.345310	−0.895239				

10c (ground state) b3lyp/6-31+g(d,p) scrf=(cpcm,solvent=acetonitrile)

# opt freq b3lyp/6-31+g(d,p) scrf=(cpcm,solvent=acetonitrile)
Charge = 0, Multiplicity = 1, Point group = C1
Electronic Energy = −1056.074375 Hartree
Number of imaginary frequencies = 0
Sum of electronic and zero-point Energies = −1055.711281 Hartree
Sum of electronic and thermal Energies = −1055.690319 Hartree
Sum of electronic and thermal Enthalpies = −1055.689374 Hartree
Sum of electronic and thermal Free Energies = −1055.762751 Hartree

Atoms	Cartesian Coordinates			Atoms	Cartesian Coordinates		
	X	*Y*	*Z*		*X*	*Y*	*Z*
C	0.129902	3.546330	0.073984	C	1.519411	3.286321	0.063867

C	2.006386	1.980283	0.034957	C	1.086469	0.908204	0.010958
C	−0.304746	1.194460	0.012608	C	−0.790307	2.507259	0.049887
C	1.209826	−0.535986	−0.008034	C	−0.105488	−1.048587	−0.017094
N	−1.018044	−0.003598	−0.004055	C	2.317952	−1.446433	−0.027223
C	2.027720	−2.855383	−0.060706	C	0.670366	−3.309706	−0.078887
C	−0.388666	−2.434598	−0.058844	C	3.681356	−1.047100	−0.015564
C	4.703868	−1.979289	−0.034956	C	4.416253	−3.362490	−0.067175
C	3.100692	−3.784877	−0.079630	C	−2.441361	−0.127244	−0.002318
C	−3.185477	0.308284	−1.104930	C	−4.576451	0.190605	−1.096010
C	−5.253159	−0.370841	−0.001305	C	−4.489241	−0.811159	1.090494
C	−3.098341	−0.684870	1.099866	C	−6.760487	−0.473417	0.010270
O	2.316072	4.406225	0.086804	C	3.732721	4.232099	0.081767
H	−0.204015	4.578547	0.101675	H	3.070807	1.796185	0.033275
H	−1.855124	2.713712	0.059250	H	0.485261	−4.379880	−0.111043
H	−1.412435	−2.792137	−0.075590	H	3.933921	0.005234	0.010179
H	5.736631	−1.642653	−0.024895	H	5.225298	−4.086625	−0.082232
H	2.866944	−4.846352	−0.104894	H	−2.675204	0.729472	−1.965684
H	−5.142697	0.531141	−1.958957	H	−4.986959	−1.252879	1.949726
H	−2.522509	−1.017711	1.957952	H	−7.156120	−0.643951	−0.995509
H	−7.099818	−1.287942	0.656608	H	−7.211987	0.453518	0.385393
H	4.156449	5.236418	0.102299	H	4.067953	3.676408	0.965477
H	4.066148	3.713680	−0.824964				

12 (ground state) b3lyp/6-31+g(d,p) scrf=(cpcm,solvent=acetonitrile)

opt freq b3lyp/6-31+g(d,p) scrf=(cpcm,solvent=acetonitrile)

Charge = 0, Multiplicity = 1, Point group = C1

Electronic Energy = −5659.769501 Hartree

Number of imaginary frequencies = 0

Sum of electronic and zero-point Energies = −5659.611085 Hartree

Sum of electronic and thermal Energies = −5659.599698 Hartree

Sum of electronic and thermal Enthalpies = −5659.598754 Hartree

Sum of electronic and thermal Free Energies = −5659.650840 Hartree

**Atoms**	**Cartesian Coordinates**			**Atoms**	**Cartesian Coordinates**		
	X	***Y***	***Z***		***X***	***Y***	***Z***
C	−1.129054	1.956998	−0.000005	C	−0.724350	0.593513	0.000009
C	0.724350	0.593513	0.000010	C	1.129054	1.956998	−0.000003
N	−0.000000	2.755829	−0.000020	C	−2.479664	2.320387	−0.000011
C	−3.430791	1.302449	0.000004	C	−3.025904	−0.043792	0.000042
C	−1.689514	−0.423053	0.000040	C	1.689514	−0.423053	0.000041

C	3.025904	−0.043792	0.000045	C	3.430791	1.302449	0.000010
C	2.479664	2.320387	−0.000005	Br	−4.378724	−1.406019	−0.000012
Br	4.378724	−1.406020	−0.000014	H	−0.000000	3.765504	−0.000034
H	−2.786585	3.361282	−0.000034	H	−4.485928	1.551350	−0.000017
H	−1.403974	−1.469118	0.000049	H	1.403974	−1.469118	0.000047
H	4.485928	1.551350	−0.000009	H	2.786585	3.361283	−0.000025

3a* (excited state) b3lyp/6-311+g(d,p) scrf=(cpcm,solvent=acetonitrile)

```
# p opt freq td=(nstates=2)/b3lyp/6-311+g(d,p)
scrf=(cpcm,solvent=acetonitrile)
```

Charge = 0, Multiplicity = 1, Point group = C1

Electronic Energy = -1016.956482 Hartree

Number of imaginary frequencies = 0

Sum of electronic and zero-point Energies = -1016.516478 Hartree

Sum of electronic and thermal Energies = -1016.496575 Hartree

Sum of electronic and thermal Enthalpies = -1016.495631 Hartree

Sum of electronic and thermal Free Energies = -1016.565805 Hartree

	Cartesian Coordinates				Cartesian Coordinates		
	X	*Y*	*Z*		*X*	*Y*	*Z*
C	-0.340735	3.750819	0.135810	C	1.064323	3.697344	0.096295
C	1.779980	2.472835	0.054077	C	1.070384	1.278430	0.037499
C	-0.371884	1.351216	0.052416	C	-1.069957	2.565644	0.120786
C	1.426142	-0.120666	0.021445	C	0.220220	-0.857285	0.024435
N	-0.865435	0.074386	0.045202	C	2.683094	-0.813532	-0.024160
C	2.624943	-2.269723	-0.074623	C	1.387552	-2.935236	-0.083587
C	0.153093	-2.236439	-0.039386	C	3.942482	-0.182868	-0.025782
C	5.131841	-0.917918	-0.070749	C	5.080564	-2.322780	-0.116676
C	3.866945	-2.984344	-0.118855	C	-2.237998	-0.281884	0.014132
C	-3.080801	0.252962	-0.966409	C	-4.425333	-0.100340	-0.987929
C	-4.961330	-0.983645	-0.042433	C	-4.098807	-1.512760	0.928840
C	-2.752299	-1.174561	0.962142	O	1.717733	4.877262	0.112399
C	-6.425181	-1.340791	-0.052326	H	-0.831256	4.713609	0.185802
H	2.860386	2.505978	0.043754	H	-2.150131	2.586037	0.168037
H	1.379373	-4.018625	-0.126525	H	-0.787633	-2.767640	-0.062434
H	4.003915	0.895571	0.012371	H	6.086468	-0.404848	-0.068688
H	6.003361	-2.893297	-0.150908	H	3.836122	-4.068475	-0.155677
H	-2.677839	0.916612	-1.721220	H	-5.065341	0.308973	-1.761792
H	-4.488789	-2.196645	1.674874	H	-2.104417	-1.580210	1.728577
H	2.674950	4.742061	0.089808	H	-6.865362	-1.188741	-1.039473
H	-6.582202	-2.380891	0.241807	H	-6.978841	-0.715134	0.656298

3b* (excited state) b3lyp/6-311+g(d,p) scrf=(cpcm,solvent=acetonitrile)

```
# p opt freq td=(nstates=2)/b3lyp/6-311+g(d,p)
scrf=(cpcm,solvent=acetonitrile)
```

Charge = 0, Multiplicity = 1, Point group = C1

Electronic Energy = -1437.250762 Hartree

Number of imaginary frequencies = 0

Sum of electronic and zero-point Energies = -1436.846186 Hartree

Sum of electronic and thermal Energies = -1436.826993 Hartree

Sum of electronic and thermal Enthalpies = -1436.826049 Hartree

Sum of electronic and thermal Free Energies = -1436.894346 Hartree

	Cartesian Coordinates				Cartesian Coordinates		
	X	*Y*	*Z*		*X*	*Y*	*Z*
C	-0.182489	3.811116	0.132414	C	-1.584769	3.673931	0.093176
C	-2.227579	2.410339	0.050763	C	-1.448992	1.259823	0.035446
C	-0.010789	1.419103	0.050405	C	0.615322	2.673906	0.116469
C	-1.720337	-0.156044	0.017750	C	-0.472837	-0.819785	0.017418
N	0.556489	0.176124	0.041171	C	-2.934043	-0.923923	-0.024710
C	-2.788185	-2.373733	-0.077455	C	-1.513216	-2.963256	-0.090224
C	-0.322456	-2.190908	-0.047234	C	-4.228880	-0.371276	-0.020916
C	-5.371410	-1.177751	-0.064550	C	-5.235883	-2.576676	-0.113521
C	-3.984376	-3.163017	-0.119934	C	1.948295	-0.099643	0.018231
C	2.508379	-0.935768	0.989896	C	3.872110	-1.202483	0.974295
C	4.665184	-0.633554	-0.019776	C	4.120990	0.195576	-0.996456
C	2.756329	0.461336	-0.976243	O	-2.301451	4.814062	0.108799
Cl	6.389485	-0.973547	-0.042806	H	0.248151	4.802125	0.181468
H	-3.307935	2.379095	0.037582	H	1.692553	2.758434	0.160761
H	-1.439230	-4.043995	-0.135286	H	0.648392	-2.665281	-0.071631
H	-4.356471	0.701297	0.020053	H	-6.355322	-0.723331	-0.059202
H	-6.122562	-3.201779	-0.146814	H	-3.887202	-4.243118	-0.158879
H	1.884323	-1.358649	1.766300	H	4.312606	-1.838030	1.731145
H	4.748234	0.620127	-1.769154	H	2.317561	1.085479	-1.744120
H	-3.250385	4.628678	0.085435				

3c* (excited state) b3lyp/6-311+g(d,p) scrf=(cpcm,solvent=acetonitrile)

```
# p opt freq td=(nstates=2)/b3lyp/6-311+g(d,p)
scrf=(cpcm,solvent=acetonitrile)
```

Charge = 0, Multiplicity = 1, Point group = C1

Electronic Energy = -1056.283225 Hartree

Number of imaginary frequencies = 0

Sum of electronic and zero-point Energies = -1055.816534 Hartree

Sum of electronic and thermal Energies = -1055.794853 Hartree

Sum of electronic and thermal Enthalpies = -1055.793909 Hartree

Sum of electronic and thermal Free Energies = -1055.868022 Hartree

	**Cartesian Coordinates**				**Cartesian Coordinates**		
	X	***Y***	***Z***		***X***	***Y***	***Z***
C	0.529510	3.505720	0.093961	C	-0.889612	3.521690	0.056643
C	-1.683174	2.347982	0.027954	C	-1.058594	1.109621	0.021389
C	0.387209	1.093836	0.035547	C	1.159861	2.265073	0.087503
C	-1.499806	-0.265121	0.015181	C	-0.341659	-1.071001	0.023036
N	0.802034	-0.205775	0.038432	C	-2.798408	-0.878404	-0.023541
C	-2.829322	-2.335635	-0.059441	C	-1.635337	-3.073123	-0.061221
C	-0.357173	-2.451426	-0.024957	C	-4.015069	-0.171173	-0.031076
C	-5.249792	-0.833538	-0.068614	C	-5.285317	-2.238265	-0.100838
C	-4.114691	-2.974332	-0.096610	C	2.151719	-0.649788	0.010457
C	2.619841	-1.526535	0.994759	C	3.943129	-1.949696	0.966705
C	4.825153	-1.522274	-0.035978	C	4.336488	-0.646023	-1.012610
C	3.014978	-0.211556	-0.998134	O	-1.471724	4.737528	0.060882
C	6.249192	-2.015084	-0.074598	C	1.302167	4.794580	0.146117
H	-2.759258	2.455187	0.018873	H	2.239768	2.208475	0.128436
H	-1.692706	-4.155709	-0.092477	H	0.549538	-3.039260	-0.039836
H	-4.009160	0.909471	-0.003977	H	-6.170925	-0.262337	-0.071586
H	-6.241770	-2.750963	-0.129504	H	-4.150489	-4.058646	-0.122892
H	1.956457	-1.853557	1.785505	H	4.299557	-2.617171	1.743817
H	4.995597	-0.302690	-1.802531	H	2.648149	0.449866	-1.773341
H	-2.435102	4.657544	0.039607	H	6.649254	-2.151891	0.932476
H	6.308943	-2.983475	-0.583171	H	6.896156	-1.320330	-0.613381
H	1.101287	5.418102	-0.730975	H	1.032560	5.388690	1.024959
H	2.373951	4.595717	0.184117				

3d* (excited state) b3lyp/6-311+g(d,p) scrf=(cpcm,solvent=acetonitrile)

```
# p opt freq td=(nstates=2)/b3lyp/6-311+g(d,p)
scrf=(cpcm,solvent=acetonitrile)
```

Charge = 0, Multiplicity = 1, Point group = C1

Electronic Energy = -3590.499212 Hartree

Number of imaginary frequencies = 0

Sum of electronic and zero-point Energies = -3590.072567 Hartree

Sum of electronic and thermal Energies = -3590.050998 Hartree

Sum of electronic and thermal Enthalpies = -3590.050053 Hartree

Sum of electronic and thermal Free Energies = -3590.125297 Hartree

	Cartesian Coordinates				Cartesian Coordinates		
	X	*Y*	*Z*		*X*	*Y*	*Z*
C	-1.483675	3.747593	0.141745	C	-0.075792	3.697470	0.107120
C	0.640182	2.474697	0.069359	C	-0.067939	1.281477	0.051882
C	-1.510996	1.350115	0.062651	C	-2.211067	2.566218	0.125528
C	0.288915	-0.118205	0.036379	C	-0.915854	-0.854162	0.035403
N	-2.003720	0.076298	0.053667	C	1.545280	-0.809157	-0.004337
C	1.488312	-2.264249	-0.052390	C	0.253994	-2.928195	-0.064368
C	-0.982698	-2.232842	-0.025079	C	2.799105	-0.169413	-0.002655
C	3.974619	-0.926120	-0.043569	C	3.950037	-2.325233	-0.087380
C	2.732813	-2.980210	-0.091570	C	-3.376926	-0.282633	0.013078
C	-4.208682	0.236781	-0.984261	C	-5.552900	-0.118089	-1.014897
C	-6.097666	-0.988966	-0.063115	C	-5.245107	-1.503401	0.924586
C	-3.899459	-1.162004	0.968169	Br	5.671832	-0.013725	-0.036181
O	0.571089	4.878794	0.123756	C	-7.560930	-1.348568	-0.083919
H	-1.974268	4.710565	0.188903	H	1.720412	2.509808	0.061731
H	-3.291440	2.585357	0.167874	H	0.247007	-4.011625	-0.105402
H	-1.922161	-2.765809	-0.049302	H	2.867928	0.906150	0.034088
H	4.874485	-2.889014	-0.117846	H	2.708265	-4.063768	-0.126810
H	-3.798319	0.890684	-1.743728	H	-6.185166	0.279991	-1.800869
H	-5.642044	-2.177979	1.675389	H	-3.259135	-1.556338	1.746906
H	1.529501	4.750334	0.104701	H	-7.990667	-1.210264	-1.077659
H	-7.719518	-2.384998	0.222056	H	-8.123072	-0.714577	0.610420

3e* (excited state) b3lyp/6-311+g(d,p) scrf=(cpcm,solvent=acetonitrile)

```
# p opt freq td=(nstates=2)/b3lyp/6-311+g(d,p)
scrf=(cpcm,solvent=acetonitrile)
```

Charge = 0, Multiplicity = 1, Point group = C1

Electronic Energy = -1109.227289 Hartree

Number of imaginary frequencies = 0

Sum of electronic and zero-point Energies = -1108.804608 Hartree

Sum of electronic and thermal Energies = -1108.783023 Hartree

Sum of electronic and thermal Enthalpies = -1108.782079 Hartree

Sum of electronic and thermal Free Energies = -1108.856384 Hartree

	Cartesian Coordinates				Cartesian Coordinates		
	X	*Y*	*Z*		*X*	*Y*	*Z*
C	-0.806721	3.746412	0.137138	C	0.603992	3.698064	0.100237
C	1.317884	2.472213	0.063268	C	0.609790	1.286117	0.047186
C	-0.829395	1.352687	0.061609	C	-1.530882	2.571492	0.124357
C	0.965771	-0.123288	0.030156	C	-0.234710	-0.844161	0.033162

N	-1.321110	0.082468	0.053570	C	2.222240	-0.816698	-0.012962
C	2.146966	-2.268915	-0.056941	C	0.911550	-2.922510	-0.063928
C	-0.314853	-2.234497	-0.022786	C	3.472841	-0.189851	-0.016346
C	4.676899	-0.944443	-0.059146	C	4.597861	-2.372912	-0.100422
C	3.388457	-3.004289	-0.099168	C	-2.697637	-0.277051	0.014761
C	-3.519302	0.215906	-1.002655	C	-4.864261	-0.137285	-1.031161
C	-5.415674	-0.979490	-0.057935	C	-4.570793	-1.467229	0.949741
C	-3.224766	-1.126992	0.992147	O	1.246054	4.875637	0.114201
C	-6.879398	-1.337107	-0.077277	C	5.926941	-0.303554	-0.059778
N	6.965492	0.232549	-0.060796	H	-1.295910	4.709952	0.184229
H	2.398085	2.504696	0.054438	H	-2.611205	2.589281	0.169053
H	0.902525	-4.006679	-0.104136	H	-1.258022	-2.759842	-0.042115
H	3.548617	0.886111	0.017502	H	5.514620	-2.950834	-0.132991
H	3.343978	-4.087569	-0.131447	H	-3.102114	0.847054	-1.777642
H	-5.491210	0.239571	-1.831558	H	-4.973787	-2.119601	1.716619
H	-2.589179	-1.500229	1.785137	H	2.205963	4.755055	0.094377
H	-7.303524	-1.224134	-1.076518	H	-7.041300	-2.364802	0.255097
H	-7.443431	-0.683963	0.597377				

6a* (excited state) b3lyp/6-311+g(d,p) scrf=(cpcm,solvent=acetonitrile)

```
# p opt freq td=(nstates=2)/b3lyp/6-311+g(d,p)
scrf=(cpcm,solvent=acetonitrile)
```

Charge = 0, Multiplicity = 1, Point group = C1

Electronic Energy = -1591.542287 Hartree

Number of imaginary frequencies = 0

Sum of electronic and zero-point Energies = -1590.946216 Hartree

Sum of electronic and thermal Energies = -1590.914986 Hartree

Sum of electronic and thermal Enthalpies = -1590.914042 Hartree

Sum of electronic and thermal Free Energies = -1591.009222 Hartree

	**Cartesian Coordinates**				**Cartesian Coordinates**		
	X	***Y***	***Z***		***X***	***Y***	***Z***
C	-0.502623	-3.114487	0.336714	C	0.686803	-2.423714	0.493873
C	0.748188	-0.973391	0.497354	C	-0.486578	-0.284701	0.201058
C	-1.693098	-1.046385	0.145970	C	-1.723935	-2.428145	0.186931
C	-0.849109	1.078728	-0.120588	C	-2.280819	1.106552	-0.219802
N	-2.763234	-0.177909	-0.067321	C	-0.139396	2.303272	-0.412744
C	-0.920262	3.504876	-0.596970	C	-2.341983	3.468492	-0.545612
C	-3.027969	2.277198	-0.392131	C	1.258351	2.390732	-0.598832
C	1.883922	3.587037	-0.914468	C	1.126253	4.766065	-1.033889
C	-0.245397	4.720389	-0.877446	C	-4.132683	-0.568326	-0.133692

C	-4.727788	-1.213881	0.952992	C	-6.062139	-1.601840	0.880647
C	-6.831390	-1.353170	-0.263134	C	-6.215194	-0.707195	-1.342602
C	-4.880549	-0.317715	-1.286722	O	1.769903	-3.189797	0.797987
C	-8.286295	-1.746143	-0.321341	C	1.925187	-0.307330	1.022042
C	3.259588	-0.546393	0.496740	C	4.408712	-0.024518	1.185405
C	4.215407	0.792293	2.323101	C	2.938270	1.099868	2.772483
C	1.811798	0.573154	2.139907	C	3.462598	-1.209546	-0.726460
C	4.750532	-1.458463	-1.221587	C	5.876138	-1.017087	-0.509374
C	5.693291	-0.301576	0.664442	O	0.618342	0.853583	2.725346
O	4.801045	-2.121705	-2.407098	C	6.078806	-2.404333	-2.987190
H	-0.481668	-4.195176	0.395178	H	-2.652477	-2.974785	0.089469
H	-2.889003	4.395362	-0.675033	H	-4.109280	2.246244	-0.408091
H	1.859649	1.499323	-0.510613	H	2.957664	3.611595	-1.058991
H	1.616120	5.707123	-1.258558	H	-0.836319	5.623187	-0.992296
H	-4.150361	-1.402333	1.849974	H	-6.513213	-2.101275	1.731391
H	-6.783383	-0.514790	-2.246382	H	-4.414903	0.164906	-2.137329
H	2.582077	-2.663106	0.776461	H	-8.485264	-2.626913	0.292690
H	-8.921397	-0.935737	0.052722	H	-8.599348	-1.960267	-1.345332
H	5.080348	1.195212	2.837969	H	2.790420	1.718858	3.649325
H	2.618685	-1.516813	-1.332525	H	6.877362	-1.202693	-0.872445
H	6.560845	0.074785	1.195645	H	-0.107581	0.499941	2.187482
H	5.869796	-2.932161	-3.915060	H	6.623768	-1.481773	-3.205000
H	6.675972	-3.041897	-2.329727				

6b* (excited state) b3lyp/6-311+g(d,p) scrf=(cpcm,solvent=acetonitrile)

```
# p opt freq td=(nstates=2)/b3lyp/6-311+g(d,p)
scrf=(cpcm,solvent=acetonitrile)
```

Charge = 0, Multiplicity = 1, Point group = C1

Electronic Energy = -6624.068847 Hartree

Number of imaginary frequencies = 0

Sum of electronic and zero-point Energies = -6623.528043 Hartree

Sum of electronic and thermal Energies = -6623.496191 Hartree

Sum of electronic and thermal Enthalpies = -6623.495247 Hartree

Sum of electronic and thermal Free Energies = -6623.595738 Hartree

	Cartesian Coordinates				Cartesian Coordinates		
	X	***Y***	***Z***		***X***	***Y***	***Z***
C	-1.639381	-3.029322	1.358531	C	-0.387036	-2.405183	1.552270
C	-0.095912	-1.034947	1.184535	C	-1.139919	-0.320341	0.588163
C	-2.435175	-0.956439	0.501002	C	-2.680070	-2.301808	0.845762
C	-1.327265	1.030487	0.028566	C	-2.695846	1.142651	-0.259479

N	-3.340956	-0.071868	0.019475	C	-0.482684	2.138387	-0.307250
C	-1.118220	3.334939	-0.802152	C	-2.524299	3.395392	-0.980495
C	-3.320702	2.304624	-0.735145	C	0.935477	2.116153	-0.245762
C	1.658678	3.220144	-0.625298	C	1.049510	4.412693	-1.068336
C	-0.318869	4.454557	-1.149858	C	-4.741592	-0.324433	-0.166477
C	-5.555495	-0.551217	0.942414	C	-6.911797	-0.800290	0.753266
C	-7.474410	-0.821842	-0.528631	C	-6.633168	-0.592452	-1.625482
C	-5.275236	-0.343734	-1.454846	O	0.551305	-3.134474	2.142572
C	-8.950362	-1.059973	-0.723886	C	1.241805	-0.534388	1.574732
C	2.435151	-1.029130	0.920168	C	3.726206	-0.652220	1.456418
C	3.778562	0.220578	2.561204	C	2.608731	0.744751	3.147322
C	1.368181	0.371440	2.664281	C	2.384584	-1.843273	-0.231574
C	3.573000	-2.324919	-0.806029	C	4.820813	-2.009012	-0.288892
C	4.894204	-1.173760	0.826652	Br	3.447513	-3.469685	-2.360297
O	0.263354	0.886942	3.307079	Br	3.577709	3.162725	-0.575293
H	-1.757408	-4.062154	1.657818	H	-3.661281	-2.735261	0.710817
H	-2.963530	4.316914	-1.343404	H	-4.389324	2.335194	-0.898663
H	1.438740	1.223226	0.089072	H	1.651447	5.267154	-1.347312
H	-0.810250	5.353434	-1.504429	H	-5.133695	-0.524344	1.939870
H	-7.541120	-0.976636	1.618446	H	-7.042663	-0.616542	-2.629234
H	-4.632906	-0.181736	-2.311920	H	1.347616	-2.576298	2.257480
H	-9.369935	-1.647903	0.094520	H	-9.491898	-0.108586	-0.757682
H	-9.147445	-1.581233	-1.662880	H	4.746746	0.509449	2.954740
H	2.661627	1.422131	3.991122	H	1.432880	-2.093373	-0.681213
H	5.724553	-2.395806	-0.743237	H	5.865591	-0.907129	1.228751
H	-0.545916	0.537765	2.915302				

7* (excited state) b3lyp/6-311+g(d,p) scrf=(cpcm,solvent=acetonitrile)

```
# p opt freq td=(nstates=2)/b3lyp/6-311+g(d,p)
scrf=(cpcm,solvent=acetonitrile)
```

Charge = 0, Multiplicity = 1, Point group = C1

Electronic Energy = -1722.950252 Hartree

Number of imaginary frequencies = 0

Sum of electronic and zero-point Energies = -1722.330561 Hartree

Sum of electronic and thermal Energies = -1722.299057 Hartree

Sum of electronic and thermal Enthalpies = -1722.298113 Hartree

Sum of electronic and thermal Free Energies = -1722.394621 Hartree

	**Cartesian Coordinates**				**Cartesian Coordinates**		
	X	***Y***	***Z***		***X***	***Y***	***Z***
C	4.154588	2.998194	-2.341721	C	5.514242	2.675971	-2.199822

C	5.923169	1.601258	-1.416326	C	4.953692	0.819597	-0.767716
C	3.584754	1.148547	-0.939011	C	3.180142	2.239720	-1.708599
C	5.000518	-0.322026	0.126334	C	3.668547	-0.629087	0.455910
N	2.808543	0.260558	-0.185823	C	6.059547	-1.123299	0.658109
C	5.702058	-2.225833	1.505426	C	4.328740	-2.500091	1.781589
C	3.318309	-1.727788	1.271285	C	7.436743	-0.899476	0.402302
C	8.407163	-1.711378	0.951369	C	8.053018	-2.792675	1.785054
C	6.724738	-3.040070	2.052707	C	1.396061	0.268631	-0.095540
C	0.616510	0.239591	-1.259886	C	-0.766963	0.245282	-1.176052
C	-1.395346	0.287093	0.077126	C	-0.616226	0.312704	1.243096
C	0.767785	0.305379	1.155434	O	6.406594	3.480228	-2.864907
N	-2.803275	0.301501	0.161514	C	-4.095905	3.018605	2.355160
C	-5.452429	2.674130	2.231688	C	-5.890056	1.587874	1.435167
C	-4.944960	0.821702	0.757989	C	-3.555490	1.168356	0.922005
C	-3.134952	2.262019	1.687320	C	-5.003922	-0.301792	-0.143921
C	-3.672955	-0.620627	-0.504446	C	-6.087498	-1.093957	-0.655615
C	-5.731205	-2.215332	-1.515033	C	-4.382966	-2.487551	-1.811818
C	-3.325219	-1.691873	-1.305690	C	-7.448628	-0.857737	-0.378530
C	-8.457452	-1.669971	-0.906067	C	-8.117382	-2.752738	-1.735211
C	-6.793891	-3.021617	-2.034168	O	-6.338933	3.442214	2.903830
H	3.878442	3.850596	-2.950208	H	6.980251	1.393281	-1.318058
H	2.135679	2.502277	-1.813939	H	4.089399	-3.350042	2.411051
H	2.283033	-1.960526	1.482319	H	7.741690	-0.078720	-0.230554
H	9.452289	-1.515331	0.739734	H	8.824316	-3.424011	2.210742
H	6.440516	-3.869919	2.691319	H	1.095724	0.201143	-2.229679
H	-1.362963	0.233540	-2.078781	H	-1.094119	0.320159	2.213825
H	1.362914	0.335406	2.059093	H	7.309320	3.183189	-2.699207
H	-3.818845	3.871304	2.960328	H	-6.950464	1.388738	1.368402
H	-2.089161	2.527779	1.758242	H	-4.149883	-3.333117	-2.449006
H	-2.296961	-1.931795	-1.535355	H	-7.732807	-0.027571	0.252453
H	-9.495896	-1.461978	-0.675765	H	-8.899918	-3.383405	-2.145033
H	-6.538728	-3.859558	-2.674813	H	-7.243253	3.136201	2.752282

10a* (excited state) b3lyp/6-311+g(d,p) scrf=(cpcm,solvent=acetonitrile)

```
# p opt freq td=(nstates=2)/b3lyp/6-311+g(d,p)
scrf=(cpcm,solvent=acetonitrile)
```

Charge = 0, Multiplicity = 1, Point group = C1

Electronic Energy = -785.834713 Hartree

Number of imaginary frequencies = 0

Sum of electronic and zero-point Energies = -785.472494 Hartree

Sum of electronic and thermal Energies = -785.457666 Hartree

Sum of electronic and thermal Enthalpies = -785.456721 Hartree

Sum of electronic and thermal Free Energies = -785.514004 Hartree

	Cartesian Coordinates				Cartesian Coordinates		
	X	*Y*	*Z*		*X*	*Y*	*Z*
C	-3.668553	-0.901612	0.000017	C	-3.146428	0.421435	-0.000019
C	-1.754122	0.686677	-0.000020	C	-0.870169	-0.387307	0.000025
C	-1.426535	-1.730064	0.000039	C	-2.809957	-1.985874	0.000043
C	0.565118	-0.532289	0.000024	C	0.838403	-1.918815	0.000013
N	-0.398882	-2.602636	0.000032	C	1.655327	0.407093	0.000029
C	3.000288	-0.158661	-0.000043	C	3.192597	-1.547944	-0.000072
C	2.106327	-2.466794	-0.000034	C	1.505254	1.804419	0.000110
C	2.613332	2.663772	0.000091	C	3.912413	2.124820	-0.000007
C	4.108326	0.757388	-0.000072	O	-4.076468	1.386254	-0.000054
C	-3.694701	2.773854	-0.000066	H	-4.742878	-1.029181	0.000025
H	-1.402790	1.705030	-0.000077	H	-3.186349	-3.001018	0.000064
H	-0.495488	-3.608623	0.000034	H	4.207204	-1.931397	-0.000121
H	2.268635	-3.536826	-0.000048	H	0.515138	2.239061	0.000203
H	2.465950	3.737517	0.000155	H	4.769960	2.790399	-0.000030
H	5.113671	0.348729	-0.000141	H	-4.629802	3.327272	-0.000045
H	-3.120401	3.013434	0.896434	H	-3.120445	3.013428	-0.896596

10b* (excited state) b3lyp/6-311+g(d,p) scrf=(cpcm,solvent=acetonitrile)

```
# p opt freq td=(nstates=2)/b3lyp/6-311+g(d,p)
scrf=(cpcm,solvent=acetonitrile)
```

Charge = 0, Multiplicity = 1, Point group = C1

Electronic Energy = -825.151437 Hartree

Number of imaginary frequencies = 0

Sum of electronic and zero-point Energies = -824.765075 Hartree

Sum of electronic and thermal Energies = -824.748536 Hartree

Sum of electronic and thermal Enthalpies = -824.747592 Hartree

Sum of electronic and thermal Free Energies = -824.808823 Hartree

	Cartesian Coordinates				Cartesian Coordinates		
	X	*Y*	*Z*		*X*	*Y*	*Z*
C	3.661006	0.519488	-0.000069	C	3.092277	-0.779375	0.000191
C	1.689067	-0.986559	0.000211	C	0.848557	0.119616	-0.000209
C	1.448121	1.440811	-0.000397	C	2.841615	1.635575	-0.000308
C	-0.583301	0.309233	-0.000002	C	-0.807793	1.702125	0.000059
N	0.452082	2.358847	-0.000243	C	-1.701637	-0.593278	-0.000237
C	-3.027406	0.016007	0.000485	C	-3.174239	1.411614	0.000829

C	-2.058440	2.292930	0.000479	C	-1.597189	-1.996328	-0.001133
C	-2.731249	-2.818713	-0.001059	C	-4.011884	-2.236811	-0.000050
C	-4.163192	-0.863456	0.000699	C	0.589505	3.802464	-0.000489
O	3.981001	-1.783650	0.000424	C	3.539068	-3.152851	0.000654
H	4.738918	0.611075	-0.000066	H	1.297129	-1.989973	0.000727
H	3.272001	2.628089	-0.000340	H	-4.175682	1.828062	0.001396
H	-2.191860	3.366223	0.000502	H	-0.621798	-2.462734	-0.002040
H	-2.619349	-3.896720	-0.001785	H	-4.891069	-2.873642	0.000122
H	-5.155061	-0.423014	0.001410	H	0.108377	4.220134	-0.888558
H	0.109498	4.220360	0.888070	H	1.640361	4.077750	-0.001192
H	4.448883	-3.747150	0.000724	H	2.954914	-3.367926	0.897120
H	2.954843	-3.368215	-0.895696				

10c* (excited state) b3lyp/6-311+g(d,p) scrf=(cpcm,solvent=acetonitrile)

```
# p opt freq td=(nstates=2)/b3lyp/6-311+g(d,p)
scrf=(cpcm,solvent=acetonitrile)
```

Charge = 0, Multiplicity = 1, Point group = C1

Electronic Energy = -1056.263251 Hartree

Number of imaginary frequencies = 0

Sum of electronic and zero-point Energies = -1055.795760 Hartree

Sum of electronic and thermal Energies = -1055.774314 Hartree

Sum of electronic and thermal Enthalpies = -1055.773370 Hartree

Sum of electronic and thermal Free Energies = -1055.847054 Hartree

	**Cartesian Coordinates**				**Cartesian Coordinates**		
	X	***Y***	***Z***		***X***	***Y***	***Z***
C	0.055369	3.551486	0.114141	C	1.446915	3.289697	0.077787
C	1.962491	1.969870	0.041247	C	1.071902	0.900718	0.029224
C	-0.344333	1.190076	0.043527	C	-0.847528	2.499982	0.102187
C	1.211599	-0.535625	0.016627	C	-0.091716	-1.079558	0.021457
N	-1.025391	0.006775	0.041424	C	2.350166	-1.412962	-0.026813
C	2.069982	-2.843253	-0.071654	C	0.746741	-3.310935	-0.076954
C	-0.368896	-2.431863	-0.035222	C	3.690248	-0.983536	-0.031368
C	4.754547	-1.892981	-0.075162	C	4.489418	-3.273194	-0.115732
C	3.189017	-3.741137	-0.114270	C	-2.437978	-0.138484	0.014079
C	-3.189928	0.490526	-0.983075	C	-4.573183	0.344815	-1.000115
C	-5.236243	-0.422978	-0.035024	C	-4.463315	-1.050964	0.952249
C	-3.080757	-0.917480	0.982538	C	-6.737732	-0.555007	-0.041475
O	2.227897	4.381453	0.092005	C	3.660381	4.255985	0.070655
H	-0.277129	4.579985	0.158552	H	3.028058	1.813222	0.030843
H	-1.911942	2.687093	0.143610	H	0.572941	-4.380713	-0.115062

H	-1.379710	-2.813568	-0.054406	H	3.917174	0.072621	0.002904
H	5.776226	-1.531228	-0.075789	H	5.314112	-3.978298	-0.148780
H	2.991950	-4.807825	-0.146986	H	-2.691563	1.067586	-1.752161
H	-5.144211	0.827381	-1.785754	H	-4.951567	-1.650135	1.713223
H	-2.500912	-1.397967	1.760380	H	-7.149538	-0.365376	-1.034417
H	-7.050367	-1.551221	0.279289	H	-7.191284	0.165964	0.647400
H	4.037528	5.275112	0.087961	H	4.012377	3.715156	0.951076
H	3.989419	3.753987	-0.841027				

12* (excited state) b3lyp/6-311+g(d,p) scrf=(cpcm,solvent=acetonitrile)

```
# p opt freq td=(nstates=2)/b3lyp/6-311+g(d,p)
scrf=(cpcm,solvent=acetonitrile)
```

Charge = 0, Multiplicity = 1, Point group = C1

Electronic Energy = -5664.689486 Hartree

Number of imaginary frequencies = 0

Sum of electronic and zero-point Energies = -5664.410921 Hartree

Sum of electronic and thermal Energies = -5664.398286 Hartree

Sum of electronic and thermal Enthalpies = -5664.397342 Hartree

Sum of electronic and thermal Free Energies = -5664.451888 Hartree

	Cartesian Coordinates				Cartesian Coordinates		
	X	*Y*	*Z*		*X*	*Y*	*Z*
C	-1.139999	1.924662	0.000004	C	-0.716967	0.554837	0.000004
C	0.716967	0.554837	0.000002	C	1.139998	1.924662	-0.000002
N	-0.000002	2.701260	0.000001	C	-2.468133	2.324707	0.000004
C	-3.460217	1.305689	0.000004	C	-3.051617	-0.024583	-0.000000
C	-1.701235	-0.448688	-0.000003	C	1.701236	-0.448688	0.000008
C	3.051617	-0.024581	0.000001	C	3.460216	1.305690	-0.000007
C	2.468131	2.324708	-0.000007	Br	-4.394750	-1.387585	-0.000002
Br	4.394751	-1.387585	0.000001	H	-0.000002	3.714231	0.000001
H	-2.745306	3.371388	0.000004	H	-4.509416	1.563060	0.000008
H	-1.451169	-1.500257	-0.000011	H	1.451170	-1.500257	0.000023
H	4.509415	1.563062	-0.000018	H	2.745303	3.371389	-0.000009

3a* (excited state) b3lyp/6-31+g(d,p) scrf=(cpcm,solvent=acetonitrile)

p opt freq td=(nstates=2)/b3lyp/6-31+g(d,p) scrf=(cpcm,solvent=aceto nitrile)

Charge = 0, Multiplicity = 1, Point group = C1

Electronic Energy = −1016.765324 Hartree

Number of imaginary frequencies = 0

Sum of electronic and zero-point Energies = −1016.323672 Hartree

Sum of electronic and thermal Energies = −1016.303776 Hartree

Sum of electronic and thermal Enthalpies = −1016.302831 Hartree

Sum of electronic and thermal Free Energies = −1016.373284 Hartree

Atoms	Cartesian Coordinates			Atoms	Cartesian Coordinates		
	X	*Y*	*Z*		*X*	*Y*	*Z*
C	−0.332943	3.760954	0.136486	C	1.074789	3.703003	0.095295
C	1.789117	2.474765	0.051917	C	1.074490	1.279627	0.036846
C	−0.369066	1.356352	0.051469	C	−1.066364	2.574370	0.121857
C	1.427127	−0.122335	0.021244	C	0.216677	−0.856631	0.024684
N	−0.867276	0.078143	0.045513	C	2.684316	−0.819152	−0.023994
C	2.622175	−2.277489	−0.077337	C	1.380017	−2.941351	−0.088331
C	0.145118	−2.238909	−0.041806	C	3.947795	−0.190015	−0.022246
C	5.138218	−0.929974	−0.067608	C	5.083256	−2.337373	−0.117353
C	3.863728	−2.996783	−0.122235	C	−2.240079	−0.275519	0.014735
C	−3.087868	0.272349	−0.958592	C	−4.437526	−0.078499	−0.976730
C	−4.972145	−0.972956	−0.037080	C	−4.104492	−1.511720	0.930782
C	−2.754456	−1.177180	0.961019	O	1.731742	4.884230	0.110835
C	−6.430187	−1.359465	−0.061867	H	−0.822544	4.726215	0.187469
H	2.871337	2.503726	0.040074	H	−2.148253	2.597559	0.170431
H	1.369287	−4.026591	−0.133816	H	−0.798247	−2.769292	−0.066321
H	4.011673	0.889973	0.019497	H	6.095517	−0.417577	−0.062813
H	6.005678	−2.912146	−0.152112	H	3.829200	−4.082694	−0.161734
H	−2.687336	0.945709	−1.708754	H	−5.082189	0.344493	−1.741855
H	−4.494938	−2.198381	1.676943	H	−2.104097	−1.588098	1.725239
H	2.692188	4.750429	0.086988	H	−6.980271	−0.793864	−0.818035
H	−6.548572	−2.426479	−0.284146	H	−6.901615	−1.180232	0.910739

3b* (excited state) b3lyp/6-31+g(d,p) scrf=(cpcm,solvent=acetonitrile)

p opt freq td=(nstates=2)/b3lyp/6-31+g(d,p) scrf=(cpcm,solvent=aceto nitrile)

Charge = 0, Multiplicity = 1, Point group = C1

Electronic Energy = −1437.037837 Hartree

Number of imaginary frequencies = 0

Sum of electronic and zero-point Energies = −1436.631690 Hartree

Sum of electronic and thermal Energies = −1436.612517 Hartree

Sum of electronic and thermal Enthalpies = −1436.611572 Hartree

Sum of electronic and thermal Free Energies = −1436.679761 Hartree

Atoms	Cartesian Coordinates			Atoms	Cartesian Coordinates		
	X	*Y*	*Z*		*X*	*Y*	*Z*
C	−0.181787	3.818059	0.135276	C	−1.586783	3.679820	0.092906

C	−2.231393	2.414304	0.048780	C	−1.450824	1.261255	0.035670
C	−0.011139	1.420798	0.051470	C	0.617195	2.677537	0.120428
C	−1.722587	−0.156481	0.018260	C	−0.472425	−0.821019	0.018942
N	0.557858	0.175294	0.043212	C	−2.938354	−0.924966	−0.024298
C	−2.792523	−2.377082	−0.079965	C	−1.514490	−2.968177	−0.094293
C	−0.321520	−2.195420	−0.048061	C	−4.235682	−0.370494	−0.017795
C	−5.381127	−1.178747	−0.062366	C	−5.245744	−2.580387	−0.114967
C	−3.990106	−3.167692	−0.123463	C	1.949280	−0.100066	0.019548
C	2.508973	−0.951931	0.983239	C	3.876010	−1.219256	0.966713
C	4.674137	−0.635140	−0.020036	C	4.129897	0.209652	−0.989000
C	2.761956	0.475813	−0.968141	O	−2.304293	4.822780	0.107196
Cl	6.398304	−0.974853	−0.044018	H	0.250327	4.810326	0.186064
H	−3.313397	2.382000	0.032980	H	1.696141	2.762058	0.167295
H	−1.440718	−4.050798	−0.141790	H	0.650502	−2.671707	−0.073429
H	−4.362866	0.703816	0.026635	H	−6.366394	−0.722414	−0.054869
H	−6.133560	−3.207196	−0.149105	H	−3.892092	−4.249618	−0.164899
H	1.882523	−1.386133	1.754078	H	4.314974	−1.866981	1.716947
H	4.759568	0.646001	−1.755922	H	2.324279	1.110912	−1.730281
H	−3.256884	4.640664	0.081481				

3c* (excited state) b3lyp/6-31+g(d,p) scrf=(cpcm,solvent=acetonitrile)

p opt freq td=(nstates=2)/b3lyp/6-31+g(d,p) scrf=(cpcm,solvent=aceto nitrile)

Charge = 0, Multiplicity = 1, Point group = C1

Electronic Energy = −1056.085420 Hartree

Number of imaginary frequencies = 0

Sum of electronic and zero-point Energies = −1055.616892 Hartree

Sum of electronic and thermal Energies = −1055.595239 Hartree

Sum of electronic and thermal Enthalpies = −1055.594295 Hartree

Sum of electronic and thermal Free Energies = −1055.668370 Hartree

Atoms	Cartesian Coordinates			Atoms	Cartesian Coordinates		
	X	*Y*	*Z*		*X*	*Y*	*Z*
C	0.533199	3.511788	0.094979	C	−0.888413	3.527868	0.057171
C	−1.684980	2.353002	0.027318	C	−1.059702	1.111402	0.021070
C	0.387520	1.095005	0.034293	C	1.163440	2.267290	0.087593
C	−1.502152	−0.264969	0.014228	C	−0.341841	−1.072436	0.020926
N	0.803001	−0.207446	0.036761	C	−2.803113	−0.878223	−0.022925
C	−2.834715	−2.337817	−0.061600	C	−1.638212	−3.077697	−0.066932
C	−0.357485	−2.456166	−0.029892	C	−4.021789	−0.168435	−0.026650
C	−5.259818	−0.831700	−0.063331	C	−5.296240	−2.239247	−0.098827

C	−4.121954	−2.977163	−0.098072	C	2.152213	−0.651731	0.008399
C	2.617118	−1.543339	0.987238	C	3.942622	−1.968187	0.959000
C	4.831970	−1.528252	−0.037865	C	4.346260	−0.639676	−1.008804
C	3.021291	−0.202472	−0.994659	O	−1.470356	4.746957	0.061748
C	6.259831	−2.016226	−0.065880	C	1.307844	4.801220	0.148249
H	−2.762810	2.460090	0.017755	H	2.245123	2.210023	0.128667
H	−1.696446	−4.162123	−0.100622	H	0.550087	−3.046157	−0.047388
H	−4.014753	0.913970	0.003493	H	−6.181890	−0.258125	−0.063243
H	−6.254217	−2.753237	−0.127011	H	−4.157541	−4.063419	−0.126653
H	1.949123	−1.879964	1.772622	H	4.295621	−2.647752	1.729968
H	5.009334	−0.288768	−1.794534	H	2.657351	0.467603	−1.766390
H	−2.436978	4.670423	0.040041	H	6.723014	−1.942018	0.923631
H	6.306270	−3.070311	−0.365147	H	6.864017	−1.441200	−0.772297
H	1.106603	5.428416	−0.728396	H	1.041302	5.395506	1.030214
H	2.381282	4.600472	0.183826				

3d* (excited state) b3lyp/6-31+g(d,p) scrf=(cpcm,solvent=acetonitrile)

p opt freq td=(nstates=2)/b3lyp/6-31+g(d,p) scrf=(cpcm,solvent=aceto nitrile)

Charge = 0, Multiplicity = 1, Point group = C1

Electronic Energy = −3587.893515 Hartree

Number of imaginary frequencies = 0

Sum of electronic and zero-point Energies = −3587.464510 Hartree

Sum of electronic and thermal Energies = −3587.443202 Hartree

Sum of electronic and thermal Enthalpies = −3587.442257 Hartree

Sum of electronic and thermal Free Energies = −3587.517352 Hartree

Atoms	Cartesian Coordinates			Atoms	Cartesian Coordinates		
	X	*Y*	*Z*		*X*	*Y*	*Z*
C	−1.479228	3.758044	0.142593	C	−0.068585	3.704782	0.106262
C	0.647252	2.478965	0.066709	C	−0.064976	1.284602	0.050797
C	−1.509385	1.355609	0.062052	C	−2.209832	2.574629	0.126914
C	0.290044	−0.117416	0.035599	C	−0.918035	−0.852342	0.035044
N	−2.005289	0.079957	0.053822	C	1.547650	−0.809699	−0.005487
C	1.488515	−2.266854	−0.055455	C	0.250765	−2.931170	−0.068063
C	−0.987494	−2.234277	−0.027349	C	2.803258	−0.168746	−0.001251
C	3.980980	−0.926951	−0.042638	C	3.954164	−2.328152	−0.089123
C	2.733879	−2.984717	−0.094636	C	−3.378230	−0.278255	0.013620
C	−4.216139	0.254318	−0.975863	C	−5.565267	−0.098661	−1.002205
C	−6.107104	−0.982110	−0.056392	C	−5.248453	−1.506031	0.927239
C	−3.899367	−1.167408	0.967050	Br	5.670179	−0.020839	−0.035286

O	0.580759	4.887971	0.122446	C	−7.562913	−1.376198	−0.094625
H	−1.969637	4.723089	0.190977	H	1.729270	2.510358	0.057458
H	−3.291931	2.595702	0.170830	H	0.243054	−4.016514	−0.110476
H	−1.928880	−2.767687	−0.052760	H	2.873125	0.908373	0.038027
H	4.879404	−2.894517	−0.120009	H	2.708199	−4.070153	−0.131183
H	−3.809309	0.919280	−1.730275	H	−6.203478	0.313781	−1.778411
H	−5.645023	−2.183555	1.678466	H	−3.255750	−1.566534	1.743280
H	1.542510	4.761793	0.102201	H	−8.118455	−0.781010	−0.823673
H	−7.674396	−2.432632	−0.366521	H	−8.033019	−1.245649	0.886001

3e* (excited state) b3lyp/6-31+g(d,p) scrf=(cpcm,solvent=acetonitrile)

p opt freq td=(nstates=2)/b3lyp/6-31+g(d,p) scrf=(cpcm,solvent=aceto nitrile)

Charge = 0, Multiplicity = 1, Point group = C1

Electronic Energy = −1109.014319 Hartree

Number of imaginary frequencies = 0

Sum of electronic and zero-point Energies = −1108.590470 Hartree

Sum of electronic and thermal Energies = −1108.568863 Hartree

Sum of electronic and thermal Enthalpies = −1108.567918 Hartree

Sum of electronic and thermal Free Energies = −1108.642521 Hartree

Atoms	Cartesian Coordinates			Atoms	Cartesian Coordinates		
	X	*Y*	*Z*		*X*	*Y*	*Z*
C	−0.803506	3.756213	0.138038	C	0.609967	3.704718	0.099582
C	1.324017	2.475830	0.061727	C	0.611990	1.288290	0.046418
C	−0.828675	1.357426	0.060839	C	−1.530817	2.579122	0.125898
C	0.966055	−0.123511	0.029743	C	−0.238205	−0.842991	0.033412
N	−1.323760	0.085684	0.053777	C	2.223451	−0.819878	−0.013338
C	2.144936	−2.274125	−0.058987	C	0.905456	−2.927194	−0.066407
C	−0.322017	−2.236449	−0.023745	C	3.477451	−0.193305	−0.015807
C	4.682811	−0.952190	−0.059204	C	4.600907	−2.383487	−0.102171
C	3.386433	−3.013366	−0.101808	C	−2.700411	−0.272334	0.015045
C	−3.526397	0.230965	−0.997813	C	−4.876533	−0.119634	−1.022841
C	−5.426795	−0.970809	−0.052970	C	−4.577404	−1.465396	0.953692
C	−3.227832	−1.128452	0.993280	O	1.254348	4.884256	0.112876
C	−6.884029	−1.359982	−0.086915	C	5.937016	−0.311708	−0.058654
N	6.983903	0.227090	−0.058461	H	−1.292542	4.721789	0.186150
H	2.405967	2.505243	0.052202	H	−2.612858	2.598403	0.172059
H	0.894738	−4.013224	−0.108011	H	−1.267575	−2.761459	−0.043535
H	3.555339	0.884228	0.019885	H	5.517831	−2.964616	−0.135174
H	3.339041	−4.098424	−0.135299	H	−3.111184	0.870290	−1.769782

H	−5.508083	0.269346	−1.816231	H	−4.981348	−2.118249	1.722466
H	−2.590034	−1.504496	1.785782	H	2.217558	4.766163	0.091657
H	−7.426124	−0.809827	−0.859986	H	−6.997382	−2.431110	−0.291295
H	−7.366334	−1.164036	0.876822				

6a* (excited state) b3lyp/6-31+g(d,p) scrf=(cpcm,solvent=acetonitrile)

p opt freq td=(nstates=2)/b3lyp/6-31+g(d,p) scrf=(cpcm,solvent=aceto nitrile)

Charge = 0, Multiplicity = 1, Point group = C1

Electronic Energy = −1591.234167 Hartree

Number of imaginary frequencies = 0

Sum of electronic and zero-point Energies = −1590.636113 Hartree

Sum of electronic and thermal Energies = −1590.605765 Hartree

Sum of electronic and thermal Enthalpies = −1590.604821 Hartree

Sum of electronic and thermal Free Energies = −1590.697261 Hartree

Atoms	Cartesian Coordinates			Atoms	Cartesian Coordinates		
	X	*Y*	*Z*		*X*	*Y*	*Z*
C	−0.505608	−3.119582	0.338343	C	0.686230	−2.425776	0.496400
C	0.749749	−0.973240	0.497763	C	−0.487119	−0.283991	0.199695
C	−1.695502	−1.046563	0.144753	C	−1.728758	−2.431884	0.187366
C	−0.850957	1.081061	−0.124698	C	−2.284328	1.107729	−0.223977
N	−2.767280	−0.178195	−0.069788	C	−0.140648	2.307749	−0.415619
C	−0.923723	3.510346	−0.604642	C	−2.348288	3.473122	−0.554792
C	−3.035292	2.279416	−0.398545	C	1.260794	2.396919	−0.595471
C	1.887656	3.596773	−0.910462	C	1.127268	4.777050	−1.036209
C	−0.248734	4.728715	−0.885187	C	−4.136439	−0.569173	−0.134883
C	−4.729988	−1.220992	0.954175	C	−6.067054	−1.610289	0.883258
C	−6.841627	−1.356794	−0.260831	C	−6.227219	−0.704476	−1.341441
C	−4.888852	−0.313473	−1.287677	O	1.769819	−3.194300	0.802726
C	−8.297558	−1.753275	−0.314668	C	1.928356	−0.305032	1.022459
C	3.264747	−0.543190	0.495803	C	4.415844	−0.013257	1.181117
C	4.222294	0.809582	2.318051	C	2.941831	1.113341	2.772328
C	1.814856	0.576313	2.143169	C	3.468650	−1.218580	−0.724396
C	4.759755	−1.468692	−1.219370	C	5.888091	−1.017734	−0.511792
C	5.703261	−0.291531	0.659557	O	0.619489	0.851924	2.733606
O	4.809877	−2.143815	−2.401601	C	6.088425	−2.425346	−2.982184
H	−0.484408	−4.202012	0.397871	H	−2.659348	−2.978636	0.090076
H	−2.896872	4.400917	−0.687086	H	−4.118398	2.247526	−0.414454
H	1.863214	1.504584	−0.503164	H	2.964123	3.622025	−1.049250
H	1.617080	5.720377	−1.260660	H	−0.842054	5.631734	−1.004132

H	−4.149002	−1.412738	1.850458	H	−6.516640	−2.114393	1.734536
H	−6.799159	−0.507346	−2.244049	H	−4.425130	0.173964	−2.139014
H	2.587550	−2.670812	0.776418	H	−8.472320	−2.699605	0.206162
H	−8.925666	−0.994825	0.169108	H	−8.644298	−1.857805	−1.346516
H	5.088591	1.219421	2.829170	H	2.793795	1.734791	3.649610
H	2.623623	−1.531054	−1.329429	H	6.891317	−1.203757	−0.874324
H	6.571734	0.091584	1.188331	H	−0.110184	0.489922	2.201328
H	5.879218	−2.962042	−3.907115	H	6.629555	−1.500242	−3.208023
H	6.690484	−3.056139	−2.319534				

6b* (excited state) b3lyp/6-31+g(d,p) scrf=(cpcm,solvent=acetonitrile)

p opt freq td=(nstates=2)/b3lyp/6-31+g(d,p) scrf=(cpcm,solvent=aceto nitrile)

Charge = 0, Multiplicity = 1, Point group = C1

Electronic Energy = −6618.962567 Hartree

Number of imaginary frequencies = 0

Sum of electronic and zero-point Energies = −6618.418949 Hartree

Sum of electronic and thermal Energies = −6618.387410 Hartree

Sum of electronic and thermal Enthalpies = −6618.386466 Hartree

Sum of electronic and thermal Free Energies = −6618.486424 Hartree

Atoms	Cartesian Coordinates			Atoms	Cartesian Coordinates		
	X	*Y*	*Z*		*X*	*Y*	*Z*
C	−1.748256	−2.965272	1.530526	C	−0.474088	−2.371589	1.712053
C	−0.130639	−1.030683	1.287790	C	−1.140658	−0.307578	0.644211
C	−2.458324	−0.902412	0.563982	C	−2.756510	−2.224680	0.968270
C	−1.273723	1.025137	0.028411	C	−2.633753	1.172864	−0.286417
N	−3.326539	−0.008056	0.030427	C	−0.383126	2.090429	−0.327178
C	−0.965624	3.295324	−0.869065	C	−2.367744	3.396411	−1.081033
C	−3.209754	2.340775	−0.816615	C	1.031732	2.018466	−0.229827
C	1.805697	3.090352	−0.609451	C	1.249400	4.292632	−1.101174
C	−0.117658	4.378987	−1.225416	C	−4.732475	−0.218470	−0.168495
C	−5.572820	−0.370008	0.937816	C	−6.937281	−0.580122	0.734673
C	−7.481090	−0.635713	−0.558076	C	−6.612809	−0.481036	−1.651538
C	−5.246456	−0.272281	−1.467656	O	0.443140	−3.103585	2.336177
C	−8.963004	−0.829878	−0.769815	C	1.235231	−0.574335	1.653351
C	2.387658	−1.047158	0.912531	C	3.708750	−0.666038	1.374340
C	3.824286	0.178263	2.499247	C	2.688094	0.678761	3.176058
C	1.421594	0.304155	2.759653	C	2.267617	−1.843934	−0.247286
C	3.423729	−2.298965	−0.911319	C	4.701237	−1.976964	−0.471533
C	4.839974	−1.162241	0.658013	Br	3.202614	−3.416412	−2.460034

O	0.349618	0.802276	3.475136	Br	3.708082	2.951853	−0.505847
H	−1.905899	−3.981764	1.870768	H	−3.751923	−2.630489	0.836961
H	−2.768904	4.321324	−1.483103	H	−4.275016	2.403733	−1.003139
H	1.495806	1.116560	0.137766	H	1.888491	5.120869	−1.384285
H	−0.568337	5.285626	−1.617987	H	−5.165421	−0.315614	1.942199
H	−7.587423	−0.698705	1.596631	H	−7.007962	−0.532037	−2.662049
H	−4.583633	−0.168710	−2.320572	H	1.252310	−2.549914	2.429281
H	−9.420766	−1.355153	0.072833	H	−9.468864	0.138435	−0.869082
H	−9.163435	−1.397488	−1.683119	H	4.815111	0.466477	2.838640
H	2.790990	1.341274	4.029373	H	1.289959	−2.103639	−0.635652
H	5.578919	−2.345004	−0.992707	H	5.834391	−0.890381	1.001355
H	−0.484295	0.481815	3.104042				

7* (excited state) b3lyp/6-31+g(d,p) scrf=(cpcm,solvent=acetonitrile)

p opt freq td=(nstates=2)/b3lyp/6-31+g(d,p) scrf=(cpcm,solvent=aceto nitrile)

Charge = 0, Multiplicity = 1, Point group = C1

Electronic Energy = −1722.624021 Hartree

Number of imaginary frequencies = 0

Sum of electronic and zero-point Energies = −1722.002127 Hartree

Sum of electronic and thermal Energies = −1721.970703 Hartree

Sum of electronic and thermal Enthalpies = −1721.969759 Hartree

Sum of electronic and thermal Free Energies = −1722.065913 Hartree

Atoms	Cartesian Coordinates			Atoms	Cartesian Coordinates		
	X	*Y*	*Z*		*X*	*Y*	*Z*
C	4.151857	2.972210	−2.389596	C	5.515037	2.653389	−2.243666
C	5.928691	1.586940	−1.445182	C	4.959243	0.810865	−0.784700
C	3.587029	1.134459	−0.960549	C	3.177429	2.217544	−1.744565
C	5.008942	−0.319672	0.125786	C	3.675292	−0.625759	0.460124
N	2.811889	0.254171	−0.194462	C	6.071998	−1.111440	0.669543
C	5.716233	−2.206329	1.532558	C	4.341288	−2.481834	1.811771
C	3.325887	−1.716969	1.290179	C	7.451007	−0.885775	0.410977
C	8.426319	−1.689304	0.972857	C	8.074229	−2.763091	1.822168
C	6.742748	−3.011563	2.092463	C	1.400215	0.262110	−0.101586
C	0.616018	0.231608	−1.267556	C	−0.770346	0.236820	−1.180829
C	−1.398949	0.281240	0.076958	C	−0.614439	0.308639	1.244640
C	0.772321	0.300258	1.154152	O	6.405831	3.452101	−2.920295
N	−2.805321	0.296295	0.164057	C	−4.091686	2.989862	2.405788
C	−5.451075	2.647093	2.281490	C	−5.893250	1.571016	1.470100
C	−4.948253	0.812342	0.776803	C	−3.556947	1.153981	0.943388

C	−3.131602	2.239126	1.722228	C	−5.011831	−0.297719	−0.144118
C	−3.679425	−0.612942	−0.514827	C	−6.100175	−1.080314	−0.665984
C	−5.748544	−2.191658	−1.543835	C	−4.397985	−2.463933	−1.848308
C	−3.335714	−1.676444	−1.334282	C	−7.463009	−0.843612	−0.381663
C	−8.477550	−1.647326	−0.919985	C	−8.141823	−2.720838	−1.767303
C	−6.815202	−2.988605	−2.073381	O	−6.336551	3.408664	2.969289
H	3.872677	3.817737	−3.009335	H	6.987813	1.381847	−1.344062
H	2.130586	2.477056	−1.852198	H	4.103287	−3.325995	2.452659
H	2.289619	−1.951142	1.503834	H	7.753771	−0.070635	−0.233141
H	9.472593	−1.491606	0.758712	H	8.848135	−3.387701	2.257842
H	6.459900	−3.835291	2.742788	H	1.093784	0.191363	−2.240061
H	−1.368375	0.224747	−2.084407	H	−1.090564	0.316298	2.218262
H	1.369820	0.331172	2.058446	H	7.313487	3.160741	−2.752885
H	−3.810077	3.835291	3.022261	H	−6.955592	1.372908	1.402272
H	−2.083910	2.504850	1.793098	H	−4.168339	−3.302687	−2.498951
H	−2.307251	−1.917750	−1.570494	H	−7.743580	−0.020122	0.262614
H	−9.516585	−1.438674	−0.683372	H	−8.927593	−3.345045	−2.185847
H	−6.562397	−3.819086	−2.727902	H	−7.245279	3.106081	2.818416

10a* (excited state) b3lyp/6-31+g(d,p) scrf=(cpcm,solvent=acetonitrile)

p opt freq td=(nstates=2)/b3lyp/6-31+g(d,p) scrf=(cpcm,solvent=aceto nitrile)

Charge = 0, Multiplicity = 1, Point group = C1

Electronic Energy = −785.685396 Hartree

Number of imaginary frequencies = 0

Sum of electronic and zero-point Energies = −785.321510 Hartree

Sum of electronic and thermal Energies = −785.306704 Hartree

Sum of electronic and thermal Enthalpies = −785.305760 Hartree

Sum of electronic and thermal Free Energies = −785.362995 Hartree

Atoms	Cartesian Coordinates			Atoms	Cartesian Coordinates		
	X	*Y*	*Z*		*X*	*Y*	*Z*
C	−3.675460	−0.903548	0.000015	C	−3.151687	0.421697	−0.000021
C	−1.756869	0.689127	−0.000021	C	−0.870887	−0.387111	0.000025
C	−1.428069	−1.731713	0.000038	C	−2.814004	−1.990027	0.000041
C	0.566425	−0.532512	0.000025	C	0.839332	−1.921743	0.000015
N	−0.398635	−2.605773	0.000033	C	1.658237	0.408207	0.000030
C	3.005252	−0.159113	−0.000042	C	3.197667	−1.551745	−0.000070
C	2.109671	−2.472312	−0.000033	C	1.507617	1.808517	0.000108
C	2.618722	2.669619	0.000089	C	3.920351	2.129250	−0.000009
C	4.115535	0.757592	−0.000072	O	−4.084725	1.388226	−0.000054

C	−3.703226	2.776541	−0.000061	H	−4.751523	−1.032182	0.000023
H	−1.404953	1.709166	−0.000080	H	−3.190399	−3.007146	0.000061
H	−0.495399	−3.612851	0.000035	H	4.214136	−1.935885	−0.000118
H	2.272180	−3.544326	−0.000046	H	0.515606	2.243469	0.000204
H	2.470448	3.745318	0.000150	H	4.780216	2.795028	−0.000032
H	5.122324	0.347160	−0.000139	H	−4.640387	3.330115	−0.000041
H	−3.127798	3.016188	0.897935	H	−3.127837	3.016187	−0.898083

10b* (excited state) b3lyp/6-31+g(d,p) scrf=(cpcm,solvent=acetonitrile)

p opt freq td=(nstates=2)/b3lyp/6-31+g(d,p) scrf=(cpcm,solvent=aceto nitrile)

Charge = 0, Multiplicity = 1, Point group = C1

Electronic Energy = −824.995557 Hartree

Number of imaginary frequencies = 0

Sum of electronic and zero-point Energies = −824.607410 Hartree

Sum of electronic and thermal Energies = −824.590932 Hartree

Sum of electronic and thermal Enthalpies = −824.589987 Hartree

Sum of electronic and thermal Free Energies = −824.650944 Hartree

Atoms	Cartesian Coordinates			Atoms	Cartesian Coordinates		
	X	*Y*	*Z*		*X*	*Y*	*Z*
C	3.667896	0.522158	−0.000190	C	3.097836	−0.779072	0.000103
C	1.692207	−0.988597	0.000171	C	0.849411	0.119711	−0.000136
C	1.449327	1.442857	−0.000318	C	2.845431	1.640240	−0.000355
C	−0.584419	0.309088	0.000056	C	−0.808963	1.704517	0.000060
N	0.451079	2.362185	−0.000242	C	−1.704093	−0.595145	−0.000110
C	−3.032041	0.015307	0.000238	C	−3.179418	1.414216	0.000405
C	−2.062162	2.297599	0.000280	C	−1.598671	−2.001098	−0.000591
C	−2.735510	−2.825560	−0.000610	C	−4.018834	−2.242621	−0.000067
C	−4.169750	−0.865121	0.000363	C	0.585969	3.806188	−0.000010
O	3.989767	−1.784892	0.000357	C	3.548392	−3.154932	0.000404
H	4.747533	0.615145	−0.000377	H	1.299768	−1.993706	0.000691
H	3.275905	2.634693	−0.000512	H	−4.182850	1.831032	0.000786
H	−2.196103	3.372785	0.000030	H	−0.621212	−2.467500	−0.001046
H	−2.622402	−3.905503	−0.001036	H	−4.900091	−2.879911	0.000036
H	−5.163143	−0.423188	0.000787	H	0.102203	4.223883	−0.888912
H	0.104679	4.223450	0.890437	H	1.637781	4.084131	−0.001454
H	4.460365	−3.749306	0.000292	H	2.963234	−3.370291	0.898381
H	2.962996	−3.370278	−0.897417				

10c* (excited state) b3lyp/6-31+g(d,p) scrf=(cpcm,solvent=acetonitrile)

# p opt freq td=(nstates=2)/b3lyp/6-31+g(d,p) scrf=(cpcm,solvent=aceto nitrile)
Charge = 0, Multiplicity = 1, Point group = C1
Electronic Energy = −1056.066281 Hartree
Number of imaginary frequencies = 0
Sum of electronic and zero-point Energies = −1055.596928 Hartree
Sum of electronic and thermal Energies = −1055.575472 Hartree
Sum of electronic and thermal Enthalpies = −1055.574528 Hartree
Sum of electronic and thermal Free Energies = −1055.648635 Hartree

Atoms	Cartesian Coordinates			Atoms	Cartesian Coordinates		
	X	*Y*	*Z*		*X*	*Y*	*Z*
C	0.065912	3.560170	0.117498	C	1.458850	3.292715	0.077855
C	1.972218	1.969053	0.039636	C	1.076193	0.900404	0.029955
C	−0.340553	1.194729	0.045747	C	−0.841949	2.508251	0.106700
C	1.211437	−0.538439	0.016740	C	−0.096283	−1.078498	0.022996
N	−1.027119	0.011332	0.044460	C	2.348903	−1.420490	−0.027074
C	2.063421	−2.852184	−0.073569	C	0.735418	−3.316658	−0.079354
C	−0.379403	−2.432980	−0.035001	C	3.693109	−0.994012	−0.030428
C	4.756976	−1.909129	−0.075299	C	4.486845	−3.291237	−0.118187
C	3.180692	−3.755327	−0.117506	C	−2.439644	−0.130211	0.016315
C	−3.192995	0.513537	−0.974792	C	−4.580935	0.373351	−0.989234
C	−5.246122	−0.405224	−0.030171	C	−4.471429	−1.043607	0.955157
C	−3.085510	−0.916432	0.983292	C	−6.745633	−0.572342	−0.058874
O	2.244848	4.384992	0.090320	C	3.677450	4.254650	0.066068
H	−0.264871	4.591085	0.163373	H	3.038934	1.808475	0.026244
H	−1.907491	2.698899	0.150437	H	0.558014	−4.387774	−0.119122
H	−1.393060	−2.812639	−0.054249	H	3.923286	0.063236	0.006153
H	5.781449	−1.549160	−0.074923	H	5.310039	−4.001019	−0.152233
H	2.978939	−4.823115	−0.151817	H	−2.694163	1.100809	−1.738442
H	−5.153444	0.871845	−1.766307	H	−4.962069	−1.643167	1.717152
H	−2.505296	−1.402088	1.760307	H	−7.211974	0.128972	−0.755600
H	−7.019295	−1.587484	−0.371051	H	−7.180657	−0.412846	0.933353
H	4.058238	5.274406	0.083047	H	4.029522	3.711041	0.947031
H	4.003123	3.750723	−0.847966				

12* (excited state) b3lyp/6-31+g(d,p) scrf=(cpcm,solvent=acetonitrile)

# p opt freq td=(nstates=2)/b3lyp/6-31+g(d,p) scrf=(cpcm,solvent=aceto nitrile)
Charge = 0, Multiplicity = 1, Point group = C1
Electronic Energy = −5659.764655 Hartree
Number of imaginary frequencies = 0

Sum of electronic and zero-point Energies = −5659.482244 Hartree

Sum of electronic and thermal Energies = −5659.470296 Hartree

Sum of electronic and thermal Enthalpies = −5659.469352 Hartree

Sum of electronic and thermal Free Energies = −5659.522450 Hartree

Atoms	Cartesian Coordinates			Atoms	Cartesian Coordinates		
	X	*Y*	*Z*		*X*	*Y*	*Z*
C	−1.140797	1.920919	−0.000003	C	−0.717813	0.548852	0.000014
C	0.717813	0.548853	0.000016	C	1.140796	1.920919	−0.000004
N	−0.000002	2.698255	−0.000016	C	−2.470780	2.324724	−0.000006
C	−3.463676	1.304237	0.000004	C	−3.055560	−0.029488	0.000034
C	−1.703546	−0.456400	0.000030	C	1.703547	−0.456400	0.000035
C	3.055560	−0.029487	0.000036	C	3.463675	1.304238	0.000002
C	2.470778	2.324725	−0.000008	Br	−4.396598	−1.382936	−0.000011
Br	4.396599	−1.382935	−0.000011	H	−0.000002	3.712356	−0.000026
H	−2.747077	3.373588	−0.000026	H	−4.514841	1.562113	−0.000009
H	−1.454113	−1.510101	0.000033	H	1.454114	−1.510101	0.000048
H	4.514840	1.562115	−0.000018	H	2.747074	3.373589	−0.000027

3a$^{\bullet-}$ (anion radical) b3lyp/6-311+g(d,p) scrf=(cpcm,solvent=acetonitrile)

```
# opt freq b3lyp/6-311+g(d,p)
scrf=(cpcm,solvent=acetonitrile)
```

Charge = -1, Multiplicity = 2, Point group = C1

Electronic Energy = -1017.035909 Hartree

Number of imaginary frequencies = 0

Sum of electronic and zero-point Energies = -1016.707317 Hartree

Sum of electronic and thermal Energies = -1016.687173 Hartree

Sum of electronic and thermal Enthalpies = -1016.686229 Hartree

Sum of electronic and thermal Free Energies = -1016.757888 Hartree

	Cartesian Coordinates				Cartesian Coordinates		
	X	*Y*	*Z*		*X*	*Y*	*Z*
C	-0.256070	3.760392	0.106461	C	1.136314	3.691447	0.099957
C	1.818658	2.471771	0.062622	C	1.078300	1.263850	0.025781
C	-0.350439	1.356396	0.020851	C	-1.015801	2.571693	0.067598
C	1.415641	-0.125662	0.000861	C	0.189253	-0.858449	-0.017666
N	-0.873598	0.057083	-0.002035	C	2.671611	-0.841926	-0.023422
C	2.608309	-2.292429	-0.078980	C	1.358032	-2.951772	-0.108397
C	0.134013	-2.244179	-0.078762	C	3.936523	-0.225069	0.000880
C	5.129299	-0.967202	-0.026411	C	5.070392	-2.365557	-0.079214
C	3.842953	-3.013964	-0.104442	C	-2.253735	-0.276057	-0.004283
C	-3.092349	0.174987	-1.028176	C	-4.447444	-0.149183	-1.021564

C	-5.001730	-0.940413	-0.008612	C	-4.148653	-1.395697	1.004343
C	-2.795982	-1.063935	1.015989	O	1.825876	4.895100	0.134512
C	-6.474906	-1.270100	0.006595	H	-0.743940	4.727181	0.139201
H	2.901199	2.475707	0.066744	H	-2.097776	2.619852	0.073181
H	1.344838	-4.036449	-0.153761	H	-0.811656	-2.771230	-0.102411
H	4.002655	0.853735	0.043489	H	6.085967	-0.456358	-0.006363
H	5.987242	-2.947477	-0.100693	H	3.804318	-4.098986	-0.145843
H	-2.679835	0.773407	-1.831907	H	-5.080706	0.209537	-1.826730
H	-4.548438	-2.012554	1.802922	H	-2.155255	-1.415180	1.816033
H	2.773286	4.717598	0.128037	H	-6.892717	-1.269940	-1.002578
H	-6.658978	-2.249532	0.453979	H	-7.035180	-0.533355	0.592795

3b$^{\bullet-}$ (anion radical) b3lyp/6-311+g(d,p) scrf=(cpcm,solvent=acetonitrile)

```
# opt freq b3lyp/6-311+g(d,p)
scrf=(cpcm,solvent=acetonitrile)
```

Charge = -1, Multiplicity = 2, Point group = C1

Electronic Energy = -1437.331534 Hartree

Number of imaginary frequencies = 0

Sum of electronic and zero-point Energies = -1437.039445 Hartree

Sum of electronic and thermal Energies = -1437.020040 Hartree

Sum of electronic and thermal Enthalpies = -1437.019095 Hartree

Sum of electronic and thermal Free Energies = -1437.088544 Hartree

	Cartesian Coordinates				Cartesian Coordinates		
	X	***Y***	***Z***		***X***	***Y***	***Z***
C	-0.263160	3.814252	0.118382	C	-1.649227	3.664715	0.107088
C	-2.258770	2.407684	0.064237	C	-1.449497	1.244891	0.024722
C	-0.029170	1.420321	0.019985	C	0.563601	2.671653	0.075840
C	-1.708493	-0.161041	0.001319	C	-0.444446	-0.826214	-0.015768
N	0.568600	0.149612	-0.001013	C	-2.923636	-0.944675	-0.025757
C	-2.782298	-2.388919	-0.083821	C	-1.497665	-2.979444	-0.115736
C	-0.314907	-2.205926	-0.083173	C	-4.220430	-0.397641	-0.002584
C	-5.370345	-1.203520	-0.032713	C	-5.235898	-2.596303	-0.086433
C	-3.974722	-3.176629	-0.110746	C	1.959101	-0.104586	-0.002254
C	2.528178	-0.944360	0.962994	C	3.895912	-1.203453	0.961968
C	4.698850	-0.604829	-0.003867	C	4.158844	0.239102	-0.968482
C	2.787382	0.479708	-0.968474	O	-2.406604	4.825580	0.143740
Cl	6.436185	-0.922696	-0.004682	H	0.168313	4.807162	0.157044
H	-3.339527	2.348223	0.067248	H	1.640292	2.785923	0.088291
H	-1.424584	-4.061372	-0.165252	H	0.656130	-2.683798	-0.112398
H	-4.346042	0.675652	0.041324	H	-6.353303	-0.745303	-0.013810

H	-6.119639	-3.227155	-0.109688	H	-3.876653	-4.257775	-0.153563
H	1.898896	-1.391110	1.722239	H	4.330166	-1.851704	1.712337
H	4.793422	0.692789	-1.719023	H	2.357170	1.118935	-1.729117
H	-3.342391	4.594717	0.130775				

3c$^{\bullet-}$ (anion radical) b3lyp/6-311+g(d,p) scrf=(cpcm,solvent=acetonitrile)

```
# opt freq b3lyp/6-311+g(d,p)
scrf=(cpcm,solvent=acetonitrile)
```

Charge = -1, Multiplicity = 2, Point group = C1

Electronic Energy = -1056.361190 Hartree

Number of imaginary frequencies = 0

Sum of electronic and zero-point Energies = -1056.005340 Hartree

Sum of electronic and thermal Energies = -1055.983409 Hartree

Sum of electronic and thermal Enthalpies = -1055.982464 Hartree

Sum of electronic and thermal Free Energies = -1056.057861 Hartree

	Cartesian Coordinates				Cartesian Coordinates		
	X	***Y***	***Z***		***X***	***Y***	***Z***
C	0.430723	3.521098	0.075732	C	-0.969359	3.516946	0.071900
C	-1.724031	2.339859	0.043369	C	-1.065301	1.089203	0.011750
C	0.362909	1.099685	0.003610	C	1.097021	2.276236	0.042444
C	-1.483703	-0.281204	-0.004272	C	-0.301446	-1.081418	-0.020696
N	0.812418	-0.227159	-0.012594	C	-2.778791	-0.921179	-0.022520
C	-2.800048	-2.373770	-0.070453	C	-1.590020	-3.103482	-0.097980
C	-0.325323	-2.468770	-0.074235	C	-4.004937	-0.230128	0.001179
C	-5.241101	-0.902208	-0.019904	C	-5.263914	-2.300826	-0.065987
C	-4.075981	-3.021873	-0.090439	C	2.170726	-0.639557	-0.010094
C	2.665677	-1.447253	1.019097	C	3.998093	-1.852157	1.016152
C	4.877834	-1.455916	0.000843	C	4.373322	-0.637921	-1.016554
C	3.037692	-0.240384	-1.031705	O	-1.595143	4.756327	0.100094
C	6.315117	-1.917834	-0.007689	C	1.201129	4.817609	0.110781
H	-2.804492	2.413713	0.050687	H	2.180738	2.255180	0.045755
H	-1.639751	-4.187503	-0.137383	H	0.587972	-3.050131	-0.096140
H	-4.006525	0.851049	0.038613	H	-6.166258	-0.336120	-0.000281
H	-6.213460	-2.828196	-0.082844	H	-4.101812	-4.107502	-0.126604
H	2.005246	-1.750478	1.822775	H	4.362689	-2.476384	1.825826
H	5.030757	-0.311901	-1.816218	H	2.664303	0.381221	-1.836978
H	-2.550120	4.626199	0.092321	H	6.704042	-2.026131	1.007546
H	6.410402	-2.892788	-0.498301	H	6.955344	-1.216557	-0.547179
H	0.978874	5.449908	-0.756131	H	0.962247	5.412319	0.999392
H	2.276296	4.625073	0.116576				

3d•⁻ (anion radical) b3lyp/6-311+g(d,p) scrf=(cpcm,solvent=acetonitrile)

```
# opt freq b3lyp/6-311+g(d,p)
scrf=(cpcm,solvent=acetonitrile)
```

Charge = -1, Multiplicity = 2, Point group = C1

Electronic Energy = -3590.583891 Hartree

Number of imaginary frequencies = 0

Sum of electronic and zero-point Energies = -3590.265566 Hartree

Sum of electronic and thermal Energies = -3590.243721 Hartree

Sum of electronic and thermal Enthalpies = -3590.242777 Hartree

Sum of electronic and thermal Free Energies = -3590.319544 Hartree

	Cartesian Coordinates				Cartesian Coordinates		
	X	***Y***	***Z***		***X***	***Y***	***Z***
C	-1.412345	3.758523	0.105426	C	-0.018924	3.698916	0.098265
C	0.667896	2.482023	0.061610	C	-0.066176	1.273980	0.025904
C	-1.493581	1.356764	0.022411	C	-2.165178	2.569658	0.068310
C	0.278066	-0.116936	0.000674	C	-0.945373	-0.853796	-0.016405
N	-2.010606	0.055767	0.000038	C	1.533945	-0.824191	-0.024021
C	1.478773	-2.275305	-0.076804	C	0.233586	-2.938250	-0.103782
C	-0.994579	-2.240133	-0.074470	C	2.791781	-0.190717	-0.001873
C	3.970172	-0.952070	-0.030386	C	3.948093	-2.342750	-0.080473
C	2.718087	-2.991397	-0.102414	C	-3.390378	-0.283800	-0.003378
C	-4.224845	0.147808	-1.038454	C	-5.578554	-0.181992	-1.032974
C	-6.133927	-0.959403	-0.009918	C	-5.284082	-1.395165	1.014244
C	-3.932863	-1.057522	1.026951	Br	5.674085	-0.028741	0.000248
O	0.661791	4.905776	0.131645	C	-7.605683	-1.295136	0.003473
H	-1.905006	4.722886	0.138027	H	1.750297	2.491820	0.064923
H	-3.247325	2.612484	0.074750	H	0.226452	-4.022921	-0.147284
H	-1.936783	-2.772834	-0.096370	H	2.861229	0.885148	0.038842
H	4.869262	-2.912818	-0.102134	H	2.691861	-4.075942	-0.141802
H	-3.810643	0.735261	-1.849429	H	-6.209644	0.161193	-1.846493
H	-5.685160	-2.001180	1.820354	H	-3.294061	-1.393253	1.835197
H	1.610822	4.736800	0.124631	H	-8.017500	-1.317480	-1.007886
H	-7.788191	-2.265817	0.470034	H	-8.172155	-0.548650	0.571039

3e•⁻ (anion radical) b3lyp/6-311+g(d,p) scrf=(cpcm,solvent=acetonitrile)

```
# opt freq b3lyp/6-311+g(d,p)
scrf=(cpcm,solvent=acetonitrile)
```

Charge = -1, Multiplicity = 2, Point group = C1

Electronic Energy = -1109.328367 Hartree

Number of imaginary frequencies = 0

Sum of electronic and zero-point Energies = -1109.000056 Hartree

Sum of electronic and thermal Energies = -1108.978209 Hartree

Sum of electronic and thermal Enthalpies = -1108.977265 Hartree

Sum of electronic and thermal Free Energies = -1109.053230 Hartree

	**Cartesian Coordinates**				**Cartesian Coordinates**		
	X	***Y***	***Z***		***X***	***Y***	***Z***
C	-0.731139	3.755168	0.100056	C	0.666737	3.697286	0.092980
C	1.348152	2.481332	0.059376	C	0.612581	1.278639	0.026429
C	-0.811423	1.359864	0.024243	C	-1.482851	2.575854	0.066955
C	0.955440	-0.118438	0.002345	C	-0.265780	-0.840906	-0.012352
N	-1.328934	0.062295	0.003048	C	2.211488	-0.829923	-0.021975
C	2.134161	-2.281396	-0.068977	C	0.891696	-2.930508	-0.091230
C	-0.328010	-2.236854	-0.064340	C	3.466813	-0.215020	-0.004862
C	4.677530	-0.976401	-0.031777	C	4.589875	-2.400177	-0.077254
C	3.370965	-3.021262	-0.094652	C	-2.711915	-0.277343	-0.001843
C	-3.528959	0.104866	-1.068683	C	-4.882752	-0.223354	-1.065565
C	-5.452307	-0.949110	-0.012396	C	-4.618143	-1.334902	1.043810
C	-3.266505	-0.998073	1.058439	O	1.339771	4.905029	0.123704
C	-6.924529	-1.282172	-0.003658	C	5.927981	-0.342150	-0.013908
N	6.969820	0.192016	0.000918	H	-1.221290	4.720860	0.130721
H	2.430587	2.485229	0.062378	H	-2.565022	2.619436	0.073847
H	0.879740	-4.015771	-0.130784	H	-1.273554	-2.762410	-0.084076
H	3.545785	0.861644	0.030679	H	5.501767	-2.987563	-0.098187
H	3.320575	-4.105265	-0.129773	H	-3.101531	0.651630	-1.901084
H	-5.502646	0.079831	-1.903069	H	-5.031321	-1.900758	1.872466
H	-2.638323	-1.293854	1.890403	H	2.290464	4.744652	0.116101
H	-7.312120	-1.397077	-1.018338	H	-7.120238	-2.205435	0.545927
H	-7.501877	-0.485686	0.478409				

6a$^{\bullet-}$ (anion radical) b3lyp/6-311+g(d,p) scrf=(cpcm,solvent=acetonitrile)

```
# opt freq b3lyp/6-311+g(d,p)
scrf=(cpcm,solvent=acetonitrile)
```

Charge = -1, Multiplicity = 2, Point group = C1

Electronic Energy = -1591.627788 Hartree

Number of imaginary frequencies = 0

Sum of electronic and zero-point Energies = -1591.136224 Hartree

Sum of electronic and thermal Energies = -1591.104797 Hartree

Sum of electronic and thermal Enthalpies = -1591.103853 Hartree

Sum of electronic and thermal Free Energies = -1591.200971 Hartree

	Cartesian Coordinates				Cartesian Coordinates		
	X	*Y*	*Z*		*X*	*Y*	*Z*
C	-0.567288	-3.119620	0.555544	C	0.623772	-2.421744	0.717311
C	0.720236	-1.011146	0.595235	C	-0.487002	-0.303075	0.231847
C	-1.713159	-1.050476	0.184251	C	-1.769985	-2.427497	0.313412
C	-0.827456	1.061236	-0.100760	C	-2.250525	1.115284	-0.224031
N	-2.765923	-0.170393	-0.066962	C	-0.087713	2.275814	-0.397872
C	-0.841688	3.498849	-0.589066	C	-2.258344	3.487079	-0.562376
C	-2.968553	2.292115	-0.420269	C	1.309185	2.334082	-0.585657
C	1.972038	3.521738	-0.883410	C	1.245800	4.720092	-0.998028
C	-0.127739	4.703637	-0.855317	C	-4.138822	-0.532418	-0.157419
C	-4.800506	-1.079296	0.945153	C	-6.141838	-1.442714	0.848521
C	-6.861719	-1.257684	-0.338037	C	-6.186421	-0.706471	-1.433889
C	-4.841601	-0.352820	-1.351936	O	1.711484	-3.185868	1.099889
C	-8.325342	-1.616475	-0.425818	C	1.952067	-0.338027	1.067352
C	3.246159	-0.554889	0.466554	C	4.417908	0.041590	1.050684
C	4.275007	0.881939	2.185858	C	3.029795	1.130465	2.720002
C	1.884900	0.526345	2.167034	C	3.404544	-1.311450	-0.719710
C	4.653906	-1.527394	-1.282419	C	5.809550	-0.984024	-0.681784
C	5.676086	-0.210798	0.455714	O	0.702198	0.792199	2.805532
O	4.674698	-2.273077	-2.432080	C	5.927701	-2.512911	-3.073760
H	-0.564066	-4.195175	0.687184	H	-2.708811	-2.961369	0.239342
H	-2.792041	4.420087	-0.709731	H	-4.050773	2.279199	-0.464205
H	1.885186	1.425030	-0.511765	H	3.047688	3.518257	-1.022434
H	1.759524	5.651791	-1.212777	H	-0.697575	5.620912	-0.972463
H	-4.262393	-1.220340	1.875195	H	-6.637622	-1.870630	1.713873
H	-6.715457	-0.561675	-2.370347	H	-4.331466	0.058763	-2.214884
H	2.498504	-2.626920	1.137685	H	-8.574190	-2.440579	0.246358
H	-8.953994	-0.764263	-0.145175	H	-8.603448	-1.905640	-1.441774
H	5.158509	1.332818	2.624707	H	2.907681	1.770830	3.586311
H	2.534870	-1.723204	-1.217315	H	6.790640	-1.149433	-1.105369
H	6.561083	0.225121	0.908074	H	-0.024240	0.426042	2.275909
H	5.699162	-3.109461	-3.955027	H	6.402627	-1.576915	-3.382625
H	6.607311	-3.072541	-2.424219				

6b•⁻ (anion radical) b3lyp/6-311+g(d,p) scrf=(cpcm,solvent=acetonitrile)

```
# opt freq b3lyp/6-311+g(d,p)
scrf=(cpcm,solvent=acetonitrile)
```

Charge = -1, Multiplicity = 2, Point group = C1

Electronic Energy = -6624.160974 Hartree

Number of imaginary frequencies = 0

Sum of electronic and zero-point Energies = -6623.722499 Hartree

Sum of electronic and thermal Energies = -6623.690568 Hartree

Sum of electronic and thermal Enthalpies = -6623.689623 Hartree

Sum of electronic and thermal Free Energies = -6623.789584 Hartree

	**Cartesian Coordinates**				**Cartesian Coordinates**		
	X	***Y***	***Z***		***X***	***Y***	***Z***
C	-1.507091	-3.222124	0.820581	C	-0.286471	-2.599545	1.060769
C	-0.090532	-1.199475	0.926838	C	-1.218839	-0.433514	0.455213
C	-2.481205	-1.099620	0.325334	C	-2.638754	-2.468813	0.476150
C	-1.449661	0.945671	0.065932	C	-2.849892	1.075138	-0.166920
N	-3.453879	-0.165331	-0.021806	C	-0.625875	2.103214	-0.210267
C	-1.288561	3.351731	-0.516012	C	-2.702773	3.422019	-0.597855
C	-3.484533	2.285721	-0.459990	C	0.787085	2.073138	-0.282746
C	1.501991	3.213303	-0.600161	C	0.879753	4.447877	-0.836163
C	-0.496847	4.500171	-0.789109	C	-4.837982	-0.447502	-0.216777
C	-5.632495	-0.850187	0.858328	C	-6.981751	-1.135345	0.660968
C	-7.572599	-1.013423	-0.602497	C	-6.762168	-0.607503	-1.669935
C	-5.409234	-0.332358	-1.485991	O	0.711751	-3.424155	1.537930
C	-9.043404	-1.285971	-0.803865	C	1.139993	-0.597867	1.478482
C	2.460651	-0.947155	1.010184	C	3.626161	-0.463851	1.708563
C	3.454911	0.410173	2.805447	C	2.184441	0.811492	3.198649
C	1.050187	0.312405	2.551002	C	2.655951	-1.728684	-0.158714
C	3.935085	-2.070317	-0.565936	C	5.076476	-1.662423	0.135961
C	4.912273	-0.863681	1.253459	Br	4.155968	-3.123769	-2.170159
O	-0.160389	0.698118	3.068438	Br	3.428348	3.118997	-0.732494
H	-1.581411	-4.292364	0.973725	H	-3.601943	-2.943445	0.340322
H	-3.165570	4.376965	-0.820869	H	-4.559852	2.328825	-0.580701
H	1.308978	1.145618	-0.112207	H	1.463372	5.330680	-1.063676
H	-1.001382	5.438922	-0.993826	H	-5.192348	-0.940220	1.844597
H	-7.585058	-1.451827	1.505495	H	-7.191154	-0.514846	-2.662322
H	-4.792362	-0.032823	-2.325201	H	1.522855	-2.911692	1.654735
H	-9.417978	-2.006910	-0.074176	H	-9.629093	-0.367531	-0.687755
H	-9.242296	-1.674454	-1.805171	H	4.328998	0.780390	3.329714
H	2.045829	1.481196	4.039948	H	1.796609	-2.041313	-0.737140
H	6.064711	-1.952435	-0.197305	H	5.786433	-0.522136	1.798065
H	-0.867898	0.365886	2.494763				

$7^{\bullet -}$ (anion radical) b3lyp/6-311+g(d,p) scrf=(cpcm,solvent=acetonitrile)

```
# opt freq b3lyp/6-311+g(d,p)
scrf=(cpcm,solvent=acetonitrile)
```

Charge = -1, Multiplicity = 2, Point group = C1

Electronic Energy = -1723.030718 Hartree

Number of imaginary frequencies = 0

Sum of electronic and zero-point Energies = -1722.522889 Hartree

Sum of electronic and thermal Energies = -1722.491139 Hartree

Sum of electronic and thermal Enthalpies = -1722.490194 Hartree

Sum of electronic and thermal Free Energies = -1722.588467 Hartree

	**Cartesian Coordinates**				**Cartesian Coordinates**		
	X	***Y***	***Z***		***X***	***Y***	***Z***
C	4.183578	-3.281151	1.921127	C	5.529366	-2.940323	1.792981
C	5.938758	-1.767620	1.152494	C	4.961440	-0.890706	0.618761
C	3.584961	-1.248994	0.777278	C	3.192308	-2.421519	1.402861
C	5.003975	0.342367	-0.103470	C	3.654293	0.721336	-0.378704
N	2.800637	-0.258881	0.161192	C	6.085260	1.196941	-0.541610
C	5.726328	2.421643	-1.234394	C	4.367002	2.744171	-1.454809
C	3.316841	1.899078	-1.029849	C	7.449646	0.917141	-0.337091
C	8.462921	1.782200	-0.781352	C	8.118525	2.962320	-1.451610
C	6.784299	3.275931	-1.674247	C	1.390183	-0.259269	0.083358
C	0.613970	-0.409420	1.240175	C	-0.774743	-0.426679	1.161093
C	-1.409762	-0.263896	-0.071560	C	-0.642430	-0.100049	-1.227171
C	0.746035	-0.110962	-1.152133	O	6.454152	-3.822701	2.329908
N	-2.835266	-0.269305	-0.152452	C	-4.173825	-3.247696	-1.952784
C	-5.536179	-2.921307	-1.837517	C	-5.950056	-1.765721	-1.186764
C	-4.982205	-0.904711	-0.636860	C	-3.610788	-1.247841	-0.771988
C	-3.200970	-2.413792	-1.419712	C	-5.025200	0.340792	0.100421
C	-3.687673	0.688956	0.373052	C	-6.078690	1.200542	0.547927
C	-5.712761	2.394400	1.256581	C	-4.335701	2.695315	1.491366
C	-3.330251	1.868585	1.063463	C	-7.458304	0.949160	0.336569
C	-8.424284	1.821113	0.795876	C	-8.062200	2.992812	1.492044
C	-6.730430	3.267784	1.714622	O	-6.427273	-3.807903	-2.400344
H	3.909432	-4.202697	2.420194	H	6.997156	-1.555654	1.071949
H	2.147284	-2.687905	1.498208	H	4.129665	3.669761	-1.969874
H	2.284962	2.172694	-1.209439	H	7.737135	0.009491	0.175722
H	9.503648	1.532651	-0.604896	H	8.895703	3.636632	-1.799155
H	6.522745	4.193384	-2.194027	H	1.099921	-0.512243	2.202118
H	-1.368112	-0.556765	2.058403	H	-1.133608	0.026099	-2.184748
H	1.336530	-0.003779	-2.053050	H	7.343342	-3.483842	2.176062
H	-3.895924	-4.161437	-2.464138	H	-7.008575	-1.551365	-1.114307

H	-2.152082	-2.667840	-1.507749	H	-4.092291	3.608431	2.023701
H	-2.290723	2.110337	1.245825	H	-7.767978	0.058909	-0.192134
H	-9.471514	1.601815	0.619614	H	-8.828862	3.671051	1.848562
H	-6.439720	4.166514	2.248914	H	-7.330189	-3.497683	-2.263986

10a•⁻ (anion radical) b3lyp/6-311+g(d,p) scrf=(cpcm,solvent=acetonitrile)

```
# opt freq b3lyp/6-311+g(d,p)
scrf=(cpcm,solvent=acetonitrile)
```

Charge = -1, Multiplicity = 2, Point group = C1

Electronic Energy = -785.912844 Hartree

Number of imaginary frequencies = 0

Sum of electronic and zero-point Energies = -785.663396 Hartree

Sum of electronic and thermal Energies = -785.648421 Hartree

Sum of electronic and thermal Enthalpies = -785.647477 Hartree

Sum of electronic and thermal Free Energies = -785.705727 Hartree

	Cartesian Coordinates				Cartesian Coordinates		
	X	*Y*	*Z*		*X*	*Y*	*Z*
C	-3.655158	-0.903645	-0.000034	C	-3.153953	0.404233	-0.000061
C	-1.778970	0.665342	-0.000043	C	-0.869687	-0.424520	0.000004
C	-1.407459	-1.750051	0.000021	C	-2.772412	-1.999593	0.000002
C	0.559287	-0.548593	0.000021	C	0.857554	-1.946259	0.000039
N	-0.344601	-2.641945	0.000055	C	1.646145	0.405858	0.000014
C	2.998930	-0.129596	-0.000024	C	3.218001	-1.525675	-0.000020
C	2.150258	-2.454577	0.000019	C	1.474822	1.802132	0.000050
C	2.566003	2.689470	0.000027	C	3.869502	2.176628	-0.000032
C	4.084066	0.805027	-0.000058	O	-4.115345	1.400712	-0.000131
C	-3.687140	2.757422	0.000111	H	-4.727929	-1.056052	-0.000040
H	-1.419946	1.682125	-0.000082	H	-3.156589	-3.013953	0.000018
H	-0.430506	-3.645198	0.000028	H	4.241400	-1.888704	-0.000042
H	2.334490	-3.523416	0.000030	H	0.474728	2.215265	0.000107
H	2.394619	3.760567	0.000058	H	4.718993	2.853738	-0.000059
H	5.098530	0.415788	-0.000100	H	-4.595187	3.359116	0.000161
H	-3.098228	2.993362	0.892851	H	-3.098140	2.993661	-0.892493

10b•⁻ (anion radical) b3lyp/6-311+g(d,p) scrf=(cpcm,solvent=acetonitrile)

```
# opt freq b3lyp/6-311+g(d,p)
scrf=(cpcm,solvent=acetonitrile)
```

Charge = -1, Multiplicity = 2, Point group = C1

Electronic Energy = -825.230071 Hartree

Number of imaginary frequencies = 0

Sum of electronic and zero-point Energies = -824.952656 Hartree

Sum of electronic and thermal Energies = -824.936109 Hartree

Sum of electronic and thermal Enthalpies = -824.935165 Hartree

Sum of electronic and thermal Free Energies = -824.997146 Hartree

	Cartesian Coordinates				Cartesian Coordinates		
	X	*Y*	*Z*		*X*	*Y*	*Z*
C	3.645298	0.517933	0.011911	C	3.095550	-0.769697	0.004579
C	1.711615	-0.976874	-0.007805	C	0.844842	0.146514	-0.014624
C	1.429564	1.453125	-0.018009	C	2.804842	1.647173	0.003118
C	-0.575430	0.323097	-0.015173	C	-0.818884	1.733110	-0.017422
N	0.406368	2.394096	-0.047506	C	-1.694711	-0.591850	-0.008873
C	-3.027597	-0.010493	0.007079	C	-3.195446	1.392508	0.015473
C	-2.095563	2.283248	0.005359	C	-1.573501	-1.994012	-0.015681
C	-2.695262	-2.841598	-0.006788	C	-3.979987	-2.283670	0.009182
C	-4.145107	-0.905004	0.016063	C	0.588034	3.827067	0.024565
O	4.017695	-1.802456	0.014290	C	3.537850	-3.141756	0.008408
H	4.722813	0.630758	0.028428	H	1.313942	-1.979128	-0.008296
H	3.236197	2.641366	0.016303	H	-4.204606	1.793034	0.033198
H	-2.253404	3.355141	0.019887	H	-0.589232	-2.443017	-0.029348
H	-2.561675	-3.918078	-0.012770	H	-4.852827	-2.930251	0.016076
H	-5.144812	-0.479425	0.028347	H	-0.243895	4.329456	-0.470796
H	0.645539	4.185640	1.059868	H	1.506415	4.110655	-0.492004
H	4.422176	-3.777758	0.015743	H	2.932168	-3.356276	0.895329
H	2.948419	-3.354079	-0.889910				

10c$^{\bullet-}$ (anion radical) b3lyp/6-311+g(d,p) scrf=(cpcm,solvent=acetonitrile)

```
# opt freq b3lyp/6-311+g(d,p)
scrf=(cpcm,solvent=acetonitrile)
```

Charge = -1, Multiplicity = 2, Point group = C1

Electronic Energy = -1056.342045 Hartree

Number of imaginary frequencies = 0

Sum of electronic and zero-point Energies = -1055.985113 Hartree

Sum of electronic and thermal Energies = -1055.963649 Hartree

Sum of electronic and thermal Enthalpies = -1055.962705 Hartree

Sum of electronic and thermal Free Energies = -1056.037558 Hartree

	Cartesian Coordinates				Cartesian Coordinates		
	X	*Y*	*Z*		*X*	*Y*	*Z*
C	0.124532	3.551642	0.085033	C	1.498454	3.282162	0.075632
C	1.987479	1.972046	0.043365	C	1.068532	0.890712	0.014740
C	-0.328091	1.191906	0.013412	C	-0.803537	2.494774	0.054802

C	1.197451	-0.534897	-0.002572	C	-0.123980	-1.078038	-0.010773
N	-1.039369	-0.014711	0.002110	C	2.333615	-1.429870	-0.029184
C	2.054904	-2.855831	-0.071446	C	0.721086	-3.322190	-0.088468
C	-0.385160	-2.440843	-0.059842	C	3.676274	-1.008432	-0.020091
C	4.746230	-1.919913	-0.049811	C	4.479626	-3.294034	-0.088093
C	3.169133	-3.753172	-0.098094	C	-2.453578	-0.137777	-0.001368
C	-3.214477	0.435206	-1.025104	C	-4.603001	0.318840	-1.019148
C	-5.270643	-0.380909	-0.007370	C	-4.496095	-0.960806	1.005147
C	-3.108939	-0.836557	1.017569	C	-6.776721	-0.484614	0.007496
O	2.316502	4.397391	0.102210	C	3.726705	4.206437	0.102540
H	-0.211111	4.581368	0.113645	H	3.050151	1.790866	0.045234
H	-1.865962	2.705595	0.063610	H	0.546986	-4.393338	-0.124330
H	-1.398532	-2.822077	-0.075289	H	3.903056	0.048666	0.011154
H	5.768192	-1.556412	-0.042011	H	5.299489	-4.006222	-0.110431
H	2.969434	-4.820739	-0.128957	H	-2.716468	0.965508	-1.828237
H	-5.174557	0.769911	-1.824040	H	-4.984445	-1.511188	1.803066
H	-2.528867	-1.281138	1.817447	H	-7.190419	-0.407823	-1.000429
H	-7.106468	-1.430950	0.442409	H	-7.218931	0.320218	0.605016
H	4.164589	5.203499	0.126194	H	4.056374	3.646660	0.984177
H	4.062256	3.686397	-0.800913				

12$^{\bullet-}$ (anion radical) b3lyp/6-311+g(d,p) scrf=(cpcm,solvent=acetonitrile)

```
# opt freq b3lyp/6-311+g(d,p)
scrf=(cpcm,solvent=acetonitrile)
```

Charge = -1, Multiplicity = 2, Point group = C1

Electronic Energy = -5664.765214 Hartree

Number of imaginary frequencies = 0

Sum of electronic and zero-point Energies = -5664.615207 Hartree

Sum of electronic and thermal Energies = -5664.602423 Hartree

Sum of electronic and thermal Enthalpies = -5664.601479 Hartree

Sum of electronic and thermal Free Energies = -5664.656840 Hartree

	**Cartesian Coordinates**				**Cartesian Coordinates**		
	X	***Y***	***Z***		***X***	***Y***	***Z***
C	-1.139050	1.967356	0.000002	C	-0.708817	0.599588	0.000003
C	0.708817	0.599588	0.000002	C	1.139050	1.967356	-0.000000
N	-0.000000	2.762704	0.000002	C	-2.473561	2.335457	0.000001
C	-3.456290	1.300983	0.000001	C	-3.032467	-0.025044	0.000000
C	-1.699708	-0.430327	0.000001	C	1.699708	-0.430327	0.000004
C	3.032467	-0.025044	0.000000	C	3.456290	1.300983	-0.000005
C	2.473561	2.335457	-0.000004	Br	-4.400362	-1.414012	-0.000001

Br	4.400362	-1.414012	0.000000	H	-0.000000	3.770252	-0.000004
H	-2.775161	3.376824	-0.000000	H	-4.509151	1.548289	0.000003
H	-1.427376	-1.477366	-0.000002	H	1.427376	-1.477366	0.000014
H	4.509151	1.548289	-0.000012	H	2.775161	3.376825	-0.000005

3a$^{\bullet-}$ (anion radical) b3lyp/6-31+g(d,p) scrf=(cpcm,solvent=acetonitrile)

opt freq b3lyp/6-31+g(d,p) scrf=(cpcm,solvent=acetonitrile)

Charge = −1, Multiplicity = 2, Point group = C1

Electronic Energy = −1016.841803 Hartree

Number of imaginary frequencies = 0

Sum of electronic and zero-point Energies = −1016.512147 Hartree

Sum of electronic and thermal Energies = −1016.492016 Hartree

Sum of electronic and thermal Enthalpies = −1016.491071 Hartree

Sum of electronic and thermal Free Energies = −1016.563244 Hartree

Atoms	Cartesian Coordinates			Atoms	Cartesian Coordinates		
	X	*Y*	*Z*		*X*	*Y*	*Z*
C	−0.259658	3.766685	0.107973	C	1.136284	3.697759	0.100742
C	1.821335	2.476289	0.062106	C	1.080043	1.265918	0.025334
C	−0.350929	1.357854	0.020331	C	−1.019714	2.575082	0.068607
C	1.417988	−0.125443	−0.000404	C	0.189542	−0.858992	−0.019608
N	−0.874720	0.057250	−0.003382	C	2.675963	−0.842425	−0.023678
C	2.612486	−2.295369	−0.080524	C	1.359587	−2.956187	−0.111976
C	0.133018	−2.247722	−0.081982	C	3.943228	−0.223878	0.002919
C	5.138926	−0.967786	−0.023464	C	5.080138	−2.369118	−0.077850
C	3.848867	−3.018511	−0.105144	C	−2.254764	−0.276338	−0.005353
C	−3.097168	0.181227	−1.027727	C	−4.455886	−0.144053	−1.019775
C	−5.009428	−0.942838	−0.007947	C	−4.151874	−1.404624	1.003671
C	−2.796286	−1.071862	1.014814	O	1.824811	4.903735	0.135241
C	−6.483732	−1.275336	0.009158	H	−0.749265	4.734703	0.141602
H	2.905700	2.479593	0.065250	H	−2.103646	2.622015	0.074355
H	1.346554	−4.042863	−0.158460	H	−0.814231	−2.775902	−0.106731
H	4.008849	0.856789	0.047075	H	6.096938	−0.455104	−0.001520
H	5.998220	−2.952846	−0.098772	H	3.809281	−4.105488	−0.147642
H	−2.685761	0.785598	−1.830138	H	−5.092208	0.220487	−1.822461
H	−4.550806	−2.027095	1.801003	H	−2.152643	−1.427981	1.812953
H	2.776288	4.730326	0.128531	H	−6.922609	−1.191311	−0.989392
H	−6.659786	−2.291348	0.376353	H	−7.034117	−0.592196	0.668399

3b$^{\bullet-}$ (anion radical) b3lyp/6-31+g(d,p) scrf=(cpcm,solvent=acetonitrile)

# opt freq b3lyp/6-31+g(d,p) scrf=(cpcm,solvent=acetonitrile)
Charge = −1, Multiplicity = 2, Point group = C1
Electronic Energy = −1437.115663 Hartree
Number of imaginary frequencies = 0
Sum of electronic and zero-point Energies = −1436.822627 Hartree
Sum of electronic and thermal Energies = −1436.803257 Hartree
Sum of electronic and thermal Enthalpies = −1436.802313 Hartree
Sum of electronic and thermal Free Energies = −1436.871682 Hartree

Atoms	Cartesian Coordinates			Atoms	Cartesian Coordinates		
	X	*Y*	*Z*		*X*	*Y*	*Z*
C	−0.259105	3.820383	0.119616	C	−1.648747	3.671144	0.107978
C	−2.261218	2.412422	0.063852	C	−1.451249	1.246956	0.024434
C	−0.028436	1.421274	0.018684	C	0.567794	2.674764	0.075753
C	−1.711336	−0.160824	0.000554	C	−0.445187	−0.827009	−0.017956
N	0.569537	0.149193	−0.003599	C	−2.928834	−0.944696	−0.025232
C	−2.787944	−2.391327	−0.085912	C	−1.500811	−2.983686	−0.120524
C	−0.315314	−2.209807	−0.087046	C	−4.227907	−0.395596	0.000930
C	−5.380936	−1.202975	−0.028434	C	−5.247068	−2.598733	−0.084744
C	−3.982269	−3.180325	−0.112013	C	1.959937	−0.105545	−0.003513
C	2.528054	−0.955945	0.958312	C	3.898946	−1.216205	0.958126
C	4.707237	−0.607707	−0.002762	C	4.167666	0.247085	−0.963731
C	2.792883	0.488106	−0.965335	O	−2.404873	4.834590	0.144238
Cl	6.444252	−0.925898	−0.002020	H	0.174342	4.814476	0.159443
H	−3.343763	2.352713	0.066020	H	1.646503	2.787987	0.088482
H	−1.428201	−4.067604	−0.172108	H	0.656913	−2.689563	−0.118400
H	−4.352713	0.679564	0.047094	H	−6.365182	−0.742680	−0.006894
H	−6.132244	−3.231039	−0.107618	H	−3.883560	−4.263371	−0.156798
H	1.895736	−1.410342	1.713220	H	4.331375	−1.872940	1.705079
H	4.804943	0.708787	−1.710004	H	2.363852	1.135071	−1.722831
H	−3.345003	4.607610	0.134719				

3c$^{\bullet-}$ (anion radical) b3lyp/6-31+g(d,p) scrf=(cpcm,solvent=acetonitrile)

# opt freq b3lyp/6-31+g(d,p) scrf=(cpcm,solvent=acetonitrile)
Charge = −1, Multiplicity = 2, Point group = C1
Electronic Energy = −1056.160525 Hartree
Number of imaginary frequencies = 0
Sum of electronic and zero-point Energies = −1055.803508 Hartree
Sum of electronic and thermal Energies = −1055.781586 Hartree
Sum of electronic and thermal Enthalpies = −1055.780641 Hartree

Sum of electronic and thermal Free Energies = −1055.856871 Hartree

Atoms	Cartesian Coordinates			Atoms	Cartesian Coordinates		
	X	*Y*	*Z*		*X*	*Y*	*Z*
C	0.437784	3.525851	0.075820	C	−0.965750	3.523278	0.073456
C	−1.724501	2.345478	0.044024	C	−1.066268	1.091522	0.010529
C	0.364181	1.099928	−0.000235	C	1.102801	2.277389	0.039412
C	−1.486667	−0.280093	−0.006871	C	−0.303187	−1.082408	−0.027076
N	0.813035	−0.228693	−0.019660	C	−2.784512	−0.919375	−0.022140
C	−2.807376	−2.374269	−0.073104	C	−1.595612	−3.106873	−0.105889
C	−0.327665	−2.472747	−0.083648	C	−4.012127	−0.225242	0.007419
C	−5.251928	−0.897499	−0.011365	C	−5.276654	−2.299068	−0.061178
C	−4.085784	−3.022472	−0.090973	C	2.170754	−0.642540	−0.014681
C	2.660967	−1.462696	1.012882	C	3.995592	−1.869578	1.013076
C	4.883937	−1.463340	0.003328	C	4.384119	−0.634626	−1.011546
C	3.044946	−0.234548	−1.030709	O	−1.589030	4.765514	0.103732
C	6.323768	−1.923140	0.005346	C	1.211018	4.822118	0.111905
H	−2.806735	2.419977	0.051991	H	2.188348	2.254305	0.040847
H	−1.646797	−4.192769	−0.147749	H	0.586445	−3.056330	−0.109159
H	−4.011913	0.857709	0.048285	H	−6.177642	−0.328488	0.012744
H	−6.228116	−2.827134	−0.076586	H	−4.111996	−4.110036	−0.129791
H	1.994476	−1.773916	1.811056	H	4.355649	−2.503936	1.819606
H	5.047155	−0.301958	−1.806358	H	2.675809	0.395178	−1.834170
H	−2.547858	4.640001	0.097840	H	6.761916	−1.858033	1.006914
H	6.404609	−2.969456	−0.314572	H	6.934644	−1.321851	−0.673926
H	0.988728	5.458451	−0.754477	H	0.975506	5.417466	1.003369
H	2.287726	4.627256	0.115207				

3d•− (anion radical) b3lyp/6-31+g(d,p) scrf=(cpcm,solvent=acetonitrile)

opt freq b3lyp/6-31+g(d,p) scrf=(cpcm,solvent=acetonitrile)

Charge = −1, Multiplicity = 2, Point group = C1

Electronic Energy = −3587.975389 Hartree

Number of imaginary frequencies = 0

Sum of electronic and zero-point Energies = −3587.655159 Hartree

Sum of electronic and thermal Energies = −3587.633590 Hartree

Sum of electronic and thermal Enthalpies = −3587.632646 Hartree

Sum of electronic and thermal Free Energies = −3587.709776 Hartree

Atoms	Cartesian Coordinates			Atoms	Cartesian Coordinates		
	X	*Y*	*Z*		*X*	*Y*	*Z*
C	−1.414619	3.763887	0.106869	C	−0.017651	3.703938	0.099136

C	0.671580	2.485169	0.061073	C	−0.063973	1.275049	0.025189
C	−1.493566	1.357380	0.021334	C	−2.168193	2.572303	0.068931
C	0.280501	−0.117531	−0.000834	C	−0.944737	−0.855092	−0.018929
N	−2.011300	0.055145	−0.002212	C	1.538634	−0.824418	−0.025482
C	1.483274	−2.277805	−0.079207	C	0.235990	−2.942808	−0.107505
C	−0.994996	−2.244451	−0.078410	C	2.797235	−0.187998	−0.001839
C	3.978912	−0.949002	−0.030328	C	3.956501	−2.342192	−0.081054
C	2.724759	−2.994152	−0.103907	C	−3.390883	−0.284799	−0.005105
C	−4.230004	0.156241	−1.036827	C	−5.587588	−0.173636	−1.029171
C	−6.141550	−0.960521	−0.008287	C	−5.286294	−1.405971	1.012553
C	−3.931967	−1.068234	1.024059	Br	5.672512	−0.029676	0.002042
O	0.662371	4.912952	0.132773	C	−7.614642	−1.298040	0.008089
H	−1.908709	4.729609	0.140510	H	1.755777	2.493407	0.063886
H	−3.252269	2.614346	0.075467	H	0.229544	−4.029480	−0.151782
H	−1.938668	−2.778428	−0.101817	H	2.866015	0.889637	0.039866
H	4.879412	−2.913467	−0.102173	H	2.698872	−4.080683	−0.143642
H	−3.817449	0.751312	−1.845623	H	−6.222481	0.177813	−1.838709
H	−5.685990	−2.019254	1.816513	H	−3.289550	−1.411031	1.829028
H	1.615371	4.747785	0.126541	H	−8.050120	−1.229600	−0.993090
H	−7.788364	−2.309323	0.389075	H	−8.169508	−0.607489	0.655695

3e$^{\bullet-}$ (anion radical) b3lyp/6-31+g(d,p) scrf=(cpcm,solvent=acetonitrile)

opt freq b3lyp/6-31+g(d,p) scrf=(cpcm,solvent=acetonitrile)

Charge = −1, Multiplicity = 2, Point group = C1

Electronic Energy = −1109.112943 Hartree

Number of imaginary frequencies = 0

Sum of electronic and zero-point Energies = −1108.783705 Hartree

Sum of electronic and thermal Energies = −1108.762737 Hartree

Sum of electronic and thermal Enthalpies = −1108.761793 Hartree

Sum of electronic and thermal Free Energies = −1108.834942 Hartree

**Atoms**	**Cartesian Coordinates**			**Atoms**	**Cartesian Coordinates**		
	X	***Y***	***Z***		***X***	***Y***	***Z***
C	−0.735511	3.761013	0.100955	C	0.665853	3.703061	0.093699
C	1.350099	2.485262	0.058805	C	0.613401	1.280135	0.024972
C	−0.812818	1.360847	0.021947	C	−1.487761	2.578657	0.066708
C	0.956764	−0.118676	−0.000178	C	−0.266493	−0.841713	−0.016387
N	−1.331023	0.062055	−0.001036	C	2.214811	−0.831077	−0.023157
C	2.136862	−2.284831	−0.071419	C	0.891667	−2.935505	−0.095877
C	−0.330386	−2.240712	−0.069730	C	3.472489	−0.214387	−0.003846

C	4.685754	−0.977823	−0.029639	C	4.597811	−2.404714	−0.076179
C	3.375122	−3.026425	−0.095751	C	−2.713872	−0.277840	−0.004437
C	−3.535319	0.110239	−1.069804	C	−4.893141	−0.218410	−1.063776
C	−5.461199	−0.950227	−0.010015	C	−4.621692	−1.341974	1.045225
C	−3.267148	−1.004767	1.057795	O	1.337960	4.913089	0.125194
C	−6.934647	−1.285740	0.003482	C	5.939239	−0.341914	−0.009918
N	6.988661	0.196398	0.006562	H	−1.227300	4.727917	0.132889
H	2.434304	2.488532	0.061551	H	−2.571857	2.620861	0.073306
H	0.879745	−4.022692	−0.136479	H	−1.277750	−2.766919	−0.091199
H	3.551546	0.864056	0.033115	H	5.510940	−2.993742	−0.096119
H	3.323751	−4.112330	−0.131530	H	−3.109456	0.661884	−1.902266
H	−5.516748	0.090102	−1.898982	H	−5.033535	−1.912335	1.874070
H	−2.635269	−1.304672	1.887981	H	2.292569	4.756641	0.117131
H	−7.355085	−1.274240	−1.006258	H	−7.115089	−2.273286	0.439368
H	−7.497236	−0.558546	0.602333				

6a$^{•−}$ (anion radical) b3lyp/6-31+g(d,p) scrf=(cpcm,solvent=acetonitrile)

opt freq b3lyp/6-31+g(d,p) scrf=(cpcm,solvent=acetonitrile)

Charge = −1, Multiplicity = 2, Point group = C1

Electronic Energy = −1591.317077 Hartree

Number of imaginary frequencies = 0

Sum of electronic and zero-point Energies = −1590.823984 Hartree

Sum of electronic and thermal Energies = −1590.792549 Hartree

Sum of electronic and thermal Enthalpies = −1590.791605 Hartree

Sum of electronic and thermal Free Energies = −1590.889071 Hartree

Atoms	Cartesian Coordinates			Atoms	Cartesian Coordinates		
	X	*Y*	*Z*		*X*	*Y*	*Z*
C	−0.568270	−3.123535	0.559931	C	0.624663	−2.422295	0.722803
C	0.723160	−1.009232	0.596803	C	−0.487280	−0.300989	0.233479
C	−1.714769	−1.050019	0.186715	C	−1.773105	−2.430491	0.316677
C	−0.830071	1.064506	−0.101682	C	−2.255130	1.116191	−0.224077
N	−2.769886	−0.170876	−0.064819	C	−0.091107	2.281877	−0.398496
C	−0.848506	3.504809	−0.595592	C	−2.268141	3.490963	−0.569263
C	−2.978014	2.293524	−0.422910	C	1.309377	2.343251	−0.580765
C	1.971869	3.534728	−0.880066	C	1.241617	4.733415	−1.002346
C	−0.136150	4.712836	−0.864104	C	−4.142217	−0.534677	−0.155823
C	−4.803740	−1.087953	0.948121	C	−6.147792	−1.454354	0.850382
C	−6.870625	−1.265281	−0.337857	C	−6.195203	−0.707247	−1.434783
C	−4.847593	−0.351069	−1.352488	O	1.712746	−3.187847	1.107169

C	−8.335531	−1.625899	−0.426397	C	1.956469	−0.335011	1.067173
C	3.252312	−0.552283	0.463884	C	4.427001	0.048145	1.045435
C	4.285631	0.892867	2.180964	C	3.037723	1.141506	2.719883
C	1.891679	0.531190	2.170312	C	3.410051	−1.316378	−0.720976
C	4.662287	−1.535485	−1.284724	C	5.821143	−0.987850	−0.688017
C	5.687232	−0.207209	0.449163	O	0.708229	0.794976	2.813650
O	4.681057	−2.289758	−2.431862	C	5.934345	−2.533069	−3.073311
H	−0.564950	−4.200622	0.694384	H	−2.713789	−2.964808	0.242273
H	−2.804186	4.424390	−0.720130	H	−4.062110	2.278603	−0.465779
H	1.887638	1.433996	−0.502039	H	3.050182	3.532732	−1.014203
H	1.754015	5.667725	−1.218707	H	−0.709725	5.629447	−0.986347
H	−4.263587	−1.232095	1.878721	H	−6.643184	−1.887676	1.715662
H	−6.726413	−0.558741	−2.371656	H	−4.337738	0.065673	−2.215528
H	2.501263	−2.626734	1.157890	H	−8.586672	−2.445538	0.253399
H	−8.967527	−0.770767	−0.155224	H	−8.611694	−1.925120	−1.442075
H	5.171148	1.347422	2.616758	H	2.917118	1.784004	3.587096
H	2.538429	−1.731609	−1.216078	H	6.803859	−1.155164	−1.111605
H	6.573876	0.231921	0.899838	H	−0.022363	0.425313	2.286833
H	5.704632	−3.136135	−3.952135	H	6.409197	−1.596872	−3.388344
H	6.615639	−3.089061	−2.419227				

6b$^{\bullet-}$ (anion radical) b3lyp/6-31+g(d,p) scrf=(cpcm,solvent=acetonitrile)

opt freq b3lyp/6-31+g(d,p) scrf=(cpcm,solvent=acetonitrile)

Charge = −1, Multiplicity = 2, Point group = C1

Electronic Energy = −6619.052100 Hartree

Number of imaginary frequencies = 0

Sum of electronic and zero-point Energies = −6618.611161 Hartree

Sum of electronic and thermal Energies = −6618.579533 Hartree

Sum of electronic and thermal Enthalpies = −6618.578589 Hartree

Sum of electronic and thermal Free Energies = −6618.677982 Hartree

Atoms	Cartesian Coordinates			Atoms	Cartesian Coordinates		
	X	*Y*	*Z*		*X*	*Y*	*Z*
C	−1.553364	−3.219675	0.891748	C	−0.327104	−2.600986	1.133479
C	−0.113232	−1.206947	0.962095	C	−1.228388	−0.437758	0.461097
C	−2.499965	−1.091335	0.334144	C	−2.674627	−2.458152	0.514346
C	−1.435783	0.935268	0.044983	C	−2.834641	1.084397	−0.198694
N	−3.458367	−0.148786	−0.040031	C	−0.587222	2.075054	−0.238588
C	−1.225769	3.340273	−0.538882	C	−2.639892	3.433748	−0.630490
C	−3.447038	2.306395	−0.500240	C	0.824706	2.014409	−0.308939

C	1.571559	3.146601	−0.596224	C	0.974625	4.396975	−0.825800
C	−0.405683	4.476947	−0.795426	C	−4.845321	−0.411781	−0.232755
C	−5.642875	−0.822071	0.842109	C	−6.999276	−1.090277	0.644917
C	−7.595401	−0.941679	−0.616943	C	−6.782221	−0.527178	−1.683853
C	−5.422224	−0.270221	−1.501180	O	0.664347	−3.420534	1.639267
C	−9.071581	−1.194664	−0.817705	C	1.122956	−0.595818	1.498662
C	2.440578	−0.914970	0.999301	C	3.608920	−0.349444	1.630163
C	3.440107	0.551714	2.710143	C	2.169705	0.900463	3.151726
C	1.033297	0.333412	2.556017	C	2.626232	−1.747028	−0.137979
C	3.904577	−2.047303	−0.581082	C	5.054759	−1.548875	0.051602
C	4.895890	−0.704330	1.138675	Br	4.111618	−3.184010	−2.112886
O	−0.175495	0.690449	3.100463	Br	3.484577	2.995201	−0.729031
H	−1.643995	−4.286218	1.072068	H	−3.643270	−2.926230	0.379056
H	−3.086770	4.398315	−0.854849	H	−4.522206	2.370099	−0.629606
H	1.327504	1.072484	−0.155906	H	1.578023	5.273237	−1.036635
H	−0.889763	5.429274	−0.997413	H	−5.199620	−0.932191	1.827002
H	−7.603855	−1.413709	1.488388	H	−7.214984	−0.413625	−2.674573
H	−4.804019	0.034958	−2.339821	H	1.456849	−2.891209	1.817446
H	−9.460495	−1.897473	−0.075189	H	−9.645272	−0.264302	−0.721147
H	−9.274844	−1.599385	−1.813926	H	4.317441	0.983977	3.183112
H	2.033280	1.587851	3.981292	H	1.761032	−2.132384	−0.664254
H	6.043364	−1.809437	−0.309950	H	5.773622	−0.294447	1.631656
H	−0.888915	0.309900	2.560171				

7$^{\bullet-}$ (anion radical) b3lyp/6-31+g(d,p) scrf=(cpcm,solvent=acetonitrile)

# opt freq b3lyp/6-31+g(d,p) scrf=(cpcm,solvent=acetonitrile)
Charge = −1, Multiplicity = 2, Point group = C1
Electronic Energy = −1722.701578 Hartree
Number of imaginary frequencies = 0
Sum of electronic and zero-point Energies = −1722.192042 Hartree
Sum of electronic and thermal Energies = −1722.160379 Hartree
Sum of electronic and thermal Enthalpies = −1722.159434 Hartree
Sum of electronic and thermal Free Energies = −1722.257469 Hartree

Atoms	Cartesian Coordinates			Atoms	Cartesian Coordinates		
	X	*Y*	*Z*		*X*	*Y*	*Z*
C	4.174933	3.241334	−1.979333	C	5.540420	2.915189	−1.862257
C	5.957422	1.761398	−1.203323	C	4.988385	0.901836	−0.646347
C	3.614261	1.243811	−0.783104	C	3.200951	2.407777	−1.438840
C	5.032219	−0.340106	0.099600	C	3.692399	−0.687652	0.374811

N	2.838334	0.267861	−0.157035	C	6.088130	−1.197358	0.553895
C	5.721729	−2.389068	1.271906	C	4.342436	−2.690042	1.508680
C	3.333601	−1.864680	1.074088	C	7.469948	−0.945377	0.340978
C	8.439255	−1.815556	0.807971	C	8.077057	−2.985402	1.513279
C	6.741562	−3.260225	1.737339	C	1.413079	0.262944	−0.074159
C	0.642144	0.098870	−1.231836	C	−0.749581	0.109843	−1.154559
C	−1.393399	0.257785	0.085365	C	−0.613572	0.407705	1.244312
C	0.778384	0.425750	1.162866	O	6.431236	3.800217	−2.432248
N	−2.803656	0.256801	0.165428	C	−4.183942	3.274760	1.947333
C	−5.533350	2.934087	1.817881	C	−5.945532	1.763324	1.169440
C	−4.967315	0.888009	0.627948	C	−3.588282	1.244467	0.788444
C	−3.192238	2.415658	1.421989	C	−5.010916	−0.341986	−0.103080
C	−3.659228	−0.720377	−0.380962	C	−6.094704	−1.193211	−0.548278
C	−5.736068	−2.416600	−1.248642	C	−4.373968	−2.740492	−1.470020
C	−3.320977	−1.897171	−1.040116	C	−7.461671	−0.911498	−0.343403
C	−8.478056	−1.774613	−0.794886	C	−8.134043	−2.954344	−1.472335
C	−6.795963	−3.268694	−1.695212	O	−6.457146	3.815130	2.362516
H	3.895276	4.153123	−2.497006	H	7.017674	1.547165	−1.129899
H	2.149850	2.660220	−1.527701	H	4.098803	−3.601303	2.047930
H	2.292441	−2.106103	1.258276	H	7.779038	−0.056678	−0.194398
H	9.488023	−1.595363	0.630160	H	8.844988	−3.662183	1.875716
H	6.450474	−4.157072	2.278518	H	1.132998	−0.026758	−2.191803
H	−1.342454	0.002866	−2.056237	H	−1.099027	0.509474	2.208758
H	1.374311	0.555206	2.060876	H	7.338526	3.492686	−2.296186
H	−3.907798	4.194616	2.452502	H	−7.005644	1.550890	1.088450
H	−2.145038	2.680947	1.518002	H	−4.136981	−3.665303	−1.990901
H	−2.287588	−2.171823	−1.221235	H	−7.748554	−0.004224	0.174368
H	−9.520348	−1.523057	−0.617713	H	−8.912567	−3.627432	−1.825392
H	−6.533701	−4.185227	−2.220465	H	−7.351228	3.479330	2.209874

10a$^{\bullet-}$ (anion radical) b3lyp/6-31+g(d,p) scrf=(cpcm,solvent=acetonitrile)

\# opt freq b3lyp/6-31+g(d,p) scrf=(cpcm,solvent=acetonitrile)

Charge = −1, Multiplicity = 2, Point group = C1

Electronic Energy = −785.760484 Hartree

Number of imaginary frequencies = 0

Sum of electronic and zero-point Energies = −785.510128 Hartree

Sum of electronic and thermal Energies = −785.495174 Hartree

Sum of electronic and thermal Enthalpies = −785.494230 Hartree

Sum of electronic and thermal Free Energies = −785.552409 Hartree

Atoms	Cartesian Coordinates			Atoms	Cartesian Coordinates		
	X	Y	Z		X	Y	Z
C	−3.661989	−0.905140	−0.000031	C	−3.159003	0.405579	−0.000059
C	−1.781152	0.668169	−0.000041	C	−0.870446	−0.423863	0.000006
C	−1.409241	−1.751565	0.000021	C	−2.777230	−2.003109	0.000003
C	0.560365	−0.548900	0.000023	C	0.857906	−1.949001	0.000038
N	−0.345864	−2.644787	0.000055	C	1.649000	0.406623	0.000016
C	3.003733	−0.130623	−0.000025	C	3.222633	−1.529768	−0.000023
C	2.152725	−2.460329	0.000015	C	1.477499	1.805847	0.000055
C	2.571788	2.694750	0.000032	C	3.877925	2.180280	−0.000031
C	4.091499	0.804585	−0.000060	O	−4.122587	1.402689	−0.000127
C	−3.694500	2.760452	0.000100	H	−4.736645	−1.058085	−0.000037
H	−1.420890	1.686486	−0.000082	H	−3.161515	−3.019495	0.000019
H	−0.432463	−3.649212	0.000027	H	4.247838	−1.893858	−0.000048
H	2.336797	−3.531222	0.000024	H	0.475608	2.219584	0.000117
H	2.399665	3.767857	0.000064	H	4.729863	2.857523	−0.000058
H	5.107357	0.413283	−0.000104	H	−4.604473	3.362528	0.000149
H	−3.104272	2.996200	0.894305	H	−3.104193	2.996481	−0.893980

10b$^{\bullet-}$ (anion radical) b3lyp/6-31+g(d,p) scrf=(cpcm,solvent=acetonitrile)

opt freq b3lyp/6-31+g(d,p) scrf=(cpcm,solvent=acetonitrile)

Charge = −1, Multiplicity = 2, Point group = C1

Electronic Energy = −825.071238 Hartree

Number of imaginary frequencies = 0

Sum of electronic and zero-point Energies = −824.792780 Hartree

Sum of electronic and thermal Energies = −824.776271 Hartree

Sum of electronic and thermal Enthalpies = −824.775327 Hartree

Sum of electronic and thermal Free Energies = −824.837175 Hartree

Atoms	Cartesian Coordinates			Atoms	Cartesian Coordinates		
	X	Y	Z		X	Y	Z
C	3.652114	0.519510	0.012304	C	3.100653	−0.771000	0.004569
C	1.713888	−0.979554	−0.008552	C	0.845683	0.146022	−0.015608
C	1.431195	1.454843	−0.018591	C	2.809616	1.650670	0.003584
C	−0.576485	0.323197	−0.015937	C	−0.819363	1.735421	−0.017746
N	0.407145	2.397123	−0.048811	C	−1.697412	−0.593021	−0.009379
C	−3.032284	−0.010074	0.007733	C	−3.200132	1.395987	0.017297
C	−2.098168	2.288419	0.007157	C	−1.575829	−1.998100	−0.017267
C	−2.700564	−2.847307	−0.008277	C	−3.987971	−2.287925	0.009141
C	−4.152226	−0.905261	0.017047	C	0.587259	3.830211	0.023579

O	4.025005	−1.804337	0.014649	C	3.545325	−3.144745	0.010154
H	4.731498	0.632974	0.029518	H	1.314859	−1.983258	−0.009007
H	3.241527	2.646680	0.017377	H	−4.211113	1.797424	0.036358
H	−2.256147	3.362278	0.022644	H	−0.589697	−2.447547	−0.032232
H	−2.566145	−3.925792	−0.015201	H	−4.863118	−2.934753	0.016254
H	−5.153322	−0.477777	0.030368	H	−0.238240	4.332360	−0.486900
H	0.628494	4.192297	1.060549	H	1.514979	4.113163	−0.480004
H	4.431621	−3.781095	0.018292	H	2.938223	−3.358044	0.898697
H	2.954734	−3.357826	−0.889497				

10c$^{\bullet-}$ (anion radical) b3lyp/6-31+g(d,p) scrf=(cpcm,solvent=acetonitrile)

opt freq b3lyp/6-31+g(d,p) scrf=(cpcm,solvent=acetonitrile)

Charge = −1, Multiplicity = 2, Point group = C1

Electronic Energy = −1056.142199 Hartree

Number of imaginary frequencies = 0

Sum of electronic and zero-point Energies = −1055.784069 Hartree

Sum of electronic and thermal Energies = −1055.762589 Hartree

Sum of electronic and thermal Enthalpies = −1055.761645 Hartree

Sum of electronic and thermal Free Energies = −1055.836965 Hartree

Atoms	Cartesian Coordinates			Atoms	Cartesian Coordinates		
	X	*Y*	*Z*		*X*	*Y*	*Z*
C	0.120838	3.557674	0.086278	C	1.498149	3.288152	0.076675
C	1.990173	1.975934	0.043069	C	1.070330	0.892003	0.013960
C	−0.328774	1.192589	0.011873	C	−0.807818	2.497657	0.054731
C	1.199946	−0.535440	−0.004772	C	−0.123474	−1.079389	−0.014821
N	−1.040505	−0.015409	−0.001809	C	2.338251	−1.431094	−0.029398
C	2.059485	−2.859494	−0.073523	C	0.722953	−3.327345	−0.093838
C	−0.385915	−2.445040	−0.065663	C	3.683375	−1.007860	−0.016398
C	4.756220	−1.921147	−0.044395	C	4.489766	−3.298332	−0.085014
C	3.175477	−3.758290	−0.098591	C	−2.454626	−0.139100	−0.003534
C	−3.219379	0.441471	−1.024720	C	−4.611563	0.324551	−1.016119
C	−5.278627	−0.383219	−0.005044	C	−4.499656	−0.970747	1.004948
C	−3.109373	−0.845991	1.015550	C	−6.786242	−0.489204	0.013014
O	2.315427	4.406263	0.103874	C	3.726847	4.216921	0.102550
H	−0.216679	4.588780	0.115911	H	3.054690	1.794681	0.044174
H	−1.872397	2.707330	0.063062	H	0.549058	−4.400540	−0.131213
H	−1.400906	−2.827468	−0.083048	H	3.909557	0.051237	0.017009
H	5.779672	−1.555669	−0.033575	H	5.310840	−4.012201	−0.106234
H	2.974928	−4.827721	−0.131055	H	−2.722345	0.978164	−1.826787

H	−5.186077	0.782103	−1.817803	H	−4.987229	−1.527240	1.801769
H	−2.526398	−1.296258	1.812720	H	−7.207872	−0.342655	−0.985775
H	−7.113691	−1.465850	0.383198	H	−7.226843	0.271231	0.670165
H	4.163855	5.216320	0.127167	H	4.057870	3.654863	0.984533
H	4.061903	3.697084	−0.803457				

$12^{\bullet-}$ (anion radical) b3lyp/6-31+g(d,p) scrf=(cpcm,solvent=acetonitrile)

opt freq b3lyp/6-31+g(d,p) scrf=(cpcm,solvent=acetonitrile)

Charge = −1, Multiplicity = 2, Point group = C1

Electronic Energy = −5659.837785 Hartree

Number of imaginary frequencies = 0

Sum of electronic and zero-point Energies = −5659.684154 Hartree

Sum of electronic and thermal Energies = −5659.672161 Hartree

Sum of electronic and thermal Enthalpies = −5659.671217 Hartree

Sum of electronic and thermal Free Energies = −5659.724973 Hartree

Atoms	Cartesian Coordinates			Atoms	Cartesian Coordinates		
	X	*Y*	*Z*		*X*	*Y*	*Z*
C	−1.139494	1.963744	−0.000007	C	−0.709714	0.593803	0.000009
C	0.709714	0.593803	0.000015	C	1.139494	1.963744	−0.000001
N	−0.000000	2.759859	−0.000020	C	−2.476297	2.335175	−0.000017
C	−3.459978	1.299348	−0.000002	C	−3.036399	−0.030107	0.000032
C	−1.701179	−0.437671	0.000035	C	1.701179	−0.437671	0.000052
C	3.036399	−0.030107	0.000062	C	3.459978	1.299348	0.000018
C	2.476297	2.335175	−0.000004	Br	−4.399682	−1.409405	−0.000009
Br	4.399682	−1.409405	−0.000019	H	−0.000000	3.768653	−0.000039
H	−2.776667	3.378925	−0.000041	H	−4.514837	1.547463	−0.000019
H	−1.428928	−1.486805	0.000046	H	1.428928	−1.486805	0.000064
H	4.514837	1.547463	−0.000006	H	2.776666	3.378926	−0.000030

$3a^{\bullet+}$ (cation radical) b3lyp/6-311+g(d,p) scrf=(cpcm,solvent=acetonitrile)

```
# opt freq b3lyp/6-311+g(d,p)
scrf=(cpcm,solvent=acetonitrile)
```

Charge = 1, Multiplicity = 2, Point group = C1

Electronic Energy = -1016.764582 Hartree

Number of imaginary frequencies = 0

Sum of electronic and zero-point Energies = -1016.430754 Hartree

Sum of electronic and thermal Energies = -1016.411311 Hartree

Sum of electronic and thermal Enthalpies = -1016.410367 Hartree

Sum of electronic and thermal Free Energies = -1016.480209 Hartree

	Cartesian Coordinates				Cartesian Coordinates		
	X	*Y*	*Z*		*X*	*Y*	*Z*
C	-0.357093	3.741480	0.130204	C	1.058509	3.709202	0.090452
C	1.780210	2.490273	0.052379	C	1.077711	1.304324	0.034556
C	-0.357932	1.354718	0.049754	C	-1.072118	2.569890	0.116350
C	1.440597	-0.110852	0.015120	C	0.237972	-0.824171	0.014455
N	-0.844277	0.083521	0.036724	C	2.673789	-0.814567	-0.024140
C	2.609472	-2.254712	-0.070076	C	1.353178	-2.917201	-0.086799
C	0.164865	-2.224610	-0.050499	C	3.952524	-0.197025	-0.022090
C	5.100671	-0.952263	-0.060148	C	5.037001	-2.365540	-0.103855
C	3.817911	-2.998449	-0.108750	C	-2.224942	-0.282365	0.012190
C	-3.046992	0.187119	-1.014672	C	-4.389599	-0.173719	-1.030963
C	-4.934178	-0.995682	-0.036160	C	-4.085862	-1.456586	0.981100
C	-2.741167	-1.110291	1.012624	O	1.678629	4.893032	0.104317
C	-6.396052	-1.358981	-0.043234	H	-0.852018	4.702145	0.180189
H	2.860601	2.526577	0.043333	H	-2.152460	2.579125	0.162525
H	1.340104	-3.999689	-0.134703	H	-0.785244	-2.739920	-0.077845
H	4.035429	0.879217	0.013772	H	6.066366	-0.460556	-0.055736
H	5.952782	-2.943768	-0.133642	H	3.760290	-4.080629	-0.143102
H	-2.633836	0.801517	-1.805103	H	-5.020355	0.181400	-1.837992
H	-4.484411	-2.091513	1.764439	H	-2.103400	-1.458254	1.815509
H	2.641698	4.796279	0.081368	H	-6.822468	-1.273893	-1.044091
H	-6.553291	-2.377633	0.317534	H	-6.959741	-0.689239	0.615148

3b$^{\bullet+}$ (cation radical) b3lyp/6-311+g(d,p) scrf=(cpcm,solvent=acetonitrile)

```
# opt freq b3lyp/6-311+g(d,p)
scrf=(cpcm,solvent=acetonitrile)
```

Charge = 1, Multiplicity = 2, Point group = C1

Electronic Energy = -1437.056670 Hartree

Number of imaginary frequencies = 0

Sum of electronic and zero-point Energies = -1436.759292 Hartree

Sum of electronic and thermal Energies = -1436.740558 Hartree

Sum of electronic and thermal Enthalpies = -1436.739614 Hartree

Sum of electronic and thermal Free Energies = -1436.807698 Hartree

	Cartesian Coordinates				Cartesian Coordinates		
	X	*Y*	*Z*		*X*	*Y*	*Z*
C	-0.166809	3.802237	0.117942	C	-1.582073	3.683809	0.082663
C	-2.231008	2.425024	0.047165	C	-1.459135	1.283351	0.032839
C	-0.025444	1.421406	0.048787	C	0.617109	2.678994	0.105516
C	-1.734567	-0.148996	0.012451	C	-0.491919	-0.787855	0.012772

N	0.534532	0.186138	0.036390	C	-2.923166	-0.927964	-0.024405
C	-2.769388	-2.361440	-0.066034	C	-1.475487	-2.944787	-0.077539
C	-0.330737	-2.179368	-0.042527	C	-4.237043	-0.391550	-0.023622
C	-5.335890	-1.217637	-0.060239	C	-5.185267	-2.624455	-0.100618
C	-3.929603	-3.179896	-0.103366	C	1.937410	-0.096991	0.017312
C	2.509800	-0.809922	1.071790	C	3.873040	-1.082083	1.055173
C	4.641636	-0.637877	-0.018223	C	4.076933	0.071650	-1.074611
C	2.713032	0.342925	-1.056304	O	-2.265699	4.829011	0.095476
Cl	6.362484	-0.982602	-0.039775	H	0.266640	4.792416	0.161463
H	-3.311489	2.396956	0.035765	H	1.695431	2.752079	0.144101
H	-1.395037	-4.024630	-0.119154	H	0.649804	-2.635169	-0.062976
H	-4.388001	0.677513	0.009154	H	-6.330187	-0.786718	-0.057434
H	-6.063612	-3.258054	-0.129351	H	-3.804066	-4.256334	-0.134737
H	1.903217	-1.133094	1.908153	H	4.330553	-1.625436	1.871085
H	4.687364	0.401282	-1.904660	H	2.255228	0.875111	-1.880661
H	-3.222757	4.681816	0.075379				

3c$^{\bullet+}$ (cation radical) b3lyp/6-311+g(d,p) scrf=(cpcm,solvent=acetonitrile)

```
# opt freq b3lyp/6-311+g(d,p)
scrf=(cpcm,solvent=acetonitrile)
```

Charge = 1, Multiplicity = 2, Point group = C1

Electronic Energy = -1056.094357 Hartree

Number of imaginary frequencies = 0

Sum of electronic and zero-point Energies = -1055.732818 Hartree

Sum of electronic and thermal Energies = -1055.711648 Hartree

Sum of electronic and thermal Enthalpies = -1055.710704 Hartree

Sum of electronic and thermal Free Energies = -1055.784388 Hartree

	**Cartesian Coordinates**				**Cartesian Coordinates**		
	X	***Y***	***Z***		***X***	***Y***	***Z***
C	0.558705	3.494351	0.086640	C	-0.877767	3.532721	0.049343
C	-1.680170	2.371231	0.024622	C	-1.065032	1.137334	0.019257
C	0.377323	1.097778	0.035486	C	1.170814	2.265545	0.082705
C	-1.515839	-0.246171	0.009724	C	-0.362529	-1.034069	0.015978
N	0.781783	-0.194100	0.033637	C	-2.793697	-0.871924	-0.022762
C	-2.820746	-2.312744	-0.052562	C	-1.610119	-3.051407	-0.057845
C	-0.377159	-2.432612	-0.028498	C	-4.029309	-0.176127	-0.029170
C	-5.223868	-0.858576	-0.060433	C	-5.251407	-2.273066	-0.088151
C	-4.074904	-2.980589	-0.084441	C	2.137685	-0.652717	0.010595
C	2.620231	-1.441083	1.056823	C	3.940032	-1.876739	1.027737
C	4.794154	-1.543793	-0.032699	C	4.284943	-0.751315	-1.068906

C	2.966946	-0.306370	-1.057053	O	-1.421454	4.751695	0.050981
C	6.214062	-2.047819	-0.067426	C	1.332123	4.779966	0.137346
H	-2.755600	2.486251	0.014438	H	2.249539	2.191100	0.121011
H	-1.663463	-4.133427	-0.089676	H	0.538888	-3.007223	-0.043842
H	-4.043738	0.903776	-0.006637	H	-6.156095	-0.305817	-0.063242
H	-6.202195	-2.791949	-0.112671	H	-4.084269	-4.064596	-0.106618
H	1.977906	-1.690458	1.892472	H	4.314271	-2.477819	1.848927
H	4.924331	-0.481687	-1.902041	H	2.580601	0.289417	-1.875102
H	-2.388725	4.713038	0.030827	H	6.633616	-2.121185	0.938007
H	6.255576	-3.047617	-0.512756	H	6.853325	-1.394985	-0.664408
H	1.118135	5.399087	-0.738251	H	1.057205	5.366928	1.017985
H	2.403010	4.581493	0.170406				

3d$^{•+}$ (cation radical) b3lyp/6-311+g(d,p) scrf=(cpcm,solvent=acetonitrile)

```
# opt freq b3lyp/6-311+g(d,p)
scrf=(cpcm,solvent=acetonitrile)
```

Charge = 1, Multiplicity = 2, Point group = C1

Electronic Energy = -3590.305965 Hartree

Number of imaginary frequencies = 0

Sum of electronic and zero-point Energies = -3589.982225 Hartree

Sum of electronic and thermal Energies = -3589.961229 Hartree

Sum of electronic and thermal Enthalpies = -3589.960285 Hartree

Sum of electronic and thermal Free Energies = -3590.034913 Hartree

	Cartesian Coordinates				Cartesian Coordinates		
	X	*Y*	*Z*		*X*	*Y*	*Z*
C	-1.530231	3.743330	0.132565	C	-0.110699	3.724300	0.096013
C	0.623195	2.514658	0.061842	C	-0.067782	1.321235	0.044798
C	-1.506939	1.357702	0.057303	C	-2.234051	2.568036	0.118994
C	0.306430	-0.087645	0.025956	C	-0.888720	-0.812509	0.021912
N	-1.982066	0.086369	0.042922	C	1.545004	-0.784789	-0.009586
C	1.491579	-2.223472	-0.055410	C	0.243566	-2.896045	-0.074844
C	-0.951443	-2.210955	-0.041446	C	2.814547	-0.151891	-0.003151
C	3.957044	-0.912195	-0.037965	C	3.924291	-2.324242	-0.081934
C	2.707007	-2.956809	-0.090158	C	-3.359895	-0.293872	0.011832
C	-4.176593	0.151236	-1.029571	C	-5.515686	-0.222921	-1.052073
C	-6.060856	-1.035353	-0.049964	C	-5.217274	-1.472380	0.981548
C	-3.876418	-1.111331	1.020167	Br	5.664538	-0.042948	-0.025212
O	0.492841	4.914863	0.108778	C	-7.518985	-1.413673	-0.064647
H	-2.032289	4.700466	0.179534	H	1.703065	2.563689	0.054263
H	-3.314527	2.565869	0.161194	H	0.238707	-3.978455	-0.121279

H	-1.897480	-2.733621	-0.069551	H	2.892864	0.923048	0.032940
H	4.845158	-2.891165	-0.108669	H	2.663064	-4.039300	-0.124464
H	-3.762498	0.758454	-1.825154	H	-6.142763	0.114102	-1.869672
H	-5.616343	-2.100006	1.770496	H	-3.242316	-1.441166	1.833649
H	1.457449	4.831740	0.087532	H	-7.937172	-1.347485	-1.070397
H	-7.669171	-2.428604	0.309356	H	-8.095394	-0.740356	0.578866

3e$^{\bullet+}$ (cation radical) b3lyp/6-311+g(d,p) scrf=(cpcm,solvent=acetonitrile)

```
# opt freq b3lyp/6-311+g(d,p)
scrf=(cpcm,solvent=acetonitrile)
```

Charge = 1, Multiplicity = 2, Point group = C1

Electronic Energy = -1109.032586 Hartree

Number of imaginary frequencies = 0

Sum of electronic and zero-point Energies = -1108.699805 Hartree

Sum of electronic and thermal Energies = -1108.678568 Hartree

Sum of electronic and thermal Enthalpies = -1108.677624 Hartree

Sum of electronic and thermal Free Energies = -1108.751806 Hartree

	**Cartesian Coordinates**				**Cartesian Coordinates**		
	X	***Y***	***Z***		***X***	***Y***	***Z***
C	-0.821859	3.742848	0.133928	C	0.596023	3.711966	0.092658
C	1.319753	2.493117	0.056276	C	0.618270	1.307529	0.041647
C	-0.818811	1.356133	0.057980	C	-1.535112	2.572709	0.122906
C	0.980134	-0.106878	0.022944	C	-0.220703	-0.819935	0.022680
N	-1.305394	0.088307	0.045231	C	2.211163	-0.817682	-0.015929
C	2.147101	-2.255432	-0.061846	C	0.891075	-2.916687	-0.078388
C	-0.294347	-2.219579	-0.041584	C	3.483389	-0.199481	-0.012306
C	4.636382	-0.962758	-0.050145	C	4.575352	-2.382863	-0.094438
C	3.353966	-3.002464	-0.099649	C	-2.685999	-0.279660	0.014181
C	-3.501687	0.184309	-1.020191	C	-4.843744	-0.177934	-1.042801
C	-5.393250	-0.996032	-0.047522	C	-4.550646	-1.451643	0.977060
C	-3.206639	-1.103763	1.015349	O	1.211814	4.894554	0.103748
C	-6.854495	-1.361039	-0.061738	C	5.911338	-0.314164	-0.043609
N	6.945882	0.201600	-0.038614	H	-1.316216	4.703806	0.183191
H	2.399926	2.533442	0.045826	H	-2.615339	2.579804	0.170265
H	0.875495	-3.998751	-0.125671	H	-1.245508	-2.733073	-0.067767
H	3.572764	0.875044	0.023963	H	5.490951	-2.958867	-0.123757
H	3.295914	-4.083955	-0.133916	H	-3.084278	0.795284	-1.811078
H	-5.469989	0.172764	-1.855203	H	-4.953376	-2.083335	1.760821
H	-2.573718	-1.447207	1.824046	H	2.175835	4.803365	0.080397
H	-7.275222	-1.280171	-1.065312	H	-7.012685	-2.378389	0.302207

H	-7.422411	-0.689245	0.590899

6a$^{\bullet+}$ (cation radical) b3lyp/6-311+g(d,p) scrf=(cpcm,solvent=acetonitrile)

```
# opt freq b3lyp/6-311+g(d,p)
scrf=(cpcm,solvent=acetonitrile)
```

Charge = 1, Multiplicity = 2, Point group = C1

Electronic Energy = -1591.353545 Hartree

Number of imaginary frequencies = 0

Sum of electronic and zero-point Energies = -1590.856735 Hartree

Sum of electronic and thermal Energies = -1590.825902 Hartree

Sum of electronic and thermal Enthalpies = -1590.824958 Hartree

Sum of electronic and thermal Free Energies = -1590.920900 Hartree

	**Cartesian Coordinates**				**Cartesian Coordinates**		
	X	***Y***	***Z***		***X***	***Y***	***Z***
C	-0.687695	-3.039937	1.005319	C	0.532035	-2.351616	1.179337
C	0.700544	-0.949333	0.892386	C	-0.416883	-0.253518	0.423622
C	-1.666910	-0.970204	0.352389	C	-1.798231	-2.350401	0.601219
C	-0.727004	1.125808	-0.011508	C	-2.113507	1.157390	-0.223360
N	-2.656682	-0.119497	-0.017437	C	0.015536	2.322601	-0.264280
C	-0.731959	3.513526	-0.599107	C	-2.146048	3.477275	-0.713752
C	-2.846525	2.309521	-0.549441	C	1.431035	2.421921	-0.260996
C	2.068436	3.612491	-0.521254	C	1.331126	4.785201	-0.801087
C	-0.039940	4.728481	-0.843034	C	-4.041094	-0.458442	-0.158787
C	-4.752883	-0.931742	0.944427	C	-6.095870	-1.261867	0.796922
C	-6.746496	-1.127532	-0.435981	C	-6.006697	-0.652674	-1.528049
C	-4.664707	-0.314698	-1.399530	O	1.542760	-3.076466	1.657580
C	-8.209285	-1.459028	-0.580508	C	2.044679	-0.386961	1.203499
C	3.169902	-0.650731	0.342364	C	4.463569	-0.171884	0.727578
C	4.600718	0.556747	1.937616	C	3.522500	0.808885	2.742458
C	2.239609	0.333688	2.377369	C	3.037495	-1.339429	-0.882656
C	4.139441	-1.578157	-1.689311	C	5.422150	-1.120195	-1.303057
C	5.564891	-0.429835	-0.120857	O	1.242271	0.625090	3.254043
O	3.899126	-2.256112	-2.842963	C	4.988448	-2.536408	-3.729954
H	-0.720211	-4.099959	1.218092	H	-2.751532	-2.845149	0.475548
H	-2.671576	4.393577	-0.955261	H	-3.921958	2.275099	-0.655886
H	2.022842	1.543572	-0.070684	H	3.151576	3.648754	-0.517139
H	1.848412	5.717287	-0.994951	H	-0.620772	5.612996	-1.079305
H	-4.266738	-1.022240	1.907996	H	-6.646581	-1.624471	1.657526
H	-6.484021	-0.553932	-2.496531	H	-4.100576	0.034336	-2.255559
H	2.335871	-2.522798	1.765719	H	-8.524822	-2.197111	0.159015

H	-8.821356	-0.562619	-0.433902	H	-8.431703	-1.846507	-1.576758
H	5.583085	0.917039	2.222608	H	3.621543	1.362194	3.667940
H	2.069827	-1.687700	-1.222402	H	6.286584	-1.299567	-1.926656
H	6.544360	-0.067977	0.172132	H	0.397727	0.262157	2.955241
H	4.551053	-3.076463	-4.566808	H	5.449559	-1.614011	-4.092433
H	5.740654	-3.163787	-3.244738				

6b$^{\bullet+}$ (cation radical) b3lyp/6-311+g(d,p) scrf=(cpcm,solvent=acetonitrile)

```
# opt freq b3lyp/6-311+g(d,p)
scrf=(cpcm,solvent=acetonitrile)
```

Charge = 1, Multiplicity = 2, Point group = C1

Electronic Energy = -6623.877826 Hartree

Number of imaginary frequencies = 0

Sum of electronic and zero-point Energies = -6623.433456 Hartree

Sum of electronic and thermal Energies = -6623.402244 Hartree

Sum of electronic and thermal Enthalpies = -6623.401300 Hartree

Sum of electronic and thermal Free Energies = -6623.500397 Hartree

	Cartesian Coordinates				Cartesian Coordinates		
	X	*Y*	*Z*		*X*	*Y*	*Z*
C	-1.752121	-2.943919	1.554862	C	-0.473231	-2.364490	1.734251
C	-0.128258	-1.043482	1.282039	C	-1.129407	-0.303478	0.645409
C	-2.448970	-0.893459	0.571119	C	-2.749733	-2.209237	0.982349
C	-1.259094	1.028551	0.024969	C	-2.618078	1.174114	-0.290083
N	-3.313198	-0.005919	0.030238	C	-0.379057	2.107204	-0.314897
C	-0.974700	3.312249	-0.843398	C	-2.371007	3.402938	-1.057131
C	-3.202130	2.338186	-0.804419	C	1.034879	2.062189	-0.219662
C	1.791430	3.149592	-0.583309	C	1.218393	4.350285	-1.053882
C	-0.144436	4.413794	-1.180342	C	-4.717602	-0.213320	-0.165209
C	-5.541139	-0.448888	0.936563	C	-6.902256	-0.652861	0.736697
C	-7.460938	-0.624027	-0.547336	C	-6.609912	-0.387930	-1.635965
C	-5.247478	-0.179737	-1.456155	O	0.411712	-3.125393	2.374795
C	-8.941462	-0.814793	-0.752342	C	1.254202	-0.593625	1.617717
C	2.390651	-1.089065	0.887728	C	3.707740	-0.735793	1.316509
C	3.860239	0.109487	2.447762	C	2.773646	0.587478	3.126247
C	1.464555	0.233728	2.716650	C	2.246642	-1.902889	-0.267474
C	3.364952	-2.350848	-0.928960	C	4.670114	-2.026595	-0.509066
C	4.825392	-1.225587	0.598292	Br	3.152176	-3.449326	-2.489791
O	0.461749	0.744426	3.474780	Br	3.701972	3.049739	-0.475546
H	-1.912040	-3.957225	1.897849	H	-3.742535	-2.615457	0.846712
H	-2.779889	4.327460	-1.446522	H	-4.266529	2.397229	-0.984554

H	1.521156	1.164081	0.117774	H	1.844441	5.191155	-1.320264
H	-0.609779	5.316545	-1.558764	H	-5.124900	-0.454786	1.936548
H	-7.539612	-0.831632	1.595296	H	-7.016362	-0.375450	-2.640993
H	-4.598876	-0.015886	-2.307861	H	1.256421	-2.655070	2.482955
H	-9.377704	-1.423379	0.041789	H	-9.456102	0.152071	-0.746951
H	-9.151086	-1.291043	-1.712121	H	4.859885	0.377553	2.770055
H	2.883340	1.232934	3.988783	H	1.260611	-2.160513	-0.630426
H	5.529150	-2.396460	-1.052520	H	5.821063	-0.956060	0.932301
H	-0.402103	0.454018	3.152255				

7•+ (cation radical) b3lyp/6-311+g(d,p) scrf=(cpcm,solvent=acetonitrile)

```
# opt freq b3lyp/6-311+g(d,p)
scrf=(cpcm,solvent=acetonitrile)
```

Charge = 1, Multiplicity = 2, Point group = C1

Electronic Energy = -1722.757906 Hartree

Number of imaginary frequencies = 0

Sum of electronic and zero-point Energies = -1722.245351 Hartree

Sum of electronic and thermal Energies = -1722.214392 Hartree

Sum of electronic and thermal Enthalpies = -1722.213448 Hartree

Sum of electronic and thermal Free Energies = -1722.308910 Hartree

	Cartesian Coordinates				Cartesian Coordinates		
	X	*Y*	*Z*		*X*	*Y*	*Z*
C	4.079425	-2.921244	2.454837	C	5.443275	-2.615279	2.318526
C	5.876665	-1.567182	1.493570	C	4.928372	-0.803977	0.816002
C	3.552710	-1.109614	0.987791	C	3.124405	-2.176142	1.783178
C	4.997701	0.303296	-0.130906	C	3.675228	0.609267	-0.487254
N	2.798828	-0.248521	0.190893	C	6.067899	1.069519	-0.675696
C	5.728400	2.143758	-1.569918	C	4.363065	2.424040	-1.867766
C	3.338721	1.682880	-1.338359	C	7.440632	0.841623	-0.392354
C	8.422146	1.621802	-0.961051	C	8.085970	2.673881	-1.841343
C	6.765210	2.926173	-2.135667	C	1.392511	-0.236928	0.094170
C	0.608179	-0.237950	1.257611	C	-0.772645	-0.231620	1.164182
C	-1.392511	-0.236928	-0.094171	C	-0.608179	-0.237950	-1.257611
C	0.772645	-0.231621	-1.164182	O	6.315284	-3.393928	3.011266
N	-2.798828	-0.248521	-0.190893	C	-4.079425	-2.921244	-2.454837
C	-5.443275	-2.615279	-2.318526	C	-5.876665	-1.567182	-1.493569
C	-4.928372	-0.803977	-0.816001	C	-3.552710	-1.109614	-0.987791
C	-3.124405	-2.176142	-1.783178	C	-4.997701	0.303296	0.130906
C	-3.675228	0.609267	0.487253	C	-6.067899	1.069519	0.675697
C	-5.728400	2.143758	1.569918	C	-4.363065	2.424040	1.867766

C	-3.338721	1.682880	1.338358	C	-7.440632	0.841622	0.392354
C	-8.422146	1.621802	0.961051	C	-8.085970	2.673881	1.841343
C	-6.765210	2.926173	2.135667	O	-6.315284	-3.393928	-3.011266
H	3.788945	-3.752369	3.084546	H	6.937672	-1.383681	1.395061
H	2.077555	-2.431437	1.874320	H	4.138598	3.254723	-2.526942
H	2.308943	1.926094	-1.559896	H	7.730503	0.043898	0.275307
H	9.463129	1.426326	-0.731185	H	8.869043	3.279985	-2.281466
H	6.498361	3.733940	-2.808370	H	1.079834	-0.222471	2.231027
H	-1.371005	-0.245752	2.065272	H	-1.079834	-0.222471	-2.231028
H	1.371005	-0.245753	-2.065272	H	7.226019	-3.116000	2.850379
H	-3.788945	-3.752369	-3.084546	H	-6.937672	-1.383681	-1.395061
H	-2.077555	-2.431437	-1.874320	H	-4.138597	3.254723	2.526942
H	-2.308943	1.926094	1.559896	H	-7.730503	0.043898	-0.275306
H	-9.463129	1.426326	0.731186	H	-8.869043	3.279985	2.281467
H	-6.498361	3.733941	2.808370	H	-7.226019	-3.116000	-2.850379

10a$^{\bullet+}$ (cation radical) b3lyp/6-311+g(d,p) scrf=(cpcm,solvent=acetonitrile)

```
# opt freq b3lyp/6-311+g(d,p)
scrf=(cpcm,solvent=acetonitrile)
```

Charge = 1, Multiplicity = 2, Point group = C1

Electronic Energy = -785.643912 Hartree

Number of imaginary frequencies = 0

Sum of electronic and zero-point Energies = -785.388914 Hartree

Sum of electronic and thermal Energies = -785.374502 Hartree

Sum of electronic and thermal Enthalpies = -785.373558 Hartree

Sum of electronic and thermal Free Energies = -785.430974 Hartree

	**Cartesian Coordinates**				**Cartesian Coordinates**		
	X	***Y***	***Z***		***X***	***Y***	***Z***
C	-3.652974	-0.924902	0.000084	C	-3.146134	0.418730	-0.000020
C	-1.760197	0.694373	-0.000036	C	-0.877712	-0.369689	0.000063
C	-1.412059	-1.717591	0.000098	C	-2.801740	-1.989699	0.000133
C	0.568407	-0.507896	0.000044	C	0.831054	-1.882635	0.000005
N	-0.385575	-2.580801	0.000058	C	1.658121	0.411743	0.000054
C	2.987569	-0.145019	-0.000098	C	3.178303	-1.551224	-0.000185
C	2.116396	-2.433015	-0.000116	C	1.521328	1.821461	0.000240
C	2.629469	2.639609	0.000216	C	3.934353	2.094875	0.000010
C	4.105697	0.732484	-0.000132	O	-4.086574	1.352405	-0.000102
C	-3.742295	2.756710	-0.000216	H	-4.726888	-1.056690	0.000106
H	-1.416825	1.715973	-0.000161	H	-3.168130	-3.007928	0.000189
H	-0.471579	-3.589536	0.000064	H	4.191570	-1.935739	-0.000299

H	2.270658	-3.504398	-0.000166	H	0.539308	2.272102	0.000443
H	2.499546	3.715576	0.000369	H	4.793351	2.755024	-0.000017
H	5.101336	0.303078	-0.000261	H	-4.694016	3.279423	-0.000196
H	-3.176057	3.004861	0.898215	H	-3.176163	3.004735	-0.898748

10b$^{\bullet+}$ (cation radical) b3lyp/6-311+g(d,p) scrf=(cpcm,solvent=acetonitrile)

```
# opt freq b3lyp/6-311+g(d,p)
scrf=(cpcm,solvent=acetonitrile)
```

Charge = 1, Multiplicity = 2, Point group = C1

Electronic Energy = -824.963742 Hartree

Number of imaginary frequencies = 0

Sum of electronic and zero-point Energies = -824.680929 Hartree

Sum of electronic and thermal Energies = -824.664823 Hartree

Sum of electronic and thermal Enthalpies = -824.663879 Hartree

Sum of electronic and thermal Free Energies = -824.724943 Hartree

	Cartesian Coordinates				Cartesian Coordinates		
	X	***Y***	***Z***		***X***	***Y***	***Z***
C	3.646311	0.551607	-0.000937	C	3.096403	-0.767354	0.000225
C	1.697860	-0.987951	0.000523	C	0.857046	0.106436	-0.000311
C	1.429547	1.433979	-0.000723	C	2.830155	1.646764	-0.001315
C	-0.588207	0.286428	0.000082	C	-0.805695	1.667183	0.000531
N	0.432707	2.341095	-0.000039	C	-1.701196	-0.599675	-0.000363
C	-3.014646	-0.005358	0.000755	C	-3.164284	1.406079	0.001506
C	-2.077796	2.254396	0.001234	C	-1.604129	-2.015063	-0.002099
C	-2.733515	-2.800800	-0.002022	C	-4.022729	-2.217806	-0.000263
C	-4.156224	-0.851206	0.000943	C	0.561619	3.794660	-0.000669
O	3.997468	-1.741650	0.001043	C	3.592252	-3.128730	0.001649
H	4.723574	0.651516	-0.001460	H	1.315955	-1.995775	0.001777
H	3.249444	2.643410	-0.002163	H	-4.165750	1.820240	0.002131
H	-2.208562	3.328343	0.001646	H	-0.635148	-2.492558	-0.003908
H	-2.634926	-3.880061	-0.003436	H	-4.900262	-2.853235	-0.000030
H	-5.139673	-0.394592	0.001971	H	0.083961	4.203254	-0.892237
H	0.082684	4.204075	0.889824	H	1.611685	4.069686	-0.000054
H	4.519895	-3.693295	0.001093	H	3.016581	-3.353270	0.900421
H	3.015210	-3.353740	-0.896116				

10c$^{\bullet+}$ (cation radical) b3lyp/6-311+g(d,p) scrf=(cpcm,solvent=acetonitrile)

```
# opt freq b3lyp/6-311+g(d,p)
scrf=(cpcm,solvent=acetonitrile)
```

Charge = 1, Multiplicity = 2, Point group = C1

Electronic Energy = -1056.073553 Hartree

Number of imaginary frequencies = 0

Sum of electronic and zero-point Energies = -1055.711504 Hartree

Sum of electronic and thermal Energies = -1055.690455 Hartree

Sum of electronic and thermal Enthalpies = -1055.689511 Hartree

Sum of electronic and thermal Free Energies = -1055.763125 Hartree

	**Cartesian Coordinates**				**Cartesian Coordinates**		
	X	***Y***	***Z***		***X***	***Y***	***Z***
C	0.051765	3.542194	0.103243	C	1.457684	3.292109	0.070521
C	1.977027	1.975804	0.037650	C	1.090623	0.916443	0.023618
C	-0.324125	1.190194	0.038031	C	-0.839438	2.506327	0.091736
C	1.227220	-0.533977	0.007418	C	-0.070956	-1.050717	0.009485
N	-1.001895	0.017357	0.030276	C	2.336684	-1.424931	-0.027335
C	2.046659	-2.836404	-0.064159	C	0.702884	-3.292336	-0.074565
C	-0.363497	-2.419678	-0.042890	C	3.695612	-1.018416	-0.029555
C	4.710871	-1.946528	-0.062890	C	4.426433	-3.332099	-0.097181
C	3.122867	-3.763293	-0.097975	C	-2.425515	-0.130586	0.011116
C	-3.161796	0.397643	-1.050291	C	-4.544926	0.249436	-1.060592
C	-5.212380	-0.419294	-0.026965	C	-4.448130	-0.945396	1.023838
C	-3.065281	-0.807516	1.051219	C	-6.713639	-0.550734	-0.029938
O	2.214122	4.383374	0.085011	C	3.655430	4.287533	0.066886
H	-0.279513	4.571293	0.145339	H	3.043708	1.822383	0.029033
H	-1.905283	2.685594	0.128448	H	0.518936	-4.359703	-0.112758
H	-1.383284	-2.778995	-0.062675	H	3.948442	0.031286	-0.001935
H	5.741828	-1.612333	-0.062247	H	5.239570	-4.047630	-0.123129
H	2.893585	-4.822692	-0.125323	H	-2.656716	0.896688	-1.868383
H	-5.111393	0.652051	-1.892756	H	-4.941514	-1.466407	1.836752
H	-2.490143	-1.204902	1.878405	H	-7.115221	-0.502325	-1.043639
H	-7.030732	-1.490998	0.425697	H	-7.170467	0.261746	0.545282
H	4.006686	5.314955	0.085358	H	4.009476	3.753928	0.949902
H	3.989875	3.792907	-0.845937				

$12^{•+}$ (cation radical) b3lyp/6-311+g(d,p) scrf=(cpcm,solvent=acetonitrile)

```
# opt freq b3lyp/6-311+g(d,p)
scrf=(cpcm,solvent=acetonitrile)
```

Charge = 1, Multiplicity = 2, Point group = C1

Electronic Energy = -5664.479198 Hartree

Number of imaginary frequencies = 0

Sum of electronic and zero-point Energies = -5664.324482 Hartree

Sum of electronic and thermal Energies = -5664.312462 Hartree

Sum of electronic and thermal Enthalpies = -5664.311518 Hartree

Sum of electronic and thermal Free Energies = -5664.365693 Hartree

	Cartesian Coordinates				Cartesian Coordinates		
	X	*Y*	*Z*		*X*	*Y*	*Z*
C	-1.128874	1.915007	0.000016	C	-0.731390	0.547471	0.000017
C	0.731390	0.547472	0.000005	C	1.128874	1.915007	-0.000010
N	-0.000002	2.692097	-0.000000	C	-2.471668	2.309269	0.000020
C	-3.433779	1.308787	0.000013	C	-3.044608	-0.037609	0.000006
C	-1.692574	-0.440805	0.000008	C	1.692575	-0.440803	0.000016
C	3.044609	-0.037607	-0.000001	C	3.433778	1.308788	-0.000024
C	2.471666	2.309270	-0.000027	Br	-4.381771	-1.380587	-0.000010
Br	4.381772	-1.380587	0.000004	H	-0.000002	3.707307	-0.000003
H	-2.753331	3.354762	0.000021	H	-4.483625	1.568250	0.000013
H	-1.433690	-1.491205	-0.000001	H	1.433691	-1.491203	0.000034
H	4.483624	1.568252	-0.000040	H	2.753329	3.354763	-0.000041

3a$^{\bullet+}$ (cation radical) b3lyp/6-31+g(d,p) scrf=(cpcm,solvent=acetonitrile)

opt freq b3lyp/6-31+g(d,p) scrf=(cpcm,solvent=acetonitrile)

Charge = 1, Multiplicity = 2, Point group = C1

Electronic Energy = −1016.575430 Hartree

Number of imaginary frequencies = 0

Sum of electronic and zero-point Energies = −1016.240523 Hartree

Sum of electronic and thermal Energies = −1016.221084 Hartree

Sum of electronic and thermal Enthalpies = −1016.220140 Hartree

Sum of electronic and thermal Free Energies = −1016.290347 Hartree

Atoms	Cartesian Coordinates			Atoms	Cartesian Coordinates		
	X	*Y*	*Z*		*X*	*Y*	*Z*
C	−0.349059	3.751828	0.131132	C	1.068188	3.715353	0.091286
C	1.788950	2.492355	0.052071	C	1.081156	1.305594	0.033180
C	−0.355124	1.359611	0.047342	C	−1.068606	2.577576	0.116451
C	1.441331	−0.112468	0.013982	C	0.234115	−0.824091	0.012973
N	−0.846038	0.086065	0.034166	C	2.674860	−0.819994	−0.024675
C	2.606742	−2.262861	−0.072575	C	1.346337	−2.924047	−0.091229
C	0.156697	−2.227621	−0.054181	C	3.957210	−0.203630	−0.020356
C	5.106960	−0.963288	−0.057853	C	5.039704	−2.379284	−0.103354
C	3.815250	−3.010746	−0.110459	C	−2.226756	−0.277011	0.010949
C	−3.055161	0.208596	−1.007428	C	−4.403102	−0.148867	−1.019299
C	−4.944956	−0.984393	−0.030566	C	−4.090156	−1.457992	0.981543
C	−2.741681	−1.116151	1.009170	O	1.693211	4.900618	0.106108

C	−6.401126	−1.376505	−0.048568	H	−0.843624	4.714586	0.182318
H	2.871152	2.524616	0.044099	H	−2.150662	2.589756	0.163528
H	1.330806	−4.008274	−0.140929	H	−0.795905	−2.741994	−0.082821
H	4.041814	0.874200	0.017083	H	6.075091	−0.472295	−0.051518
H	5.955319	−2.961351	−0.132487	H	3.754945	−4.094654	−0.146071
H	−2.645255	0.834309	−1.793195	H	−5.039884	0.222446	−1.816643
H	−4.488379	−2.097060	1.764386	H	−2.100191	−1.471470	1.808399
H	2.659200	4.804100	0.082528	H	−6.944127	−0.859522	−0.843310
H	−6.511918	−2.455411	−0.207403	H	−6.882439	−1.141182	0.906798

3b$^{•+}$ (cation radical) b3lyp/6-31+g(d,p) scrf=(cpcm,solvent=acetonitrile)

opt freq b3lyp/6-31+g(d,p) scrf=(cpcm,solvent=acetonitrile)

Charge = 1, Multiplicity = 2, Point group = C1

Electronic Energy = −1436.845660 Hartree

Number of imaginary frequencies = 0

Sum of electronic and zero-point Energies = −1436.547362 Hartree

Sum of electronic and thermal Energies = −1436.528641 Hartree

Sum of electronic and thermal Enthalpies = −1436.527696 Hartree

Sum of electronic and thermal Free Energies = −1436.595762 Hartree

Atoms	Cartesian Coordinates			Atoms	Cartesian Coordinates		
	X	*Y*	*Z*		*X*	*Y*	*Z*
C	−0.167093	3.810137	0.119937	C	−1.584095	3.690531	0.084254
C	−2.235216	2.429281	0.047507	C	−1.460984	1.285229	0.032534
C	−0.026253	1.423805	0.048334	C	0.618625	2.682697	0.107008
C	−1.736533	−0.149172	0.011759	C	−0.490774	−0.788580	0.011860
N	0.535442	0.185566	0.035578	C	−2.926798	−0.929498	−0.024844
C	−2.771977	−2.365925	−0.068153	C	−1.475327	−2.950305	−0.081103
C	−0.327917	−2.183194	−0.045320	C	−4.243097	−0.391790	−0.022421
C	−5.344899	−1.220146	−0.058790	C	−5.193470	−2.629844	−0.100728
C	−3.933767	−3.186137	−0.105008	C	1.938046	−0.097107	0.016905
C	2.509086	−0.823615	1.067758	C	3.875659	−1.096536	1.051369
C	4.649625	−0.639194	−0.017774	C	4.085686	0.084258	−1.070390
C	2.718399	0.355900	−1.052718	O	−2.270073	4.838267	0.097660
Cl	6.370307	−0.983845	−0.038781	H	0.268029	4.801441	0.164449
H	−3.317383	2.400286	0.036184	H	1.698718	2.755814	0.146068
H	−1.394456	−4.031911	−0.124231	H	0.654249	−2.639700	−0.066674
H	−4.393627	0.679071	0.011805	H	−6.340705	−0.788042	−0.054504
H	−6.072761	−3.265392	−0.129191	H	−3.807644	−4.264405	−0.137434
H	1.899276	−1.156262	1.900489	H	4.331423	−1.650380	1.863806

H	4.699150	0.424230	−1.896588	H	2.261779	0.897786	−1.873884
H	−3.230326	4.692958	0.076802				

3c$^{\bullet+}$ (cation radical) b3lyp/6-31+g(d,p) scrf=(cpcm,solvent=acetonitrile)

opt freq b3lyp/6-31+g(d,p) scrf=(cpcm,solvent=acetonitrile)

Charge = 1, Multiplicity = 2, Point group = C1

Electronic Energy = −1055.898346 Hartree

Number of imaginary frequencies = 0

Sum of electronic and zero-point Energies = −1055.535668 Hartree

Sum of electronic and thermal Energies = −1055.514474 Hartree

Sum of electronic and thermal Enthalpies = −1055.513529 Hartree

Sum of electronic and thermal Free Energies = −1055.588709 Hartree

Atoms	Cartesian Coordinates			Atoms	Cartesian Coordinates		
	X	*Y*	*Z*		*X*	*Y*	*Z*
C	0.562573	3.500697	0.088878	C	−0.875197	3.539477	0.051150
C	−1.681392	2.376688	0.024834	C	−1.065655	1.139489	0.018401
C	0.377784	1.099002	0.034088	C	1.174712	2.267225	0.083501
C	−1.518016	−0.245690	0.008049	C	−0.362585	−1.035450	0.013328
N	0.782587	−0.196156	0.031054	C	−2.798329	−0.871327	−0.023499
C	−2.826109	−2.315232	−0.054686	C	−1.613694	−3.056304	−0.062882
C	−0.377249	−2.437192	−0.033675	C	−4.035519	−0.172805	−0.027401
C	−5.233927	−0.855972	−0.057403	C	−5.262463	−2.273465	−0.086490
C	−4.082841	−2.983426	−0.085180	C	2.137810	−0.655078	0.008275
C	2.614684	−1.463655	1.047583	C	3.936776	−1.900916	1.018787
C	4.800394	−1.549980	−0.033708	C	4.296854	−0.738797	−1.062093
C	2.975461	−0.291391	−1.051289	O	−1.419397	4.761998	0.053831
C	6.223014	−2.052018	−0.060894	C	1.338744	4.786778	0.140926
H	−2.758488	2.492044	0.014886	H	2.255131	2.191856	0.122197
H	−1.667708	−4.140091	−0.096544	H	0.539682	−3.013689	−0.051152
H	−4.048289	0.908862	−0.003587	H	−6.166988	−0.300943	−0.058124
H	−6.214975	−2.793227	−0.109942	H	−4.092925	−4.069328	−0.108243
H	1.965928	−1.726708	1.876465	H	4.306080	−2.518069	1.832853
H	4.942544	−0.456521	−1.888487	H	2.593831	0.317817	−1.864124
H	−2.389596	4.726216	0.033129	H	6.691594	−1.967115	0.924610
H	6.254487	−3.111072	−0.344190	H	6.828511	−1.496087	−0.781149
H	1.127505	5.408002	−0.736083	H	1.064725	5.375443	1.022908
H	2.411039	4.585756	0.174970				

3d$^{\bullet+}$ (cation radical) b3lyp/6-31+g(d,p) scrf=(cpcm,solvent=acetonitrile)

# opt freq b3lyp/6-31+g(d,p) scrf=(cpcm,solvent=acetonitrile)
Charge = 1, Multiplicity = 2, Point group = C1
Electronic Energy = −3587.702230 Hartree
Number of imaginary frequencies = 0
Sum of electronic and zero-point Energies = −3587.376743 Hartree
Sum of electronic and thermal Energies = −3587.355928 Hartree
Sum of electronic and thermal Enthalpies = −3587.354984 Hartree
Sum of electronic and thermal Free Energies = −3587.429487 Hartree

Atoms	Cartesian Coordinates			Atoms	Cartesian Coordinates		
	X	*Y*	*Z*		*X*	*Y*	*Z*
C	−1.525579	3.753949	0.133713	C	−0.104069	3.731844	0.096582
C	0.630004	2.518856	0.061048	C	−0.065383	1.324207	0.044010
C	−1.505579	1.362917	0.056189	C	−2.233017	2.575683	0.120121
C	0.307237	−0.086809	0.025808	C	−0.891211	−0.811247	0.021796
N	−1.983834	0.089197	0.042092	C	1.547207	−0.785316	−0.010544
C	1.491876	−2.226713	−0.057930	C	0.241080	−2.899799	−0.077742
C	−0.956415	−2.212737	−0.043462	C	2.818021	−0.151159	−0.002676
C	3.963282	−0.912824	−0.038127	C	3.928746	−2.327076	−0.083653
C	2.708706	−2.961751	−0.092611	C	−3.361126	−0.290203	0.011209
C	−4.184721	0.171076	−1.021821	C	−5.528855	−0.200782	−1.040341
C	−6.070383	−1.028249	−0.044790	C	−5.219977	−1.477701	0.981729
C	−3.875578	−1.119855	1.016995	Br	5.663078	−0.049538	−0.024814
O	0.503105	4.924389	0.109864	C	−7.521194	−1.439569	−0.072541
H	−2.027825	4.712903	0.181886	H	1.711684	2.564282	0.053751
H	−3.315228	2.575592	0.163603	H	0.235299	−3.984015	−0.125376
H	−1.904236	−2.735793	−0.072381	H	2.896972	0.925369	0.034717
H	4.850663	−2.896031	−0.110459	H	2.664497	−4.046071	−0.127712
H	−3.774508	0.790909	−1.812202	H	−6.162611	0.152848	−1.848090
H	−5.618307	−2.110032	1.769975	H	−3.237532	−1.456636	1.827042
H	1.470745	4.842563	0.088166	H	−8.070102	−0.914533	−0.857940
H	−7.616553	−2.516463	−0.254330	H	−8.007121	−1.231019	0.886505

3e$^{•+}$ (cation radical) b3lyp/6-31+g(d,p) scrf=(cpcm,solvent=acetonitrile)

# opt freq b3lyp/6-31+g(d,p) scrf=(cpcm,solvent=acetonitrile)
Charge = 1, Multiplicity = 2, Point group = C1
Electronic Energy = −1108.821600 Hartree
Number of imaginary frequencies = 0
Sum of electronic and zero-point Energies = −1108.487790 Hartree
Sum of electronic and thermal Energies = −1108.466544 Hartree

Sum of electronic and thermal Enthalpies = −1108.465600 Hartree
Sum of electronic and thermal Free Energies = −1108.540160 Hartree

Atoms	Cartesian Coordinates			Atoms	Cartesian Coordinates		
	X	*Y*	*Z*		*X*	*Y*	*Z*
C	−0.817663	3.752767	0.135541	C	0.602124	3.718636	0.093158
C	1.325896	2.496400	0.055286	C	0.620053	1.309413	0.040110
C	−0.818123	1.360611	0.056105	C	−1.534584	2.579691	0.124076
C	0.980067	−0.107303	0.021468	C	−0.224673	−0.819598	0.021402
N	−1.308285	0.090578	0.043485	C	2.212134	−0.820966	−0.016888
C	2.145414	−2.261549	−0.064046	C	0.885995	−2.922322	−0.081581
C	−0.301534	−2.222165	−0.044296	C	3.487272	−0.202870	−0.012287
C	4.641885	−0.970512	−0.049968	C	4.579186	−2.393343	−0.095359
C	3.352975	−3.012084	−0.101640	C	−2.688744	−0.275474	0.013249
C	−3.510728	0.203989	−1.013196	C	−4.858056	−0.155176	−1.031636
C	−5.404804	−0.986625	−0.042135	C	−4.555847	−1.454172	0.977890
C	−3.208128	−1.110339	1.012577	O	1.221589	4.903218	0.104752
C	−6.859811	−1.381993	−0.068103	C	5.920806	−0.320864	−0.041975
N	6.962922	0.198378	−0.036052	H	−1.312162	4.715533	0.186441
H	2.407835	2.533463	0.045040	H	−2.616509	2.588866	0.172989
H	0.868665	−4.006166	−0.129969	H	−1.254697	−2.735637	−0.071265
H	3.578211	0.873220	0.024983	H	5.495278	−2.972036	−0.124466
H	3.292811	−4.095303	−0.136662	H	−3.096484	0.826135	−1.799573
H	−5.490351	0.211408	−1.834669	H	−4.958337	−2.089680	1.761395
H	−2.571623	−1.460441	1.818127	H	2.188619	4.813399	0.080302
H	−7.401407	−0.860235	−0.860641	H	−6.966941	−2.459917	−0.236192
H	−7.344752	−1.155960	0.887588				

6a$^{\bullet+}$ (cation radical) b3lyp/6-31+g(d,p) scrf=(cpcm,solvent=acetonitrile)

# opt freq b3lyp/6-31+g(d,p) scrf=(cpcm,solvent=acetonitrile)
Charge = 1, Multiplicity = 2, Point group = C1
Electronic Energy = −1591.047236 Hartree
Number of imaginary frequencies = 0
Sum of electronic and zero-point Energies = −1590.548812 Hartree
Sum of electronic and thermal Energies = −1590.518003 Hartree
Sum of electronic and thermal Enthalpies = −1590.517059 Hartree
Sum of electronic and thermal Free Energies = −1590.613131 Hartree

Atoms	Cartesian Coordinates			Atoms	Cartesian Coordinates		
	X	*Y*	*Z*		*X*	*Y*	*Z*
C	−0.683937	−3.049886	0.985497	C	0.535591	−2.358738	1.161110

C	0.702397	−0.952142	0.879610	C	−0.420794	−0.255377	0.414887
C	−1.670292	−0.973913	0.343924	C	−1.800346	−2.357264	0.586493
C	−0.734102	1.126422	−0.015720	C	−2.124562	1.156949	−0.223900
N	−2.665490	−0.120793	−0.020767	C	0.007948	2.325446	−0.269453
C	−0.742801	3.518927	−0.600646	C	−2.160480	3.482743	−0.709848
C	−2.861837	2.311964	−0.545199	C	1.426349	2.424568	−0.270793
C	2.064654	3.618514	−0.532943	C	1.324448	4.793775	−0.809013
C	−0.050941	4.736196	−0.845881	C	−4.049154	−0.461879	−0.158391
C	−4.754194	−0.953572	0.944641	C	−6.101873	−1.284721	0.800214
C	−6.761737	−1.134305	−0.428685	C	−6.027970	−0.638073	−1.521353
C	−4.683477	−0.299206	−1.396768	O	1.550477	−3.086711	1.636531
C	−8.221200	−1.484489	−0.581668	C	2.043007	−0.384252	1.199274
C	3.178680	−0.645300	0.346581	C	4.470223	−0.160105	0.741761
C	4.595925	0.572971	1.953289	C	3.507353	0.824376	2.751315
C	2.227752	0.341090	2.376316	C	3.058663	−1.339214	−0.880132
C	4.171434	−1.578003	−1.677704	C	5.452509	−1.115192	−1.281782
C	5.582170	−0.418627	−0.097400	O	1.221988	0.631578	3.247043
O	3.941748	−2.261585	−2.833200	C	5.040813	−2.544395	−3.708524
H	−0.715472	−4.112589	1.193921	H	−2.754447	−2.854320	0.460673
H	−2.688067	4.400941	−0.947981	H	−3.939439	2.277692	−0.647796
H	2.019072	1.543899	−0.084105	H	3.149687	3.654046	−0.532473
H	1.841031	5.728200	−1.004111	H	−0.633713	5.622517	−1.079422
H	−4.261946	−1.055168	1.906110	H	−6.647739	−1.659286	1.660953
H	−6.514149	−0.521940	−2.485776	H	−4.124936	0.066373	−2.251943
H	2.346829	−2.533629	1.746884	H	−8.621410	−1.935257	0.329678
H	−8.814530	−0.590897	−0.807359	H	−8.371698	−2.187638	−1.407904
H	5.576338	0.938317	2.245546	H	3.597608	1.380989	3.677777
H	2.092974	−1.690791	−1.227561	H	6.324728	−1.294402	−1.897485
H	6.559386	−0.051881	0.203913	H	0.377073	0.262649	2.947062
H	4.611349	−3.090313	−4.548087	H	5.504403	−1.621145	−4.071298
H	5.790486	−3.168794	−3.211493				

6b$^{\bullet+}$ (cation radical) b3lyp/6-31+g(d,p) scrf=(cpcm,solvent=acetonitrile)

opt freq b3lyp/6-31+g(d,p) scrf=(cpcm,solvent=acetonitrile)

Charge = 1, Multiplicity = 2, Point group = C1

Electronic Energy = −6618.773518 Hartree

Number of imaginary frequencies = 0

Sum of electronic and zero-point Energies = −6618.326726 Hartree

Sum of electronic and thermal Energies = −6618.295807 Hartree

Sum of electronic and thermal Enthalpies = −6618.294863 Hartree

Sum of electronic and thermal Free Energies = −6618.392929 Hartree

Atoms	Cartesian Coordinates			Atoms	Cartesian Coordinates		
	X	Y	Z		X	Y	Z
C	−1.772260	−2.997254	1.477407	C	−0.496978	−2.414095	1.688682
C	−0.152866	−1.082545	1.265782	C	−1.150697	−0.338993	0.621832
C	−2.471344	−0.923751	0.529470	C	−2.771980	−2.251150	0.911032
C	−1.270861	1.002491	0.024253	C	−2.630856	1.168653	−0.282917
N	−3.333873	−0.016745	0.007482	C	−0.375740	2.072484	−0.303051
C	−0.956001	3.307320	−0.781607	C	−2.356005	3.424818	−0.977162
C	−3.203055	2.359524	−0.757530	C	1.038493	1.989226	−0.241017
C	1.816773	3.075854	−0.566946	C	1.261661	4.308347	−0.981262
C	−0.105163	4.405390	−1.089076	C	−4.737300	−0.212055	−0.196037
C	−5.561354	−0.510817	0.894898	C	−6.925805	−0.704688	0.684971
C	−7.487876	−0.603046	−0.597701	C	−6.636579	−0.303199	−1.675177
C	−5.270993	−0.103916	−1.485798	O	0.385622	−3.184012	2.329816
C	−8.970118	−0.782483	−0.812469	C	1.211815	−0.604720	1.635902
C	2.375747	−1.043376	0.908983	C	3.673767	−0.614621	1.335987
C	3.780860	0.239168	2.468588	C	2.667709	0.657474	3.151078
C	1.378076	0.231666	2.739249	C	2.274580	−1.865883	−0.245951
C	3.417143	−2.252266	−0.910697	C	4.705108	−1.855503	−0.492081
C	4.817883	−1.044224	0.616977	Br	3.261153	−3.375398	−2.445708
O	0.347762	0.695918	3.495079	Br	3.715168	2.906365	−0.504377
H	−1.932734	−4.020316	1.795922	H	−3.763270	−2.660150	0.759349
H	−2.757209	4.369390	−1.330034	H	−4.269105	2.440672	−0.930106
H	1.511561	1.065003	0.042387	H	1.902164	5.148223	−1.223417
H	−0.556088	5.332313	−1.430083	H	−5.143445	−0.571313	1.894373
H	−7.563141	−0.932510	1.534268	H	−7.045976	−0.232759	−2.678650
H	−4.622669	0.108683	−2.329307	H	1.227508	−2.710252	2.466020
H	−9.413327	−1.408101	−0.033136	H	−9.482118	0.187491	−0.788297
H	−9.178792	−1.236870	−1.785240	H	4.765916	0.562477	2.791411
H	2.743085	1.310548	4.013745	H	1.303148	−2.180960	−0.608054
H	5.584230	−2.181178	−1.035627	H	5.798984	−0.719183	0.950647
H	−0.504629	0.363920	3.173600				

$7^{\bullet +}$ (cation radical) b3lyp/6-31+g(d,p) scrf=(cpcm,solvent=acetonitrile)

opt freq b3lyp/6-31+g(d,p) scrf=(cpcm,solvent=acetonitrile)

Charge = 1, Multiplicity = 2, Point group = C1

Electronic Energy = −1722.433751 Hartree

Number of imaginary frequencies = 0
Sum of electronic and zero-point Energies = −1721.919463 Hartree
Sum of electronic and thermal Energies = −1721.888565 Hartree
Sum of electronic and thermal Enthalpies = −1721.887620 Hartree
Sum of electronic and thermal Free Energies = −1721.982916 Hartree

Atoms	Cartesian Coordinates			Atoms	Cartesian Coordinates		
	X	***Y***	***Z***		***X***	***Y***	***Z***
C	4.084889	−2.931632	2.453491	C	5.451520	−2.624244	2.316284
C	5.886582	−1.571910	1.492484	C	4.935756	−0.806274	0.814936
C	3.557805	−1.112620	0.987218	C	3.127475	−2.182917	1.781929
C	5.004352	0.304431	−0.129962	C	3.678827	0.611103	−0.485418
N	2.802430	−0.249008	0.191786	C	6.076017	1.072875	−0.675199
C	5.734489	2.151918	−1.567628	C	4.366373	2.433395	−1.864142
C	3.339585	1.688613	−1.335025	C	7.451340	0.843125	−0.393499
C	8.435188	1.626607	−0.962963	C	8.097398	2.683319	−1.841828
C	6.772373	2.936968	−2.134074	C	1.395512	−0.238155	0.094750
C	0.609476	−0.239042	1.261063	C	−0.774987	−0.233370	1.167022
C	−1.395512	−0.238155	−0.094750	C	−0.609476	−0.239042	−1.261063
C	0.774987	−0.233370	−1.167022	O	6.324729	−3.406174	3.008367
N	−2.802430	−0.249008	−0.191786	C	−4.084889	−2.931632	−2.453491
C	−5.451519	−2.624244	−2.316284	C	−5.886582	−1.571910	−1.492484
C	−4.935756	−0.806274	−0.814936	C	−3.557805	−1.112620	−0.987218
C	−3.127475	−2.182917	−1.781929	C	−5.004352	0.304431	0.129962
C	−3.678827	0.611103	0.485418	C	−6.076017	1.072875	0.675199
C	−5.734489	2.151918	1.567628	C	−4.366373	2.433394	1.864142
C	−3.339586	1.688613	1.335025	C	−7.451340	0.843125	0.393498
C	−8.435188	1.626607	0.962963	C	−8.097398	2.683319	1.841828
C	−6.772373	2.936968	2.134074	O	−6.324729	−3.406174	−3.008367
H	3.793787	−3.765272	3.082645	H	6.949064	−1.387045	1.393779
H	2.078737	−2.438280	1.873205	H	4.140610	3.267203	−2.522022
H	2.307895	1.932643	−1.555819	H	7.741757	0.041850	0.272586
H	9.478013	1.429144	−0.734205	H	8.880901	3.291752	−2.282678
H	6.504028	3.747876	−2.805524	H	1.081810	−0.222959	2.236278
H	−1.374716	−0.247800	2.069515	H	−1.081810	−0.222959	−2.236278
H	1.374716	−0.247800	−2.069515	H	7.239509	−3.129797	2.849076
H	−3.793787	−3.765272	−3.082645	H	−6.949064	−1.387045	−1.393779
H	−2.078737	−2.438280	−1.873205	H	−4.140610	3.267203	2.522023
H	−2.307895	1.932643	1.555819	H	−7.741757	0.041850	−0.272586
H	−9.478013	1.429144	0.734204	H	−8.880901	3.291752	2.282678
H	−6.504028	3.747876	2.805524	H	−7.239508	−3.129797	−2.849076

10a$^{•+}$ (cation radical) b3lyp/6-31+g(d,p) scrf=(cpcm,solvent=acetonitrile)

opt freq b3lyp/6-31+g(d,p) scrf=(cpcm,solvent=acetonitrile)

Charge = 1, Multiplicity = 2, Point group = C1

Electronic Energy = −785.496514 Hartree

Number of imaginary frequencies = 0

Sum of electronic and zero-point Energies = −785.240503 Hartree

Sum of electronic and thermal Energies = −785.226140 Hartree

Sum of electronic and thermal Enthalpies = −785.225196 Hartree

Sum of electronic and thermal Free Energies = −785.282460 Hartree

Atoms	Cartesian Coordinates			Atoms	Cartesian Coordinates		
	X	*Y*	*Z*		*X*	*Y*	*Z*
C	−3.659996	−0.926377	0.000079	C	−3.151492	0.418974	−0.000021
C	−1.763103	0.696941	−0.000035	C	−0.878469	−0.369688	0.000061
C	−1.413735	−1.719508	0.000094	C	−2.805598	−1.993963	0.000127
C	0.569143	−0.508115	0.000044	C	0.831697	−1.885823	0.000007
N	−0.385446	−2.584329	0.000057	C	1.660832	0.412944	0.000053
C	2.992824	−0.145763	−0.000094	C	3.183721	−1.554568	−0.000178
C	2.118988	−2.438719	−0.000111	C	1.523751	1.825197	0.000233
C	2.635131	2.645364	0.000208	C	3.942664	2.099117	0.000007
C	4.113432	0.732747	−0.000129	O	−4.095072	1.355030	−0.000101
C	−3.750385	2.759602	−0.000205	H	−4.735556	−1.059289	0.000100
H	−1.419232	1.720203	−0.000158	H	−3.172198	−3.014041	0.000181
H	−0.471609	−3.594086	0.000064	H	4.198553	−1.940204	−0.000288
H	2.273438	−3.511938	−0.000159	H	0.539899	2.276145	0.000434
H	2.504495	3.723136	0.000356	H	4.803797	2.759605	−0.000021
H	5.110600	0.302016	−0.000254	H	−4.703888	3.282982	−0.000188
H	−3.182968	3.007741	0.899718	H	−3.183062	3.007625	−0.900219

10b$^{•+}$ (cation radical) b3lyp/6-31+g(d,p) scrf=(cpcm,solvent=acetonitrile)

opt freq b3lyp/6-31+g(d,p) scrf=(cpcm,solvent=acetonitrile)

Charge = 1, Multiplicity = 2, Point group = C1

Electronic Energy = −824.809751 Hartree

Number of imaginary frequencies = 0

Sum of electronic and zero-point Energies = −824.525810 Hartree

Sum of electronic and thermal Energies = −824.509767 Hartree

Sum of electronic and thermal Enthalpies = −824.508823 Hartree

Sum of electronic and thermal Free Energies = −824.569692 Hartree

Atoms	Cartesian Coordinates	Atoms	Cartesian Coordinates

	X	Y	Z		X	Y	Z
C	3.653387	0.553883	−0.000346	C	3.102368	−0.766846	0.000014
C	1.701194	−0.989957	0.000215	C	0.858042	0.106739	0.000044
C	1.430851	1.436085	−0.000148	C	2.833620	1.651523	−0.000408
C	−0.589025	0.286329	0.000162	C	−0.806784	1.669889	0.000176
N	0.431533	2.344567	−0.000019	C	−1.703522	−0.601554	0.000037
C	−3.019700	−0.005758	0.000160	C	−3.170073	1.408356	0.000341
C	−2.081182	2.259251	0.000311	C	−1.605520	−2.019526	−0.000304
C	−2.737769	−2.807696	−0.000446	C	−4.029810	−2.223581	−0.000238
C	−4.163299	−0.853054	0.000049	C	0.558401	3.798508	−0.000149
O	4.006496	−1.743480	0.000143	C	3.601190	−3.130934	0.000397
H	4.732273	0.655381	−0.000525	H	1.318938	−1.999492	0.000562
H	3.253134	2.649996	−0.000644	H	−4.173298	1.823151	0.000483
H	−2.212349	3.334971	0.000465	H	−0.634444	−2.496838	−0.000528
H	−2.638121	−3.888748	−0.000745	H	−4.909224	−2.859660	−0.000329
H	−5.148474	−0.395583	0.000177	H	0.078517	4.207264	−0.892628
H	0.078456	4.207399	0.892233	H	1.609501	4.075689	−0.000119
H	4.530740	−3.695982	0.000210	H	3.023964	−3.355698	0.900346
H	3.023470	−3.355923	−0.899175				

10c$^{\bullet+}$ (cation radical) b3lyp/6-31+g(d,p) scrf=(cpcm,solvent=acetonitrile)

opt freq b3lyp/6-31+g(d,p) scrf=(cpcm,solvent=acetonitrile)

Charge = 1, Multiplicity = 2, Point group = C1

Electronic Energy = −1055.878435 Hartree

Number of imaginary frequencies = 0

Sum of electronic and zero-point Energies = −1055.515141 Hartree

Sum of electronic and thermal Energies = −1055.494100 Hartree

Sum of electronic and thermal Enthalpies = −1055.493156 Hartree

Sum of electronic and thermal Free Energies = −1055.566980 Hartree

Atoms	Cartesian Coordinates			Atoms	Cartesian Coordinates		
	X	Y	Z		X	Y	Z
C	0.051064	3.549064	0.105790	C	1.458568	3.298041	0.072034
C	1.980344	1.979425	0.037908	C	1.091854	0.917568	0.023704
C	−0.324203	1.191531	0.038073	C	−0.842107	2.509130	0.093864
C	1.228858	−0.534749	0.006368	C	−0.072098	−1.052216	0.007669
N	−1.003578	0.015884	0.029114	C	2.340314	−1.426763	−0.027840
C	2.049553	−2.841217	−0.066680	C	0.703277	−3.298352	−0.079657
C	−0.366048	−2.424021	−0.047413	C	3.701583	−1.018712	−0.027219
C	4.719931	−1.948969	−0.060027	C	4.435065	−3.337474	−0.096626

C	3.127667	−3.769616	−0.099954	C	−2.426695	−0.131899	0.010194
C	−3.166758	0.413345	−1.044791	C	−4.553448	0.265254	−1.053970
C	−5.220759	−0.419722	−0.026286	C	−4.452358	−0.962597	1.017929
C	−3.066230	−0.825266	1.044904	C	−6.723697	−0.551956	−0.027309
O	2.217386	4.392523	0.086840	C	3.658905	4.295620	0.066934
H	−0.282126	4.579356	0.149049	H	3.048688	1.825618	0.028892
H	−1.909745	2.688318	0.131300	H	0.519054	−4.367486	−0.119991
H	−1.387320	−2.784412	−0.069058	H	3.953841	0.032895	0.002527
H	5.752377	−1.613288	−0.057013	H	5.249286	−4.054682	−0.122137
H	2.898050	−4.830869	−0.128886	H	−2.662441	0.924424	−1.858410
H	−5.122685	0.681257	−1.880063	H	−4.945255	−1.496064	1.825593
H	−2.488615	−1.234708	1.866929	H	−7.133252	−0.442132	−1.034914
H	−7.038034	−1.520585	0.372334	H	−7.178876	0.223200	0.601515
H	4.011112	5.324731	0.084727	H	4.014080	3.760906	0.951131
H	3.992339	3.799440	−0.847639				

$12^{\bullet+}$ (cation radical) b3lyp/6-31+g(d,p) scrf=(cpcm,solvent=acetonitrile)

opt freq b3lyp/6-31+g(d,p) scrf=(cpcm,solvent=acetonitrile)

Charge = 1, Multiplicity = 2, Point group = C1

Electronic Energy = −5659.556648 Hartree

Number of imaginary frequencies = 0

Sum of electronic and zero-point Energies = −5659.398835 Hartree

Sum of electronic and thermal Energies = −5659.387428 Hartree

Sum of electronic and thermal Enthalpies = −5659.386484 Hartree

Sum of electronic and thermal Free Energies = −5659.439371 Hartree

**Atoms**	**Cartesian Coordinates**			**Atoms**	**Cartesian Coordinates**		
	X	***Y***	***Z***		***X***	***Y***	***Z***
C	−1.129678	1.911322	−0.000004	C	−0.732359	0.541729	0.000008
C	0.732359	0.541730	0.000009	C	1.129678	1.911322	−0.000002
N	−0.000002	2.689833	−0.000012	C	−2.473886	2.309547	−0.000007
C	−3.438225	1.307117	−0.000001	C	−3.049634	−0.042222	0.000025
C	−1.695501	−0.448478	0.000022	C	1.695502	−0.448477	0.000021
C	3.049634	−0.042220	0.000027	C	3.438224	1.307117	0.000005
C	2.473884	2.309548	−0.000001	Br	−4.385185	−1.376135	−0.000006
Br	4.385186	−1.376135	−0.000008	H	−0.000002	3.705809	−0.000017
H	−2.754659	3.356910	−0.000021	H	−4.489597	1.567330	−0.000014
H	−1.438197	−1.500937	0.000027	H	1.438197	−1.500936	0.000021
H	4.489596	1.567332	−0.000006	H	2.754657	3.356911	−0.000011

3a-O• (neutral radical) b3lyp/6-311+g(d,p) scrf=(cpcm,solvent=acetonitrile)

```
# opt freq b3lyp/6-311+g(d,p)
scrf=(cpcm,solvent=acetonitrile)
```

Charge = 0, Multiplicity = 2, Point group = C1

Electronic Energy = -1016.331788 Hartree

Number of imaginary frequencies = 0

Sum of electronic and zero-point Energies = -1016.010425 Hartree

Sum of electronic and thermal Energies = -1015.991188 Hartree

Sum of electronic and thermal Enthalpies = -1015.990244 Hartree

Sum of electronic and thermal Free Energies = -1016.060627 Hartree

	**Cartesian Coordinates**				**Cartesian Coordinates**		
	X	***Y***	***Z***		***X***	***Y***	***Z***
C	-0.348720	1.384012	0.032451	C	1.086573	1.345805	0.024860
C	1.454388	-0.063709	0.004162	C	0.254195	-0.785450	-0.001480
N	-0.830160	0.104504	0.017231	C	-1.078445	2.593846	0.069777
C	-0.381864	3.770370	0.087235	C	1.073813	3.802862	0.071006
C	1.781221	2.539940	0.042792	C	2.695968	-0.767928	-0.019024
C	2.643846	-2.203447	-0.052161	C	1.386668	-2.876082	-0.064396
C	0.197123	-2.191257	-0.040532	C	3.969470	-0.143144	-0.011670
C	5.127987	-0.889319	-0.035295	C	5.075874	-2.299869	-0.067668
C	3.856876	-2.938541	-0.075792	C	-2.213911	-0.272254	0.005606
C	-2.983285	-0.056696	-1.136915	C	-4.326666	-0.424016	-1.141011
C	-4.921065	-1.014545	-0.019325	C	-4.126162	-1.229250	1.113700
C	-2.784120	-0.859309	1.134734	C	-6.383031	-1.386680	-0.021386
H	-2.161296	2.580119	0.084765	H	-0.897114	4.723098	0.116347
H	2.861231	2.592611	0.038761	H	1.382390	-3.959915	-0.094721
H	-0.752149	-2.711041	-0.052688	H	4.040223	0.934895	0.014080
H	6.089038	-0.387654	-0.028558	H	5.994651	-2.874419	-0.086084
H	3.806202	-4.021978	-0.100832	H	-2.531317	0.386754	-2.016270
H	-4.917899	-0.255868	-2.034646	H	-4.561787	-1.688238	1.994629
H	-2.182336	-1.022257	2.021016	H	-6.736164	-1.604269	-1.031311
H	-6.571799	-2.259684	0.606879	H	-6.991593	-0.564226	0.369600
O	1.700054	4.898564	0.085051				

3a-O⁻ (anion) b3lyp/6-311+g(d,p) scrf=(cpcm,solvent=acetonitrile)

```
# opt freq b3lyp/6-311+g(d,p)
scrf=(cpcm,solvent=acetonitrile)
```

Charge = -1, Multiplicity = 1, Point group = C1

Electronic Energy = -1016.480272 Hartree

Number of imaginary frequencies = 0

Sum of electronic and zero-point Energies = -1016.160068 Hartree

Sum of electronic and thermal Energies = -1016.140819 Hartree

Sum of electronic and thermal Enthalpies = -1016.139874 Hartree

Sum of electronic and thermal Free Energies = -1016.209519 Hartree

	Cartesian Coordinates				Cartesian Coordinates		
	X	***Y***	***Z***		***X***	***Y***	***Z***
C	-0.332517	1.385867	0.027971	C	1.085042	1.332334	0.027458
C	1.434069	-0.078847	0.003517	C	0.220742	-0.801737	-0.006359
N	-0.847104	0.077551	0.010384	C	-1.010756	2.606188	0.079398
C	-0.268722	3.777632	0.112931	C	1.174161	3.802015	0.101262
C	1.819116	2.531719	0.062838	C	2.671594	-0.795122	-0.025262
C	2.622876	-2.231453	-0.071939	C	1.361914	-2.902056	-0.095696
C	0.176539	-2.212482	-0.065580	C	3.949612	-0.176269	-0.012145
C	5.110480	-0.922612	-0.040831	C	5.058484	-2.331495	-0.084904
C	3.834457	-2.965891	-0.100103	C	-2.226102	-0.272801	0.002727
C	-3.055979	0.152585	-1.038845	C	-4.406219	-0.186262	-1.039944
C	-4.962662	-0.964266	-0.016427	C	-4.117494	-1.390211	1.014249
C	-2.768000	-1.042627	1.034747	C	-6.432378	-1.308275	-0.013976
H	-2.094694	2.642017	0.092789	H	-0.782344	4.734072	0.150441
H	2.902184	2.532553	0.065811	H	1.354417	-3.985909	-0.140894
H	-0.771095	-2.736140	-0.089011	H	4.018449	0.901617	0.022254
H	6.071484	-0.419831	-0.029036	H	5.975746	-2.908963	-0.107212
H	3.781888	-4.049662	-0.135107	H	-2.639404	0.739430	-1.848939
H	-5.034732	0.149648	-1.858376	H	-4.519551	-1.994925	1.820529
H	-2.134210	-1.365943	1.852196	H	-6.806655	-1.460745	-1.029017
H	-6.627790	-2.213907	0.563909	H	-7.021927	-0.499891	0.432077
O	1.828554	4.919767	0.129297				

11 (ground state) b3lyp/6-311+g(d,p) scrf=(cpcm,solvent=acetonitrile)

```
# opt freq b3lyp/6-311+g(d,p)
scrf=(cpcm,solvent=acetonitrile)
```

Charge = 0, Multiplicity = 1, Point group = C1

Electronic Energy = -517.608787 Hartree

Number of imaginary frequencies = 0

Sum of electronic and zero-point Energies = -517.432980 Hartree

Sum of electronic and thermal Energies = -517.424061 Hartree

Sum of electronic and thermal Enthalpies = -517.423117 Hartree

Sum of electronic and thermal Free Energies = -517.466926 Hartree

	Cartesian Coordinates				Cartesian Coordinates		
	X	***Y***	***Z***		***X***	***Y***	***Z***

C	1.130884	0.856129	-0.000000	C	0.724834	-0.505229	-0.000000
C	-0.724834	-0.505229	-0.000000	C	-1.130884	0.856129	-0.000000
N	0.000000	1.654690	-0.000000	C	2.479239	1.221047	-0.000000
C	3.424861	0.200094	0.000000	C	3.041950	-1.153379	0.000000
C	1.697766	-1.511960	-0.000000	C	-1.697766	-1.511960	-0.000000
C	-3.041950	-1.153379	0.000000	C	-3.424861	0.200094	0.000000
C	-2.479239	1.221047	0.000000	H	0.000000	2.662849	0.000000
H	2.779401	2.262619	0.000000	H	4.478784	0.454895	0.000000
H	3.805032	-1.923190	0.000000	H	1.409013	-2.557377	0.000000
H	-1.409013	-2.557377	-0.000000	H	-3.805032	-1.923190	0.000000
H	-4.478784	0.454895	0.000000	H	-2.779401	2.262619	0.000000

13a (ground state) b3lyp/6-311+g(d,p) scrf=(cpcm,solvent=acetonitrile)

```
# opt freq b3lyp/6-311+g(d,p)
scrf=(cpcm,solvent=acetonitrile)
```

Charge = 0, Multiplicity = 1, Point group = C1

Electronic Energy = -671.287644 Hartree

Number of imaginary frequencies = 0

Sum of electronic and zero-point Energies = -671.064889 Hartree

Sum of electronic and thermal Energies = -671.053318 Hartree

Sum of electronic and thermal Enthalpies = -671.052374 Hartree

Sum of electronic and thermal Free Energies = -671.102373 Hartree

	Cartesian Coordinates				Cartesian Coordinates		
	X	***Y***	***Z***		***X***	***Y***	***Z***
C	-2.299721	0.709815	-0.000001	C	-1.378957	-0.378124	-0.000001
C	-0.050904	0.207419	-0.000001	C	-0.233456	1.603415	-0.000001
N	-1.582212	1.888404	-0.000002	C	-3.683510	0.523517	-0.000001
C	-4.160243	-0.781754	0.000001	C	-3.275402	-1.874877	0.000003
C	-1.898294	-1.684669	0.000002	C	1.278655	-0.321845	-0.000001
C	2.370362	0.611664	0.000002	C	2.114570	2.018082	0.000002
C	0.839791	2.520938	0.000000	C	1.585244	-1.706350	-0.000003
C	2.891522	-2.150412	-0.000003	C	3.962215	-1.232331	0.000001
C	3.699809	0.120243	0.000003	H	-1.981350	2.814199	-0.000002
H	-4.360390	1.370182	-0.000002	H	-5.229740	-0.959077	0.000002
H	-3.674564	-2.882706	0.000005	H	-1.247187	-2.548709	0.000004
H	2.962122	2.694874	0.000004	H	0.654578	3.588797	-0.000000
H	0.784372	-2.432525	-0.000007	H	3.096172	-3.215344	-0.000005
H	4.984844	-1.591876	0.000002	H	4.516532	0.834987	0.000005

13b (ground state) b3lyp/6-311+g(d,p) scrf=(cpcm,solvent=acetonitrile)

```
# opt freq b3lyp/6-311+g(d,p)
scrf=(cpcm,solvent=acetonitrile)
```

Charge = 0, Multiplicity = 1, Point group = C1

Electronic Energy = -671.288502 Hartree

Number of imaginary frequencies = 0

Sum of electronic and zero-point Energies = -671.065895 Hartree

Sum of electronic and thermal Energies = -671.054304 Hartree

Sum of electronic and thermal Enthalpies = -671.053360 Hartree

Sum of electronic and thermal Free Energies = -671.103349 Hartree

	Cartesian Coordinates				Cartesian Coordinates		
	X	***Y***	***Z***		***X***	***Y***	***Z***
C	1.992710	-0.887484	-0.000000	C	1.914071	0.531207	-0.000000
C	0.507697	0.864777	-0.000000	C	-0.200334	-0.349244	-0.000000
N	0.704576	-1.393712	-0.000000	C	3.216828	-1.560081	0.000000
C	4.374774	-0.789368	0.000000	C	4.318226	0.617360	0.000000
C	3.097600	1.282588	-0.000000	C	-0.198579	2.092677	-0.000000
C	-1.570616	2.081591	0.000000	C	-2.308800	0.856072	0.000000
C	-1.622008	-0.402837	-0.000000	C	-3.726766	0.847076	0.000000
C	-4.434275	-0.335517	0.000000	C	-3.751303	-1.571575	-0.000000
C	-2.373340	-1.603885	-0.000000	H	0.474080	-2.374762	0.000000
H	3.263591	-2.643016	0.000000	H	5.340068	-1.283022	0.000000
H	5.240909	1.186342	0.000000	H	3.063307	2.366541	-0.000000
H	0.342649	3.032318	-0.000000	H	-2.123916	3.014322	0.000000
H	-4.252223	1.796490	0.000000	H	-5.518286	-0.320312	0.000000
H	-4.313992	-2.498111	-0.000000	H	-1.863531	-2.560946	-0.000000

13c (ground state) b3lyp/6-311+g(d,p) scrf=(cpcm,solvent=acetonitrile)

```
# opt freq b3lyp/6-311+g(d,p)
scrf=(cpcm,solvent=acetonitrile)
```

Charge = 0, Multiplicity = 1, Point group = C1

Electronic Energy = -671.285640 Hartree

Number of imaginary frequencies = 0

Sum of electronic and zero-point Energies = -671.063476 Hartree

Sum of electronic and thermal Energies = -671.051834 Hartree

Sum of electronic and thermal Enthalpies = -671.050890 Hartree

Sum of electronic and thermal Free Energies = -671.100983 Hartree

	Cartesian Coordinates				Cartesian Coordinates		
	X	***Y***	***Z***		***X***	***Y***	***Z***
C	2.418963	-0.812389	-0.000000	C	1.860395	0.491735	-0.000000

C	0.416067	0.326850	-0.000000	C	0.167709	-1.089478	-0.000000
N	1.390062	-1.741461	-0.000000	C	3.799025	-1.018203	0.000000
C	4.620704	0.106976	0.000000	C	4.085294	1.405535	0.000000
C	2.706690	1.604094	-0.000000	C	-0.646212	1.207417	-0.000000
C	-1.972809	0.712964	-0.000000	C	-2.204624	-0.710367	-0.000000
C	-1.108797	-1.607630	-0.000000	C	-3.095833	1.588907	0.000000
C	-4.379594	1.100886	0.000000	C	-4.607784	-0.299445	0.000000
C	-3.550748	-1.176802	0.000000	H	1.513110	-2.741715	0.000000
H	4.217979	-2.017714	0.000000	H	5.696912	-0.025004	0.000001
H	4.753066	2.259109	0.000000	H	2.297479	2.608393	-0.000000
H	-0.480998	2.279834	-0.000000	H	-1.285212	-2.677523	-0.000000
H	-2.917088	2.659446	-0.000000	H	-5.222584	1.782587	0.000000
H	-5.624648	-0.676160	0.000001	H	-3.729129	-2.247312	0.000000

13d (ground state) b3lyp/6-311+g(d,p) scrf=(cpcm,solvent=acetonitrile)

```
# opt freq b3lyp/6-311+g(d,p)
scrf=(cpcm,solvent=acetonitrile)
```

Charge = 0, Multiplicity = 1, Point group = C1

Electronic Energy = -788.037076 Hartree

Number of imaginary frequencies = 0

Sum of electronic and zero-point Energies = -787.753977 Hartree

Sum of electronic and thermal Energies = -787.738469 Hartree

Sum of electronic and thermal Enthalpies = -787.737524 Hartree

Sum of electronic and thermal Free Energies = -787.798499 Hartree

	Cartesian Coordinates				Cartesian Coordinates		
	X	***Y***	***Z***		***X***	***Y***	***Z***
C	-0.948499	1.130291	-0.042402	C	-2.306556	0.719923	-0.027008
C	-2.303695	-0.726641	0.028933	C	-0.944030	-1.131680	0.042856
N	-0.131110	0.000905	-0.000053	C	-0.592504	2.479348	-0.113274
C	-1.619135	3.418370	-0.159730	C	-2.969971	3.029743	-0.138660
C	-3.319380	1.684622	-0.074785	C	-3.312663	-1.695330	0.077495
C	-2.957901	-3.039087	0.140588	C	-1.605527	-3.422417	0.160027
C	-0.582643	-2.479349	0.112776	C	1.296817	0.003253	-0.001051
C	1.999323	-0.540296	-1.077721	C	3.392363	-0.539624	-1.070913
C	4.113325	0.010964	-0.004602	C	3.391828	0.562156	1.061554
C	1.999228	0.553476	1.072746	C	5.622281	-0.011139	0.008291
H	0.445404	2.787703	-0.131951	H	-1.368780	4.471885	-0.214217
H	-3.744352	3.787173	-0.175337	H	-4.362533	1.388289	-0.064563
H	-4.356984	-1.403106	0.068385	H	-3.729266	-3.799562	0.177824
H	-1.350984	-4.474965	0.213799	H	0.456505	-2.783579	0.130049

H	1.456310	-0.954854	-1.919030	H	3.925594	-0.964083	-1.915036
H	3.924825	0.998508	1.899806	H	1.455971	0.971762	1.912057
H	6.028284	-0.026686	-1.005274	H	6.028304	0.858924	0.528887
H	5.993510	-0.903252	0.524231				

13e (ground state) b3lyp/6-311+g(d,p) scrf=(cpcm,solvent=acetonitrile)

```
# opt freq b3lyp/6-311+g(d,p)
scrf=(cpcm,solvent=acetonitrile)
```

Charge = 0, Multiplicity = 1, Point group = C1

Electronic Energy = -941.715522 Hartree

Number of imaginary frequencies = 0

Sum of electronic and zero-point Energies = -941.385821 Hartree

Sum of electronic and thermal Energies = -941.367537 Hartree

Sum of electronic and thermal Enthalpies = -941.366592 Hartree

Sum of electronic and thermal Free Energies = -941.434083 Hartree

	**Cartesian Coordinates**				**Cartesian Coordinates**		
	X	***Y***	***Z***		***X***	***Y***	***Z***
C	-0.355905	1.583262	0.035134	C	1.067292	1.615033	0.037693
C	1.510858	0.235502	0.004991	C	0.347890	-0.555869	-0.016083
N	-0.776390	0.259202	0.002464	C	-1.130644	2.744368	0.079156
C	-0.467488	3.965421	0.117138	C	0.936670	4.026471	0.113065
C	1.703793	2.867669	0.075520	C	2.792020	-0.401562	-0.016492
C	2.825433	-1.836184	-0.064814	C	1.606953	-2.581140	-0.093360
C	0.381900	-1.966638	-0.070503	C	4.028347	0.293343	0.006835
C	5.230352	-0.383404	-0.015494	C	5.260849	-1.792961	-0.062761
C	4.078231	-2.499133	-0.086812	C	-2.135736	-0.184941	-0.001694
C	-2.937164	0.024685	-1.124753	C	-4.261800	-0.405333	-1.122167
C	-4.810804	-1.058687	-0.011788	C	-3.988563	-1.268352	1.101773
C	-2.665888	-0.831261	1.115373	C	-6.253666	-1.500714	-0.006723
H	-2.212500	2.694635	0.083164	H	-1.043574	4.883109	0.150884
H	1.429028	4.991709	0.141743	H	2.782305	2.950039	0.077828
H	1.666120	-3.663329	-0.135513	H	-0.534646	-2.542593	-0.094653
H	4.036193	1.373516	0.044068	H	6.160076	0.174327	0.003789
H	6.210868	-2.314569	-0.080127	H	4.089872	-3.583659	-0.123421
H	-2.521372	0.515990	-1.996698	H	-4.874138	-0.238811	-2.002153
H	-4.387468	-1.775409	1.973939	H	-2.044586	-0.990638	1.989020
H	-6.591282	-1.766831	-1.010680	H	-6.403376	-2.362195	0.647324
H	-6.904373	-0.697168	0.354830				

13f (ground state) b3lyp/6-311+g(d,p) scrf=(cpcm,solvent=acetonitrile)

```
# opt freq b3lyp/6-311+g(d,p)
scrf=(cpcm,solvent=acetonitrile)
```

Charge = 0, Multiplicity = 1, Point group = C1

Electronic Energy = -941.712421 Hartree

Number of imaginary frequencies = 0

Sum of electronic and zero-point Energies = -941.382659 Hartree

Sum of electronic and thermal Energies = -941.364440 Hartree

Sum of electronic and thermal Enthalpies = -941.363495 Hartree

Sum of electronic and thermal Free Energies = -941.430550 Hartree

	Cartesian Coordinates				Cartesian Coordinates		
	X	***Y***	***Z***		***X***	***Y***	***Z***
C	0.296480	2.002325	-0.000042	C	1.710170	2.077602	-0.000035
C	2.190940	0.719168	-0.000029	C	1.066803	-0.129288	-0.000033
N	-0.085416	0.662953	-0.000056	C	-0.503274	3.148428	-0.000038
C	0.139573	4.382140	0.000010	C	1.543986	4.476786	0.000045
C	2.333891	3.333097	0.000022	C	3.500868	0.185596	-0.000002
C	3.672669	-1.173018	0.000029	C	2.553751	-2.061379	0.000027
C	1.207795	-1.553694	0.000003	C	2.754452	-3.465350	0.000030
C	1.693818	-4.343077	0.000004	C	0.374255	-3.845193	-0.000007
C	0.137368	-2.487065	-0.000003	C	-1.460835	0.255614	-0.000027
C	-2.135922	0.075013	-1.206815	C	-3.476728	-0.302555	-1.201231
C	-4.169926	-0.497017	0.000030	C	-3.476702	-0.302488	1.201253
C	-2.135888	0.075083	1.206782	C	-5.630054	-0.877530	0.000044
H	-1.584207	3.080958	-0.000074	H	-0.455089	5.288670	0.000014
H	2.011803	5.454585	0.000102	H	3.415397	3.412900	0.000029
H	4.357231	0.850617	-0.000009	H	4.669094	-1.600760	0.000059
H	3.772799	-3.839881	0.000057	H	1.869723	-5.412783	-0.000007
H	-0.462385	-4.534670	-0.000017	H	-0.883066	-2.134326	0.000010
H	-1.608556	0.219511	-2.142597	H	-3.990393	-0.449381	-2.145433
H	-3.990338	-0.449253	2.145481	H	-1.608503	0.219627	2.142546
H	-5.888732	-1.461282	-0.885763	H	-5.888834	-1.460838	0.886113
H	-6.262975	0.016449	-0.000221				

13g (ground state) b3lyp/6-311+g(d,p) scrf=(cpcm,solvent=acetonitrile)

```
# opt freq b3lyp/6-311+g(d,p)
scrf=(cpcm,solvent=acetonitrile)
```

Charge = 0, Multiplicity = 1, Point group = C1

Electronic Energy = -941.714041 Hartree

Number of imaginary frequencies = 0

Sum of electronic and zero-point Energies = -941.384661 Hartree

Sum of electronic and thermal Energies = -941.366406 Hartree

Sum of electronic and thermal Enthalpies = -941.365462 Hartree

Sum of electronic and thermal Free Energies = -941.432817 Hartree

	**Cartesian Coordinates**				**Cartesian Coordinates**		
	X	***Y***	***Z***		***X***	***Y***	***Z***
C	-0.771823	1.860872	0.037454	C	0.559099	2.346757	0.037237
C	1.429541	1.185206	0.018172	C	0.573476	0.032611	0.008484
N	-0.753815	0.463975	0.021128	C	-1.864730	2.728076	0.071049
C	-1.605274	4.097246	0.096134	C	-0.292795	4.595230	0.090042
C	0.793552	3.723979	0.062659	C	2.799377	1.023864	0.000476
C	3.356697	-0.277253	-0.029395	C	2.484693	-1.424240	-0.045755
C	1.078834	-1.248811	-0.027560	C	4.765098	-0.487056	-0.047387
C	5.295007	-1.753899	-0.080207	C	4.436740	-2.883429	-0.097315
C	3.072522	-2.721307	-0.080668	C	-1.903007	-0.383039	0.009860
C	-2.760638	-0.388676	-1.091463	C	-3.882924	-1.213840	-1.094931
C	-4.167859	-2.056893	-0.014117	C	-3.290120	-2.046703	1.076991
C	-2.173322	-1.214825	1.098039	C	-5.397709	-2.931539	-0.013246
H	-2.880769	2.353839	0.077532	H	-2.438206	4.790856	0.121696
H	-0.125926	5.665806	0.109116	H	1.806832	4.110195	0.063140
H	3.461030	1.883734	0.006204	H	0.428066	-2.114871	-0.040974
H	5.418143	0.379648	-0.034943	H	6.369702	-1.895580	-0.093518
H	4.863686	-3.879813	-0.123684	H	2.419072	-3.587562	-0.093758
H	-2.545147	0.245955	-1.943176	H	-4.540406	-1.209076	-1.957913
H	-3.484941	-2.692514	1.926775	H	-1.510879	-1.209162	1.955760
H	-5.698520	-3.195416	-1.029285	H	-5.229507	-3.853768	0.547050
H	-6.242142	-2.414059	0.454776				

13h (ground state) b3lyp/6-311+g(d,p) scrf=(cpcm,solvent=acetonitrile)

```
# opt freq b3lyp/6-311+g(d,p)
scrf=(cpcm,solvent=acetonitrile)
```

Charge = 0, Multiplicity = 1, Point group = C1

Electronic Energy = -592.857281 Hartree

Number of imaginary frequencies = 0

Sum of electronic and zero-point Energies = -592.677685 Hartree

Sum of electronic and thermal Energies = -592.667382 Hartree

Sum of electronic and thermal Enthalpies = -592.666438 Hartree

Sum of electronic and thermal Free Energies = -592.713202 Hartree

	**Cartesian Coordinates**				**Cartesian Coordinates**		
	X	***Y***	***Z***		***X***	***Y***	***Z***

C	0.627413	1.150514	-0.000001	C	0.432886	-0.253341	-0.000002
C	-0.998753	-0.472873	-0.000002	C	-1.607074	0.812614	-0.000001
N	-0.613735	1.772532	0.000000	C	1.907956	1.705423	0.000002
C	2.996941	0.842025	0.000010	C	2.814905	-0.552621	-0.000001
C	1.542247	-1.110874	-0.000016	C	-1.808055	-1.616123	-0.000000
C	-3.190567	-1.466364	0.000003	C	-3.774902	-0.185952	0.000002
C	-2.996253	0.966666	0.000001	O	3.959475	-1.320757	-0.000029
H	-0.766234	2.768551	0.000001	H	2.057434	2.778732	0.000006
H	4.007042	1.234287	0.000025	H	1.414690	-2.188663	-0.000042
H	-1.364074	-2.605709	0.000001	H	-3.827851	-2.343121	0.000005
H	-4.855372	-0.094653	0.000004	H	-3.451067	1.950551	0.000001
H	3.725327	-2.256210	0.000266				

13i (ground state) b3lyp/6-311+g(d,p) scrf=(cpcm,solvent=acetonitrile)

```
# opt freq b3lyp/6-311+g(d,p)
scrf=(cpcm,solvent=acetonitrile)
```

Charge = 0, Multiplicity = 1, Point group = C1

Electronic Energy = -746.536249 Hartree

Number of imaginary frequencies = 0

Sum of electronic and zero-point Energies = -746.309987 Hartree

Sum of electronic and thermal Energies = -746.297006 Hartree

Sum of electronic and thermal Enthalpies = -746.296061 Hartree

Sum of electronic and thermal Free Energies = -746.349033 Hartree

	Cartesian Coordinates				Cartesian Coordinates		
	X	*Y*	*Z*		*X*	*Y*	*Z*
C	-1.871249	-1.250567	0.000011	C	-1.144587	-0.028040	0.000006
C	0.260405	-0.384917	0.000003	C	0.311594	-1.793831	0.000004
N	-0.968027	-2.298517	0.000011	C	-3.265987	-1.283926	0.000011
C	-3.951965	-0.077722	0.000007	C	-3.252649	1.142521	-0.000008
C	-1.864091	1.181978	-0.000015	C	1.484240	0.357975	0.000004
C	2.715560	-0.381643	-0.000010	C	2.696500	-1.811634	-0.000016
C	1.523584	-2.519302	-0.000006	C	1.558397	1.773821	0.000020
C	2.773610	2.428039	0.000018	C	3.981031	1.700029	-0.000001
C	3.945185	0.322367	-0.000014	O	-4.021372	2.286046	-0.000041
H	-1.207534	-3.277715	0.000009	H	-3.804226	-2.224661	0.000019
H	-5.035213	-0.059120	0.000017	H	-1.362261	2.141244	-0.000041
H	3.644777	-2.338195	-0.000026	H	1.518797	-3.603162	-0.000007
H	0.649773	2.359514	0.000039	H	2.798967	3.512165	0.000033
H	4.930307	2.223385	-0.000004	H	4.868673	-0.247773	-0.000025
H	-3.452350	3.064693	0.000147				

13j (ground state) b3lyp/6-311+g(d,p) scrf=(cpcm,solvent=acetonitrile)

```
# opt freq b3lyp/6-311+g(d,p)
scrf=(cpcm,solvent=acetonitrile)
```

Charge = 0, Multiplicity = 1, Point group = C1

Electronic Energy = -746.536975 Hartree

Number of imaginary frequencies = 0

Sum of electronic and zero-point Energies = -746.310860 Hartree

Sum of electronic and thermal Energies = -746.297793 Hartree

Sum of electronic and thermal Enthalpies = -746.296849 Hartree

Sum of electronic and thermal Free Energies = -746.349985 Hartree

	Cartesian Coordinates				Cartesian Coordinates		
	X	*Y*	*Z*		*X*	*Y*	*Z*
C	1.564897	-1.073781	0.000003	C	1.572569	0.345366	0.000005
C	0.189533	0.762448	0.000003	C	-0.591107	-0.407995	0.000004
N	0.245412	-1.504188	0.000004	C	2.749683	-1.808107	0.000001
C	3.952135	-1.108497	-0.000021	C	3.971107	0.299318	-0.000002
C	2.792846	1.034653	0.000021	C	-0.441558	2.030782	0.000001
C	-1.811570	2.103255	-0.000000	C	-2.622857	0.924182	0.000000
C	-2.013947	-0.373846	0.000002	C	-4.038628	1.000682	-0.000002
C	-4.816357	-0.137268	-0.000003	C	-4.209573	-1.412272	-0.000002
C	-2.835840	-1.527375	0.000000	O	5.156891	1.002197	-0.000072
H	-0.044892	-2.468831	0.000007	H	2.744348	-2.891847	0.000001
H	4.889533	-1.654973	-0.000052	H	2.834896	2.117620	0.000050
H	0.155648	2.935960	-0.000001	H	-2.307461	3.067698	-0.000002
H	-4.505871	1.980086	-0.000003	H	-5.897486	-0.056545	-0.000005
H	-4.827199	-2.303120	-0.000004	H	-2.383954	-2.513301	-0.000000
H	5.901422	0.389720	0.000499				

13k (ground state) b3lyp/6-311+g(d,p) scrf=(cpcm,solvent=acetonitrile)

```
# opt freq b3lyp/6-311+g(d,p)
scrf=(cpcm,solvent=acetonitrile)
```

Charge = 0, Multiplicity = 1, Point group = C1

Electronic Energy = -746.533907 Hartree

Number of imaginary frequencies = 0

Sum of electronic and zero-point Energies = -746.308143 Hartree

Sum of electronic and thermal Energies = -746.295091 Hartree

Sum of electronic and thermal Enthalpies = -746.294146 Hartree

Sum of electronic and thermal Free Energies = -746.347211 Hartree

	Cartesian Coordinates				Cartesian Coordinates		

	X	Y	Z		X	Y	Z
C	-1.990727	1.182088	-0.000000	C	-1.560288	-0.165793	-0.000002
C	-0.108310	-0.140336	-0.000002	C	0.275832	1.246494	-0.000001
N	-0.875201	2.013113	-0.000002	C	-3.346961	1.506012	0.000002
C	-4.271092	0.465733	-0.000001	C	-3.852661	-0.875019	0.000001
C	-2.500424	-1.202374	0.000004	C	0.862623	-1.121177	-0.000001
C	2.230605	-0.759682	-0.000000	C	2.600384	0.634662	-0.000001
C	1.598741	1.635117	-0.000001	C	3.262316	-1.741867	0.000001
C	4.587556	-1.382424	0.000002	C	4.951826	-0.010792	0.000001
C	3.986368	0.966089	0.000000	O	-4.844555	-1.831365	0.000014
H	-0.901902	3.020450	0.000002	H	-3.681279	2.536714	0.000001
H	-5.333524	0.678983	-0.000005	H	-2.185609	-2.240913	0.000009
H	0.592490	-2.172106	-0.000001	H	1.880092	2.682527	-0.000002
H	2.978966	-2.789648	0.000001	H	5.359740	-2.143403	0.000003
H	6.000817	0.264180	0.000002	H	4.269369	2.013795	-0.000000
H	-4.451046	-2.711846	-0.000119				

13l (ground state) b3lyp/6-311+g(d,p) scrf=(cpcm,solvent=acetonitrile)

```
# opt freq b3lyp/6-311+g(d,p)
scrf=(cpcm,solvent=acetonitrile)
```

Charge = 0, Multiplicity = 1, Point group = C1

Electronic Energy = -863.285704 Hartree

Number of imaginary frequencies = 0

Sum of electronic and zero-point Energies = -862.998792 Hartree

Sum of electronic and thermal Energies = -862.981947 Hartree

Sum of electronic and thermal Enthalpies = -862.981003 Hartree

Sum of electronic and thermal Free Energies = -863.044834 Hartree

	Cartesian Coordinates				Cartesian Coordinates		
	X	Y	Z		X	Y	Z
C	0.891856	-0.742138	-0.024327	C	2.136217	-0.068383	-0.004139
C	1.842372	1.347121	0.039567	C	0.427584	1.470983	0.042917
N	-0.141324	0.201181	0.003761	C	0.826424	-2.135058	-0.090020
C	2.018956	-2.848392	-0.123131	C	3.259581	-2.187669	-0.094211
C	3.330584	-0.800737	-0.038178	C	2.637266	2.498674	0.086341
C	2.021433	3.744415	0.139013	C	0.618913	3.849286	0.150202
C	-0.194681	2.721359	0.103720	C	-1.537761	-0.090809	-0.000868
C	-2.339374	0.322859	-1.066830	C	-3.703123	0.040289	-1.062943
C	-4.295292	-0.669655	-0.011270	C	-3.474347	-1.087834	1.042875
C	-2.112190	-0.796854	1.057838	C	-5.777607	-0.952912	-0.002712
O	4.381779	-2.985897	-0.127632	H	-0.122446	-2.655933	-0.115055

H	2.007019	-3.930750	-0.172415	H	4.292390	-0.298273	-0.023516
H	3.719000	2.421012	0.084220	H	2.624907	4.643950	0.174924
H	0.158913	4.830008	0.196859	H	-1.273617	2.812780	0.114707
H	-1.893871	0.856985	-1.897929	H	-4.313310	0.367014	-1.898487
H	-3.905703	-1.642493	1.869567	H	-1.494265	-1.114960	1.889413
H	-6.166708	-1.064542	-1.017105	H	-6.005279	-1.863186	0.555851
H	-6.327749	-0.131975	0.469867	H	5.174894	-2.438004	-0.099241

13m (ground state) b3lyp/6-311+g(d,p) scrf=(cpcm,solvent=acetonitrile)

```
# opt freq b3lyp/6-311+g(d,p)
scrf=(cpcm,solvent=acetonitrile)
```

Charge = 0, Multiplicity = 1, Point group = C1

Electronic Energy = -1170.637864 Hartree

Number of imaginary frequencies = 0

Sum of electronic and zero-point Energies = -1170.257545 Hartree

Sum of electronic and thermal Energies = -1170.235072 Hartree

Sum of electronic and thermal Enthalpies = -1170.234128 Hartree

Sum of electronic and thermal Free Energies = -1170.311795 Hartree

	**Cartesian Coordinates**				**Cartesian Coordinates**		
	X	***Y***	***Z***		***X***	***Y***	***Z***
C	-0.465042	1.803168	0.003114	C	0.949748	1.860111	0.002229
C	1.407214	0.487797	0.001649	C	0.268508	-0.330646	0.002486
N	-0.868858	0.471733	0.003149	C	-1.260124	2.950745	0.002595
C	-0.630524	4.185959	0.001784	C	0.773069	4.267840	0.001024
C	1.566350	3.128164	0.001053	C	2.726500	-0.092034	-0.000275
C	2.852632	-1.513127	-0.002433	C	1.655001	-2.355531	0.000344
C	0.350171	-1.771137	0.003910	C	1.747412	-3.765782	0.000963
C	0.628255	-4.575824	0.005616	C	-0.648433	-3.997996	0.010307
C	-0.780038	-2.623436	0.009422	C	-2.257914	0.110546	-0.000192
C	-2.936091	-0.043878	-1.208387	C	-4.291742	-0.365359	-1.205752
C	-4.996171	-0.526418	-0.006434	C	-4.299250	-0.355404	1.196643
C	-2.944255	-0.034136	1.205342	C	-6.470645	-0.846663	-0.007698
O	1.310293	5.535855	0.000031	C	3.903548	0.692506	-0.000621
C	5.159512	0.116985	-0.003972	C	5.286390	-1.279155	-0.007272
C	4.151920	-2.069048	-0.006396	H	-2.340714	2.883673	0.003047
H	-1.209522	5.101535	0.001543	H	2.641555	3.245281	-0.000360
H	2.718088	-4.241370	-0.001859	H	0.742051	-5.653756	0.005921
H	-1.533370	-4.623873	0.014870	H	-1.771177	-2.199561	0.013328
H	-2.400455	0.075585	-2.142955	H	-4.808011	-0.493239	-2.151214
H	-4.821689	-0.475827	2.139785	H	-2.414412	0.092999	2.142206

H	-6.766070	-1.357302	-0.926301	H	-6.740456	-1.478763	0.841121
H	-7.065867	0.069871	0.065674	H	2.273256	5.484197	0.000094
H	3.829921	1.768718	0.001930	H	6.042064	0.746662	-0.004160
H	6.266998	-1.740820	-0.010319	H	4.281461	-3.142471	-0.008935

13n (ground state) b3lyp/6-311+g(d,p) scrf=(cpcm,solvent=acetonitrile)

```
# opt freq b3lyp/6-311+g(d,p)
scrf=(cpcm,solvent=acetonitrile)
```

Charge = 0, Multiplicity = 1, Point group = C1

Electronic Energy = -1092.206566 Hartree

Number of imaginary frequencies = 0

Sum of electronic and zero-point Energies = -1091.869279 Hartree

Sum of electronic and thermal Energies = -1091.848343 Hartree

Sum of electronic and thermal Enthalpies = -1091.847399 Hartree

Sum of electronic and thermal Free Energies = -1091.920377 Hartree

	**Cartesian Coordinates**				**Cartesian Coordinates**		
	X	***Y***	***Z***		***X***	***Y***	***Z***
C	-0.809287	1.642740	-0.029081	C	0.504206	2.155995	0.015537
C	1.405175	1.019386	0.015692	C	0.579390	-0.152849	-0.030034
N	-0.763649	0.243498	-0.061626	C	-1.916465	2.491439	-0.020816
C	-1.691234	3.863477	0.020643	C	-0.386760	4.383096	0.055907
C	0.718478	3.538354	0.055957	C	2.782172	0.919350	0.034426
C	3.382048	-0.358456	0.008294	C	2.559595	-1.539312	-0.045967
C	1.144020	-1.418599	-0.072460	C	4.797558	-0.520038	0.027976
C	5.375733	-1.764824	-0.003385	C	4.561663	-2.925031	-0.056893
C	3.192084	-2.814990	-0.078136	C	-1.918518	-0.599237	-0.022828
C	-2.731826	-0.724903	-1.153295	C	-3.868056	-1.526444	-1.106186
C	-4.215877	-2.227969	0.056348	C	-3.394393	-2.088989	1.180355
C	-2.262050	-1.275780	1.151560	C	-5.428658	-3.124531	0.084399
O	-0.269773	5.754439	0.092624	O	0.427223	-2.585164	-0.151257
H	-2.926950	2.103317	-0.046589	H	-2.526522	4.553634	0.026640
H	1.723625	3.945797	0.089521	H	3.406756	1.805063	0.064152
H	5.416403	0.370444	0.068415	H	6.455118	-1.865124	0.012387
H	5.025256	-3.904891	-0.080948	H	2.574125	-3.702645	-0.119031
H	-2.468026	-0.197178	-2.062249	H	-4.490866	-1.613875	-1.990070
H	-3.646728	-2.611211	2.096849	H	-1.648280	-1.158921	2.037486
H	-6.245289	-2.706026	-0.508050	H	-5.192098	-4.108097	-0.335278
H	-5.785408	-3.278838	1.104432	H	0.661577	6.003653	0.113678
H	-0.520735	-2.405084	-0.098981				

13o (ground state) b3lyp/6-311+g(d,p) scrf=(cpcm,solvent=acetonitrile)

```
# opt freq b3lyp/6-311+g(d,p)
scrf=(cpcm,solvent=acetonitrile)
```

Charge = 0, Multiplicity = 1, Point group = C1

Electronic Energy = -592.857615 Hartree

Number of imaginary frequencies = 0

Sum of electronic and zero-point Energies = -592.678170 Hartree

Sum of electronic and thermal Energies = -592.668465 Hartree

Sum of electronic and thermal Enthalpies = -592.667520 Hartree

Sum of electronic and thermal Free Energies = -592.713037 Hartree

	Cartesian Coordinates				Cartesian Coordinates		
	X	***Y***	***Z***		***X***	***Y***	***Z***
C	-0.918435	-1.176226	0.000065	C	-0.638356	0.217868	0.000033
C	0.807985	0.356889	-0.000013	C	1.334439	-0.965149	0.000151
N	0.281999	-1.860611	0.000193	C	-2.218712	-1.685381	-0.000124
C	-3.259831	-0.763291	-0.000284	C	-3.022656	0.618395	-0.000136
C	-1.721258	1.112908	0.000113	C	1.704458	1.435677	-0.000350
C	3.074061	1.188423	-0.000287	C	3.568336	-0.126031	-0.000014
C	2.706784	-1.218387	0.000175	O	-1.566758	2.474116	0.000194
H	0.374121	-2.864383	0.000216	H	-2.406598	-2.751931	-0.000152
H	-4.284314	-1.117909	-0.000450	H	-3.847890	1.320363	-0.000112
H	1.363488	2.465481	-0.000735	H	3.767567	2.021078	-0.000518
H	4.639355	-0.293559	0.000007	H	3.087413	-2.233159	0.000310
H	-0.633956	2.711199	0.002557				

13p (ground state) b3lyp/6-311+g(d,p) scrf=(cpcm,solvent=acetonitrile)

```
# opt freq b3lyp/6-311+g(d,p)
scrf=(cpcm,solvent=acetonitrile)
```

Charge = 0, Multiplicity = 1, Point group = C1

Electronic Energy = -746.530553 Hartree

Number of imaginary frequencies = 0

Sum of electronic and zero-point Energies = -746.303766 Hartree

Sum of electronic and thermal Energies = -746.290923 Hartree

Sum of electronic and thermal Enthalpies = -746.289979 Hartree

Sum of electronic and thermal Free Energies = -746.342554 Hartree

	Cartesian Coordinates				Cartesian Coordinates		
	X	***Y***	***Z***		***X***	***Y***	***Z***
C	2.182608	0.987618	-0.099756	C	1.326882	-0.150405	-0.004063
C	-0.041122	0.359122	0.003269	C	0.070938	1.763763	0.002776
N	1.397463	2.119316	-0.083266	C	3.574257	0.904740	-0.183591

C	4.138365	-0.362163	-0.130246	C	3.342519	-1.502198	0.051091
C	1.958670	-1.404334	0.148356	C	-1.355447	-0.218703	-0.044923
C	-2.480400	0.665857	0.098208	C	-2.284366	2.078021	0.182737
C	-1.035936	2.633805	0.101197	O	1.281765	-2.570881	0.412279
C	-1.631466	-1.589515	-0.291239	C	-2.921994	-2.078101	-0.299636
C	-4.016628	-1.219109	-0.076480	C	-3.791971	0.128012	0.101509
H	1.744662	3.065484	-0.111402	H	4.183418	1.795589	-0.274743
H	5.214501	-0.474622	-0.193990	H	3.797074	-2.479531	0.160472
H	-3.156454	2.714702	0.282247	H	-0.891888	3.707661	0.123673
H	0.435345	-2.376926	0.830352	H	-0.825078	-2.264636	-0.542927
H	-3.093381	-3.130003	-0.498819	H	-5.026439	-1.612605	-0.075905
H	-4.627587	0.808254	0.230419				

13q (ground state) b3lyp/6-311+g(d,p) scrf=(cpcm,solvent=acetonitrile)

```
# opt freq b3lyp/6-311+g(d,p)
scrf=(cpcm,solvent=acetonitrile)
```

Charge = 0, Multiplicity = 1, Point group = C1

Electronic Energy = -592.859354 Hartree

Number of imaginary frequencies = 0

Sum of electronic and zero-point Energies = -592.679619 Hartree

Sum of electronic and thermal Energies = -592.669402 Hartree

Sum of electronic and thermal Enthalpies = -592.668458 Hartree

Sum of electronic and thermal Free Energies = -592.715034 Hartree

	**Cartesian Coordinates**				**Cartesian Coordinates**		
	X	***Y***	***Z***		***X***	***Y***	***Z***
C	0.760704	-0.772003	0.000004	C	0.297938	0.569715	0.000004
C	-1.150468	0.510682	0.000002	C	-1.499878	-0.866271	0.000002
N	-0.333194	-1.616763	0.000005	C	2.120946	-1.090953	-0.000015
C	3.024623	-0.031586	-0.000002	C	2.592043	1.308067	0.000008
C	1.236702	1.607820	0.000004	C	-2.165970	1.473747	0.000000
C	-3.494572	1.057291	-0.000003	C	-3.820155	-0.309957	-0.000003
C	-2.829551	-1.289333	-0.000000	O	4.381841	-0.234112	-0.000043
H	-0.290148	-2.623909	-0.000000	H	2.462578	-2.120430	-0.000038
H	3.337038	2.094445	0.000027	H	0.915293	2.643442	0.000010
H	-1.923222	2.530810	0.000001	H	-4.289172	1.794681	-0.000005
H	-4.862032	-0.610056	-0.000006	H	-3.083667	-2.343266	-0.000000
H	4.576778	-1.178790	0.000307				

13r (ground state) b3lyp/6-311+g(d,p) scrf=(cpcm,solvent=acetonitrile)

```
# opt freq b3lyp/6-311+g(d,p)
scrf=(cpcm,solvent=acetonitrile)
```

Charge = 0, Multiplicity = 1, Point group = C1

Electronic Energy = -746.538006 Hartree

Number of imaginary frequencies = 0

Sum of electronic and zero-point Energies = -746.311579 Hartree

Sum of electronic and thermal Energies = -746.298642 Hartree

Sum of electronic and thermal Enthalpies = -746.297698 Hartree

Sum of electronic and thermal Free Energies = -746.350630 Hartree

	**Cartesian Coordinates**				**Cartesian Coordinates**		
	X	***Y***	***Z***		***X***	***Y***	***Z***
C	1.883103	0.892480	-0.000002	C	1.028093	-0.247656	0.000003
C	-0.333384	0.256624	0.000005	C	-0.235390	1.660183	0.000003
N	1.097638	2.024908	-0.000001	C	3.274790	0.798340	0.000007
C	3.828881	-0.476061	-0.000001	C	3.016133	-1.625205	-0.000006
C	1.631434	-1.514862	0.000007	C	-1.627950	-0.350630	0.000002
C	-2.774642	0.515586	0.000001	C	-2.604442	1.933822	0.000000
C	-1.360182	2.511264	0.000001	O	5.198587	-0.564694	-0.000039
C	-1.849725	-1.751836	-0.000001	C	-3.125971	-2.274807	-0.000003
C	-4.250885	-1.422971	-0.000003	C	-4.071810	-0.057226	-0.000002
H	1.440783	2.972932	-0.000005	H	3.911124	1.674894	0.000023
H	3.480731	-2.605623	-0.000021	H	1.041226	-2.421305	0.000013
H	-3.490379	2.559462	-0.000001	H	-1.238754	3.588339	-0.000000
H	5.467139	-1.491033	0.000248	H	-1.005981	-2.428060	-0.000002
H	-3.265600	-3.350194	-0.000005	H	-5.249682	-1.844283	-0.000005
H	-4.931088	0.605800	-0.000002				

13s (ground state) b3lyp/6-311+g(d,p) scrf=(cpcm,solvent=acetonitrile)

```
# opt freq b3lyp/6-311+g(d,p)
scrf=(cpcm,solvent=acetonitrile)
```

Charge = 0, Multiplicity = 1, Point group = C1

Electronic Energy = -592.857684 Hartree

Number of imaginary frequencies = 0

Sum of electronic and zero-point Energies = -592.677890 Hartree

Sum of electronic and thermal Energies = -592.667596 Hartree

Sum of electronic and thermal Enthalpies = -592.666652 Hartree

Sum of electronic and thermal Free Energies = -592.713370 Hartree

	**Cartesian Coordinates**				**Cartesian Coordinates**		
	X	***Y***	***Z***		***X***	***Y***	***Z***
C	-0.968587	-0.489459	-0.000008	C	-0.371712	0.793222	-0.000010

C	1.061244	0.568089	-0.000005	C	1.256770	-0.839778	-0.000001
N	0.019087	-1.457933	-0.000005	C	-2.357996	-0.649557	-0.000006
C	-3.147386	0.493672	-0.000005	C	-2.561200	1.774448	0.000009
C	-1.182414	1.938856	0.000002	C	2.176183	1.415522	-0.000003
C	3.449877	0.857044	0.000004	C	3.623302	-0.539328	0.000008
C	2.534489	-1.405095	0.000006	O	-2.843117	-1.932137	-0.000041
H	-0.143971	-2.452208	0.000010	H	-4.228094	0.393647	-0.000010
H	-3.209125	2.643272	0.000019	H	-0.744757	2.930230	0.000009
H	2.049372	2.492588	-0.000006	H	4.321004	1.502013	0.000006
H	4.626504	-0.950668	0.000013	H	2.672892	-2.480070	0.000008
H	-3.807916	-1.921985	0.000359				

13t (ground state) b3lyp/6-311+g(d,p) scrf=(cpcm,solvent=acetonitrile)

```
# opt freq b3lyp/6-311+g(d,p)
scrf=(cpcm,solvent=acetonitrile)
```

Charge = 0, Multiplicity = 1, Point group = C1

Electronic Energy = -900.206951 Hartree

Number of imaginary frequencies = 0

Sum of electronic and zero-point Energies = -899.933394 Hartree

Sum of electronic and thermal Energies = -899.917873 Hartree

Sum of electronic and thermal Enthalpies = -899.916928 Hartree

Sum of electronic and thermal Free Energies = -899.975433 Hartree

	**Cartesian Coordinates**				**Cartesian Coordinates**		
	X	***Y***	***Z***		***X***	***Y***	***Z***
C	1.200278	-1.603041	0.041962	C	0.632132	-0.315414	-0.004861
C	-0.810338	-0.527543	-0.055831	C	-0.999512	-1.925390	-0.151841
N	0.218832	-2.553236	-0.066238	C	2.583499	-1.847043	0.202653
C	3.427680	-0.774629	0.280253	C	2.934040	0.556844	0.110750
C	1.532275	0.810616	-0.085053	C	-1.989043	0.294282	0.064357
C	-3.267090	-0.328985	-0.150209	C	-3.363132	-1.738652	-0.367248
C	-2.256993	-2.541769	-0.326839	O	2.955821	-3.160713	0.278181
C	-1.991335	1.651444	0.474780	C	-3.159839	2.379925	0.570339
C	-4.401183	1.788647	0.264095	C	-4.446112	0.454205	-0.074204
C	3.845128	1.642117	0.073062	C	3.426002	2.923579	-0.213146
C	2.068763	3.159645	-0.502426	C	1.152588	2.127902	-0.441011
H	0.376352	-3.549040	-0.083643	H	4.492447	-0.927572	0.422484
H	-4.345288	-2.171489	-0.521491	H	-2.332098	-3.617546	-0.433756
H	3.913787	-3.231108	0.372429	H	-1.063738	2.120627	0.764676
H	-3.119870	3.413088	0.897165	H	-5.313283	2.370998	0.325803
H	-5.398711	-0.029355	-0.264852	H	4.896452	1.439771	0.250112

H	4.140810	3.737997	-0.245713	H	1.739629	4.152684	-0.787188
H	0.128267	2.328869	-0.715297				

11* (excited state) b3lyp/6-311+g(d,p) scrf=(cpcm,solvent=acetonitrile)

```
# p opt freq td=(nstates=2)/b3lyp/6-311+g(d,p)
scrf=(cpcm,solvent=acetonitrile)
```

Charge = 0, Multiplicity = 1, Point group = C1

Electronic Energy = -517.604297 Hartree

Number of imaginary frequencies = 0

Sum of electronic and zero-point Energies = -517.298056 Hartree

Sum of electronic and thermal Energies = -517.288549 Hartree

Sum of electronic and thermal Enthalpies = -517.287605 Hartree

Sum of electronic and thermal Free Energies = -517.332391 Hartree

	Cartesian Coordinates				Cartesian Coordinates		
	X	***Y***	***Z***		***X***	***Y***	***Z***
C	1.141589	0.836753	-0.000000	C	0.718002	-0.532923	-0.000000
C	-0.718002	-0.532923	0.000000	C	-1.141589	0.836753	0.000000
N	0.000000	1.613754	0.000000	C	2.469357	1.244494	-0.000000
C	3.459528	0.219632	0.000000	C	3.072887	-1.119579	0.000000
C	1.712617	-1.527218	-0.000000	C	-1.712617	-1.527218	0.000000
C	-3.072887	-1.119579	-0.000000	C	-3.459528	0.219632	-0.000000
C	-2.469357	1.244494	0.000000	H	0.000000	2.626901	-0.000000
H	2.736085	2.294438	-0.000001	H	4.509022	0.484042	0.000000
H	3.840128	-1.886808	0.000001	H	1.459684	-2.580216	-0.000000
H	-1.459684	-2.580216	-0.000000	H	-3.840128	-1.886808	-0.000000
H	-4.509022	0.484042	-0.000000	H	-2.736085	2.294438	0.000000

13a* (excited state) b3lyp/6-311+g(d,p) scrf=(cpcm,solvent=acetonitrile)

```
# p opt freq td=(nstates=2)/b3lyp/6-311+g(d,p)
scrf=(cpcm,solvent=acetonitrile)
```

Charge = 0, Multiplicity = 1, Point group = C1

Electronic Energy = -671.280195 Hartree

Number of imaginary frequencies = 0

Sum of electronic and zero-point Energies = -670.944910 Hartree

Sum of electronic and thermal Energies = -670.932786 Hartree

Sum of electronic and thermal Enthalpies = -670.931842 Hartree

Sum of electronic and thermal Free Energies = -670.983013 Hartree

	Cartesian Coordinates				Cartesian Coordinates		
	X	***Y***	***Z***		***X***	***Y***	***Z***
C	-2.303016	0.701382	-0.000004	C	-1.360906	-0.374065	0.000001

C	-0.059447	0.206296	-0.000001	C	-0.248061	1.659317	-0.000003
N	-1.585371	1.901346	-0.000007	C	-3.667367	0.512453	-0.000005
C	-4.141632	-0.824149	0.000002	C	-3.254355	-1.897820	0.000009
C	-1.867828	-1.697270	0.000009	C	1.277082	-0.329928	-0.000003
C	2.362467	0.628338	0.000005	C	2.101810	2.026029	0.000006
C	0.803059	2.566353	0.000001	C	1.573069	-1.698915	-0.000011
C	2.909228	-2.171295	-0.000009	C	3.949924	-1.253140	0.000001
C	3.686996	0.126655	0.000008	H	-2.003159	2.820397	-0.000007
H	-4.354936	1.349381	-0.000009	H	-5.209956	-1.005227	0.000001
H	-3.641797	-2.910055	0.000015	H	-1.209842	-2.554374	0.000017
H	2.952413	2.699487	0.000011	H	0.629602	3.634273	0.000000
H	0.770626	-2.422464	-0.000022	H	3.106054	-3.236074	-0.000016
H	4.979178	-1.595723	0.000004	H	4.513284	0.829512	0.000016

13b* (excited state) b3lyp/6-311+g(d,p) scrf=(cpcm,solvent=acetonitrile)

```
# p opt freq td=(nstates=2)/b3lyp/6-311+g(d,p)
scrf=(cpcm,solvent=acetonitrile)
```

Charge = 0, Multiplicity = 1, Point group = C1

Electronic Energy = -671.279902 Hartree

Number of imaginary frequencies = 0

Sum of electronic and zero-point Energies = -670.942363 Hartree

Sum of electronic and thermal Energies = -670.930147 Hartree

Sum of electronic and thermal Enthalpies = -670.929203 Hartree

Sum of electronic and thermal Free Energies = -670.980336 Hartree

	**Cartesian Coordinates**				**Cartesian Coordinates**		
	X	***Y***	***Z***		***X***	***Y***	***Z***
C	1.996501	-0.873939	0.000000	C	1.940080	0.544068	-0.000000
C	0.543144	0.904051	-0.000001	C	-0.220776	-0.340675	-0.000001
N	0.680216	-1.360560	-0.000000	C	3.188074	-1.576480	0.000001
C	4.384505	-0.822286	0.000001	C	4.355075	0.568331	0.000000
C	3.134923	1.270407	-0.000000	C	-0.153778	2.099884	-0.000001
C	-1.554239	2.066182	-0.000000	C	-2.322770	0.853908	0.000000
C	-1.631530	-0.407938	-0.000001	C	-3.726771	0.873982	0.000002
C	-4.470709	-0.344694	0.000001	C	-3.802689	-1.554865	-0.000000
C	-2.388037	-1.613111	-0.000001	H	0.444455	-2.343040	0.000000
H	3.209778	-2.659390	0.000001	H	5.335026	-1.342140	0.000002
H	5.285966	1.123181	0.000000	H	3.127258	2.353919	-0.000001
H	0.362055	3.052617	-0.000001	H	-2.096930	3.005515	-0.000000
H	-4.242785	1.827293	0.000002	H	-5.553830	-0.314999	0.000002
H	-4.364613	-2.482963	-0.000001	H	-1.893903	-2.577038	-0.000003

13c* (excited state) b3lyp/6-311+g(d,p) scrf=(cpcm,solvent=acetonitrile)

```
# p opt freq td=(nstates=2)/b3lyp/6-311+g(d,p)
scrf=(cpcm,solvent=acetonitrile)
```

Charge = 0, Multiplicity = 1, Point group = C1

Electronic Energy = -671.280541 Hartree

Number of imaginary frequencies = 0

Sum of electronic and zero-point Energies = -670.953515 Hartree

Sum of electronic and thermal Energies = -670.941533 Hartree

Sum of electronic and thermal Enthalpies = -670.940589 Hartree

Sum of electronic and thermal Free Energies = -670.991278 Hartree

	Cartesian Coordinates				Cartesian Coordinates		
	X	***Y***	***Z***		***X***	***Y***	***Z***
C	2.434112	-0.806562	0.000000	C	1.859799	0.514273	0.000000
C	0.438532	0.352894	-0.000001	C	0.178908	-1.047877	-0.000001
N	1.392088	-1.706831	-0.000000	C	3.804689	-1.044066	0.000000
C	4.649488	0.073542	0.000001	C	4.112321	1.375879	0.000001
C	2.735478	1.614885	0.000000	C	-0.659953	1.232524	-0.000001
C	-1.990693	0.722373	-0.000001	C	-2.223316	-0.707367	-0.000001
C	-1.096336	-1.595011	-0.000001	C	-3.119227	1.571296	-0.000001
C	-4.429177	1.057436	0.000001	C	-4.648188	-0.313041	0.000001
C	-3.540206	-1.188923	0.000000	H	1.500426	-2.712658	-0.000000
H	4.199112	-2.053235	0.000000	H	5.723606	-0.063967	0.000001
H	4.792221	2.220939	0.000000	H	2.355076	2.629348	-0.000000
H	-0.509656	2.306798	-0.000001	H	-1.248422	-2.668309	-0.000001
H	-2.966576	2.645558	-0.000001	H	-5.268504	1.744306	0.000002
H	-5.655160	-0.712765	0.000003	H	-3.704120	-2.261731	0.000001

13d* (excited state) b3lyp/6-311+g(d,p) scrf=(cpcm,solvent=acetonitrile)

```
# p opt freq td=(nstates=2)/b3lyp/6-311+g(d,p)
scrf=(cpcm,solvent=acetonitrile)
```

Charge = 0, Multiplicity = 1, Point group = C1

Electronic Energy = -788.030891 Hartree

Number of imaginary frequencies = 0

Sum of electronic and zero-point Energies = -787.624037 Hartree

Sum of electronic and thermal Energies = -787.608083 Hartree

Sum of electronic and thermal Enthalpies = -787.607139 Hartree

Sum of electronic and thermal Free Energies = -787.667990 Hartree

	Cartesian Coordinates				Cartesian Coordinates		
	X	***Y***	***Z***		***X***	***Y***	***Z***

C	-0.941913	1.152093	-0.034524	C	-2.298842	0.710240	-0.021668
C	-2.295210	-0.718314	0.023830	C	-0.936040	-1.153156	0.035559
N	-0.123603	0.001443	-0.000004	C	-0.571868	2.482148	-0.145780
C	-1.618072	3.451370	-0.201183	C	-2.949650	3.041958	-0.167187
C	-3.320161	1.682583	-0.083973	C	-3.311404	-1.695963	0.086928
C	-2.933712	-3.053393	0.169815	C	-1.599979	-3.455905	0.202790
C	-0.558906	-2.481250	0.146670	C	1.279242	0.004176	-0.001628
C	1.987588	-0.856010	-0.859271	C	3.372809	-0.838106	-0.861340
C	4.094329	0.010951	-0.007135	C	3.372018	0.859845	0.846541
C	1.986760	0.869672	0.851353	C	5.597825	-0.006919	0.011682
H	0.463576	2.787360	-0.204398	H	-1.369188	4.502168	-0.274861
H	-3.732440	3.792252	-0.215284	H	-4.364957	1.397277	-0.081462
H	-4.357684	-1.416155	0.084997	H	-3.712543	-3.807760	0.218474
H	-1.345629	-4.505400	0.276366	H	0.478245	-2.780830	0.204444
H	1.447613	-1.491971	-1.547731	H	3.907550	-1.484619	-1.547905
H	3.906486	1.515897	1.524267	H	1.446525	1.507632	1.537713
H	6.004598	-0.289446	-0.961063	H	6.004249	0.965864	0.294639
H	5.959944	-0.738477	0.742963				

13e* (excited state) b3lyp/6-311+g(d,p) scrf=(cpcm,solvent=acetonitrile)

```
# p opt freq td=(nstates=2)/b3lyp/6-311+g(d,p)
scrf=(cpcm,solvent=acetonitrile)
```

Charge = 0, Multiplicity = 1, Point group = C1

Electronic Energy = -941.708652 Hartree

Number of imaginary frequencies = 0

Sum of electronic and zero-point Energies = -941.267208 Hartree

Sum of electronic and thermal Energies = -941.248383 Hartree

Sum of electronic and thermal Enthalpies = -941.247439 Hartree

Sum of electronic and thermal Free Energies = -941.315864 Hartree

	Cartesian Coordinates				Cartesian Coordinates		
	X	*Y*	*Z*		*X*	*Y*	*Z*
C	-0.355360	1.574454	0.027663	C	1.068343	1.592161	0.042478
C	1.510546	0.231391	0.000688	C	0.314069	-0.601403	-0.040828
N	-0.779209	0.222257	-0.023195	C	-1.127128	2.712362	0.089914
C	-0.452417	3.962094	0.150825	C	0.933766	4.017258	0.156917
C	1.711813	2.846571	0.107443	C	2.803271	-0.390591	-0.017786
C	2.831761	-1.838143	-0.090141	C	1.623253	-2.588023	-0.144990
C	0.354231	-1.991317	-0.121458	C	4.016907	0.316863	0.028060
C	5.262302	-0.346458	0.006649	C	5.288359	-1.735511	-0.062781
C	4.095881	-2.472823	-0.111036	C	-2.139505	-0.203123	-0.015866

C	-3.006008	0.206955	-1.032171	C	-4.332710	-0.211679	-1.017490
C	-4.823413	-1.040158	-0.000016	C	-3.936702	-1.442847	1.006964
C	-2.608097	-1.030592	1.007525	C	-6.270386	-1.463527	0.025599
H	-2.208066	2.667876	0.095138	H	-1.034974	4.874474	0.193052
H	1.432347	4.978600	0.203953	H	2.788573	2.930745	0.126344
H	1.698645	-3.668091	-0.210700	H	-0.548888	-2.582783	-0.168636
H	4.007809	1.395590	0.082441	H	6.181353	0.224974	0.043658
H	6.237073	-2.261329	-0.080457	H	4.137130	-3.555350	-0.166589
H	-2.638610	0.833449	-1.835750	H	-4.994375	0.104016	-1.816884
H	-4.290857	-2.083346	1.807468	H	-1.938315	-1.338445	1.801037
H	-6.682828	-1.530562	-0.983204	H	-6.392747	-2.431751	0.515246
H	-6.875462	-0.737336	0.579264				

13f* (excited state) b3lyp/6-311+g(d,p) scrf=(cpcm,solvent=acetonitrile)

```
# p opt freq td=(nstates=2)/b3lyp/6-311+g(d,p)
scrf=(cpcm,solvent=acetonitrile)
```

Charge = 0, Multiplicity = 1, Point group = C1

Electronic Energy = -941.703358 Hartree

Number of imaginary frequencies = 0

Sum of electronic and zero-point Energies = -941.260815 Hartree

Sum of electronic and thermal Energies = -941.242071 Hartree

Sum of electronic and thermal Enthalpies = -941.241127 Hartree

Sum of electronic and thermal Free Energies = -941.308466 Hartree

	Cartesian Coordinates				Cartesian Coordinates		
	X	*Y*	*Z*		*X*	*Y*	*Z*
C	0.233779	2.031203	-0.100616	C	1.636316	2.153140	0.034978
C	2.165867	0.819207	0.108192	C	1.050757	-0.112505	-0.011521
N	-0.099340	0.646421	-0.121469	C	-0.606166	3.129621	-0.138580
C	-0.009441	4.410913	-0.077181	C	1.369321	4.552347	0.035023
C	2.210082	3.427712	0.099464	C	3.449301	0.326000	0.268412
C	3.644880	-1.055331	0.274924	C	2.585969	-1.995831	0.068806
C	1.231419	-1.521081	-0.107499	C	2.862357	-3.372025	0.002203
C	1.835010	-4.311947	-0.259930	C	0.543944	-3.858030	-0.466366
C	0.224323	-2.485099	-0.393347	C	-1.446992	0.198952	-0.031128
C	-2.367487	0.536929	-1.031041	C	-3.687596	0.120106	-0.925107
C	-4.128380	-0.632458	0.177989	C	-3.195613	-0.951087	1.171040
C	-1.866721	-0.545031	1.078951	C	-5.565558	-1.075540	0.278352
H	-1.680248	3.026773	-0.211839	H	-0.643255	5.288677	-0.114341
H	1.804591	5.543987	0.080822	H	3.281756	3.550391	0.202628
H	4.294767	0.994235	0.382221	H	4.648506	-1.445185	0.406988

H	3.884764	-3.705959	0.139861	H	2.063792	-5.370010	-0.306871
H	-0.250018	-4.564676	-0.684625	H	-0.795315	-2.180044	-0.564170
H	-2.039665	1.099948	-1.896570	H	-4.386996	0.374427	-1.714364
H	-3.512126	-1.521921	2.037502	H	-1.160567	-0.790918	1.862346
H	-5.842101	-1.709012	-0.570422	H	-5.745077	-1.640275	1.194633
H	-6.243647	-0.216373	0.270373				

13g* (excited state) b3lyp/6-311+g(d,p) scrf=(cpcm,solvent=acetonitrile)

```
# p opt freq td=(nstates=2)/b3lyp/6-311+g(d,p)
scrf=(cpcm,solvent=acetonitrile)
```

Charge = 0, Multiplicity = 1, Point group = C1

Electronic Energy = -941.708965 Hartree

Number of imaginary frequencies = 0

Sum of electronic and zero-point Energies = -941.276940 Hartree

Sum of electronic and thermal Energies = -941.258360 Hartree

Sum of electronic and thermal Enthalpies = -941.257416 Hartree

Sum of electronic and thermal Free Energies = -941.324395 Hartree

	**Cartesian Coordinates**				**Cartesian Coordinates**		
	X	***Y***	***Z***		***X***	***Y***	***Z***
C	-0.835223	1.860570	0.026629	C	0.508121	2.365256	0.044745
C	1.388374	1.239831	0.036063	C	0.577317	0.072144	0.008981
N	-0.766336	0.467568	0.009430	C	-1.953427	2.685703	0.076095
C	-1.735602	4.070770	0.112926	C	-0.427721	4.587597	0.111581
C	0.696260	3.758147	0.082678	C	2.785879	1.088893	0.018071
C	3.367826	-0.210598	-0.024996	C	2.519107	-1.382453	-0.067616
C	1.093266	-1.212356	-0.060730	C	4.766675	-0.411057	-0.038318
C	5.324436	-1.698931	-0.089189	C	4.506284	-2.822086	-0.130342
C	3.106407	-2.655407	-0.119698	C	-1.882667	-0.410523	0.005229
C	-2.874749	-0.277320	-0.972300	C	-3.961250	-1.143343	-0.971137
C	-4.090030	-2.150561	-0.004739	C	-3.084308	-2.267835	0.963687
C	-1.989238	-1.411931	0.976356	C	-5.288886	-3.063479	0.006881
H	-2.956150	2.279495	0.090427	H	-2.582076	4.745092	0.146839
H	-0.288446	5.662786	0.141708	H	1.692565	4.183388	0.097358
H	3.436350	1.956665	0.030909	H	0.450391	-2.081326	-0.117512
H	5.419834	0.455019	-0.011292	H	6.403338	-1.810473	-0.097742
H	4.934208	-3.816627	-0.170959	H	2.461214	-3.527520	-0.152398
H	-2.777496	0.478558	-1.741269	H	-4.716580	-1.041986	-1.742645
H	-3.161601	-3.035879	1.725375	H	-1.231875	-1.501608	1.744754
H	-5.668480	-3.233513	-1.002722	H	-5.049173	-4.029306	0.455730
H	-6.102772	-2.621918	0.592305				

13h* (excited state) b3lyp/6-311+g(d,p) scrf=(cpcm,solvent=acetonitrile)

```
# p opt freq td=(nstates=2)/b3lyp/6-311+g(d,p)
scrf=(cpcm,solvent=acetonitrile)
```

Charge = 0, Multiplicity = 1, Point group = C1

Electronic Energy = -592.850545 Hartree

Number of imaginary frequencies = 0

Sum of electronic and zero-point Energies = -592.552666 Hartree

Sum of electronic and thermal Energies = -592.542102 Hartree

Sum of electronic and thermal Enthalpies = -592.541158 Hartree

Sum of electronic and thermal Free Energies = -592.588331 Hartree

	Cartesian Coordinates				Cartesian Coordinates		
	X	*Y*	*Z*		*X*	*Y*	*Z*
C	0.653846	1.139019	0.000001	C	0.418254	-0.291408	0.000002
C	-0.999930	-0.487304	-0.000000	C	-1.602818	0.800237	-0.000002
N	-0.560896	1.746097	-0.000002	C	1.932260	1.711141	0.000002
C	3.034633	0.843893	0.000002	C	2.818423	-0.552153	-0.000001
C	1.526233	-1.142961	-0.000002	C	-1.841550	-1.625893	0.000003
C	-3.239389	-1.419260	0.000002	C	-3.802589	-0.149263	-0.000000
C	-2.964431	1.015811	-0.000002	O	3.924432	-1.326132	-0.000001
H	-0.700817	2.747934	-0.000006	H	2.062995	2.785966	0.000004
H	4.049927	1.216575	0.000008	H	1.423965	-2.222060	-0.000012
H	-1.431618	-2.628527	0.000005	H	-3.893267	-2.285615	0.000004
H	-4.879108	-0.030202	-0.000001	H	-3.384098	2.014213	-0.000003
H	3.685173	-2.263064	-0.000007				

13i* (excited state) b3lyp/6-311+g(d,p) scrf=(cpcm,solvent=acetonitrile)

```
# p opt freq td=(nstates=2)/b3lyp/6-311+g(d,p)
scrf=(cpcm,solvent=acetonitrile)
```

Charge = 0, Multiplicity = 1, Point group = C1

Electronic Energy = -746.527683 Hartree

Number of imaginary frequencies = 0

Sum of electronic and zero-point Energies = -746.192689 Hartree

Sum of electronic and thermal Energies = -746.179453 Hartree

Sum of electronic and thermal Enthalpies = -746.178509 Hartree

Sum of electronic and thermal Free Energies = -746.231994 Hartree

	Cartesian Coordinates				Cartesian Coordinates		
	X	*Y*	*Z*		*X*	*Y*	*Z*
C	-1.900856	-1.233540	0.000006	C	-1.127569	-0.002798	0.000003
C	0.264193	-0.386163	0.000003	C	0.302287	-1.798758	0.000003

N	-1.032028	-2.264087	0.000006	C	-3.307286	-1.260353	0.000006
C	-3.982094	-0.048828	0.000001	C	-3.246352	1.158530	-0.000005
C	-1.827002	1.196084	-0.000006	C	1.494355	0.356600	0.000004
C	2.726524	-0.425841	-0.000005	C	2.684993	-1.828630	-0.000009
C	1.460670	-2.551575	-0.000003	C	1.578432	1.760520	0.000014
C	2.812621	2.422238	0.000012	C	4.004403	1.673720	-0.000001
C	3.970220	0.293036	-0.000010	O	-3.966621	2.294618	-0.000012
H	-1.295509	-3.240038	0.000006	H	-3.842992	-2.201282	0.000011
H	-5.062768	-0.001585	0.000003	H	-1.332492	2.157521	-0.000016
H	3.621274	-2.375804	-0.000016	H	1.440741	-3.633802	-0.000004
H	0.673677	2.352843	0.000026	H	2.846927	3.505439	0.000020
H	4.960650	2.187303	-0.000005	H	4.893747	-0.276777	-0.000020
H	-3.391336	3.072383	-0.000009				

13j* (excited state) b3lyp/6-311+g(d,p) scrf=(cpcm,solvent=acetonitrile)

```
# p opt freq td=(nstates=2)/b3lyp/6-311+g(d,p)
scrf=(cpcm,solvent=acetonitrile)
```

Charge = 0, Multiplicity = 1, Point group = C1

Electronic Energy = -746.530455 Hartree

Number of imaginary frequencies = 0

Sum of electronic and zero-point Energies = -746.190268 Hartree

Sum of electronic and thermal Energies = -746.176777 Hartree

Sum of electronic and thermal Enthalpies = -746.175833 Hartree

Sum of electronic and thermal Free Energies = -746.229651 Hartree

	Cartesian Coordinates				Cartesian Coordinates		
	X	***Y***	***Z***		***X***	***Y***	***Z***
C	1.576366	-1.051702	-0.000002	C	1.591996	0.373165	0.000000
C	0.211701	0.802573	0.000000	C	-0.610169	-0.388405	-0.000001
N	0.247477	-1.460812	-0.000003	C	2.737019	-1.810497	-0.000003
C	3.975044	-1.129376	0.000001	C	4.002235	0.263523	0.000003
C	2.814779	1.037738	0.000001	C	-0.422256	2.037573	-0.000003
C	-1.820055	2.085342	-0.000003	C	-2.648462	0.917288	-0.000000
C	-2.016583	-0.383618	0.000000	C	-4.049140	1.008074	0.000000
C	-4.853418	-0.159871	0.000002	C	-4.241886	-1.409929	0.000003
C	-2.844517	-1.545995	0.000001	O	5.167465	0.973639	0.000011
H	-0.041329	-2.429264	-0.000002	H	2.706342	-2.893116	-0.000005
H	4.901485	-1.691434	0.000004	H	2.888709	2.118009	-0.000001
H	0.146097	2.960430	-0.000004	H	-2.309492	3.053422	-0.000007
H	-4.511538	1.989041	-0.000001	H	-5.933425	-0.077351	0.000004
H	-4.854204	-2.305998	0.000004	H	-2.404631	-2.536228	0.000001

H	5.924019	0.373755	-0.000056

13k* (excited state) b3lyp/6-311+g(d,p) scrf=(cpcm,solvent=acetonitrile)

```
# p opt freq td=(nstates=2)/b3lyp/6-311+g(d,p)
scrf=(cpcm,solvent=acetonitrile)
```

Charge = 0, Multiplicity = 1, Point group = C1

Electronic Energy = -746.528036 Hartree

Number of imaginary frequencies = 0

Sum of electronic and zero-point Energies = -746.205142 Hartree

Sum of electronic and thermal Energies = -746.191955 Hartree

Sum of electronic and thermal Enthalpies = -746.191011 Hartree

Sum of electronic and thermal Free Energies = -746.244281 Hartree

	**Cartesian Coordinates**				**Cartesian Coordinates**		
	X	***Y***	***Z***		***X***	***Y***	***Z***
C	-2.010222	1.166765	-0.000000	C	-1.555396	-0.205976	0.000001
C	-0.123306	-0.172680	0.000002	C	0.259732	1.196726	0.000002
N	-0.904086	1.964673	0.000001	C	-3.362489	1.516344	-0.000002
C	-4.300633	0.488406	-0.000002	C	-3.870626	-0.859801	-0.000001
C	-2.511857	-1.222642	0.000000	C	0.884970	-1.153388	0.000002
C	2.254821	-0.766691	0.000002	C	2.615096	0.637199	0.000001
C	1.570971	1.627920	0.000002	C	3.304497	-1.717761	-0.000000
C	4.649064	-1.327043	-0.000002	C	4.990714	0.022844	-0.000003
C	3.971696	0.994177	-0.000001	O	-4.851384	-1.795526	-0.000000
H	-0.923592	2.976024	0.000000	H	-3.671856	2.554233	-0.000003
H	-5.362267	0.696951	-0.000004	H	-2.228790	-2.269085	0.000002
H	0.634425	-2.208618	0.000002	H	1.821335	2.682683	0.000002
H	3.051137	-2.773082	-0.000000	H	5.424991	-2.085167	-0.000004
H	6.030587	0.328221	-0.000005	H	4.233965	2.047495	-0.000001
H	-4.472450	-2.684564	0.000002				

13l* (excited state) b3lyp/6-311+g(d,p) scrf=(cpcm,solvent=acetonitrile)

```
# p opt freq td=(nstates=2)/b3lyp/6-311+g(d,p)
scrf=(cpcm,solvent=acetonitrile)
```

Charge = 0, Multiplicity = 1, Point group = C1

Electronic Energy = -863.279487 Hartree

Number of imaginary frequencies = 0

Sum of electronic and zero-point Energies = -862.877020 Hartree

Sum of electronic and thermal Energies = -862.859835 Hartree

Sum of electronic and thermal Enthalpies = -862.858891 Hartree

Sum of electronic and thermal Free Energies = -862.922609 Hartree

	Cartesian Coordinates				Cartesian Coordinates		
	X	*Y*	*Z*		*X*	*Y*	*Z*
C	0.879851	-0.757341	-0.040194	C	2.135897	-0.052758	0.001295
C	1.847114	1.345673	0.042602	C	0.434705	1.493271	0.025807
N	-0.130225	0.184216	-0.022910	C	0.793601	-2.147645	-0.140199
C	1.992359	-2.885177	-0.162755	C	3.223899	-2.206332	-0.101413
C	3.323588	-0.795570	-0.024563	C	2.650259	2.508931	0.118236
C	2.004603	3.761617	0.176624	C	0.620168	3.880899	0.171407
C	-0.206987	2.711325	0.101888	C	-1.517965	-0.098070	-0.012268
C	-2.367010	0.518353	-0.940008	C	-3.724654	0.226606	-0.929801
C	-4.272296	-0.668591	0.000337	C	-3.406938	-1.272627	0.922030
C	-2.044953	-0.997489	0.922499	C	-5.752185	-0.949938	0.024494
O	4.339130	-2.975599	-0.129620	H	-0.161146	-2.650980	-0.206462
H	1.987251	-3.964334	-0.234320	H	4.297037	-0.319111	0.002052
H	3.731158	2.442592	0.141159	H	2.611755	4.659674	0.232369
H	0.154798	4.857463	0.220617	H	-1.284941	2.794541	0.116370
H	-1.957930	1.194591	-1.679450	H	-4.369729	0.695408	-1.664800
H	-3.805242	-1.963308	1.657084	H	-1.393989	-1.454448	1.656832
H	-6.180682	-0.908517	-0.979113	H	-5.963882	-1.931251	0.453184
H	-6.275350	-0.204685	0.633668	H	5.131087	-2.423171	-0.087967

13m* (excited state) b3lyp/6-311+g(d,p) scrf=(cpcm,solvent=acetonitrile)

```
# p opt freq td=(nstates=2)/b3lyp/6-311+g(d,p)
scrf=(cpcm,solvent=acetonitrile)
```

Charge = 0, Multiplicity = 1, Point group = C1

Electronic Energy = -1170.632100 Hartree

Number of imaginary frequencies = 0

Sum of electronic and zero-point Energies = -1170.142444 Hartree

Sum of electronic and thermal Energies = -1170.119315 Hartree

Sum of electronic and thermal Enthalpies = -1170.118371 Hartree

Sum of electronic and thermal Free Energies = -1170.196786 Hartree

	Cartesian Coordinates				Cartesian Coordinates		
	X	*Y*	*Z*		*X*	*Y*	*Z*
C	-0.443224	1.812737	-0.032438	C	0.972139	1.846851	-0.025558
C	1.419782	0.480929	-0.007588	C	0.230700	-0.366409	-0.026156
N	-0.858449	0.462946	-0.028632	C	-1.229658	2.946313	-0.037945
C	-0.582124	4.203960	-0.046741	C	0.805356	4.269107	-0.051117
C	1.603047	3.108124	-0.040728	C	2.720721	-0.100965	0.039190
C	2.826555	-1.543140	0.016022	C	1.631064	-2.372250	-0.042000
C	0.299669	-1.778279	-0.055570	C	1.714889	-3.778863	-0.083219

C	0.596533	-4.596231	-0.138402	C	-0.692711	-4.016188	-0.156718
C	-0.828772	-2.645023	-0.115468	C	-2.253600	0.123405	0.009414
C	-2.987217	0.072515	-1.174265	C	-4.345627	-0.231575	-1.127047
C	-4.992751	-0.476031	0.090524	C	-4.236123	-0.406928	1.267261
C	-2.877547	-0.103803	1.235032	C	-6.470312	-0.776762	0.136529
O	1.377836	5.516500	-0.066590	C	3.898341	0.681634	0.110720
C	5.165515	0.100019	0.146591	C	5.269736	-1.290412	0.114705
C	4.126507	-2.092149	0.052716	H	-2.309907	2.886159	-0.037350
H	-1.159257	5.119847	-0.051709	H	2.677565	3.220847	-0.055242
H	2.685250	-4.257351	-0.074753	H	0.715037	-5.672916	-0.169703
H	-1.575367	-4.643291	-0.204526	H	-1.823111	-2.226444	-0.133032
H	-2.495102	0.255437	-2.122084	H	-4.908885	-0.281221	-2.052645
H	-4.714021	-0.592078	2.223238	H	-2.300792	-0.054532	2.150992
H	-6.806202	-1.260412	-0.782862	H	-6.717704	-1.425522	0.979255
H	-7.048667	0.146123	0.252539	H	2.339178	5.439125	-0.059665
H	3.822826	1.756263	0.147502	H	6.050774	0.721662	0.200436
H	6.245089	-1.763941	0.141084	H	4.264414	-3.163884	0.035199

13n* (excited state) b3lyp/6-311+g(d,p) scrf=(cpcm,solvent=acetonitrile)

```
# p opt freq td=(nstates=2)/b3lyp/6-311+g(d,p)
scrf=(cpcm,solvent=acetonitrile)
```

Charge = 0, Multiplicity = 1, Point group = C1

Electronic Energy = -1092.201755 Hartree

Number of imaginary frequencies = 0

Sum of electronic and zero-point Energies = -1091.769288 Hartree

Sum of electronic and thermal Energies = -1091.748993 Hartree

Sum of electronic and thermal Enthalpies = -1091.748049 Hartree

Sum of electronic and thermal Free Energies = -1091.818270 Hartree

	**Cartesian Coordinates**				**Cartesian Coordinates**		
	X	***Y***	***Z***		***X***	***Y***	***Z***
C	-0.877252	1.626033	0.006994	C	0.442697	2.181972	0.022149
C	1.361116	1.091671	0.025331	C	0.586968	-0.112492	0.009128
N	-0.761037	0.239119	-0.000037	C	-2.025235	2.408651	0.018698
C	-1.866698	3.800807	0.032664	C	-0.574718	4.363906	0.038902
C	0.585773	3.581634	0.035731	C	2.758536	1.005113	0.021248
C	3.403786	-0.260215	-0.004005	C	2.623846	-1.472444	-0.032524
C	1.182268	-1.371688	-0.029715	C	4.816046	-0.383825	-0.007185
C	5.436971	-1.638998	-0.038415	C	4.677053	-2.803103	-0.067474
C	3.267933	-2.715755	-0.064092	C	-1.885295	-0.651871	0.000360
C	-2.641899	-0.812641	-1.160757	C	-3.743314	-1.665627	-1.148794

C	-4.109033	-2.364902	0.007115	C	-3.337976	-2.183785	1.163440
C	-2.237871	-1.331073	1.171169	C	-5.301929	-3.287312	0.019000
O	-0.513662	5.728726	0.051513	O	0.507020	-2.549159	-0.074663
H	-3.011988	1.963808	0.015869	H	-2.725820	4.458833	0.040099
H	1.563246	4.050595	0.046394	H	3.365019	1.904319	0.031425
H	5.418686	0.517763	0.014476	H	6.520248	-1.696906	-0.040550
H	5.156742	-3.774382	-0.092732	H	2.672057	-3.618836	-0.086113
H	-2.362769	-0.282786	-2.063318	H	-4.322572	-1.791168	-2.056673
H	-3.604497	-2.708751	2.074243	H	-1.659369	-1.184550	2.075783
H	-5.763402	-3.354115	-0.967184	H	-5.014039	-4.295214	0.330780
H	-6.059344	-2.934838	0.725449	H	0.407018	6.018975	0.055662
H	-0.449468	-2.407430	-0.031965				

13o* (excited state) b3lyp/6-311+g(d,p) scrf=(cpcm,solvent=acetonitrile)

```
# p opt freq td=(nstates=2)/b3lyp/6-311+g(d,p)
scrf=(cpcm,solvent=acetonitrile)
```

Charge = 0, Multiplicity = 1, Point group = C1

Electronic Energy = -592.853028 Hartree

Number of imaginary frequencies = 0

Sum of electronic and zero-point Energies = -592.543041 Hartree

Sum of electronic and thermal Energies = -592.532130 Hartree

Sum of electronic and thermal Enthalpies = -592.531186 Hartree

Sum of electronic and thermal Free Energies = -592.579199 Hartree

	Cartesian Coordinates				Cartesian Coordinates		
	X	*Y*	*Z*		*X*	*Y*	*Z*
C	-0.920857	-1.161989	0.000037	C	-0.618407	0.236610	-0.000002
C	0.803458	0.379398	-0.000044	C	1.348138	-0.952267	0.000061
N	0.287917	-1.831779	0.000087	C	-2.203756	-1.691642	-0.000068
C	-3.299306	-0.782219	-0.000152	C	-3.048593	0.585036	-0.000065
C	-1.726892	1.106839	0.000078	C	1.722442	1.467874	-0.000263
C	3.101620	1.173435	-0.000138	C	3.602899	-0.132585	0.000058
C	2.711946	-1.242719	0.000110	O	-1.618154	2.461996	0.000186
H	0.381689	-2.839241	0.000105	H	-2.365193	-2.762614	-0.000092
H	-4.314974	-1.153043	-0.000242	H	-3.865274	1.297791	-0.000050
H	1.402189	2.502602	-0.000624	H	3.804378	2.000729	-0.000241
H	4.672852	-0.299538	0.000128	H	3.069289	-2.264887	0.000176
H	-0.691299	2.730047	0.001071				

13p* (excited state) b3lyp/6-311+g(d,p) scrf=(cpcm,solvent=acetonitrile)

```
# p opt freq td=(nstates=2)/b3lyp/6-311+g(d,p)
scrf=(cpcm,solvent=acetonitrile)
```

Charge = 0, Multiplicity = 1, Point group = C1

Electronic Energy = -746.521317 Hartree

Number of imaginary frequencies = 0

Sum of electronic and zero-point Energies = -746.187961 Hartree

Sum of electronic and thermal Energies = -746.174912 Hartree

Sum of electronic and thermal Enthalpies = -746.173968 Hartree

Sum of electronic and thermal Free Energies = -746.226791 Hartree

	**Cartesian Coordinates**				**Cartesian Coordinates**		
	X	***Y***	***Z***		***X***	***Y***	***Z***
C	2.198399	0.966845	-0.130913	C	1.298093	-0.139987	-0.026340
C	-0.029334	0.382829	-0.064773	C	0.104875	1.830142	-0.063745
N	1.432683	2.127956	-0.162433	C	3.567776	0.837915	-0.167550
C	4.109474	-0.470035	-0.053911	C	3.287978	-1.564618	0.128459
C	1.883986	-1.416095	0.185016	C	-1.346407	-0.206561	-0.128619
C	-2.451015	0.686459	0.148201	C	-2.236134	2.085983	0.249796
C	-0.977698	2.692095	0.085250	O	1.181880	-2.520099	0.503263
C	-1.612504	-1.541216	-0.461618	C	-2.930035	-2.073709	-0.396166
C	-3.974522	-1.242171	-0.012392	C	-3.753678	0.116705	0.247993
H	1.814083	3.062750	-0.151631	H	4.214574	1.700159	-0.267976
H	5.183507	-0.603100	-0.084133	H	3.698910	-2.556656	0.268326
H	-3.103962	2.717346	0.411735	H	-0.851088	3.766158	0.105387
H	0.229107	-2.332126	0.544039	H	-0.829403	-2.164834	-0.877368
H	-3.106630	-3.112302	-0.646571	H	-4.981561	-1.639218	0.066134
H	-4.586893	0.759425	0.510858				

13q* (excited state) b3lyp/6-311+g(d,p) scrf=(cpcm,solvent=acetonitrile)

```
# p opt freq td=(nstates=2)/b3lyp/6-311+g(d,p)
scrf=(cpcm,solvent=acetonitrile)
```

Charge = 0, Multiplicity = 1, Point group = C1

Electronic Energy = -592.851069 Hartree

Number of imaginary frequencies = 0

Sum of electronic and zero-point Energies = -592.541796 Hartree

Sum of electronic and thermal Energies = -592.530674 Hartree

Sum of electronic and thermal Enthalpies = -592.529730 Hartree

Sum of electronic and thermal Free Energies = -592.578068 Hartree

	**Cartesian Coordinates**				**Cartesian Coordinates**		
	X	***Y***	***Z***		***X***	***Y***	***Z***
C	0.760708	-0.781252	0.000010	C	0.285533	0.567404	0.000039

C	-1.115434	0.517859	0.000000	C	-1.501929	-0.889643	-0.000027
N	-0.359414	-1.627522	-0.000092	C	2.090780	-1.114105	-0.000072
C	3.025392	-0.011533	0.000074	C	2.606405	1.315401	0.000074
C	1.242323	1.639076	-0.000045	C	-2.147079	1.512648	-0.000030
C	-3.462713	1.076289	0.000019	C	-3.814107	-0.304637	0.000043
C	-2.835682	-1.310826	0.000034	O	4.363167	-0.264193	-0.000474
H	-0.319676	-2.636580	-0.000067	H	2.439680	-2.139877	-0.000531
H	3.361024	2.093245	0.000301	H	0.918177	2.671857	0.000119
H	-1.900752	2.566646	-0.000019	H	-4.265354	1.806150	0.000050
H	-4.862835	-0.577453	0.000062	H	-3.093935	-2.362371	0.000044
H	4.529050	-1.215501	0.003763				

13r* (excited state) b3lyp/6-311+g(d,p) scrf=(cpcm,solvent=acetonitrile)

```
# p opt freq td=(nstates=2)/b3lyp/6-311+g(d,p)
scrf=(cpcm,solvent=acetonitrile)
```

Charge = 0, Multiplicity = 1, Point group = C1

Electronic Energy = -746.530023 Hartree

Number of imaginary frequencies = 0

Sum of electronic and zero-point Energies = -746.195592 Hartree

Sum of electronic and thermal Energies = -746.182226 Hartree

Sum of electronic and thermal Enthalpies = -746.181282 Hartree

Sum of electronic and thermal Free Energies = -746.235251 Hartree

	**Cartesian Coordinates**				**Cartesian Coordinates**		
	X	***Y***	***Z***		***X***	***Y***	***Z***
C	1.892069	0.901824	0.000002	C	1.008465	-0.234487	0.000005
C	-0.315376	0.264750	0.000008	C	-0.213926	1.718530	0.000005
N	1.115359	2.050064	0.000004	C	3.263075	0.792489	0.000000
C	3.804510	-0.515910	-0.000003	C	2.983322	-1.654540	-0.000000
C	1.598274	-1.527858	0.000007	C	-1.621162	-0.362060	0.000004
C	-2.761280	0.526171	-0.000002	C	-2.580988	1.933282	-0.000002
C	-1.316374	2.556873	0.000000	O	5.156907	-0.597289	-0.000019
C	-1.832014	-1.743319	0.000005	C	-3.142946	-2.293013	0.000001
C	-4.239116	-1.442276	-0.000006	C	-4.061955	-0.052225	-0.000008
H	1.475877	2.992335	-0.000002	H	3.920724	1.652055	0.000007
H	3.440233	-2.638020	-0.000006	H	0.991969	-2.421483	0.000013
H	-3.469356	2.556933	-0.000005	H	-1.213612	3.634012	-0.000002
H	5.440429	-1.521195	0.000045	H	-0.990209	-2.420682	0.000010
H	-3.275233	-3.368333	0.000003	H	-5.244627	-1.850373	-0.000011
H	-4.926419	0.603226	-0.000015				

13s* (excited state) b3lyp/6-311+g(d,p) scrf=(cpcm,solvent=acetonitrile)

```
# p opt freq td=(nstates=2)/b3lyp/6-311+g(d,p)
scrf=(cpcm,solvent=acetonitrile)
```

Charge = 0, Multiplicity = 1, Point group = C1

Electronic Energy = -592.851353 Hartree

Number of imaginary frequencies = 0

Sum of electronic and zero-point Energies = -592.545892 Hartree

Sum of electronic and thermal Energies = -592.535084 Hartree

Sum of electronic and thermal Enthalpies = -592.534140 Hartree

Sum of electronic and thermal Free Energies = -592.581736 Hartree

	**Cartesian Coordinates**				**Cartesian Coordinates**		
	X	***Y***	***Z***		***X***	***Y***	***Z***
C	-0.977657	-0.489489	-0.000003	C	-0.352066	0.808074	-0.000004
C	1.063204	0.590234	-0.000004	C	1.273286	-0.821339	-0.000000
N	0.017359	-1.426762	-0.000000	C	-2.361748	-0.666164	-0.000001
C	-3.193121	0.493547	0.000002	C	-2.597402	1.744853	0.000004
C	-1.184674	1.929522	0.000002	C	2.201007	1.438763	-0.000002
C	3.478344	0.830531	-0.000001	C	3.656395	-0.550688	0.000003
C	2.522247	-1.426253	0.000004	O	-2.849857	-1.926509	-0.000006
H	-0.138237	-2.426644	0.000003	H	-4.270305	0.381658	-0.000003
H	-3.231399	2.624027	0.000005	H	-0.778052	2.933317	0.000005
H	2.101341	2.516759	-0.000005	H	4.355556	1.470239	-0.000002
H	4.655295	-0.968957	0.000003	H	2.632594	-2.503399	0.000008
H	-3.816336	-1.917150	0.000036				

13t* (excited state) b3lyp/6-311+g(d,p) scrf=(cpcm,solvent=acetonitrile)

```
# p opt freq td=(nstates=2)/b3lyp/6-311+g(d,p)
scrf=(cpcm,solvent=acetonitrile)
```

Charge = 0, Multiplicity = 1, Point group = C1

Electronic Energy = -900.198153 Hartree

Number of imaginary frequencies = 0

Sum of electronic and zero-point Energies = -899.820583 Hartree

Sum of electronic and thermal Energies = -899.804357 Hartree

Sum of electronic and thermal Enthalpies = -899.803413 Hartree

Sum of electronic and thermal Free Energies = -899.863343 Hartree

	**Cartesian Coordinates**				**Cartesian Coordinates**		
	X	***Y***	***Z***		***X***	***Y***	***Z***
C	1.225829	-1.639533	0.026954	C	0.611991	-0.338725	-0.021184
C	-0.784709	-0.554558	-0.025887	C	-0.987745	-1.989331	-0.133433
N	0.238826	-2.602372	-0.052457	C	2.579966	-1.838601	0.205558

C	3.419294	-0.709804	0.293328	C	2.912035	0.592787	0.116007
C	1.501152	0.818991	-0.108559	C	-1.976957	0.282944	0.104623
C	-3.253539	-0.341305	-0.175807	C	-3.344622	-1.730334	-0.413232
C	-2.223864	-2.579663	-0.342651	O	3.033996	-3.122595	0.301495
C	-1.974053	1.602876	0.561237	C	-3.151643	2.365030	0.651907
C	-4.372596	1.795646	0.276060	C	-4.424888	0.469011	-0.124770
C	3.798688	1.707190	0.079160	C	3.350018	2.974669	-0.246231
C	2.000212	3.172357	-0.559802	C	1.099183	2.101834	-0.488780
H	0.404588	-3.595050	-0.109658	H	4.484197	-0.851257	0.446615
H	-4.323385	-2.159109	-0.600259	H	-2.320167	-3.651527	-0.460248
H	3.992251	-3.133096	0.415043	H	-1.046017	2.055206	0.880000
H	-3.104433	3.388560	1.004075	H	-5.283677	2.382997	0.315846
H	-5.377756	0.016266	-0.379341	H	4.849977	1.538125	0.286476
H	4.045954	3.805169	-0.277440	H	1.643342	4.152433	-0.853439
H	0.068862	2.279749	-0.759419				

11$^{\bullet-}$ (anion radical) b3lyp/6-311+g(d,p) scrf=(cpcm,solvent=acetonitrile)

```
# opt freq b3lyp/6-311+g(d,p)
scrf=(cpcm,solvent=acetonitrile)
```

Charge = -1, Multiplicity = 2, Point group = C1

Electronic Energy = -517.666633 Hartree

Number of imaginary frequencies = 0

Sum of electronic and zero-point Energies = -517.496203 Hartree

Sum of electronic and thermal Energies = -517.486628 Hartree

Sum of electronic and thermal Enthalpies = -517.485684 Hartree

Sum of electronic and thermal Free Energies = -517.531310 Hartree

	**Cartesian Coordinates**				**Cartesian Coordinates**		
	X	***Y***	***Z***		***X***	***Y***	***Z***
C	1.141141	0.861245	-0.000000	C	0.710281	-0.506520	-0.000000
C	-0.710281	-0.506520	-0.000000	C	-1.141141	0.861245	-0.000000
N	0.000000	1.657584	-0.000000	C	2.476514	1.233433	0.000000
C	3.454538	0.194173	0.000000	C	3.059414	-1.148904	0.000000
C	1.710469	-1.527532	-0.000000	C	-1.710469	-1.527532	-0.000000
C	-3.059414	-1.148904	0.000000	C	-3.454538	0.194173	0.000000
C	-2.476514	1.233433	0.000000	H	0.000000	2.664992	0.000000
H	2.769097	2.278462	0.000000	H	4.508619	0.450165	0.000000
H	3.823953	-1.921693	0.000000	H	1.432099	-2.576346	-0.000000
H	-1.432099	-2.576346	-0.000000	H	-3.823953	-1.921693	0.000000
H	-4.508619	0.450165	0.000000	H	-2.769097	2.278462	0.000000

13a•⁻ (anion radical) b3lyp/6-311+g(d,p) scrf=(cpcm,solvent=acetonitrile)

```
# opt freq b3lyp/6-311+g(d,p)
scrf=(cpcm,solvent=acetonitrile)
```

Charge = -1, Multiplicity = 2, Point group = C1

Electronic Energy = -671.357480 Hartree

Number of imaginary frequencies = 0

Sum of electronic and zero-point Energies = -671.139807 Hartree

Sum of electronic and thermal Energies = -671.127712 Hartree

Sum of electronic and thermal Enthalpies = -671.126768 Hartree

Sum of electronic and thermal Free Energies = -671.178333 Hartree

	Cartesian Coordinates				Cartesian Coordinates		
	X	***Y***	***Z***		***X***	***Y***	***Z***
C	-2.313925	0.707645	-0.000001	C	-1.365758	-0.369247	-0.000001
C	-0.057274	0.223785	-0.000001	C	-0.239210	1.639629	-0.000002
N	-1.606991	1.895991	-0.000003	C	-3.689097	0.505601	-0.000000
C	-4.162987	-0.821038	0.000002	C	-3.265766	-1.892313	0.000003
C	-1.878752	-1.687886	0.000002	C	1.282327	-0.317516	-0.000001
C	2.381658	0.634772	0.000002	C	2.125465	2.025292	0.000002
C	0.810219	2.548320	-0.000000	C	1.580414	-1.692690	-0.000004
C	2.904311	-2.168915	-0.000003	C	3.964632	-1.254966	0.000001
C	3.714468	0.111690	0.000004	H	-2.021392	2.814148	-0.000001
H	-4.375754	1.345599	-0.000000	H	-5.231346	-1.008137	0.000003
H	-3.649475	-2.907863	0.000005	H	-1.220055	-2.546224	0.000003
H	2.970696	2.707028	0.000004	H	0.629870	3.617819	-0.000001
H	0.771221	-2.411176	-0.000008	H	3.097009	-3.236521	-0.000005
H	4.990161	-1.613502	0.000003	H	4.543663	0.813919	0.000007

13b•⁻ (anion radical) b3lyp/6-311+g(d,p) scrf=(cpcm,solvent=acetonitrile)

```
# opt freq b3lyp/6-311+g(d,p)
scrf=(cpcm,solvent=acetonitrile)
```

Charge = -1, Multiplicity = 2, Point group = C1

Electronic Energy = -671.350671 Hartree

Number of imaginary frequencies = 0

Sum of electronic and zero-point Energies = -671.134649 Hartree

Sum of electronic and thermal Energies = -671.122129 Hartree

Sum of electronic and thermal Enthalpies = -671.121185 Hartree

Sum of electronic and thermal Free Energies = -671.173850 Hartree

	Cartesian Coordinates				Cartesian Coordinates		
	X	***Y***	***Z***		***X***	***Y***	***Z***
C	1.995105	-0.889086	0.000000	C	1.921611	0.540450	0.000000

C	0.528095	0.885178	0.000000	C	-0.220619	-0.333889	0.000000
N	0.703797	-1.389756	-0.000000	C	3.211147	-1.567613	-0.000000
C	4.395279	-0.805658	-0.000000	C	4.346095	0.593258	-0.000000
C	3.123222	1.275465	0.000000	C	-0.183228	2.102514	0.000000
C	-1.563454	2.090199	0.000000	C	-2.327373	0.865893	0.000000
C	-1.616741	-0.399167	0.000000	C	-3.732167	0.860337	-0.000000
C	-4.468868	-0.353352	-0.000000	C	-3.784227	-1.569383	-0.000000
C	-2.386920	-1.617833	0.000000	H	0.472247	-2.369149	0.000000
H	3.246516	-2.651849	-0.000000	H	5.354333	-1.312007	-0.000000
H	5.272187	1.159063	-0.000000	H	3.104639	2.360404	0.000000
H	0.348922	3.049493	0.000000	H	-2.109756	3.028929	0.000000
H	-4.256947	1.811648	-0.000000	H	-5.553440	-0.333427	-0.000000
H	-4.344969	-2.500459	-0.000000	H	-1.882039	-2.578233	0.000000

13c•− (anion radical) b3lyp/6-311+g(d,p) scrf=(cpcm,solvent=acetonitrile)

```
# opt freq b3lyp/6-311+g(d,p)
scrf=(cpcm,solvent=acetonitrile)
```

Charge = -1, Multiplicity = 2, Point group = C1

Electronic Energy = -671.360223 Hartree

Number of imaginary frequencies = 0

Sum of electronic and zero-point Energies = -671.142939 Hartree

Sum of electronic and thermal Energies = -671.130776 Hartree

Sum of electronic and thermal Enthalpies = -671.129832 Hartree

Sum of electronic and thermal Free Energies = -671.181486 Hartree

	Cartesian Coordinates				Cartesian Coordinates		
	X	***Y***	***Z***		***X***	***Y***	***Z***
C	2.441723	-0.817204	-0.000000	C	1.859524	0.493571	-0.000000
C	0.438643	0.329853	-0.000000	C	0.180004	-1.073942	-0.000000
N	1.402117	-1.738176	-0.000000	C	3.812371	-1.023911	0.000000
C	4.653911	0.107411	0.000000	C	4.108596	1.401158	0.000000
C	2.727804	1.609216	-0.000000	C	-0.662000	1.224660	-0.000000
C	-1.985490	0.725288	-0.000000	C	-2.221053	-0.711589	0.000000
C	-1.100191	-1.605737	0.000000	C	-3.128624	1.580974	-0.000000
C	-4.425874	1.078255	0.000000	C	-4.647786	-0.306271	0.000000
C	-3.552893	-1.178780	0.000000	H	1.515429	-2.739148	0.000000
H	4.226982	-2.026801	0.000000	H	5.730014	-0.026340	0.000000
H	4.775958	2.257564	0.000000	H	2.327654	2.618101	-0.000000
H	-0.501107	2.298647	-0.000000	H	-1.265413	-2.678524	0.000000
H	-2.966681	2.655706	-0.000000	H	-5.268501	1.763486	0.000000
H	-5.658780	-0.700511	0.000000	H	-3.722367	-2.252659	0.000000

13d$^{\bullet-}$ (anion radical) b3lyp/6-311+g(d,p) scrf=(cpcm,solvent=acetonitrile)

```
# opt freq b3lyp/6-311+g(d,p)
scrf=(cpcm,solvent=acetonitrile)
```

Charge = -1, Multiplicity = 2, Point group = C1

Electronic Energy = -788.095826 Hartree

Number of imaginary frequencies = 0

Sum of electronic and zero-point Energies = -787.817890 Hartree

Sum of electronic and thermal Energies = -787.801873 Hartree

Sum of electronic and thermal Enthalpies = -787.800929 Hartree

Sum of electronic and thermal Free Energies = -787.863191 Hartree

	**Cartesian Coordinates**				**Cartesian Coordinates**		
	X	***Y***	***Z***		***X***	***Y***	***Z***
C	-0.943929	1.142980	-0.043853	C	-2.307285	0.703991	-0.028874
C	-2.303754	-0.712099	0.031040	C	-0.938243	-1.144324	0.043809
N	-0.125563	0.001319	-0.000669	C	-0.586996	2.479814	-0.120394
C	-1.634988	3.447236	-0.179007	C	-2.974435	3.041820	-0.160393
C	-3.338055	1.691814	-0.087817	C	-3.329488	-1.705077	0.091482
C	-2.958997	-3.053275	0.163050	C	-1.617523	-3.452039	0.179366
C	-0.574459	-2.479368	0.119352	C	1.294470	0.004098	-0.001187
C	2.006141	-0.647770	-1.013339	C	3.399123	-0.647013	-1.006526
C	4.122012	0.011915	-0.004638	C	3.397998	0.671088	0.995889
C	2.004783	0.662539	1.007227	C	5.631582	-0.010444	0.010233
H	0.451762	2.787491	-0.135868	H	-1.386088	4.501324	-0.235787
H	-3.754516	3.797443	-0.204455	H	-4.383331	1.400952	-0.080944
H	-4.376218	-1.419456	0.086357	H	-3.735237	-3.812783	0.208172
H	-1.363299	-4.504893	0.235437	H	0.465875	-2.781769	0.133030
H	1.465171	-1.149032	-1.807172	H	3.932191	-1.157500	-1.802402
H	3.929975	1.193221	1.784852	H	1.463133	1.167741	1.798088
H	6.038546	-0.064382	-1.002129	H	6.037994	0.879268	0.496277
H	6.005247	-0.882184	0.558696				

13e$^{\bullet-}$ (anion radical) b3lyp/6-311+g(d,p) scrf=(cpcm,solvent=acetonitrile)

```
# opt freq b3lyp/6-311+g(d,p)
scrf=(cpcm,solvent=acetonitrile)
```

Charge = -1, Multiplicity = 2, Point group = C1

Electronic Energy = -941.786323 Hartree

Number of imaginary frequencies = 0

Sum of electronic and zero-point Energies = -941.461451 Hartree

Sum of electronic and thermal Energies = -941.442752 Hartree

Sum of electronic and thermal Enthalpies = -941.441808 Hartree

Sum of electronic and thermal Free Energies = -941.510517 Hartree

	Cartesian Coordinates				Cartesian Coordinates		
	X	*Y*	*Z*		*X*	*Y*	*Z*
C	-0.369638	1.575167	0.040634	C	1.063452	1.590563	0.047441
C	1.502260	0.226210	0.009195	C	0.333873	-0.592075	-0.018988
N	-0.793119	0.246469	0.002902	C	-1.134798	2.734069	0.094225
C	-0.464357	3.972313	0.143963	C	0.931398	4.020630	0.141509
C	1.702832	2.851896	0.095748	C	2.806430	-0.395772	-0.017811
C	2.847661	-1.847086	-0.083121	C	1.648097	-2.594413	-0.119400
C	0.374959	-1.977500	-0.088991	C	4.022883	0.311816	0.013689
C	5.267542	-0.342844	-0.015358	C	5.309780	-1.740463	-0.077700
C	4.131965	-2.477221	-0.110685	C	-2.146424	-0.189453	-0.003821
C	-2.999390	0.160248	-1.053946	C	-4.326989	-0.263517	-1.051202
C	-4.835175	-1.055628	-0.015214	C	-3.965831	-1.409365	1.024351
C	-2.641621	-0.978051	1.038783	C	-6.280035	-1.493013	-0.003826
H	-2.217298	2.689865	0.095988	H	-1.040371	4.890317	0.183515
H	1.432309	4.982997	0.177679	H	2.781321	2.933479	0.101863
H	1.713553	-3.676969	-0.171621	H	-0.531172	-2.569825	-0.117773
H	4.008500	1.392330	0.063553	H	6.184866	0.235567	0.010874
H	6.266459	-2.254454	-0.100620	H	4.172613	-3.561863	-0.159604
H	-2.619994	0.759154	-1.873596	H	-4.973818	0.017615	-1.876082
H	-4.330713	-2.024830	1.840458	H	-1.986831	-1.251716	1.857647
H	-6.689379	-1.539130	-1.015287	H	-6.394691	-2.476259	0.458234
H	-6.896765	-0.790633	0.567615				

13f•⁻ (anion radical) b3lyp/6-311+g(d,p) scrf=(cpcm,solvent=acetonitrile)

```
# opt freq b3lyp/6-311+g(d,p)
scrf=(cpcm,solvent=acetonitrile)
```

Charge = -1, Multiplicity = 2, Point group = C1

Electronic Energy = -941.775430 Hartree

Number of imaginary frequencies = 0

Sum of electronic and zero-point Energies = -941.451418 Hartree

Sum of electronic and thermal Energies = -941.432719 Hartree

Sum of electronic and thermal Enthalpies = -941.431774 Hartree

Sum of electronic and thermal Free Energies = -941.499990 Hartree

	Cartesian Coordinates				Cartesian Coordinates		
	X	*Y*	*Z*		*X*	*Y*	*Z*
C	0.150542	2.025063	-0.182973	C	1.545409	2.190591	0.061920
C	2.113538	0.881632	0.160500	C	1.067178	-0.075675	-0.042520

N	-0.138810	0.650370	-0.268218	C	-0.709680	3.116318	-0.265420
C	-0.165332	4.407897	-0.140376	C	1.206808	4.589056	0.069754
C	2.067707	3.494096	0.175418	C	3.427585	0.430296	0.380509
C	3.693256	-0.921984	0.376612	C	2.673035	-1.900317	0.098958
C	1.309037	-1.459927	-0.149598	C	2.979972	-3.271115	0.035490
C	2.002576	-4.237495	-0.289430	C	0.703352	-3.816762	-0.568165
C	0.345745	-2.466040	-0.508927	C	-1.461450	0.156558	-0.094468
C	-2.448507	0.431053	-1.048962	C	-3.750918	-0.023922	-0.868980
C	-4.110709	-0.776901	0.260287	C	-3.115415	-1.048834	1.203376
C	-1.809220	-0.586811	1.039735	C	-5.523966	-1.277695	0.437466
H	-1.772193	2.984618	-0.426792	H	-0.820859	5.269051	-0.208519
H	1.606394	5.594274	0.157285	H	3.127408	3.647304	0.351323
H	4.229708	1.143129	0.548661	H	4.704535	-1.277192	0.550773
H	4.003419	-3.579959	0.229281	H	2.264277	-5.289453	-0.331626
H	-0.053092	-4.548550	-0.839030	H	-0.668980	-2.180474	-0.743220
H	-2.186710	0.989690	-1.940155	H	-4.497872	0.197901	-1.624897
H	-3.362828	-1.627989	2.087608	H	-1.054812	-0.804838	1.786267
H	-5.806288	-1.964414	-0.367239	H	-5.640533	-1.807381	1.385020
H	-6.243171	-0.452793	0.421547				

13g•– (anion radical) b3lyp/6-311+g(d,p) scrf=(cpcm,solvent=acetonitrile)

```
# opt freq b3lyp/6-311+g(d,p)
scrf=(cpcm,solvent=acetonitrile)
```

Charge = -1, Multiplicity = 2, Point group = C1

Electronic Energy = -941.789231 Hartree

Number of imaginary frequencies = 0

Sum of electronic and zero-point Energies = -941.464490 Hartree

Sum of electronic and thermal Energies = -941.445846 Hartree

Sum of electronic and thermal Enthalpies = -941.444902 Hartree

Sum of electronic and thermal Free Energies = -941.513140 Hartree

	Cartesian Coordinates				Cartesian Coordinates		
	X	***Y***	***Z***		***X***	***Y***	***Z***
C	-0.815326	1.864050	0.033788	C	0.531175	2.349904	0.042945
C	1.398749	1.216603	0.023486	C	0.573876	0.054092	0.005965
N	-0.769629	0.463190	0.012270	C	-1.909453	2.714530	0.074806
C	-1.671441	4.104271	0.111186	C	-0.360321	4.603693	0.110022
C	0.741411	3.747063	0.078398	C	2.807469	1.058592	0.004822
C	3.378392	-0.234565	-0.031617	C	2.515977	-1.405324	-0.056131
C	1.091806	-1.230845	-0.038539	C	4.789148	-0.452842	-0.049141
C	5.335361	-1.731795	-0.089071	C	4.499994	-2.857753	-0.113772

C	3.110685	-2.684111	-0.096940	C	-1.899606	-0.398552	0.004715
C	-2.839074	-0.327005	-1.027940	C	-3.949939	-1.168196	-1.026484
C	-4.147842	-2.110609	-0.010645	C	-3.191994	-2.180744	1.010464
C	-2.087125	-1.333036	1.027870	C	-5.364348	-3.004590	-0.001212
H	-2.921988	2.328460	0.077270	H	-2.512160	4.788384	0.139620
H	-0.200426	5.677137	0.136671	H	1.748695	4.150688	0.086023
H	3.459618	1.926726	0.014229	H	0.447919	-2.102212	-0.057563
H	5.444665	0.413738	-0.030984	H	6.414167	-1.855859	-0.101499
H	4.924211	-3.855891	-0.145177	H	2.460412	-3.554989	-0.115954
H	-2.693811	0.382188	-1.834271	H	-4.667309	-1.097853	-1.837824
H	-3.316534	-2.903282	1.810720	H	-1.365860	-1.393713	1.834139
H	-5.748203	-3.164264	-1.011176	H	-5.138956	-3.978952	0.438329
H	-6.172491	-2.559632	0.589712				

13h$^{\bullet-}$ (anion radical) b3lyp/6-311+g(d,p) scrf=(cpcm,solvent=acetonitrile)

```
# opt freq b3lyp/6-311+g(d,p)
scrf=(cpcm,solvent=acetonitrile)
```

Charge = -1, Multiplicity = 2, Point group = C1

Electronic Energy = -592.917229 Hartree

Number of imaginary frequencies = 0

Sum of electronic and zero-point Energies = -592.743056 Hartree

Sum of electronic and thermal Energies = -592.731958 Hartree

Sum of electronic and thermal Enthalpies = -592.731014 Hartree

Sum of electronic and thermal Free Energies = -592.779963 Hartree

	Cartesian Coordinates				Cartesian Coordinates		
	X	*Y*	*Z*		*X*	*Y*	*Z*
C	0.636401	1.160848	0.000003	C	0.417527	-0.254418	0.000008
C	-0.985213	-0.470905	0.000006	C	-1.619518	0.817432	0.000002
N	-0.615828	1.777420	0.000003	C	1.901707	1.720921	-0.000005
C	3.028745	0.841181	0.000015	C	2.828210	-0.541122	-0.000005
C	1.556868	-1.120926	-0.000025	C	-1.817398	-1.630779	0.000003
C	-3.208348	-1.464529	0.000000	C	-3.803292	-0.196883	-0.000002
C	-2.996748	0.977899	-0.000002	O	3.971801	-1.337978	-0.000054
H	-0.769425	2.772690	-0.000003	H	2.043919	2.796312	-0.000003
H	4.038683	1.233935	0.000058	H	1.443885	-2.201052	-0.000094
H	-1.381675	-2.624682	0.000005	H	-3.845375	-2.345130	-0.000000
H	-4.884058	-0.104546	-0.000004	H	-3.446469	1.965554	-0.000005
H	3.703257	-2.263508	0.000454				

13i$^{\bullet-}$ (anion radical) b3lyp/6-311+g(d,p) scrf=(cpcm,solvent=acetonitrile)

```
# opt freq b3lyp/6-311+g(d,p)
scrf=(cpcm,solvent=acetonitrile)
```

Charge = -1, Multiplicity = 2, Point group = C1

Electronic Energy = -746.606743 Hartree

Number of imaginary frequencies = 0

Sum of electronic and zero-point Energies = -746.385718 Hartree

Sum of electronic and thermal Energies = -746.372027 Hartree

Sum of electronic and thermal Enthalpies = -746.371082 Hartree

Sum of electronic and thermal Free Energies = -746.426129 Hartree

	Cartesian Coordinates				Cartesian Coordinates		
	X	***Y***	***Z***		***X***	***Y***	***Z***
C	-1.888264	-1.258253	0.000009	C	-1.131950	-0.040375	0.000003
C	0.254014	-0.404465	0.000003	C	0.312473	-1.832694	0.000006
N	-0.989711	-2.315726	0.000014	C	-3.274469	-1.276931	0.000008
C	-3.965902	-0.046349	0.000010	C	-3.249183	1.149982	-0.000009
C	-1.850814	1.181246	-0.000023	C	1.485341	0.353464	0.000004
C	2.728737	-0.400997	-0.000010	C	2.710093	-1.814605	-0.000014
C	1.501597	-2.550162	-0.000004	C	1.549934	1.758818	0.000021
C	2.773853	2.449828	0.000020	C	3.973234	1.725720	-0.000001
C	3.954741	0.337886	-0.000016	O	-3.989770	2.324990	-0.000050
H	-1.243238	-3.290377	0.000011	H	-3.821846	-2.213308	0.000017
H	-5.049103	-0.019244	0.000030	H	-1.345581	2.139094	-0.000066
H	3.657826	-2.344547	-0.000024	H	1.503954	-3.634847	-0.000004
H	0.632819	2.333134	0.000041	H	2.784841	3.534528	0.000035
H	4.924198	2.250813	-0.000005	H	4.889724	-0.215712	-0.000031
H	-3.388070	3.077952	0.000255				

13j$^{\bullet-}$ (anion radical) b3lyp/6-311+g(d,p) scrf=(cpcm,solvent=acetonitrile)

```
# opt freq b3lyp/6-311+g(d,p)
scrf=(cpcm,solvent=acetonitrile)
```

Charge = -1, Multiplicity = 2, Point group = C1

Electronic Energy = -746.599905 Hartree

Number of imaginary frequencies = 0

Sum of electronic and zero-point Energies = -746.380211 Hartree

Sum of electronic and thermal Energies = -746.366987 Hartree

Sum of electronic and thermal Enthalpies = -746.366043 Hartree

Sum of electronic and thermal Free Energies = -746.419882 Hartree

	Cartesian Coordinates				Cartesian Coordinates		
	X	***Y***	***Z***		***X***	***Y***	***Z***

C	1.570462	-1.076361	-0.000002	C	1.573094	0.356286	-0.000001
C	0.208124	0.773844	-0.000003	C	-0.612777	-0.399935	-0.000001
N	0.245809	-1.504239	-0.000002	C	2.745648	-1.812127	-0.000003
C	3.978927	-1.113707	-0.000016	C	3.990641	0.283486	0.000002
C	2.813608	1.035245	0.000017	C	-0.435459	2.036246	-0.000006
C	-1.808099	2.109478	-0.000006	C	-2.644101	0.931452	-0.000001
C	-2.008555	-0.375206	0.000001	C	-4.043015	1.011896	0.000001
C	-4.853711	-0.146420	0.000004	C	-4.240391	-1.408029	0.000005
C	-2.857310	-1.542026	0.000004	O	5.184680	0.994453	-0.000030
H	-0.041903	-2.468281	0.000001	H	2.732435	-2.896546	-0.000004
H	4.913728	-1.664868	-0.000049	H	2.869949	2.117961	0.000046
H	0.153796	2.948916	-0.000009	H	-2.297102	3.079261	-0.000010
H	-4.506378	1.994866	-0.000001	H	-5.934543	-0.060229	0.000005
H	-4.857910	-2.302315	0.000008	H	-2.414148	-2.532694	0.000005
H	5.921463	0.373252	0.000305				

13k$^{\bullet-}$ (anion radical) b3lyp/6-311+g(d,p) scrf=(cpcm,solvent=acetonitrile)

```
# opt freq b3lyp/6-311+g(d,p)
scrf=(cpcm,solvent=acetonitrile)
```

Charge = -1, Multiplicity = 2, Point group = C1

Electronic Energy = -746.609652 Hartree

Number of imaginary frequencies = 0

Sum of electronic and zero-point Energies = -746.388795 Hartree

Sum of electronic and thermal Energies = -746.375101 Hartree

Sum of electronic and thermal Enthalpies = -746.374157 Hartree

Sum of electronic and thermal Free Energies = -746.429153 Hartree

	Cartesian Coordinates				Cartesian Coordinates		
	X	***Y***	***Z***		***X***	***Y***	***Z***
C	-2.012242	1.187983	-0.000001	C	-1.556840	-0.169257	-0.000003
C	-0.129866	-0.142681	-0.000003	C	0.263557	1.232812	-0.000002
N	-0.886552	2.010370	-0.000002	C	-3.357050	1.512481	0.000002
C	-4.304043	0.465344	-0.000001	C	-3.872739	-0.867462	0.000002
C	-2.521161	-1.207301	0.000006	C	0.878218	-1.139559	-0.000002
C	2.243686	-0.771106	-0.000001	C	2.617292	0.635721	-0.000000
C	1.589322	1.634941	-0.000001	C	3.297942	-1.733790	0.000000
C	4.636832	-1.359975	0.000002	C	4.992232	-0.002592	0.000002
C	3.988365	0.971281	0.000001	O	-4.862906	-1.841477	0.000018
H	-0.903783	3.017350	0.000004	H	-3.686317	2.546174	0.000002
H	-5.365974	0.680036	-0.000010	H	-2.219251	-2.250603	0.000016
H	0.613604	-2.192831	-0.000003	H	1.859328	2.686372	0.000000

H	3.032032	-2.787638	0.000000	H	5.409098	-2.123560	0.000003
H	6.036777	0.291300	0.000003	H	4.261439	2.023569	0.000001
H	-4.448872	-2.711984	-0.000142				

13l$^{\bullet-}$ (anion radical) b3lyp/6-311+g(d,p) scrf=(cpcm,solvent=acetonitrile)

```
# opt freq b3lyp/6-311+g(d,p)
scrf=(cpcm,solvent=acetonitrile)
```

Charge = -1, Multiplicity = 2, Point group = C1

Electronic Energy = -863.346625 Hartree

Number of imaginary frequencies = 0

Sum of electronic and zero-point Energies = -863.064932 Hartree

Sum of electronic and thermal Energies = -863.047442 Hartree

Sum of electronic and thermal Enthalpies = -863.046498 Hartree

Sum of electronic and thermal Free Energies = -863.111885 Hartree

	**Cartesian Coordinates**				**Cartesian Coordinates**		
	X	***Y***	***Z***		***X***	***Y***	***Z***
C	0.888407	-0.755659	-0.022726	C	2.131618	-0.049015	0.000243
C	1.841545	1.336179	0.044901	C	0.414988	1.483357	0.046916
N	-0.149907	0.198927	0.006098	C	0.822460	-2.135474	-0.098759
C	2.047117	-2.871505	-0.140336	C	3.266381	-2.191294	-0.107857
C	3.349396	-0.799002	-0.040248	C	2.645170	2.514213	0.097298
C	2.013453	3.761566	0.156792	C	0.618816	3.881621	0.169227
C	-0.206079	2.720638	0.114423	C	-1.537361	-0.093745	-0.000306
C	-2.375229	0.444619	-0.982883	C	-3.738748	0.158812	-0.980390
C	-4.305830	-0.680853	-0.014442	C	-3.455064	-1.224537	0.955590
C	-2.093427	-0.931387	0.972799	C	-5.787809	-0.969295	-0.002979
O	4.418595	-2.971847	-0.148714	H	-0.125447	-2.658568	-0.124827
H	2.039928	-3.953625	-0.193357	H	4.315567	-0.303518	-0.026521
H	3.727974	2.443829	0.096379	H	2.621475	4.661418	0.196444
H	0.157355	4.861908	0.217457	H	-1.285664	2.810649	0.123856
H	-1.954656	1.081506	-1.751818	H	-4.369431	0.586610	-1.753348
H	-3.863465	-1.881336	1.717224	H	-1.456249	-1.351645	1.741683
H	-6.212481	-0.908963	-1.007609	H	-5.996782	-1.963645	0.398203
H	-6.323540	-0.246246	0.622035	H	5.187171	-2.391521	-0.112364

13m$^{\bullet-}$ (anion radical) b3lyp/6-311+g(d,p) scrf=(cpcm,solvent=acetonitrile)

```
# opt freq b3lyp/6-311+g(d,p)
scrf=(cpcm,solvent=acetonitrile)
```

Charge = -1, Multiplicity = 2, Point group = C1

Electronic Energy = -1170.707678 Hartree

Number of imaginary frequencies = 0

Sum of electronic and zero-point Energies = -1170.332687 Hartree

Sum of electronic and thermal Energies = -1170.309703 Hartree

Sum of electronic and thermal Enthalpies = -1170.308759 Hartree

Sum of electronic and thermal Free Energies = -1170.386869 Hartree

	**Cartesian Coordinates**				**Cartesian Coordinates**		
	X	***Y***	***Z***		***X***	***Y***	***Z***
C	-0.486550	1.818649	-0.300342	C	0.931981	1.855013	-0.128707
C	1.380458	0.499043	-0.056049	C	0.241441	-0.348679	-0.211615
N	-0.900620	0.474735	-0.364083	C	-1.257935	2.968826	-0.354560
C	-0.617691	4.224569	-0.273053	C	0.767417	4.281574	-0.138974
C	1.554531	3.128269	-0.064041	C	2.683860	-0.081953	0.141931
C	2.813425	-1.513375	0.127649	C	1.641748	-2.356435	-0.150327
C	0.331405	-1.744080	-0.331403	C	1.744983	-3.750848	-0.270056
C	0.658078	-4.570528	-0.569733	C	-0.603831	-3.972764	-0.785074
C	-0.764268	-2.611119	-0.671926	C	-2.247835	0.109457	-0.071698
C	-3.262829	0.365706	-0.996410	C	-4.585073	0.038519	-0.699640
C	-4.930850	-0.557791	0.518673	C	-3.902417	-0.813612	1.435130
C	-2.581064	-0.478435	1.153194	C	-6.366335	-0.891985	0.845962
O	1.343309	5.542097	-0.079593	C	3.844170	0.695346	0.375447
C	5.090048	0.115452	0.598591	C	5.214273	-1.273569	0.602316
C	4.089400	-2.063621	0.372652	H	-2.333911	2.917614	-0.464061
H	-1.191088	5.142658	-0.317308	H	2.625961	3.239126	0.039403
H	2.710275	-4.225206	-0.140501	H	0.786996	-5.643133	-0.656433
H	-1.456891	-4.589500	-1.052096	H	-1.739054	-2.183611	-0.859060
H	-3.012015	0.806654	-1.954248	H	-5.357719	0.239827	-1.434785
H	-4.138716	-1.277913	2.387506	H	-1.799009	-0.682757	1.875347
H	-6.955899	-1.043930	-0.060802	H	-6.436087	-1.796038	1.455494
H	-6.838566	-0.081148	1.411725	H	2.296626	5.449941	0.028738
H	3.767287	1.771955	0.394826	H	5.954922	0.746484	0.774866
H	6.175590	-1.742129	0.783587	H	4.215924	-3.138578	0.385501

13n$^{\bullet-}$ (anion radical) b3lyp/6-311+g(d,p) scrf=(cpcm,solvent=acetonitrile)

```
# opt freq b3lyp/6-311+g(d,p)
scrf=(cpcm,solvent=acetonitrile)
```

Charge = -1, Multiplicity = 2, Point group = C1

Electronic Energy = -1092.280204 Hartree

Number of imaginary frequencies = 0

Sum of electronic and zero-point Energies = -1091.947697 Hartree

Sum of electronic and thermal Energies = -1091.926180 Hartree

Sum of electronic and thermal Enthalpies = -1091.925236 Hartree

Sum of electronic and thermal Free Energies = -1091.999630 Hartree

	Cartesian Coordinates				Cartesian Coordinates		
	X	*Y*	*Z*		*X*	*Y*	*Z*
C	-0.877469	1.631745	-0.112460	C	0.440407	2.163345	0.013053
C	1.354202	1.072868	0.021407	C	0.580852	-0.127915	-0.104450
N	-0.779104	0.226925	-0.201048	C	-1.999065	2.440571	-0.111291
C	-1.823431	3.840009	-0.005871	C	-0.533844	4.375949	0.095959
C	0.602482	3.569653	0.108322	C	2.763001	0.992757	0.094614
C	3.400647	-0.263314	0.049448	C	2.613649	-1.475650	-0.087714
C	1.186049	-1.371203	-0.179735	C	4.820646	-0.409795	0.126046
C	5.434172	-1.655183	0.073223	C	4.665780	-2.821040	-0.059380
C	3.271048	-2.723478	-0.139634	C	-1.897703	-0.644123	-0.079615
C	-2.865068	-0.692027	-1.090825	C	-3.970359	-1.526997	-0.964852
C	-4.139335	-2.350138	0.157654	C	-3.167784	-2.293815	1.161369
C	-2.065798	-1.443780	1.056771	C	-5.321897	-3.281723	0.263542
O	-0.439658	5.757152	0.187698	O	0.501987	-2.566797	-0.379628
H	-2.996277	2.025053	-0.192495	H	-2.678362	4.505196	-0.006089
H	1.589381	4.013526	0.197968	H	3.362517	1.893308	0.182294
H	5.424285	0.487694	0.227734	H	6.516321	-1.723420	0.134997
H	5.145581	-3.793169	-0.100023	H	2.673260	-3.620931	-0.243117
H	-2.740317	-0.077789	-1.974733	H	-4.709013	-1.549437	-1.759611
H	-3.276494	-2.910132	2.047597	H	-1.339408	-1.391846	1.860213
H	-6.240162	-2.798221	-0.078881	H	-5.173959	-4.172530	-0.356268
H	-5.472993	-3.616150	1.291681	H	0.489053	6.005963	0.256969
H	-0.445401	-2.428733	-0.258943				

13o•⁻ (anion radical) b3lyp/6-311+g(d,p) scrf=(cpcm,solvent=acetonitrile)

```
# opt freq b3lyp/6-311+g(d,p)
scrf=(cpcm,solvent=acetonitrile)
```

Charge = -1, Multiplicity = 2, Point group = C1

Electronic Energy = -592.912173 Hartree

Number of imaginary frequencies = 0

Sum of electronic and zero-point Energies = -592.738592 Hartree

Sum of electronic and thermal Energies = -592.728015 Hartree

Sum of electronic and thermal Enthalpies = -592.727071 Hartree

Sum of electronic and thermal Free Energies = -592.774989 Hartree

	Cartesian Coordinates				Cartesian Coordinates		
	X	*Y*	*Z*		*X*	*Y*	*Z*
C	-0.938809	-1.185346	0.000065	C	-0.625520	0.216745	-0.000008

C	0.793137	0.352710	-0.000121	C	1.338107	-0.974310	0.000150
N	0.272461	-1.868008	0.000231	C	-2.232206	-1.682463	-0.000144
C	-3.299774	-0.751578	-0.000369	C	-3.030654	0.631781	-0.000147
C	-1.729730	1.113976	0.000153	C	1.720758	1.452212	-0.000570
C	3.097343	1.175930	-0.000351	C	3.598164	-0.126669	0.000077
C	2.699246	-1.244141	0.000263	O	-1.543150	2.495085	0.000326
H	0.365276	-2.871042	0.000306	H	-2.419790	-2.751064	-0.000168
H	-4.325964	-1.099907	-0.000560	H	-3.847319	1.347362	-0.000089
H	1.391118	2.486686	-0.001227	H	3.796125	2.008715	-0.000628
H	4.669184	-0.298198	0.000190	H	3.068423	-2.264249	0.000477
H	-0.599459	2.683999	0.003489				

13p$^{\bullet-}$ (anion radical) b3lyp/6-311+g(d,p) scrf=(cpcm,solvent=acetonitrile)

```
# opt freq b3lyp/6-311+g(d,p)
scrf=(cpcm,solvent=acetonitrile)
```

Charge = -1, Multiplicity = 2, Point group = C1

Electronic Energy = -746.600548 Hartree

Number of imaginary frequencies = 0

Sum of electronic and zero-point Energies = -746.378801 Hartree

Sum of electronic and thermal Energies = -746.365574 Hartree

Sum of electronic and thermal Enthalpies = -746.364630 Hartree

Sum of electronic and thermal Free Energies = -746.418385 Hartree

	**Cartesian Coordinates**				**Cartesian Coordinates**		
	X	***Y***	***Z***		***X***	***Y***	***Z***
C	2.210390	0.967018	-0.135260	C	1.315886	-0.144879	-0.026441
C	-0.030639	0.387725	-0.036746	C	0.096662	1.808125	-0.043014
N	1.447976	2.120121	-0.160915	C	3.596679	0.839330	-0.177734
C	4.131991	-0.450030	-0.070718	C	3.296544	-1.556854	0.115810
C	1.908968	-1.410046	0.177464	C	-1.349995	-0.197329	-0.101359
C	-2.472879	0.697637	0.141235	C	-2.263152	2.088825	0.243879
C	-0.981248	2.673408	0.092679	O	1.182734	-2.536341	0.506674
C	-1.624878	-1.539088	-0.432074	C	-2.934263	-2.071303	-0.386033
C	-3.997804	-1.237765	-0.032126	C	-3.782531	0.115388	0.214882
H	1.825697	3.053280	-0.124200	H	4.235487	1.709991	-0.272903
H	5.206323	-0.592404	-0.101901	H	3.716081	-2.546642	0.258931
H	-3.126706	2.730923	0.390363	H	-0.844660	3.748817	0.107877
H	0.239435	-2.320167	0.542407	H	-0.834498	-2.167856	-0.827397
H	-3.105068	-3.112477	-0.636947	H	-5.005604	-1.639435	0.025744
H	-4.622573	0.764890	0.444375				

13q$^{•−}$ (anion radical) b3lyp/6-311+g(d,p) scrf=(cpcm,solvent=acetonitrile)

```
# opt freq b3lyp/6-311+g(d,p)
scrf=(cpcm,solvent=acetonitrile)
```

Charge = -1, Multiplicity = 2, Point group = C1

Electronic Energy = -592.912800 Hartree

Number of imaginary frequencies = 0

Sum of electronic and zero-point Energies = -592.739361 Hartree

Sum of electronic and thermal Energies = -592.728792 Hartree

Sum of electronic and thermal Enthalpies = -592.727848 Hartree

Sum of electronic and thermal Free Energies = -592.775793 Hartree

	Cartesian Coordinates				Cartesian Coordinates		
	X	***Y***	***Z***		***X***	***Y***	***Z***
C	0.766284	-0.770312	-0.000001	C	0.282483	0.575183	0.000008
C	-1.139610	0.510518	0.000011	C	-1.509657	-0.872503	0.000001
N	-0.331913	-1.617550	0.000001	C	2.118934	-1.094337	-0.000057
C	3.039203	-0.010065	-0.000009	C	2.612789	1.309498	0.000022
C	1.237873	1.634502	-0.000012	C	-2.183927	1.487170	0.000010
C	-3.513716	1.048404	0.000007	C	-3.849681	-0.311799	0.000001
C	-2.825906	-1.306081	-0.000002	O	4.414544	-0.255927	-0.000134
H	-0.284567	-2.623862	-0.000021	H	2.461771	-2.124241	-0.000167
H	3.360613	2.096720	0.000080	H	0.923497	2.672067	-0.000001
H	-1.952462	2.547357	0.000017	H	-4.312042	1.786352	0.000008
H	-4.891386	-0.614013	-0.000003	H	-3.070232	-2.363458	-0.000008
H	4.561432	-1.207720	0.001292				

13r$^{•−}$ (anion radical) b3lyp/6-311+g(d,p) scrf=(cpcm,solvent=acetonitrile)

```
# opt freq b3lyp/6-311+g(d,p)
scrf=(cpcm,solvent=acetonitrile)
```

Charge = -1, Multiplicity = 2, Point group = C1

Electronic Energy = -746.606025 Hartree

Number of imaginary frequencies = 0

Sum of electronic and zero-point Energies = -746.384725 Hartree

Sum of electronic and thermal Energies = -746.371226 Hartree

Sum of electronic and thermal Enthalpies = -746.370281 Hartree

Sum of electronic and thermal Free Energies = -746.424756 Hartree

	Cartesian Coordinates				Cartesian Coordinates		
	X	***Y***	***Z***		***X***	***Y***	***Z***
C	1.891971	0.897509	-0.000003	C	1.015555	-0.236740	0.000002
C	-0.331772	0.276508	0.000005	C	-0.235903	1.698281	0.000003
N	1.116545	2.038070	0.000000	C	3.279175	0.789565	0.000011

C	3.827652	-0.502155	-0.000001	C	3.014284	-1.633298	-0.000015
C	1.612610	-1.512817	0.000004	C	-1.631046	-0.347900	0.000002
C	-2.788323	0.533499	-0.000000	C	-2.617989	1.937327	-0.000001
C	-1.338134	2.541725	0.000001	O	5.211050	-0.588450	-0.000047
C	-1.840781	-1.740730	0.000001	C	-3.134562	-2.298971	0.000000
C	-4.249075	-1.454289	-0.000002	C	-4.086191	-0.072337	-0.000002
H	1.474498	2.979769	-0.000004	H	3.924617	1.660263	0.000036
H	3.469595	-2.619388	-0.000042	H	1.016335	-2.415063	0.000010
H	-3.503908	2.565429	-0.000002	H	-1.224298	3.620318	-0.000001
H	5.470308	-1.516916	0.000353	H	-0.987802	-2.406760	0.000002
H	-3.260114	-3.376688	0.000001	H	-5.250050	-1.876850	-0.000003
H	-4.958209	0.575923	-0.000005				

13s$^{\bullet-}$ (anion radical) b3lyp/6-311+g(d,p) scrf=(cpcm,solvent=acetonitrile)

```
# opt freq b3lyp/6-311+g(d,p)
scrf=(cpcm,solvent=acetonitrile)
```

Charge = -1, Multiplicity = 2, Point group = C1

Electronic Energy = -592.913246 Hartree

Number of imaginary frequencies = 0

Sum of electronic and zero-point Energies = -592.739397 Hartree

Sum of electronic and thermal Energies = -592.728008 Hartree

Sum of electronic and thermal Enthalpies = -592.727064 Hartree

Sum of electronic and thermal Free Energies = -592.776889 Hartree

	Cartesian Coordinates				Cartesian Coordinates		
	X	*Y*	*Z*		*X*	*Y*	*Z*
C	-0.983867	-0.498366	-0.000013	C	-0.352961	0.791401	-0.000023
C	1.049946	0.571670	-0.000019	C	1.267117	-0.846008	-0.000006
N	0.019496	-1.463665	-0.000006	C	-2.354511	-0.649553	-0.000020
C	-3.172887	0.506837	-0.000018	C	-2.571693	1.778208	0.000021
C	-1.188021	1.948632	0.000008	C	2.197417	1.431755	-0.000001
C	3.473356	0.847874	0.000012	C	3.659318	-0.537811	0.000015
C	2.530717	-1.419917	0.000012	O	-2.876382	-1.939336	-0.000104
H	-0.137430	-2.458028	0.000014	H	-4.253241	0.402737	-0.000071
H	-3.214453	2.653994	0.000041	H	-0.757431	2.944336	0.000025
H	2.082903	2.510625	-0.000002	H	4.347295	1.494800	0.000022
H	4.662267	-0.951344	0.000026	H	2.659625	-2.497254	0.000027
H	-3.838538	-1.887847	0.000984				

13t$^{\bullet-}$ (anion radical) b3lyp/6-311+g(d,p) scrf=(cpcm,solvent=acetonitrile)

```
# opt freq b3lyp/6-311+g(d,p)
scrf=(cpcm,solvent=acetonitrile)
```

Charge = -1, Multiplicity = 2, Point group = C1

Electronic Energy = -900.276759 Hartree

Number of imaginary frequencies = 0

Sum of electronic and zero-point Energies = -900.008779 Hartree

Sum of electronic and thermal Energies = -899.992451 Hartree

Sum of electronic and thermal Enthalpies = -899.991507 Hartree

Sum of electronic and thermal Free Energies = -900.052228 Hartree

	**Cartesian Coordinates**				**Cartesian Coordinates**		
	X	***Y***	***Z***		***X***	***Y***	***Z***
C	1.232613	-1.619217	0.047041	C	0.625550	-0.330650	-0.000912
C	-0.796582	-0.553065	-0.037739	C	-0.993067	-1.969041	-0.136126
N	0.245618	-2.582405	-0.041800	C	2.599686	-1.826172	0.203904
C	3.450130	-0.727996	0.277073	C	2.935697	0.582704	0.102856
C	1.518148	0.812046	-0.089746	C	-1.990612	0.273762	0.091093
C	-3.275802	-0.358144	-0.171065	C	-3.366627	-1.746987	-0.400879
C	-2.228479	-2.577224	-0.330609	O	3.031226	-3.137947	0.285143
C	-1.996416	1.608541	0.534927	C	-3.166316	2.369750	0.622904
C	-4.394806	1.790903	0.259292	C	-4.447394	0.464192	-0.122783
C	3.815552	1.702568	0.056795	C	3.365212	2.971039	-0.242125
C	2.001102	3.179694	-0.524963	C	1.112220	2.117413	-0.447090
H	0.413464	-3.573806	-0.092030	H	4.518035	-0.872235	0.407529
H	-4.344790	-2.184646	-0.573288	H	-2.307652	-3.653867	-0.434275
H	3.992855	-3.153907	0.351747	H	-1.066342	2.061948	0.846902
H	-3.120662	3.397321	0.966563	H	-5.306778	2.379267	0.297677
H	-5.403117	0.006795	-0.363321	H	4.872985	1.531078	0.235764
H	4.063787	3.800592	-0.280863	H	1.644169	4.164773	-0.804743
H	0.076069	2.292425	-0.697300				

$11^{\bullet+}$ (cation radical) b3lyp/6-311+g(d,p) scrf=(cpcm,solvent=acetonitrile)

```
# opt freq b3lyp/6-311+g(d,p)
scrf=(cpcm,solvent=acetonitrile)
```

Charge = 1, Multiplicity = 2, Point group = C1

Electronic Energy = -517.397626 Hartree

Number of imaginary frequencies = 0

Sum of electronic and zero-point Energies = -517.222495 Hartree

Sum of electronic and thermal Energies = -517.213506 Hartree

Sum of electronic and thermal Enthalpies = -517.212562 Hartree

Sum of electronic and thermal Free Energies = -517.257193 Hartree

	Cartesian Coordinates				Cartesian Coordinates		
	X	*Y*	*Z*		*X*	*Y*	*Z*
C	1.130596	0.830843	-0.000000	C	0.732429	-0.536237	-0.000000
C	-0.732429	-0.536237	-0.000000	C	-1.130596	0.830843	0.000000
N	0.000000	1.608600	-0.000000	C	2.471734	1.234462	-0.000000
C	3.434132	0.228173	0.000000	C	3.059972	-1.119629	0.000000
C	1.703738	-1.515421	0.000000	C	-1.703738	-1.515421	-0.000000
C	-3.059972	-1.119629	-0.000000	C	-3.434132	0.228173	0.000000
C	-2.471734	1.234462	0.000000	H	0.000000	2.623872	-0.000000
H	2.742901	2.282759	-0.000000	H	4.483715	0.492488	0.000000
H	3.828187	-1.883106	0.000000	H	1.447060	-2.567325	0.000000
H	-1.447060	-2.567325	-0.000000	H	-3.828187	-1.883106	-0.000000
H	-4.483715	0.492488	0.000000	H	-2.742901	2.282759	0.000000

13a$^{\bullet+}$ (cation radical) b3lyp/6-311+g(d,p) scrf=(cpcm,solvent=acetonitrile)

```
# opt freq b3lyp/6-311+g(d,p)
scrf=(cpcm,solvent=acetonitrile)
```

Charge = 1, Multiplicity = 2, Point group = C1

Electronic Energy = -671.083490 Hartree

Number of imaginary frequencies = 0

Sum of electronic and zero-point Energies = -670.860757 Hartree

Sum of electronic and thermal Energies = -670.849070 Hartree

Sum of electronic and thermal Enthalpies = -670.848126 Hartree

Sum of electronic and thermal Free Energies = -670.899374 Hartree

	Cartesian Coordinates				Cartesian Coordinates		
	X	*Y*	*Z*		*X*	*Y*	*Z*
C	-2.288405	0.702509	-0.000004	C	-1.372311	-0.384760	0.000001
C	-0.053662	0.185140	-0.000001	C	-0.240049	1.628882	-0.000003
N	-1.557620	1.895105	-0.000007	C	-3.658179	0.533158	-0.000005
C	-4.140099	-0.784194	0.000001	C	-3.268541	-1.878575	0.000009
C	-1.889209	-1.694485	0.000010	C	1.272426	-0.335040	-0.000002
C	2.354224	0.605612	0.000005	C	2.096499	2.024035	0.000007
C	0.831324	2.541605	0.000001	C	1.577383	-1.714828	-0.000011
C	2.894563	-2.156401	-0.000009	C	3.943277	-1.235734	0.000001
C	3.670853	0.134154	0.000007	H	-1.962844	2.821566	-0.000008
H	-4.334718	1.378303	-0.000010	H	-5.210047	-0.952847	0.000001
H	-3.672530	-2.882898	0.000016	H	-1.241257	-2.559271	0.000020
H	2.949621	2.692236	0.000012	H	0.650878	3.608475	0.000001
H	0.782125	-2.445278	-0.000022	H	3.103477	-3.218883	-0.000017
H	4.970184	-1.579953	0.000003	H	4.487883	0.846343	0.000013

13b$^{•+}$ (cation radical) b3lyp/6-311+g(d,p) scrf=(cpcm,solvent=acetonitrile)

```
# opt freq b3lyp/6-311+g(d,p)
scrf=(cpcm,solvent=acetonitrile)
```

Charge = 1, Multiplicity = 2, Point group = C1

Electronic Energy = -671.087226 Hartree

Number of imaginary frequencies = 0

Sum of electronic and zero-point Energies = -670.863921 Hartree

Sum of electronic and thermal Energies = -670.852414 Hartree

Sum of electronic and thermal Enthalpies = -670.851470 Hartree

Sum of electronic and thermal Free Energies = -670.901988 Hartree

	Cartesian Coordinates				Cartesian Coordinates		
	X	*Y*	*Z*		*X*	*Y*	*Z*
C	1.996972	-0.876579	0.000000	C	1.928595	0.533773	-0.000000
C	0.525776	0.879287	-0.000000	C	-0.204537	-0.368500	0.000000
N	0.681797	-1.376093	0.000000	C	3.194173	-1.567664	0.000000
C	4.369341	-0.800288	0.000000	C	4.328438	0.595853	-0.000000
C	3.108691	1.279263	-0.000000	C	-0.165962	2.085998	0.000000
C	-1.555583	2.063962	0.000000	C	-2.305094	0.845450	0.000000
C	-1.635392	-0.416865	0.000000	C	-3.712768	0.865917	0.000000
C	-4.440299	-0.318547	-0.000000	C	-3.777832	-1.549244	-0.000000
C	-2.386433	-1.600835	0.000000	H	0.452017	-2.361623	-0.000000
H	3.230611	-2.649710	0.000000	H	5.326676	-1.306951	0.000000
H	5.254558	1.156956	-0.000000	H	3.084774	2.362147	-0.000000
H	0.363417	3.030206	0.000000	H	-2.102593	2.999356	0.000000
H	-4.226235	1.820282	0.000000	H	-5.522780	-0.286824	-0.000000
H	-4.346328	-2.470902	-0.000000	H	-1.895219	-2.566172	0.000000

13c$^{•+}$ (cation radical) b3lyp/6-311+g(d,p) scrf=(cpcm,solvent=acetonitrile)

```
# opt freq b3lyp/6-311+g(d,p)
scrf=(cpcm,solvent=acetonitrile)
```

Charge = 1, Multiplicity = 2, Point group = C1

Electronic Energy = -671.088201 Hartree

Number of imaginary frequencies = 0

Sum of electronic and zero-point Energies = -670.865435 Hartree

Sum of electronic and thermal Energies = -670.853906 Hartree

Sum of electronic and thermal Enthalpies = -670.852961 Hartree

Sum of electronic and thermal Free Energies = -670.903552 Hartree

	Cartesian Coordinates				Cartesian Coordinates		
	X	*Y*	*Z*		*X*	*Y*	*Z*

C	2.412217	-0.802956	-0.000000	C	1.860658	0.512378	-0.000000
C	0.420503	0.349993	-0.000001	C	0.171388	-1.061475	-0.000000
N	1.378223	-1.710718	-0.000000	C	3.791853	-1.040116	0.000000
C	4.618738	0.070875	0.000001	C	4.091139	1.379869	0.000000
C	2.718656	1.611467	-0.000000	C	-0.644388	1.221455	-0.000000
C	-1.981299	0.712879	-0.000000	C	-2.209766	-0.705412	-0.000000
C	-1.107064	-1.598498	-0.000000	C	-3.087537	1.578182	0.000000
C	-4.386990	1.075557	0.000000	C	-4.611195	-0.305599	0.000001
C	-3.537787	-1.186522	0.000000	H	1.493722	-2.716801	-0.000000
H	4.190290	-2.046689	0.000001	H	5.693170	-0.065454	0.000001
H	4.773116	2.221429	0.000000	H	2.330539	2.622490	-0.000001
H	-0.490449	2.294518	-0.000000	H	-1.270197	-2.669436	-0.000000
H	-2.921503	2.649254	0.000000	H	-5.226776	1.759593	0.000001
H	-5.623747	-0.689749	0.000001	H	-3.710488	-2.256581	0.000000

13d$^{\bullet+}$ (cation radical) b3lyp/6-311+g(d,p) scrf=(cpcm,solvent=acetonitrile)

```
# opt freq b3lyp/6-311+g(d,p)
scrf=(cpcm,solvent=acetonitrile)
```

Charge = 1, Multiplicity = 2, Point group = C1

Electronic Energy = -787.831086 Hartree

Number of imaginary frequencies = 0

Sum of electronic and zero-point Energies = -787.548096 Hartree

Sum of electronic and thermal Energies = -787.532645 Hartree

Sum of electronic and thermal Enthalpies = -787.531701 Hartree

Sum of electronic and thermal Free Energies = -787.592354 Hartree

	Cartesian Coordinates				Cartesian Coordinates		
	X	*Y*	*Z*		*X*	*Y*	*Z*
C	-0.946834	1.137296	-0.037491	C	-2.302833	0.725850	-0.022988
C	-2.299532	-0.733259	0.025856	C	-0.941723	-1.138636	0.037161
N	-0.131886	0.001156	-0.000950	C	-0.575579	2.480032	-0.151763
C	-1.600142	3.423784	-0.201230	C	-2.941669	3.032662	-0.162367
C	-3.305186	1.676792	-0.084558	C	-3.297471	-1.688722	0.089418
C	-2.927693	-3.042957	0.165899	C	-1.584330	-3.428087	0.201305
C	-0.564136	-2.479707	0.149768	C	1.281011	0.003844	-0.001822
C	1.980932	-0.822545	-0.894258	C	3.366254	-0.804702	-0.893803
C	4.085937	0.011104	-0.005825	C	3.364708	0.826406	0.880947
C	1.979123	0.835573	0.886817	C	5.588879	-0.007547	0.011742
H	0.460217	2.783289	-0.213882	H	-1.349213	4.473748	-0.281863
H	-3.717772	3.786766	-0.208099	H	-4.349742	1.391265	-0.082685
H	-4.343294	-1.407880	0.089981	H	-3.700294	-3.800554	0.213183

H	-1.328513	-4.476962	0.280765	H	0.473201	-2.778269	0.208927
H	1.441952	-1.429030	-1.610389	H	3.901308	-1.423619	-1.604671
H	3.898694	1.455307	1.583838	H	1.439192	1.443814	1.600714
H	5.995072	-0.225210	-0.977700	H	5.995178	0.943699	0.359537
H	5.948912	-0.787523	0.692155				

13e$^{\bullet+}$ (cation radical) b3lyp/6-311+g(d,p) scrf=(cpcm,solvent=acetonitrile)

```
# opt freq b3lyp/6-311+g(d,p)
scrf=(cpcm,solvent=acetonitrile)
```

Charge = 1, Multiplicity = 2, Point group = C1

Electronic Energy = -941.512389 Hartree

Number of imaginary frequencies = 0

Sum of electronic and zero-point Energies = -941.182735 Hartree

Sum of electronic and thermal Energies = -941.164347 Hartree

Sum of electronic and thermal Enthalpies = -941.163403 Hartree

Sum of electronic and thermal Free Energies = -941.231519 Hartree

	Cartesian Coordinates				Cartesian Coordinates		
	X	***Y***	***Z***		***X***	***Y***	***Z***
C	-0.341093	1.580450	0.029532	C	1.075578	1.614921	0.037045
C	1.523519	0.244287	-0.000989	C	0.324907	-0.570232	-0.035165
N	-0.763618	0.230765	-0.016531	C	-1.121184	2.717127	0.084606
C	-0.454912	3.952624	0.133716	C	0.938027	4.023059	0.133392
C	1.713675	2.863306	0.088807	C	2.792460	-0.394693	-0.018170
C	2.814395	-1.828211	-0.077520	C	1.585258	-2.579450	-0.125720
C	0.359994	-1.980553	-0.105498	C	4.026033	0.299928	0.020843
C	5.231068	-0.384649	0.003298	C	5.244775	-1.781700	-0.054865
C	4.045293	-2.492531	-0.095479	C	-2.131685	-0.201393	-0.010942
C	-2.954917	0.100357	-1.095986	C	-4.280602	-0.320479	-1.082835
C	-4.804814	-1.040040	-0.000722	C	-3.955870	-1.331687	1.074057
C	-2.627851	-0.915753	1.079061	C	-6.250868	-1.465763	0.016149
H	-2.201834	2.666381	0.091802	H	-1.039504	4.863500	0.173774
H	1.426791	4.988322	0.171746	H	2.790500	2.950630	0.097926
H	1.654498	-3.659430	-0.183935	H	-0.554208	-2.556159	-0.146568
H	4.038547	1.378414	0.067019	H	6.162447	0.166779	0.034853
H	6.187181	-2.315458	-0.068732	H	4.060693	-3.575191	-0.141442
H	-2.558093	0.642199	-1.945909	H	-4.914458	-0.092750	-1.932707
H	-4.337559	-1.887478	1.923243	H	-1.986127	-1.134869	1.923958
H	-6.611505	-1.686007	-0.990651	H	-6.397639	-2.349985	0.639134
H	-6.881522	-0.667798	0.422507				

13f$^{\bullet+}$ (cation radical) b3lyp/6-311+g(d,p) scrf=(cpcm,solvent=acetonitrile)

```
# opt freq b3lyp/6-311+g(d,p)
scrf=(cpcm,solvent=acetonitrile)
```

Charge = 1, Multiplicity = 2, Point group = C1

Electronic Energy = -941.513050 Hartree

Number of imaginary frequencies = 0

Sum of electronic and zero-point Energies = -941.183006 Hartree

Sum of electronic and thermal Energies = -941.164711 Hartree

Sum of electronic and thermal Enthalpies = -941.163767 Hartree

Sum of electronic and thermal Free Energies = -941.231510 Hartree

	Cartesian Coordinates				Cartesian Coordinates		
	X	*Y*	*Z*		*X*	*Y*	*Z*
C	0.352060	2.006540	0.000060	C	1.757881	2.057998	0.000100
C	2.216831	0.690748	0.000181	C	1.040064	-0.152463	0.000159
N	-0.053585	0.646511	0.000177	C	-0.437880	3.141542	0.000038
C	0.227339	4.376987	0.000006	C	1.621854	4.453519	0.000047
C	2.404044	3.294712	0.000115	C	3.489789	0.133932	0.000128
C	3.616288	-1.247369	-0.000026	C	2.482647	-2.115397	-0.000105
C	1.149681	-1.588362	-0.000040	C	2.670888	-3.511362	-0.000271
C	1.586718	-4.378713	-0.000377	C	0.289172	-3.863654	-0.000372
C	0.070228	-2.488089	-0.000174	C	-1.445597	0.270051	0.000148
C	-2.113755	0.111194	-1.211695	C	-3.462757	-0.232634	-1.202360
C	-4.158813	-0.410597	0.000007	C	-3.462366	-0.234617	1.202459
C	-2.113365	0.109188	1.211938	C	-5.626800	-0.755014	-0.000020
H	-1.518597	3.087081	0.000001	H	-0.358464	5.288051	-0.000035
H	2.103682	5.423313	-0.000008	H	3.485160	3.361093	0.000232
H	4.369643	0.764421	0.000199	H	4.603267	-1.694594	-0.000140
H	3.681342	-3.902938	-0.000345	H	1.748481	-5.449475	-0.000445
H	-0.561098	-4.534458	-0.000523	H	-0.945248	-2.123869	-0.000122
H	-1.582768	0.244873	-2.146456	H	-3.979992	-0.364725	-2.146201
H	-3.979319	-0.368277	2.146231	H	-1.582072	0.241214	2.146761
H	-5.899700	-1.330351	-0.886708	H	-5.899414	-1.331718	0.885868
H	-6.234722	0.155944	0.000775				

13g$^{\bullet+}$ (cation radical) b3lyp/6-311+g(d,p) scrf=(cpcm,solvent=acetonitrile)

```
# opt freq b3lyp/6-311+g(d,p)
scrf=(cpcm,solvent=acetonitrile)
```

Charge = 1, Multiplicity = 2, Point group = C1

Electronic Energy = -941.518377 Hartree

Number of imaginary frequencies = 0

Sum of electronic and zero-point Energies = -941.188618 Hartree

Sum of electronic and thermal Energies = -941.170397 Hartree

Sum of electronic and thermal Enthalpies = -941.169453 Hartree

Sum of electronic and thermal Free Energies = -941.236718 Hartree

	**Cartesian Coordinates**				**Cartesian Coordinates**		
	X	***Y***	***Z***		***X***	***Y***	***Z***
C	-0.793913	1.857636	0.031430	C	0.534009	2.365069	0.041022
C	1.416792	1.215283	0.029141	C	0.575271	0.056947	0.008104
N	-0.748235	0.465833	0.013343	C	-1.913134	2.696260	0.078800
C	-1.678333	4.062648	0.110970	C	-0.369237	4.581372	0.104991
C	0.742064	3.741977	0.075904	C	2.781229	1.062520	0.014226
C	3.351084	-0.248765	-0.024375	C	2.492706	-1.399005	-0.064268
C	1.083810	-1.230473	-0.055065	C	4.743727	-0.439683	-0.036567
C	5.286019	-1.719423	-0.084521	C	4.449482	-2.843710	-0.124790
C	3.072336	-2.687756	-0.116160	C	-1.884368	-0.400335	0.007916
C	-2.833247	-0.292467	-1.011665	C	-3.931986	-1.143063	-1.011883
C	-4.110072	-2.104124	-0.006970	C	-3.143165	-2.192875	1.002780
C	-2.036339	-1.351590	1.019322	C	-5.322238	-2.998839	-0.000002
H	-2.918765	2.298855	0.095538	H	-2.519162	4.744221	0.146873
H	-0.226168	5.654693	0.131904	H	1.743295	4.154393	0.084813
H	3.441733	1.921962	0.024558	H	0.434348	-2.095040	-0.104465
H	5.396434	0.425207	-0.009760	H	6.361667	-1.846720	-0.092705
H	4.880305	-3.836574	-0.163851	H	2.424395	-3.556063	-0.148101
H	-2.697604	0.428859	-1.807853	H	-4.657695	-1.065169	-1.813631
H	-3.259490	-2.925424	1.793549	H	-1.309994	-1.416441	1.820022
H	-5.661326	-3.215815	-1.014883	H	-5.117092	-3.942589	0.508491
H	-6.152203	-2.514817	0.526043				

13h$^{\bullet+}$ (cation radical) b3lyp/6-311+g(d,p) scrf=(cpcm,solvent=acetonitrile)

```
# opt freq b3lyp/6-311+g(d,p)
scrf=(cpcm,solvent=acetonitrile)
```

Charge = 1, Multiplicity = 2, Point group = C1

Electronic Energy = -592.658250 Hartree

Number of imaginary frequencies = 0

Sum of electronic and zero-point Energies = -592.477292 Hartree

Sum of electronic and thermal Energies = -592.467342 Hartree

Sum of electronic and thermal Enthalpies = -592.466398 Hartree

Sum of electronic and thermal Free Energies = -592.513249 Hartree

	**Cartesian Coordinates**				**Cartesian Coordinates**		
	X	***Y***	***Z***		***X***	***Y***	***Z***

C	0.642760	1.129542	0.000000	C	0.431777	-0.295791	0.000000
C	-1.012948	-0.492576	-0.000000	C	-1.592037	0.796010	-0.000001
N	-0.557838	1.741072	-0.000000	C	1.936503	1.700879	0.000001
C	3.007066	0.847244	0.000000	C	2.808236	-0.563924	-0.000001
C	1.514907	-1.141266	-0.000000	C	-1.836502	-1.611269	0.000001
C	-3.225571	-1.418610	0.000001	C	-3.776558	-0.135912	-0.000000
C	-2.962749	1.003779	-0.000001	O	3.915161	-1.306508	0.000000
H	-0.696089	2.744664	-0.000001	H	2.072163	2.774442	0.000002
H	4.023324	1.218605	0.000000	H	1.402779	-2.219055	0.000000
H	-1.422297	-2.612041	0.000002	H	-3.880821	-2.280795	0.000001
H	-4.852771	-0.015322	-0.000001	H	-3.387295	1.999633	-0.000002
H	3.715267	-2.254195	-0.000011				

13i$^{\bullet+}$ (cation radical) b3lyp/6-311+g(d,p) scrf=(cpcm,solvent=acetonitrile)

```
# opt freq b3lyp/6-311+g(d,p)
scrf=(cpcm,solvent=acetonitrile)
```

Charge = 1, Multiplicity = 2, Point group = C1

Electronic Energy = -746.334425 Hartree

Number of imaginary frequencies = 0

Sum of electronic and zero-point Energies = -746.107503 Hartree

Sum of electronic and thermal Energies = -746.094746 Hartree

Sum of electronic and thermal Enthalpies = -746.093802 Hartree

Sum of electronic and thermal Free Energies = -746.147195 Hartree

	**Cartesian Coordinates**				**Cartesian Coordinates**		
	X	***Y***	***Z***		***X***	***Y***	***Z***
C	-1.886363	-1.225316	0.000003	C	-1.136953	0.014296	0.000001
C	0.269729	-0.362632	-0.000000	C	0.299699	-1.761455	0.000000
N	-1.015773	-2.244886	0.000003	C	-3.301432	-1.268432	0.000004
C	-3.973629	-0.078694	0.000001	C	-3.251966	1.152283	-0.000003
C	-1.837245	1.202043	-0.000005	C	1.495000	0.361734	0.000001
C	2.714120	-0.409189	-0.000002	C	2.669221	-1.827802	-0.000004
C	1.476096	-2.520009	-0.000002	C	1.592797	1.775668	0.000006
C	2.820463	2.398119	0.000006	C	4.017315	1.643367	-0.000000
C	3.961624	0.271482	-0.000004	O	-3.996966	2.256454	-0.000003
H	-1.267969	-3.225722	0.000003	H	-3.826710	-2.214893	0.000006
H	-5.054648	-0.038216	0.000001	H	-1.348424	2.166419	-0.000011
H	3.604710	-2.374819	-0.000006	H	1.448795	-3.602261	-0.000002
H	0.698457	2.381940	0.000013	H	2.871433	3.480649	0.000010
H	4.973846	2.152032	-0.000002	H	4.872693	-0.316249	-0.000007
H	-3.456893	3.060919	-0.000014				

13j$^{\bullet+}$ (cation radical) b3lyp/6-311+g(d,p) scrf=(cpcm,solvent=acetonitrile)

```
# opt freq b3lyp/6-311+g(d,p)
scrf=(cpcm,solvent=acetonitrile)
```

Charge = 1, Multiplicity = 2, Point group = C1

Electronic Energy = -746.338255 Hartree

Number of imaginary frequencies = 0

Sum of electronic and zero-point Energies = -746.111603 Hartree

Sum of electronic and thermal Energies = -746.098800 Hartree

Sum of electronic and thermal Enthalpies = -746.097856 Hartree

Sum of electronic and thermal Free Energies = -746.151173 Hartree

	Cartesian Coordinates				Cartesian Coordinates		
	X	***Y***	***Z***		***X***	***Y***	***Z***
C	1.574744	-1.052176	0.000004	C	1.588794	0.362068	0.000003
C	0.194621	0.786669	0.000002	C	-0.591947	-0.406967	0.000002
N	0.247804	-1.467737	0.000003	C	2.742304	-1.806334	0.000004
C	3.956668	-1.121810	0.000000	C	3.986377	0.278909	-0.000002
C	2.794547	1.039201	0.000001	C	-0.428283	2.029198	0.000002
C	-1.819960	2.081545	0.000002	C	-2.628118	0.909040	0.000001
C	-2.021509	-0.388083	0.000000	C	-4.039566	1.000129	0.000000
C	-4.821305	-0.140645	-0.000003	C	-4.217024	-1.407842	-0.000005
C	-2.834773	-1.532991	-0.000004	O	5.139556	0.986428	-0.000011
H	-0.039941	-2.438487	0.000006	H	2.720473	-2.888959	0.000005
H	4.886065	-1.678825	-0.000001	H	2.856044	2.120105	0.000003
H	0.150774	2.943917	0.000003	H	-2.314952	3.045540	0.000003
H	-4.501065	1.980842	0.000001	H	-5.901200	-0.058582	-0.000004
H	-4.833502	-2.298453	-0.000008	H	-2.393921	-2.522754	-0.000006
H	5.906713	0.398930	0.000012				

13k$^{\bullet+}$ (cation radical) b3lyp/6-311+g(d,p) scrf=(cpcm,solvent=acetonitrile)

```
# opt freq b3lyp/6-311+g(d,p)
scrf=(cpcm,solvent=acetonitrile)
```

Charge = 1, Multiplicity = 2, Point group = C1

Electronic Energy = -746.343012 Hartree

Number of imaginary frequencies = 0

Sum of electronic and zero-point Energies = -746.116398 Hartree

Sum of electronic and thermal Energies = -746.103643 Hartree

Sum of electronic and thermal Enthalpies = -746.102698 Hartree

Sum of electronic and thermal Free Energies = -746.155943 Hartree

Cartesian Coordinates	Cartesian Coordinates

	X	Y	Z		X	Y	Z
C	-1.987505	1.161919	0.000000	C	-1.557458	-0.203732	0.000000
C	-0.106415	-0.172120	0.000001	C	0.269857	1.210874	0.000001
N	-0.888052	1.968081	0.000000	C	-3.349233	1.513525	-0.000000
C	-4.268911	0.491525	-0.000000	C	-3.852204	-0.866985	-0.000000
C	-2.495608	-1.221765	0.000000	C	0.868773	-1.139866	0.000001
C	2.243902	-0.757421	0.000001	C	2.600712	0.634317	0.000000
C	1.581939	1.627848	0.000000	C	3.269563	-1.725326	0.000000
C	4.602365	-1.346622	-0.000000	C	4.952169	0.014351	-0.000001
C	3.969250	0.988639	-0.000001	O	-4.839151	-1.782700	-0.000001
H	-0.914938	2.980620	-0.000000	H	-3.662007	2.549605	-0.000000
H	-5.331130	0.699703	-0.000001	H	-2.201381	-2.264567	0.000001
H	0.614879	-2.193639	0.000001	H	1.844136	2.679311	-0.000000
H	3.001309	-2.775726	0.000001	H	5.378157	-2.102715	-0.000001
H	5.996660	0.301776	-0.000002	H	4.240855	2.038039	-0.000001
H	-4.484139	-2.682335	-0.000003				

13l$^{\bullet +}$ (cation radical) b3lyp/6-311+g(d,p) scrf=(cpcm,solvent=acetonitrile)

```
# opt freq b3lyp/6-311+g(d,p)
scrf=(cpcm,solvent=acetonitrile)
```

Charge = 1, Multiplicity = 2, Point group = C1

Electronic Energy = -863.088942 Hartree

Number of imaginary frequencies = 0

Sum of electronic and zero-point Energies = -862.801119 Hartree

Sum of electronic and thermal Energies = -862.784519 Hartree

Sum of electronic and thermal Enthalpies = -862.783574 Hartree

Sum of electronic and thermal Free Energies = -862.846996 Hartree

	Cartesian Coordinates				Cartesian Coordinates		
	X	Y	Z		X	Y	Z
C	0.883641	-0.741960	-0.041157	C	2.145881	-0.066027	-0.004099
C	1.850474	1.359548	0.039577	C	0.444842	1.481110	0.030706
N	-0.121153	0.183666	-0.019764	C	0.798190	-2.147685	-0.136972
C	1.970294	-2.863013	-0.157522	C	3.223314	-2.199820	-0.096959
C	3.315129	-0.790763	-0.029896	C	2.639056	2.499957	0.107416
C	2.005268	3.748780	0.167210	C	0.613259	3.848829	0.173371
C	-0.196894	2.709072	0.111870	C	-1.518907	-0.099517	-0.011902
C	-2.347577	0.471423	-0.982267	C	-3.706225	0.183757	-0.970812
C	-4.265052	-0.660829	-0.000753	C	-3.413024	-1.219316	0.961010
C	-2.050186	-0.947082	0.963818	C	-5.745427	-0.937665	0.020717
O	4.307015	-2.983843	-0.120561	H	-0.157338	-2.649770	-0.200405

H	1.964008	-3.942606	-0.229734	H	4.285413	-0.308454	-0.005196
H	3.719993	2.432704	0.120108	H	2.606176	4.648346	0.217767
H	0.145850	4.823957	0.231515	H	-1.275004	2.791711	0.133333
H	-1.928070	1.106135	-1.752592	H	-4.341844	0.615191	-1.735847
H	-3.821133	-1.870867	1.725323	H	-1.408995	-1.365823	1.729433
H	-6.159562	-0.959596	-0.989355	H	-5.965311	-1.888671	0.508881
H	-6.273264	-0.152893	0.573317	H	5.124693	-2.467071	-0.083851

13m$^{\bullet+}$ (cation radical) b3lyp/6-311+g(d,p) scrf=(cpcm,solvent=acetonitrile)

```
# opt freq b3lyp/6-311+g(d,p)
scrf=(cpcm,solvent=acetonitrile)
```

Charge = 1, Multiplicity = 2, Point group = C1

Electronic Energy = -1170.439197 Hartree

Number of imaginary frequencies = 0

Sum of electronic and zero-point Energies = -1170.058861 Hartree

Sum of electronic and thermal Energies = -1170.036353 Hartree

Sum of electronic and thermal Enthalpies = -1170.035409 Hartree

Sum of electronic and thermal Free Energies = -1170.112954 Hartree

	**Cartesian Coordinates**				**Cartesian Coordinates**		
	X	*Y*	*Z*		*X*	*Y*	*Z*
C	-0.435053	1.808520	-0.015361	C	0.970954	1.863740	-0.019345
C	1.429106	0.486793	-0.007795	C	0.245928	-0.346164	-0.017203
N	-0.842981	0.456873	-0.012537	C	-1.233916	2.938397	-0.017019
C	-0.602802	4.182379	-0.030289	C	0.792094	4.271205	-0.044096
C	1.591730	3.119251	-0.040119	C	2.718391	-0.099859	0.025552
C	2.826015	-1.531208	0.012535	C	1.619429	-2.370434	-0.031756
C	0.318652	-1.785217	-0.040024	C	1.710619	-3.771546	-0.067212
C	0.581464	-4.577951	-0.108281	C	-0.688865	-4.001361	-0.116669
C	-0.816404	-2.623318	-0.083046	C	-2.243328	0.110497	0.011904
C	-2.950793	0.025688	-1.184626	C	-4.309034	-0.278592	-1.149467
C	-4.975408	-0.488020	0.064425	C	-4.239634	-0.383674	1.251926
C	-2.881263	-0.080269	1.235400	C	-6.452657	-0.788996	0.094416
O	1.330164	5.521870	-0.063359	C	3.903501	0.683724	0.080860
C	5.148880	0.098154	0.112343	C	5.251982	-1.299704	0.092204
C	4.110282	-2.090581	0.045471	H	-2.313554	2.869536	-0.009523
H	-1.187113	5.093684	-0.032840	H	2.665652	3.236359	-0.059260
H	2.676637	-4.254744	-0.065471	H	0.694226	-5.655037	-0.135224
H	-1.575081	-4.622819	-0.150806	H	-1.805102	-2.195612	-0.093518
H	-2.442907	0.182921	-2.128487	H	-4.856825	-0.354568	-2.082166
H	-4.733383	-0.540978	2.204460	H	-2.319663	-0.002952	2.158572

H	-6.772238	-1.301369	-0.814952	H	-6.713932	-1.410277	0.953255
H	-7.031669	0.137455	0.171047	H	2.294395	5.480549	-0.066600
H	3.834738	1.758714	0.106732	H	6.039116	0.712854	0.155667
H	6.226216	-1.772886	0.117216	H	4.240825	-3.162626	0.038162

13n$^{\bullet+}$ (cation radical) b3lyp/6-311+g(d,p) scrf=(cpcm,solvent=acetonitrile)

```
# opt freq b3lyp/6-311+g(d,p)
scrf=(cpcm,solvent=acetonitrile)
```

Charge = 1, Multiplicity = 2, Point group = C1

Electronic Energy = -1092.023000 Hartree

Number of imaginary frequencies = 0

Sum of electronic and zero-point Energies = -1091.684411 Hartree

Sum of electronic and thermal Energies = -1091.663875 Hartree

Sum of electronic and thermal Enthalpies = -1091.662931 Hartree

Sum of electronic and thermal Free Energies = -1091.735045 Hartree

	Cartesian Coordinates				Cartesian Coordinates		
	X	*Y*	*Z*		*X*	*Y*	*Z*
C	-0.831544	1.633739	0.012302	C	0.479167	2.178508	0.018538
C	1.391839	1.053969	0.019134	C	0.580478	-0.125381	0.014103
N	-0.751254	0.250352	0.010639	C	-1.972086	2.447212	0.020702
C	-1.779906	3.813559	0.027818	C	-0.477680	4.368217	0.028946
C	0.659297	3.557806	0.026324	C	2.763926	0.961605	0.008532
C	3.384012	-0.323206	-0.009424	C	2.583720	-1.509438	-0.020465
C	1.150107	-1.403233	-0.010507	C	4.787629	-0.452283	-0.021215
C	5.386821	-1.701700	-0.043099	C	4.597966	-2.861371	-0.053580
C	3.214754	-2.768761	-0.042203	C	-1.894937	-0.626231	0.005298
C	-2.601366	-0.829736	-1.181822	C	-3.719106	-1.657365	-1.173176
C	-4.148984	-2.288533	0.001962	C	-3.428428	-2.059954	1.180481
C	-2.309593	-1.230414	1.193957	C	-5.343097	-3.207963	-0.009118
O	-0.408387	5.721962	0.035428	O	0.461457	-2.548515	-0.031563
H	-2.968114	2.024780	0.022612	H	-2.625793	4.489249	0.034331
H	1.649353	3.998393	0.031562	H	3.385003	1.849478	0.008891
H	5.396446	0.444341	-0.013465	H	6.467016	-1.782291	-0.052359
H	5.070055	-3.836114	-0.070861	H	2.609532	-3.665387	-0.050015
H	-2.274491	-0.349475	-2.095988	H	-4.265137	-1.814633	-2.096514
H	-3.748800	-2.526078	2.105300	H	-1.769994	-1.044940	2.115114
H	-6.083099	-2.887687	-0.745064	H	-5.038561	-4.226568	-0.271707
H	-5.821604	-3.249778	0.970901	H	0.510822	6.019062	0.036591
H	-0.496346	-2.396675	0.004359				

13o$^{•+}$ (cation radical) b3lyp/6-311+g(d,p) scrf=(cpcm,solvent=acetonitrile)

```
# opt freq b3lyp/6-311+g(d,p)
scrf=(cpcm,solvent=acetonitrile)
```

Charge = 1, Multiplicity = 2, Point group = C1

Electronic Energy = -592.651842 Hartree

Number of imaginary frequencies = 0

Sum of electronic and zero-point Energies = -592.471810 Hartree

Sum of electronic and thermal Energies = -592.461653 Hartree

Sum of electronic and thermal Enthalpies = -592.460709 Hartree

Sum of electronic and thermal Free Energies = -592.508037 Hartree

	Cartesian Coordinates				Cartesian Coordinates		
	X	***Y***	***Z***		***X***	***Y***	***Z***
C	-0.897255	-1.154778	0.000029	C	-0.628207	0.237091	0.000009
C	0.814781	0.385741	0.000002	C	1.345604	-0.945299	0.000080
N	0.302631	-1.826636	0.000085	C	-2.183009	-1.696438	-0.000067
C	-3.263778	-0.793440	-0.000134	C	-3.046943	0.567161	-0.000076
C	-1.721422	1.106312	0.000055	C	1.706753	1.457257	-0.000174
C	3.075087	1.189848	-0.000140	C	3.576347	-0.129610	0.000006
C	2.722791	-1.215814	0.000094	O	-1.647587	2.438129	0.000175
H	0.397191	-2.835505	0.000101	H	-2.344173	-2.766511	-0.000090
H	-4.276135	-1.175384	-0.000218	H	-3.872317	1.267731	-0.000087
H	1.376157	2.488504	-0.000409	H	3.773431	2.017717	-0.000259
H	4.647246	-0.288939	0.000021	H	3.092746	-2.233107	0.000153
H	-0.736356	2.758727	0.000688				

13p$^{•+}$ (cation radical) b3lyp/6-311+g(d,p) scrf=(cpcm,solvent=acetonitrile)

```
# opt freq b3lyp/6-311+g(d,p)
scrf=(cpcm,solvent=acetonitrile)
```

Charge = 1, Multiplicity = 2, Point group = C1

Electronic Energy = -746.330721 Hartree

Number of imaginary frequencies = 0

Sum of electronic and zero-point Energies = -746.103296 Hartree

Sum of electronic and thermal Energies = -746.090632 Hartree

Sum of electronic and thermal Enthalpies = -746.089688 Hartree

Sum of electronic and thermal Free Energies = -746.142585 Hartree

	Cartesian Coordinates				Cartesian Coordinates		
	X	***Y***	***Z***		***X***	***Y***	***Z***
C	2.166068	0.988968	-0.109160	C	1.309082	-0.148043	-0.000969
C	-0.040907	0.346105	-0.006516	C	0.069383	1.787088	-0.007816
N	1.369537	2.126979	-0.100850	C	3.538313	0.915965	-0.192658

C	4.121135	-0.369227	-0.130622	C	3.348959	-1.496945	0.054357
C	1.944179	-1.406047	0.166080	C	-1.350860	-0.231905	-0.058788
C	-2.467942	0.651540	0.110019	C	-2.273678	2.074862	0.205704
C	-1.040845	2.649936	0.107482	O	1.316109	-2.551208	0.464695
C	-1.617744	-1.595018	-0.322727	C	-2.913025	-2.088408	-0.325542
C	-3.992657	-1.234645	-0.075505	C	-3.767895	0.122536	0.121191
H	1.721327	3.075227	-0.123796	H	4.149333	1.803159	-0.293862
H	5.197363	-0.464800	-0.198871	H	3.800905	-2.474742	0.158875
H	-3.151397	2.699308	0.323373	H	-0.905712	3.723333	0.132440
H	0.396178	-2.409275	0.725102	H	-0.818428	-2.265783	-0.607967
H	-3.085297	-3.136296	-0.537486	H	-5.003311	-1.623752	-0.065586
H	-4.605993	0.793875	0.268987				

13q$^{\bullet+}$ (cation radical) b3lyp/6-311+g(d,p) scrf=(cpcm,solvent=acetonitrile)

```
# opt freq b3lyp/6-311+g(d,p)
scrf=(cpcm,solvent=acetonitrile)
```

Charge = 1, Multiplicity = 2, Point group = C1

Electronic Energy = -592.652815 Hartree

Number of imaginary frequencies = 0

Sum of electronic and zero-point Energies = -592.473421 Hartree

Sum of electronic and thermal Energies = -592.463168 Hartree

Sum of electronic and thermal Enthalpies = -592.462223 Hartree

Sum of electronic and thermal Free Energies = -592.509616 Hartree

	**Cartesian Coordinates**				**Cartesian Coordinates**		
	X	***Y***	***Z***		***X***	***Y***	***Z***
C	0.760780	-0.816718	-0.000005	C	0.285137	0.555473	-0.000005
C	-1.121576	0.500491	-0.000001	C	-1.485329	-0.889944	-0.000001
N	-0.327713	-1.647430	-0.000004	C	2.105131	-1.122599	0.000001
C	2.996929	-0.036413	0.000004	C	2.554330	1.331133	0.000000
C	1.222470	1.623863	-0.000005	C	-2.133177	1.492862	0.000001
C	-3.449287	1.088010	0.000004	C	-3.781184	-0.289165	0.000003
C	-2.811111	-1.293505	0.000000	O	4.316233	-0.199998	0.000004
H	-0.291241	-2.656616	-0.000003	H	2.467636	-2.143003	0.000003
H	3.310837	2.104605	-0.000001	H	0.881814	2.651152	-0.000007
H	-1.870546	2.543268	0.000001	H	-4.244912	1.821868	0.000006
H	-4.826564	-0.574295	0.000006	H	-3.088309	-2.339888	0.000000
H	4.566721	-1.136023	0.000009				

13r$^{\bullet+}$ (cation radical) b3lyp/6-311+g(d,p) scrf=(cpcm,solvent=acetonitrile)

```
# opt freq b3lyp/6-311+g(d,p)
scrf=(cpcm,solvent=acetonitrile)
```

Charge = 1, Multiplicity = 2, Point group = C1

Electronic Energy = -746.341013 Hartree

Number of imaginary frequencies = 0

Sum of electronic and zero-point Energies = -746.114172 Hartree

Sum of electronic and thermal Energies = -746.101238 Hartree

Sum of electronic and thermal Enthalpies = -746.100294 Hartree

Sum of electronic and thermal Free Energies = -746.154979 Hartree

	**Cartesian Coordinates**				**Cartesian Coordinates**		
	X	***Y***	***Z***		***X***	***Y***	***Z***
C	1.883311	0.896817	-0.000011	C	1.017823	-0.247196	0.000004
C	-0.313532	0.239541	-0.000001	C	-0.214631	1.685032	-0.000007
N	1.095327	2.038038	-0.000018	C	3.254835	0.803844	-0.000015
C	3.805550	-0.492179	0.000001	C	2.986993	-1.648850	0.000029
C	1.616998	-1.532645	0.000032	C	-1.617022	-0.365566	-0.000005
C	-2.749553	0.508880	0.000013	C	-2.572349	1.934730	0.000019
C	-1.334168	2.528185	0.000004	O	5.143338	-0.570532	-0.000006
C	-1.842672	-1.754805	-0.000031	C	-3.133739	-2.270161	-0.000027
C	-4.237888	-1.413986	0.000001	C	-4.043961	-0.037360	0.000018
H	1.443258	2.986491	-0.000025	H	3.903635	1.669661	-0.000028
H	3.454099	-2.626396	0.000051	H	1.017915	-2.431400	0.000062
H	-3.460399	2.556140	0.000032	H	-1.220732	3.604408	0.000004
H	5.441263	-1.490982	0.000004	H	-1.010169	-2.442882	-0.000061
H	-3.279478	-3.343472	-0.000047	H	-5.241885	-1.820147	0.000005
H	-4.897480	0.630892	0.000034				

13s$^{\bullet +}$ (cation radical) b3lyp/6-311+g(d,p) scrf=(cpcm,solvent=acetonitrile)

```
# opt freq b3lyp/6-311+g(d,p)
scrf=(cpcm,solvent=acetonitrile)
```

Charge = 1, Multiplicity = 2, Point group = C1

Electronic Energy = -592.653956 Hartree

Number of imaginary frequencies = 0

Sum of electronic and zero-point Energies = -592.473610 Hartree

Sum of electronic and thermal Energies = -592.463568 Hartree

Sum of electronic and thermal Enthalpies = -592.462623 Hartree

Sum of electronic and thermal Free Energies = -592.509572 Hartree

	**Cartesian Coordinates**				**Cartesian Coordinates**		
	X	***Y***	***Z***		***X***	***Y***	***Z***
C	-0.960212	-0.483005	-0.000001	C	-0.370363	0.811545	0.000001

C	1.072971	0.591117	0.000001	C	1.264464	-0.816042	0.000000
N	0.020974	-1.422767	-0.000000	C	-2.361930	-0.670252	0.000000
C	-3.173087	0.481607	-0.000001	C	-2.594867	1.737352	-0.000001
C	-1.183971	1.923254	0.000000	C	2.182248	1.425221	0.000000
C	3.455268	0.840555	-0.000000	C	3.624195	-0.552369	-0.000001
C	2.528541	-1.409341	-0.000001	O	-2.817320	-1.921866	0.000003
H	-0.138763	-2.423922	-0.000002	H	-4.250608	0.370223	-0.000002
H	-3.235846	2.609792	-0.000001	H	-0.774017	2.924941	0.000002
H	2.074214	2.502702	0.000000	H	4.330125	1.479073	-0.000000
H	4.624006	-0.967826	-0.000001	H	2.648546	-2.485118	-0.000001
H	-3.785457	-1.953417	-0.000015				

13t•+ (cation radical) b3lyp/6-311+g(d,p) scrf=(cpcm,solvent=acetonitrile)

```
# opt freq b3lyp/6-311+g(d,p)
scrf=(cpcm,solvent=acetonitrile)
```

Charge = 1, Multiplicity = 2, Point group = C1

Electronic Energy = -900.009013 Hartree

Number of imaginary frequencies = 0

Sum of electronic and zero-point Energies = -899.735563 Hartree

Sum of electronic and thermal Energies = -899.720003 Hartree

Sum of electronic and thermal Enthalpies = -899.719059 Hartree

Sum of electronic and thermal Free Energies = -899.778375 Hartree

	Cartesian Coordinates				Cartesian Coordinates		
	X	***Y***	***Z***		***X***	***Y***	***Z***
C	1.194771	-1.624457	0.025449	C	0.613761	-0.321962	-0.023891
C	-0.794521	-0.526093	-0.046147	C	-0.994548	-1.951302	-0.150923
N	0.211575	-2.575127	-0.073645	C	2.557440	-1.859695	0.208678
C	3.396566	-0.753326	0.301434	C	2.908716	0.568835	0.124462
C	1.513043	0.818282	-0.099874	C	-1.974187	0.305851	0.076238
C	-3.244727	-0.312491	-0.157446	C	-3.341673	-1.723250	-0.389023
C	-2.246849	-2.549559	-0.343204	O	2.959836	-3.143907	0.298251
C	-1.969082	1.647129	0.499951	C	-3.146566	2.378372	0.602572
C	-4.377067	1.795201	0.285460	C	-4.421747	0.457741	-0.075948
C	3.827799	1.643887	0.088260	C	3.411558	2.926305	-0.224970
C	2.068179	3.151440	-0.537611	C	1.138348	2.113878	-0.476032
H	0.368842	-3.572527	-0.101777	H	4.457735	-0.904109	0.467349
H	-4.324941	-2.146045	-0.559714	H	-2.338395	-3.621424	-0.461433
H	3.915917	-3.206450	0.424841	H	-1.043755	2.117080	0.795059
H	-3.104292	3.407435	0.938897	H	-5.290145	2.374157	0.350107
H	-5.373205	-0.021095	-0.277934	H	4.874145	1.441560	0.286176

H	4.125459	3.739945	-0.256296	H	1.738528	4.139120	-0.836836
H	0.119111	2.320782	-0.763556				

11_NICS0

```
#p nmr=giao b3lyp/6-311+g(d,p)
```

Charge = 0, Multiplicity = 1, Point group = C1

Electronic Energy = -517.600601 Hartree

	Cartesian Coordinates				Cartesian Coordinates		
	X	*Y*	*Z*		*X*	*Y*	*Z*
C	1.130884	0.856129	0.000000	C	0.724834	-0.505229	0.000000
C	-0.724834	-0.505229	0.000000	C	-1.130884	0.856129	0.000000
N	0.000000	1.654690	0.000000	C	2.479239	1.221047	0.000000
C	3.424861	0.200094	0.000000	C	3.041950	-1.153379	0.000000
C	1.697766	-1.511960	0.000000	C	-1.697766	-1.511960	0.000000
C	-3.041950	-1.153379	0.000000	C	-3.424861	0.200094	0.000000
C	-2.479239	1.221047	0.000000	H	0.000000	2.662849	0.000000
H	2.779401	2.262619	0.000000	H	4.478784	0.454895	0.000000
H	3.805032	-1.923190	0.000000	H	1.409013	-2.557377	0.000000
H	-1.409013	-2.557377	0.000000	H	-3.805032	-1.923190	0.000000
H	-4.478784	0.454895	0.000000	H	-2.779401	2.262619	0.000000
Bq	0.000000	0.471298	0.000000	Bq	2.083256	-0.148883	0.000000
Bq	-2.083256	-0.148883	0.000000				

11_NICS1

```
#p nmr=giao b3lyp/6-311+g(d,p)
```

Charge = 0, Multiplicity = 1, Point group = C1

Electronic Energy = -517.600601 Hartree

	Cartesian Coordinates				Cartesian Coordinates		
	X	*Y*	*Z*		*X*	*Y*	*Z*
C	1.130884	0.856129	0.000000	C	0.724834	-0.505229	0.000000
C	-0.724834	-0.505229	0.000000	C	-1.130884	0.856129	0.000000
N	0.000000	1.654690	0.000000	C	2.479239	1.221047	0.000000
C	3.424861	0.200094	0.000000	C	3.041950	-1.153379	0.000000
C	1.697766	-1.511960	0.000000	C	-1.697766	-1.511960	0.000000
C	-3.041950	-1.153379	0.000000	C	-3.424861	0.200094	0.000000
C	-2.479239	1.221047	0.000000	H	0.000000	2.662849	0.000000
H	2.779401	2.262619	0.000000	H	4.478784	0.454895	0.000000

H	3.805032	-1.923190	0.000000	H	1.409013	-2.557377	0.000000
H	-1.409013	-2.557377	0.000000	H	-3.805032	-1.923190	0.000000
H	-4.478784	0.454895	0.000000	H	-2.779401	2.262619	0.000000
Bq	0.000000	0.471298	1.000000	Bq	0.000000	0.471298	-1.000000
Bq	2.083256	-0.148883	1.000000	Bq	2.083256	-0.148883	-1.000000
Bq	-2.083256	-0.148883	1.000000	Bq	-2.083256	-0.148883	-1.000000

13a_NICS0

`#p nmr=giao b3lyp/6-311+g(d,p)`

Charge = 0, Multiplicity = 1, Point group = C1

Electronic Energy = -671.277913 Hartree

	Cartesian Coordinates				Cartesian Coordinates		
	X	***Y***	***Z***		***X***	***Y***	***Z***
C	-2.299721	0.709815	-0.000001	C	-1.378957	-0.378124	-0.000001
C	-0.050904	0.207419	-0.000001	C	-0.233456	1.603415	-0.000001
N	-1.582212	1.888404	-0.000002	C	-3.683510	0.523517	-0.000001
C	-4.160243	-0.781754	0.000001	C	-3.275402	-1.874877	0.000003
C	-1.898294	-1.684669	0.000002	C	1.278655	-0.321845	-0.000001
C	2.370362	0.611664	0.000002	C	2.114570	2.018082	0.000002
C	0.839791	2.520938	0.000000	C	1.585244	-1.706350	-0.000003
C	2.891522	-2.150412	-0.000003	C	3.962215	-1.232331	0.000001
C	3.699809	0.120243	0.000003	H	-1.981350	2.814199	-0.000002
H	-4.360390	1.370182	-0.000002	H	-5.229740	-0.959077	0.000002
H	-3.674564	-2.882706	0.000005	H	-1.247187	-2.548709	0.000004
H	2.962122	2.694874	0.000004	H	0.654578	3.588797	0.000000
H	0.784372	-2.432525	-0.000007	H	3.096172	-3.215344	-0.000005
H	4.984844	-1.591876	0.000002	H	4.516532	0.834987	0.000005
Bq	-1.109050	0.806186	-0.000001	Bq	-2.782688	-0.581015	0.000000
Bq	1.053170	1.106612	0.000000	Bq	2.631301	-0.779838	0.000000

13a_NICS1

`#p nmr=giao b3lyp/6-311+g(d,p)`

Charge = 0, Multiplicity = 1, Point group = C1

Electronic Energy = -671.277913 Hartree

	Cartesian Coordinates				Cartesian Coordinates		
	X	***Y***	***Z***		***X***	***Y***	***Z***
C	-2.299721	0.709815	-0.000001	C	-1.378957	-0.378124	-0.000001

C	-0.050904	0.207419	-0.000001	C	-0.233456	1.603415	-0.000001
N	-1.582212	1.888404	-0.000002	C	-3.683510	0.523517	-0.000001
C	-4.160243	-0.781754	0.000001	C	-3.275402	-1.874877	0.000003
C	-1.898294	-1.684669	0.000002	C	1.278655	-0.321845	-0.000001
C	2.370362	0.611664	0.000002	C	2.114570	2.018082	0.000002
C	0.839791	2.520938	0.000000	C	1.585244	-1.706350	-0.000003
C	2.891522	-2.150412	-0.000003	C	3.962215	-1.232331	0.000001
C	3.699809	0.120243	0.000003	H	-1.981350	2.814199	-0.000002
H	-4.360390	1.370182	-0.000002	H	-5.229740	-0.959077	0.000002
H	-3.674564	-2.882706	0.000005	H	-1.247187	-2.548709	0.000004
H	2.962122	2.694874	0.000004	H	0.654578	3.588797	0.000000
H	0.784372	-2.432525	-0.000007	H	3.096172	-3.215344	-0.000005
H	4.984844	-1.591876	0.000002	H	4.516532	0.834987	0.000005
Bq	-1.109050	0.806185	-1.008646	Bq	-1.109050	0.806186	1.008644
Bq	-2.782687	-0.581014	1.000034	Bq	-2.782688	-0.581017	-1.000033
Bq	1.053171	1.106613	-1.000032	Bq	1.053168	1.106612	1.000032
Bq	2.631302	-0.779837	-1.000155	Bq	2.631300	-0.779840	1.000155

13b_NICS0

#p nmr=giao b3lyp/6-311+g(d,p)

Charge = 0, Multiplicity = 1, Point group = C1

Electronic Energy = -671.279239 Hartree

	Cartesian Coordinates				Cartesian Coordinates		
	X	***Y***	***Z***		***X***	***Y***	***Z***
C	1.992710	-0.887484	0.000000	C	1.914071	0.531207	0.000000
C	0.507697	0.864777	0.000000	C	-0.200334	-0.349244	0.000000
N	0.704576	-1.393712	0.000000	C	3.216828	-1.560081	0.000000
C	4.374774	-0.789368	0.000000	C	4.318226	0.617360	0.000000
C	3.097600	1.282588	0.000000	C	-0.198579	2.092677	0.000000
C	-1.570616	2.081591	0.000000	C	-2.308800	0.856072	0.000000
C	-1.622008	-0.402837	0.000000	C	-3.726766	0.847076	0.000000
C	-4.434275	-0.335517	0.000000	C	-3.751303	-1.571575	0.000000
C	-2.373340	-1.603885	0.000000	H	0.474080	-2.374762	0.000000
H	3.263591	-2.643016	0.000000	H	5.340068	-1.283022	0.000000
H	5.240909	1.186342	0.000000	H	3.063307	2.366541	0.000000
H	0.342649	3.032318	0.000000	H	-2.123916	3.014322	0.000000
H	-4.252223	1.796490	0.000000	H	-5.518286	-0.320312	0.000000
H	-4.313992	-2.498111	0.000000	H	-1.863531	-2.560946	0.000000

Bq	0.983744	-0.246891	0.000000	Bq	3.152368	-0.134296	0.000000
Bq	-0.898773	0.857173	0.000000	Bq	-3.036082	-0.368444	0.000000

13b_NICS1

```
#p nmr=giao b3lyp/6-311+g(d,p)
```

Charge = 0, Multiplicity = 1, Point group = C1

Electronic Energy = -671.279239 Hartree

	Cartesian Coordinates				Cartesian Coordinates		
	X	***Y***	***Z***		***X***	***Y***	***Z***
C	1.992710	-0.887484	0.000000	C	1.914071	0.531207	0.000000
C	0.507697	0.864777	0.000000	C	-0.200334	-0.349244	0.000000
N	0.704576	-1.393712	0.000000	C	3.216828	-1.560081	0.000000
C	4.374774	-0.789368	0.000000	C	4.318226	0.617360	0.000000
C	3.097600	1.282588	0.000000	C	-0.198579	2.092677	0.000000
C	-1.570616	2.081591	0.000000	C	-2.308800	0.856072	0.000000
C	-1.622008	-0.402837	0.000000	C	-3.726766	0.847076	0.000000
C	-4.434275	-0.335517	0.000000	C	-3.751303	-1.571575	0.000000
C	-2.373340	-1.603885	0.000000	H	0.474080	-2.374762	0.000000
H	3.263591	-2.643016	0.000000	H	5.340068	-1.283022	0.000000
H	5.240909	1.186342	0.000000	H	3.063307	2.366541	0.000000
H	0.342649	3.032318	0.000000	H	-2.123916	3.014322	0.000000
H	-4.252223	1.796490	0.000000	H	-5.518286	-0.320312	0.000000
H	-4.313992	-2.498111	0.000000	H	-1.863531	-2.560946	0.000000
Bq	0.983744	-0.246891	1.000000	Bq	0.983744	-0.246891	-1.000000
Bq	3.152368	-0.134296	1.000000	Bq	3.152368	-0.134296	-1.000000
Bq	-0.898773	0.857173	1.000000	Bq	-0.898773	0.857173	-1.000000
Bq	-3.036082	-0.368444	1.000000	Bq	-3.036082	-0.368444	-1.000000

13c_NICS0

```
#p nmr=giao b3lyp/6-311+g(d,p)
```

Charge = 0, Multiplicity = 1, Point group = C1

Electronic Energy = -671.276233 Hartree

	Cartesian Coordinates				Cartesian Coordinates		
	X	***Y***	***Z***		***X***	***Y***	***Z***
C	2.418963	-0.812389	0.000000	C	1.860395	0.491735	0.000000
C	0.416067	0.326850	0.000000	C	0.167709	-1.089478	0.000000
N	1.390062	-1.741461	0.000000	C	3.799025	-1.018203	0.000000

C	4.620704	0.106976	0.000000	C	4.085294	1.405535	0.000000
C	2.706690	1.604094	0.000000	C	-0.646212	1.207417	0.000000
C	-1.972809	0.712964	0.000000	C	-2.204624	-0.710367	0.000000
C	-1.108797	-1.607630	0.000000	C	-3.095833	1.588907	0.000000
C	-4.379594	1.100886	0.000000	C	-4.607784	-0.299445	0.000000
C	-3.550748	-1.176802	0.000000	H	1.513110	-2.741715	0.000000
H	4.217979	-2.017714	0.000000	H	5.696912	-0.025004	0.000001
H	4.753066	2.259109	0.000000	H	2.297479	2.608393	0.000000
H	-0.480998	2.279834	0.000000	H	-1.285212	-2.677523	0.000000
H	-2.917088	2.659446	0.000000	H	-5.222584	1.782587	0.000000
H	-5.624648	-0.676160	0.000001	H	-3.729129	-2.247312	0.000000
Bq	1.250639	-0.564949	0.000000	Bq	3.248512	0.296291	0.000000
Bq	-0.891444	-0.193374	0.000000	Bq	-3.301899	0.202691	0.000000

13c_NICS1

```
#p nmr=giao b3lyp/6-311+g(d,p)
```

Charge = 0, Multiplicity = 1, Point group = C1

Electronic Energy = -671.276233 Hartree

	**Cartesian Coordinates**				**Cartesian Coordinates**		
	X	***Y***	***Z***		***X***	***Y***	***Z***
C	2.418963	-0.812389	0.000000	C	1.860395	0.491735	0.000000
C	0.416067	0.326850	0.000000	C	0.167709	-1.089478	0.000000
N	1.390062	-1.741461	0.000000	C	3.799025	-1.018203	0.000000
C	4.620704	0.106976	0.000000	C	4.085294	1.405535	0.000000
C	2.706690	1.604094	0.000000	C	-0.646212	1.207417	0.000000
C	-1.972809	0.712964	0.000000	C	-2.204624	-0.710367	0.000000
C	-1.108797	-1.607630	0.000000	C	-3.095833	1.588907	0.000000
C	-4.379594	1.100886	0.000000	C	-4.607784	-0.299445	0.000000
C	-3.550748	-1.176802	0.000000	H	1.513110	-2.741715	0.000000
H	4.217979	-2.017714	0.000000	H	5.696912	-0.025004	0.000001
H	4.753066	2.259109	0.000000	H	2.297479	2.608393	0.000000
H	-0.480998	2.279834	0.000000	H	-1.285212	-2.677523	0.000000
H	-2.917088	2.659446	0.000000	H	-5.222584	1.782587	0.000000
H	-5.624648	-0.676160	0.000001	H	-3.729129	-2.247312	0.000000
Bq	1.250639	-0.564949	1.000000	Bq	1.250639	-0.564949	-1.000000
Bq	3.248512	0.296291	1.000000	Bq	3.248512	0.296291	-1.000000
Bq	-0.891444	-0.193374	1.000000	Bq	-0.891444	-0.193374	-1.000000
Bq	-3.301899	0.202691	1.000000	Bq	-3.301899	0.202691	-1.000000

13d_NICS0

`#p nmr=giao b3lyp/6-311+g(d,p)`

Charge = 0, Multiplicity = 1, Point group = C1

Electronic Energy = -788.030170 Hartree

	Cartesian Coordinates				Cartesian Coordinates		
	X	***Y***	***Z***		***X***	***Y***	***Z***
C	-0.948499	1.130291	-0.042402	C	-2.306556	0.719923	-0.027008
C	-2.303695	-0.726641	0.028933	C	-0.944030	-1.131680	0.042856
N	-0.131110	0.000905	-0.000053	C	-0.592504	2.479348	-0.113274
C	-1.619135	3.418370	-0.159730	C	-2.969971	3.029743	-0.138660
C	-3.319380	1.684622	-0.074785	C	-3.312663	-1.695330	0.077495
C	-2.957901	-3.039087	0.140588	C	-1.605527	-3.422417	0.160027
C	-0.582643	-2.479349	0.112776	C	1.296817	0.003253	-0.001051
C	1.999323	-0.540296	-1.077721	C	3.392363	-0.539624	-1.070913
C	4.113325	0.010964	-0.004602	C	3.391828	0.562156	1.061554
C	1.999228	0.553476	1.072746	C	5.622281	-0.011139	0.008291
H	0.445404	2.787703	-0.131951	H	-1.368780	4.471885	-0.214217
H	-3.744352	3.787173	-0.175337	H	-4.362533	1.388289	-0.064563
H	-4.356984	-1.403106	0.068385	H	-3.729266	-3.799562	0.177824
H	-1.350984	-4.474965	0.213799	H	0.456505	-2.783579	0.130049
H	1.456310	-0.954854	-1.919030	H	3.925594	-0.964083	-1.915036
H	3.924825	0.998508	1.899806	H	1.455971	0.971762	1.912057
H	6.028284	-0.026686	-1.005274	H	6.028304	0.858924	0.528887
H	5.993510	-0.903252	0.524231	Bq	-1.326778	-0.001440	0.000465
Bq	-1.959341	2.077050	-0.092643	Bq	-1.951077	-2.082417	0.093779
Bq	2.698814	0.008321	-0.003331				

13d_NICS1

`#p nmr=giao b3lyp/6-311+g(d,p)`

Charge = 0, Multiplicity = 1, Point group = C1

Electronic Energy = -788.030170 Hartree

	Cartesian Coordinates				Cartesian Coordinates		
	X	***Y***	***Z***		***X***	***Y***	***Z***
C	-0.948499	1.130291	-0.042402	C	-2.306556	0.719923	-0.027008
C	-2.303695	-0.726641	0.028933	C	-0.944030	-1.131680	0.042856
N	-0.131110	0.000905	-0.000053	C	-0.592504	2.479348	-0.113274

C	-1.619135	3.418370	-0.159730	C	-2.969971	3.029743	-0.138660
C	-3.319380	1.684622	-0.074785	C	-3.312663	-1.695330	0.077495
C	-2.957901	-3.039087	0.140588	C	-1.605527	-3.422417	0.160027
C	-0.582643	-2.479349	0.112776	C	1.296817	0.003253	-0.001051
C	1.999323	-0.540296	-1.077721	C	3.392363	-0.539624	-1.070913
C	4.113325	0.010964	-0.004602	C	3.391828	0.562156	1.061554
C	1.999228	0.553476	1.072746	C	5.622281	-0.011139	0.008291
H	0.445404	2.787703	-0.131951	H	-1.368780	4.471885	-0.214217
H	-3.744352	3.787173	-0.175337	H	-4.362533	1.388289	-0.064563
H	-4.356984	-1.403106	0.068385	H	-3.729266	-3.799562	0.177824
H	-1.350984	-4.474965	0.213799	H	0.456505	-2.783579	0.130049
H	1.456310	-0.954854	-1.919030	H	3.925594	-0.964083	-1.915036
H	3.924825	0.998508	1.899806	H	1.455971	0.971762	1.912057
H	6.028284	-0.026686	-1.005274	H	6.028304	0.858924	0.528887
H	5.993510	-0.903252	0.524231	Bq	-1.326365	0.036509	0.999745
Bq	-1.327191	-0.039390	-0.998814	Bq	-1.958721	2.027494	-1.091414
Bq	-1.959961	2.126605	0.906128	Bq	-1.949586	-2.033197	1.092566
Bq	-1.952567	-2.131637	-0.905008	Bq	2.702049	-0.881554	0.452860
Bq	2.695579	0.898197	-0.459522				

13e_NICS0

`#p nmr=giao b3lyp/6-311+g(d,p)`

Charge = 0, Multiplicity = 1, Point group = C1

Electronic Energy = -941.707276 Hartree

	Cartesian Coordinates				Cartesian Coordinates		
	X	***Y***	***Z***		***X***	***Y***	***Z***
C	-0.355905	1.583262	0.035134	C	1.067292	1.615033	0.037693
C	1.510858	0.235502	0.004991	C	0.347890	-0.555869	-0.016083
N	-0.776390	0.259202	0.002464	C	-1.130644	2.744368	0.079156
C	-0.467488	3.965421	0.117138	C	0.936670	4.026471	0.113065
C	1.703793	2.867669	0.075520	C	2.792020	-0.401562	-0.016492
C	2.825433	-1.836184	-0.064814	C	1.606953	-2.581140	-0.093360
C	0.381900	-1.966638	-0.070503	C	4.028347	0.293343	0.006835
C	5.230352	-0.383404	-0.015494	C	5.260849	-1.792961	-0.062761
C	4.078231	-2.499133	-0.086812	C	-2.135736	-0.184941	-0.001694
C	-2.937164	0.024685	-1.124753	C	-4.261800	-0.405333	-1.122167
C	-4.810804	-1.058687	-0.011788	C	-3.988563	-1.268352	1.101773
C	-2.665888	-0.831261	1.115373	C	-6.253666	-1.500714	-0.006723

H	-2.212500	2.694635	0.083164	H	-1.043574	4.883109	0.150884
H	1.429028	4.991709	0.141743	H	2.782305	2.950039	0.077828
H	1.666120	-3.663329	-0.135513	H	-0.534646	-2.542593	-0.094653
H	4.036193	1.373516	0.044068	H	6.160076	0.174327	0.003789
H	6.210868	-2.314569	-0.080127	H	4.089872	-3.583659	-0.123421
H	-2.521372	0.515990	-1.996698	H	-4.874138	-0.238811	-2.002153
H	-4.387468	-1.775409	1.973939	H	-2.044586	-0.990638	1.989020
H	-6.591282	-1.766831	-1.010680	H	-6.403376	-2.362195	0.647324
H	-6.904373	-0.697168	0.354830	Bq	0.358749	0.627426	0.012840
Bq	0.292286	2.800371	0.076284	Bq	1.577509	-1.184315	-0.042710
Bq	4.035872	-1.103317	-0.039923	Bq	-3.466659	-0.620648	-0.007209

13e_NICS1

#p nmr=giao b3lyp/6-311+g(d,p)

Charge = 0, Multiplicity = 1, Point group = C1

Electronic Energy = -941.707276 Hartree

	Cartesian Coordinates				Cartesian Coordinates		
	X	***Y***	***Z***		***X***	***Y***	***Z***
C	-0.355905	1.583262	0.035134	C	1.067292	1.615033	0.037693
C	1.510858	0.235502	0.004991	C	0.347890	-0.555869	-0.016083
N	-0.776390	0.259202	0.002464	C	-1.130644	2.744368	0.079156
C	-0.467488	3.965421	0.117138	C	0.936670	4.026471	0.113065
C	1.703793	2.867669	0.075520	C	2.792020	-0.401562	-0.016492
C	2.825433	-1.836184	-0.064814	C	1.606953	-2.581140	-0.093360
C	0.381900	-1.966638	-0.070503	C	4.028347	0.293343	0.006835
C	5.230352	-0.383404	-0.015494	C	5.260849	-1.792961	-0.062761
C	4.078231	-2.499133	-0.086812	C	-2.135736	-0.184941	-0.001694
C	-2.937164	0.024685	-1.124753	C	-4.261800	-0.405333	-1.122167
C	-4.810804	-1.058687	-0.011788	C	-3.988563	-1.268352	1.101773
C	-2.665888	-0.831261	1.115373	C	-6.253666	-1.500714	-0.006723
H	-2.212500	2.694635	0.083164	H	-1.043574	4.883109	0.150884
H	1.429028	4.991709	0.141743	H	2.782305	2.950039	0.077828
H	1.666120	-3.663329	-0.135513	H	-0.534646	-2.542593	-0.094653
H	4.036193	1.373516	0.044068	H	6.160076	0.174327	0.003789
H	6.210868	-2.314569	-0.080127	H	4.089872	-3.583659	-0.123421
H	-2.521372	0.515990	-1.996698	H	-4.874138	-0.238811	-2.002153
H	-4.387468	-1.775409	1.973939	H	-2.044586	-0.990638	1.989020

H	-6.591282	-1.766831	-1.010680	H	-6.403376	-2.362195	0.647324
H	-6.904373	-0.697168	0.354830	Bq	0.360078	0.651726	-0.986864
Bq	0.357420	0.603126	1.012544	Bq	0.294623	2.767686	1.075747
Bq	0.289950	2.833055	-0.923179	Bq	1.578463	-1.219565	0.956668
Bq	1.576555	-1.149065	-1.042088	Bq	4.036074	-1.069771	-1.039360
Bq	4.035670	-1.136863	0.959514	Bq	-3.177216	-1.501533	-0.381728
Bq	-3.756102	0.260237	0.367310				

13f_NICS0

```
#p nmr=giao b3lyp/6-311+g(d,p)
```

Charge = 0, Multiplicity = 1, Point group = C1

Electronic Energy = -941.704292 Hartree

	Cartesian Coordinates				Cartesian Coordinates		
	X	***Y***	***Z***		***X***	***Y***	***Z***
C	0.296480	2.002325	-0.000042	C	1.710170	2.077602	-0.000035
C	2.190940	0.719168	-0.000029	C	1.066803	-0.129288	-0.000033
N	-0.085416	0.662953	-0.000056	C	-0.503274	3.148428	-0.000038
C	0.139573	4.382140	0.000010	C	1.543986	4.476786	0.000045
C	2.333891	3.333097	0.000022	C	3.500868	0.185596	-0.000002
C	3.672669	-1.173018	0.000029	C	2.553751	-2.061379	0.000027
C	1.207795	-1.553694	0.000003	C	2.754452	-3.465350	0.000030
C	1.693818	-4.343077	0.000004	C	0.374255	-3.845193	-0.000007
C	0.137368	-2.487065	-0.000003	C	-1.460835	0.255614	-0.000027
C	-2.135922	0.075013	-1.206815	C	-3.476728	-0.302555	-1.201231
C	-4.169926	-0.497017	0.000030	C	-3.476702	-0.302488	1.201253
C	-2.135888	0.075083	1.206782	C	-5.630054	-0.877530	0.000044
H	-1.584207	3.080958	-0.000074	H	-0.455089	5.288670	0.000014
H	2.011803	5.454585	0.000102	H	3.415397	3.412900	0.000029
H	4.357231	0.850617	-0.000009	H	4.669094	-1.600760	0.000059
H	3.772799	-3.839881	0.000057	H	1.869723	-5.412783	-0.000007
H	-0.462385	-4.534670	-0.000017	H	-0.883066	-2.134326	0.000010
H	-1.608556	0.219511	-2.142597	H	-3.990393	-0.449381	-2.145433
H	-3.990338	-0.449253	2.145481	H	-1.608503	0.219627	2.142546
H	-5.888732	-1.461282	-0.885763	H	-5.888834	-1.460838	0.886113
H	-6.262975	0.016449	-0.000221	Bq	1.035795	1.066552	-0.000039
Bq	0.920138	3.236730	-0.000006	Bq	2.365471	-0.668769	-0.000001
Bq	1.453573	-2.959293	0.000009	Bq	-2.809334	-0.116058	-0.000001

13f_NICS1

```
#p nmr=giao b3lyp/6-311+g(d,p)
```

Charge = 0, Multiplicity = 1, Point group = C1

Electronic Energy = -941.704292 Hartree

	Cartesian Coordinates				Cartesian Coordinates		
	X	*Y*	*Z*		*X*	*Y*	*Z*
C	0.296480	2.002325	-0.000042	C	1.710170	2.077602	-0.000035
C	2.190940	0.719168	-0.000029	C	1.066803	-0.129288	-0.000033
N	-0.085416	0.662953	-0.000056	C	-0.503274	3.148428	-0.000038
C	0.139573	4.382140	0.000010	C	1.543986	4.476786	0.000045
C	2.333891	3.333097	0.000022	C	3.500868	0.185596	-0.000002
C	3.672669	-1.173018	0.000029	C	2.553751	-2.061379	0.000027
C	1.207795	-1.553694	0.000003	C	2.754452	-3.465350	0.000030
C	1.693818	-4.343077	0.000004	C	0.374255	-3.845193	-0.000007
C	0.137368	-2.487065	-0.000003	C	-1.460835	0.255614	-0.000027
C	-2.135922	0.075013	-1.206815	C	-3.476728	-0.302555	-1.201231
C	-4.169926	-0.497017	0.000030	C	-3.476702	-0.302488	1.201253
C	-2.135888	0.075083	1.206782	C	-5.630054	-0.877530	0.000044
H	-1.584207	3.080958	-0.000074	H	-0.455089	5.288670	0.000014
H	2.011803	5.454585	0.000102	H	3.415397	3.412900	0.000029
H	4.357231	0.850617	-0.000009	H	4.669094	-1.600760	0.000059
H	3.772799	-3.839881	0.000057	H	1.869723	-5.412783	-0.000007
H	-0.462385	-4.534670	-0.000017	H	-0.883066	-2.134326	0.000010
H	-1.608556	0.219511	-2.142597	H	-3.990393	-0.449381	-2.145433
H	-3.990338	-0.449253	2.145481	H	-1.608503	0.219627	2.142546
H	-5.888732	-1.461282	-0.885763	H	-5.888834	-1.460838	0.886113
H	-6.262975	0.016449	-0.000221	Bq	1.035805	1.066551	-1.000039
Bq	1.035785	1.066553	0.999961	Bq	0.920155	3.236758	-1.000006
Bq	0.920120	3.236701	0.999993	Bq	2.365485	-0.668789	-0.999999
Bq	2.365457	-0.668749	0.999998	Bq	1.453587	-2.959291	-0.999992
Bq	1.453560	-2.959295	1.000010	Bq	-2.551112	-1.041344	0.277791
Bq	-3.067555	0.809227	-0.277794				

13g_NICS0

```
#p nmr=giao b3lyp/6-311+g(d,p)
```

Charge = 0, Multiplicity = 1, Point group = C1

Electronic Energy = -941.705967 Hartree

	Cartesian Coordinates				Cartesian Coordinates		
	X	*Y*	*Z*		*X*	*Y*	*Z*
C	-0.771823	1.860872	0.037454	C	0.559099	2.346757	0.037237
C	1.429541	1.185206	0.018172	C	0.573476	0.032611	0.008484
N	-0.753815	0.463975	0.021128	C	-1.864730	2.728076	0.071049
C	-1.605274	4.097246	0.096134	C	-0.292795	4.595230	0.090042
C	0.793552	3.723979	0.062659	C	2.799377	1.023864	0.000476
C	3.356697	-0.277253	-0.029395	C	2.484693	-1.424240	-0.045755
C	1.078834	-1.248811	-0.027560	C	4.765098	-0.487056	-0.047387
C	5.295007	-1.753899	-0.080207	C	4.436740	-2.883429	-0.097315
C	3.072522	-2.721307	-0.080668	C	-1.903007	-0.383039	0.009860
C	-2.760638	-0.388676	-1.091463	C	-3.882924	-1.213840	-1.094931
C	-4.167859	-2.056893	-0.014117	C	-3.290120	-2.046703	1.076991
C	-2.173322	-1.214825	1.098039	C	-5.397709	-2.931539	-0.013246
H	-2.880769	2.353839	0.077532	H	-2.438206	4.790856	0.121696
H	-0.125926	5.665806	0.109116	H	1.806832	4.110195	0.063140
H	3.461030	1.883734	0.006204	H	0.428066	-2.114871	-0.040974
H	5.418143	0.379648	-0.034943	H	6.369702	-1.895580	-0.093518
H	4.863686	-3.879813	-0.123684	H	2.419072	-3.587562	-0.093758
H	-2.545147	0.245955	-1.943176	H	-4.540406	-1.209076	-1.957913
H	-3.484941	-2.692514	1.926775	H	-1.510879	-1.209162	1.955760
H	-5.698520	-3.195416	-1.029285	H	-5.229507	-3.853768	0.547050
H	-6.242142	-2.414059	0.454776	Bq	0.207296	1.177884	0.024495
Bq	-0.530329	3.225360	0.065763	Bq	1.953770	-0.118104	-0.012596
Bq	3.901793	-1.591197	-0.063454	Bq	-3.029645	-1.217329	-0.002604

13g_NICS1

`#p nmr=giao b3lyp/6-311+g(d,p)`

Charge = 0, Multiplicity = 1, Point group = C1

Electronic Energy = -941.705967 Hartree

	Cartesian Coordinates				Cartesian Coordinates		
	X	*Y*	*Z*		*X*	*Y*	*Z*
C	-0.771823	1.860872	0.037454	C	0.559099	2.346757	0.037237
C	1.429541	1.185206	0.018172	C	0.573476	0.032611	0.008484
N	-0.753815	0.463975	0.021128	C	-1.864730	2.728076	0.071049
C	-1.605274	4.097246	0.096134	C	-0.292795	4.595230	0.090042
C	0.793552	3.723979	0.062659	C	2.799377	1.023864	0.000476
C	3.356697	-0.277253	-0.029395	C	2.484693	-1.424240	-0.045755

C	1.078834	-1.248811	-0.027560	C	4.765098	-0.487056	-0.047387
C	5.295007	-1.753899	-0.080207	C	4.436740	-2.883429	-0.097315
C	3.072522	-2.721307	-0.080668	C	-1.903007	-0.383039	0.009860
C	-2.760638	-0.388676	-1.091463	C	-3.882924	-1.213840	-1.094931
C	-4.167859	-2.056893	-0.014117	C	-3.290120	-2.046703	1.076991
C	-2.173322	-1.214825	1.098039	C	-5.397709	-2.931539	-0.013246
H	-2.880769	2.353839	0.077532	H	-2.438206	4.790856	0.121696
H	-0.125926	5.665806	0.109116	H	1.806832	4.110195	0.063140
H	3.461030	1.883734	0.006204	H	0.428066	-2.114871	-0.040974
H	5.418143	0.379648	-0.034943	H	6.369702	-1.895580	-0.093518
H	4.863686	-3.879813	-0.123684	H	2.419072	-3.587562	-0.093758
H	-2.545147	0.245955	-1.943176	H	-4.540406	-1.209076	-1.957913
H	-3.484941	-2.692514	1.926775	H	-1.510879	-1.209162	1.955760
H	-5.698520	-3.195416	-1.029285	H	-5.229507	-3.853768	0.547050
H	-6.242142	-2.414059	0.454776	Bq	0.212464	1.165698	1.024407
Bq	0.202128	1.190070	-0.975417	Bq	-0.519641	3.204529	1.065488
Bq	-0.541016	3.246191	-0.933963	Bq	1.964805	-0.138434	0.987136
Bq	1.942735	-0.097774	-1.012329	Bq	3.911410	-1.613315	0.936255
Bq	3.892175	-1.569080	-1.063164	Bq	-2.487988	-1.943794	-0.425518
Bq	-3.571302	-0.490865	0.420311				

13h_NICS0

#p nmr=giao b3lyp/6-311+g(d,p)

Charge = 0, Multiplicity = 1, Point group = C1

Electronic Energy = -592.845199 Hartree

	Cartesian Coordinates				Cartesian Coordinates		
	X	***Y***	***Z***		***X***	***Y***	***Z***
C	0.627413	1.150514	-0.000001	C	0.432886	-0.253341	-0.000002
C	-0.998753	-0.472873	-0.000002	C	-1.607074	0.812614	-0.000001
N	-0.613735	1.772532	0.000000	C	1.907956	1.705423	0.000002
C	2.996941	0.842025	0.000010	C	2.814905	-0.552621	-0.000001
C	1.542247	-1.110874	-0.000016	C	-1.808055	-1.616123	0.000000
C	-3.190567	-1.466364	0.000003	C	-3.774902	-0.185952	0.000002
C	-2.996253	0.966666	0.000001	O	3.959475	-1.320757	-0.000029
H	-0.766234	2.768551	0.000001	H	2.057434	2.778732	0.000006
H	4.007042	1.234287	0.000025	H	1.414690	-2.188663	-0.000042
H	-1.364074	-2.605709	0.000001	H	-3.827851	-2.343121	0.000005
H	-4.855372	-0.094653	0.000004	H	-3.451067	1.950551	0.000001

H	3.725327	-2.256210	0.000266	Bq	-0.431853	0.601889	-0.000001
Bq	1.720391	0.296854	-0.000001	Bq	-2.395934	-0.327005	0.000000

13h_NICS1

`#p nmr=giao b3lyp/6-311+g(d,p)`

Charge = 0, Multiplicity = 1, Point group = C1

Electronic Energy = -592.845199 Hartree

	Cartesian Coordinates				Cartesian Coordinates		
	X	***Y***	***Z***		***X***	***Y***	***Z***
C	0.627413	1.150514	-0.000001	C	0.432886	-0.253341	-0.000002
C	-0.998753	-0.472873	-0.000002	C	-1.607074	0.812614	-0.000001
N	-0.613735	1.772532	0.000000	C	1.907956	1.705423	0.000002
C	2.996941	0.842025	0.000010	C	2.814905	-0.552621	-0.000001
C	1.542247	-1.110874	-0.000016	C	-1.808055	-1.616123	0.000000
C	-3.190567	-1.466364	0.000003	C	-3.774902	-0.185952	0.000002
C	-2.996253	0.966666	0.000001	O	3.959475	-1.320757	-0.000029
H	-0.766234	2.768551	0.000001	H	2.057434	2.778732	0.000006
H	4.007042	1.234287	0.000025	H	1.414690	-2.188663	-0.000042
H	-1.364074	-2.605709	0.000001	H	-3.827851	-2.343121	0.000005
H	-4.855372	-0.094653	0.000004	H	-3.451067	1.950551	0.000001
H	3.725327	-2.256210	0.000266	Bq	-0.431852	0.601888	0.998048
Bq	-0.431853	0.601890	-0.998051	Bq	1.720395	0.296860	-0.999971
Bq	1.720388	0.296849	0.999968	Bq	-2.395932	-0.327005	0.999972
Bq	-2.395936	-0.327006	-0.999971				

13i_NICS0

`#p nmr=giao b3lyp/6-311+g(d,p)`

Charge = 0, Multiplicity = 1, Point group = C1

Electronic Energy = -746.522719 Hartree

	Cartesian Coordinates				Cartesian Coordinates		
	X	***Y***	***Z***		***X***	***Y***	***Z***
C	-1.871249	-1.250567	0.000011	C	-1.144587	-0.028040	0.000006
C	0.260405	-0.384917	0.000003	C	0.311594	-1.793831	0.000004
N	-0.968027	-2.298517	0.000011	C	-3.265987	-1.283926	0.000011
C	-3.951965	-0.077722	0.000007	C	-3.252649	1.142521	-0.000008
C	-1.864091	1.181978	-0.000015	C	1.484240	0.357975	0.000004
C	2.715560	-0.381643	-0.000010	C	2.696500	-1.811634	-0.000016

C	1.523584	-2.519302	-0.000006	C	1.558397	1.773821	0.000020
C	2.773610	2.428039	0.000018	C	3.981031	1.700029	-0.000001
C	3.945185	0.322367	-0.000014	O	-4.021372	2.286046	-0.000041
H	-1.207534	-3.277715	0.000009	H	-3.804226	-2.224661	0.000019
H	-5.035213	-0.059120	0.000017	H	-1.362261	2.141244	-0.000041
H	3.644777	-2.338195	-0.000026	H	1.518797	-3.603162	-0.000007
H	0.649773	2.359514	0.000039	H	2.798967	3.512165	0.000033
H	4.930307	2.223385	-0.000004	H	4.868673	-0.247773	-0.000025
H	-3.452350	3.064693	0.000147	Bq	-0.682373	-1.151174	0.000007
Bq	-2.558421	-0.052626	0.000002	Bq	1.498647	-1.088892	-0.000003
Bq	2.743004	1.033431	0.000003				

13i_NICS1

`#p nmr=giao b3lyp/6-311+g(d,p)`

Charge = 0, Multiplicity = 1, Point group = C1

Electronic Energy = -746.522719 Hartree

	**Cartesian Coordinates**				**Cartesian Coordinates**		
	X	***Y***	***Z***		***X***	***Y***	***Z***
C	-1.871249	-1.250567	0.000011	C	-1.144587	-0.028040	0.000006
C	0.260405	-0.384917	0.000003	C	0.311594	-1.793831	0.000004
N	-0.968027	-2.298517	0.000011	C	-3.265987	-1.283926	0.000011
C	-3.951965	-0.077722	0.000007	C	-3.252649	1.142521	-0.000008
C	-1.864091	1.181978	-0.000015	C	1.484240	0.357975	0.000004
C	2.715560	-0.381643	-0.000010	C	2.696500	-1.811634	-0.000016
C	1.523584	-2.519302	-0.000006	C	1.558397	1.773821	0.000020
C	2.773610	2.428039	0.000018	C	3.981031	1.700029	-0.000001
C	3.945185	0.322367	-0.000014	O	-4.021372	2.286046	-0.000041
H	-1.207534	-3.277715	0.000009	H	-3.804226	-2.224661	0.000019
H	-5.035213	-0.059120	0.000017	H	-1.362261	2.141244	-0.000041
H	3.644777	-2.338195	-0.000026	H	1.518797	-3.603162	-0.000007
H	0.649773	2.359514	0.000039	H	2.798967	3.512165	0.000033
H	4.930307	2.223385	-0.000004	H	4.868673	-0.247773	-0.000025
H	-3.452350	3.064693	0.000147	Bq	-0.682369	-1.151172	1.000004
Bq	-0.682376	-1.151176	-0.999990	Bq	-2.558420	-0.052617	1.001858
Bq	-2.558422	-0.052635	-1.001854	Bq	1.498654	-1.088895	0.999995
Bq	1.498640	-1.088889	-1.000002	Bq	2.743012	1.033421	0.999989
Bq	2.742996	1.033442	-0.999983				

13j_NICS0

#p nmr=giao b3lyp/6-311+g(d,p)

Charge = 0, Multiplicity = 1, Point group = C1

Electronic Energy = -746.523651 Hartree

	Cartesian Coordinates				Cartesian Coordinates		
	X	*Y*	*Z*		*X*	*Y*	*Z*
C	1.564897	-1.073781	0.000003	C	1.572569	0.345366	0.000005
C	0.189533	0.762448	0.000003	C	-0.591107	-0.407995	0.000004
N	0.245412	-1.504188	0.000004	C	2.749683	-1.808107	0.000001
C	3.952135	-1.108497	-0.000021	C	3.971107	0.299318	-0.000002
C	2.792846	1.034653	0.000021	C	-0.441558	2.030782	0.000001
C	-1.811570	2.103255	0.000000	C	-2.622857	0.924182	0.000000
C	-2.013947	-0.373846	0.000002	C	-4.038628	1.000682	-0.000002
C	-4.816357	-0.137268	-0.000003	C	-4.209573	-1.412272	-0.000002
C	-2.835840	-1.527375	0.000000	O	5.156891	1.002197	-0.000072
H	-0.044892	-2.468831	0.000007	H	2.744348	-2.891847	0.000001
H	4.889533	-1.654973	-0.000052	H	2.834896	2.117620	0.000050
H	0.155648	2.935960	-0.000001	H	-2.307461	3.067698	-0.000002
H	-4.505871	1.980086	-0.000003	H	-5.897486	-0.056545	-0.000005
H	-4.827199	-2.303120	-0.000004	H	-2.383954	-2.513301	0.000000
H	5.901422	0.389720	0.000499	Bq	0.596261	-0.375630	0.000004
Bq	2.767206	-0.385175	0.000001	Bq	-1.215251	0.839804	0.000002
Bq	-3.422867	-0.254316	-0.000001				

13j_NICS1

#p nmr=giao b3lyp/6-311+g(d,p)

Charge = 0, Multiplicity = 1, Point group = C1

Electronic Energy = -746.523651 Hartree

	Cartesian Coordinates				Cartesian Coordinates		
	X	*Y*	*Z*		*X*	*Y*	*Z*
C	1.564897	-1.073781	0.000003	C	1.572569	0.345366	0.000005
C	0.189533	0.762448	0.000003	C	-0.591107	-0.407995	0.000004
N	0.245412	-1.504188	0.000004	C	2.749683	-1.808107	0.000001
C	3.952135	-1.108497	-0.000021	C	3.971107	0.299318	-0.000002
C	2.792846	1.034653	0.000021	C	-0.441558	2.030782	0.000001
C	-1.811570	2.103255	0.000000	C	-2.622857	0.924182	0.000000
C	-2.013947	-0.373846	0.000002	C	-4.038628	1.000682	-0.000002

C	-4.816357	-0.137268	-0.000003	C	-4.209573	-1.412272	-0.000002
C	-2.835840	-1.527375	0.000000	O	5.156891	1.002197	-0.000072
H	-0.044892	-2.468831	0.000007	H	2.744348	-2.891847	0.000001
H	4.889533	-1.654973	-0.000052	H	2.834896	2.117620	0.000050
H	0.155648	2.935960	-0.000001	H	-2.307461	3.067698	-0.000002
H	-4.505871	1.980086	-0.000003	H	-5.897486	-0.056545	-0.000005
H	-4.827199	-2.303120	-0.000004	H	-2.383954	-2.513301	0.000000
H	5.901422	0.389720	0.000499	Bq	0.596261	-0.375630	-1.001988
Bq	0.596261	-0.375630	1.001996	Bq	2.767212	-0.385182	1.000001
Bq	2.767200	-0.385167	-0.999999	Bq	-1.215250	0.839803	-1.000044
Bq	-1.215252	0.839805	1.000048	Bq	-3.422865	-0.254316	-0.999763
Bq	-3.422869	-0.254316	0.999761				

13k_NICS0

`#p nmr=giao b3lyp/6-311+g(d,p)`

Charge = 0, Multiplicity = 1, Point group = C1

Electronic Energy = -746.520630 Hartree

	Cartesian Coordinates				Cartesian Coordinates		
	X	***Y***	***Z***		***X***	***Y***	***Z***
C	-1.990727	1.182088	0.000000	C	-1.560288	-0.165793	-0.000002
C	-0.108310	-0.140336	-0.000002	C	0.275832	1.246494	-0.000001
N	-0.875201	2.013113	-0.000002	C	-3.346961	1.506012	0.000002
C	-4.271092	0.465733	-0.000001	C	-3.852661	-0.875019	0.000001
C	-2.500424	-1.202374	0.000004	C	0.862623	-1.121177	-0.000001
C	2.230605	-0.759682	0.000000	C	2.600384	0.634662	-0.000001
C	1.598741	1.635117	-0.000001	C	3.262316	-1.741867	0.000001
C	4.587556	-1.382424	0.000002	C	4.951826	-0.010792	0.000001
C	3.986368	0.966089	0.000000	O	-4.844555	-1.831365	0.000014
H	-0.901902	3.020450	0.000002	H	-3.681279	2.536714	0.000001
H	-5.333524	0.678983	-0.000005	H	-2.185609	-2.240913	0.000009
H	0.592490	-2.172106	-0.000001	H	1.880092	2.682527	-0.000002
H	2.978966	-2.789648	0.000001	H	5.359740	-2.143403	0.000003
H	6.000817	0.264180	0.000002	H	4.269369	2.013795	0.000000
H	-4.451046	-2.711846	-0.000119	Bq	-0.851739	0.827113	-0.000001
Bq	-2.920359	0.151775	0.000001	Bq	1.243313	0.249180	-0.000001
Bq	3.603176	-0.382336	0.000000				

13k_NICS1

```
#p nmr=giao b3lyp/6-311+g(d,p)
```

Charge = 0, Multiplicity = 1, Point group = C1

Electronic Energy = -746.520630 Hartree

	Cartesian Coordinates				Cartesian Coordinates		
	X	*Y*	*Z*		*X*	*Y*	*Z*
C	-1.990727	1.182088	0.000000	C	-1.560288	-0.165793	-0.000002
C	-0.108310	-0.140336	-0.000002	C	0.275832	1.246494	-0.000001
N	-0.875201	2.013113	-0.000002	C	-3.346961	1.506012	0.000002
C	-4.271092	0.465733	-0.000001	C	-3.852661	-0.875019	0.000001
C	-2.500424	-1.202374	0.000004	C	0.862623	-1.121177	-0.000001
C	2.230605	-0.759682	0.000000	C	2.600384	0.634662	-0.000001
C	1.598741	1.635117	-0.000001	C	3.262316	-1.741867	0.000001
C	4.587556	-1.382424	0.000002	C	4.951826	-0.010792	0.000001
C	3.986368	0.966089	0.000000	O	-4.844555	-1.831365	0.000014
H	-0.901902	3.020450	0.000002	H	-3.681279	2.536714	0.000001
H	-5.333524	0.678983	-0.000005	H	-2.185609	-2.240913	0.000009
H	0.592490	-2.172106	-0.000001	H	1.880092	2.682527	-0.000002
H	2.978966	-2.789648	0.000001	H	5.359740	-2.143403	0.000003
H	6.000817	0.264180	0.000002	H	4.269369	2.013795	0.000000
H	-4.451046	-2.711846	-0.000119	Bq	-0.851738	0.827113	0.999704
Bq	-0.851739	0.827114	-0.999707	Bq	-2.920359	0.151775	0.990460
Bq	-2.920359	0.151774	-0.990459	Bq	1.243313	0.249180	-0.998251
Bq	1.243312	0.249180	0.998249	Bq	3.603177	-0.382336	-1.002146
Bq	3.603175	-0.382335	1.002147				

13l_NICS0

```
#p nmr=giao b3lyp/6-311+g(d,p)
```

Charge = 0, Multiplicity = 1, Point group = C1

Electronic Energy = -863.274969 Hartree

	Cartesian Coordinates				Cartesian Coordinates		
	X	*Y*	*Z*		*X*	*Y*	*Z*
C	0.891856	-0.742138	-0.024327	C	2.136217	-0.068383	-0.004139
C	1.842372	1.347121	0.039567	C	0.427584	1.470983	0.042917
N	-0.141324	0.201181	0.003761	C	0.826424	-2.135058	-0.090020
C	2.018956	-2.848392	-0.123131	C	3.259581	-2.187669	-0.094211
C	3.330584	-0.800737	-0.038178	C	2.637266	2.498674	0.086341
C	2.021433	3.744415	0.139013	C	0.618913	3.849286	0.150202

C	-0.194681	2.721359	0.103720	C	-1.537761	-0.090809	-0.000868
C	-2.339374	0.322859	-1.066830	C	-3.703123	0.040289	-1.062943
C	-4.295292	-0.669655	-0.011270	C	-3.474347	-1.087834	1.042875
C	-2.112190	-0.796854	1.057838	C	-5.777607	-0.952912	-0.002712
O	4.381779	-2.985897	-0.127632	H	-0.122446	-2.655933	-0.115055
H	2.007019	-3.930750	-0.172415	H	4.292390	-0.298273	-0.023516
H	3.719000	2.421012	0.084220	H	2.624907	4.643950	0.174924
H	0.158913	4.830008	0.196859	H	-1.273617	2.812780	0.114707
H	-1.893871	0.856985	-1.897929	H	-4.313310	0.367014	-1.898487
H	-3.905703	-1.642493	1.869567	H	-1.494265	-1.114960	1.889413
H	-6.166708	-1.064542	-1.017105	H	-6.005279	-1.863186	0.555851
H	-6.327749	-0.131975	0.469867	H	5.174894	-2.438004	-0.099241
Bq	1.031341	0.441753	0.011556	Bq	2.077270	-1.463730	-0.062334
Bq	1.225481	2.605306	0.093627	Bq	-2.910348	-0.380334	-0.006866

13l_NICS1

#p nmr=giao b3lyp/6-311+g(d,p)

Charge = 0, Multiplicity = 1, Point group = C1

Electronic Energy = -863.274969 Hartree

	Cartesian Coordinates				Cartesian Coordinates		
	X	***Y***	***Z***		***X***	***Y***	***Z***
C	0.891856	-0.742138	-0.024327	C	2.136217	-0.068383	-0.004139
C	1.842372	1.347121	0.039567	C	0.427584	1.470983	0.042917
N	-0.141324	0.201181	0.003761	C	0.826424	-2.135058	-0.090020
C	2.018956	-2.848392	-0.123131	C	3.259581	-2.187669	-0.094211
C	3.330584	-0.800737	-0.038178	C	2.637266	2.498674	0.086341
C	2.021433	3.744415	0.139013	C	0.618913	3.849286	0.150202
C	-0.194681	2.721359	0.103720	C	-1.537761	-0.090809	-0.000868
C	-2.339374	0.322859	-1.066830	C	-3.703123	0.040289	-1.062943
C	-4.295292	-0.669655	-0.011270	C	-3.474347	-1.087834	1.042875
C	-2.112190	-0.796854	1.057838	C	-5.777607	-0.952912	-0.002712
O	4.381779	-2.985897	-0.127632	H	-0.122446	-2.655933	-0.115055
H	2.007019	-3.930750	-0.172415	H	4.292390	-0.298273	-0.023516
H	3.719000	2.421012	0.084220	H	2.624907	4.643950	0.174924
H	0.158913	4.830008	0.196859	H	-1.273617	2.812780	0.114707
H	-1.893871	0.856985	-1.897929	H	-4.313310	0.367014	-1.898487
H	-3.905703	-1.642493	1.869567	H	-1.494265	-1.114960	1.889413
H	-6.166708	-1.064542	-1.017105	H	-6.005279	-1.863186	0.555851

H	-6.327749	-0.131975	0.469867	H	5.174894	-2.438004	-0.099241
Bq	1.031532	0.472306	-0.987977	Bq	1.031150	0.411200	1.011089
Bq	2.079984	-1.506939	0.936728	Bq	2.074555	-1.420520	-1.061397
Bq	1.227836	2.561880	1.092681	Bq	1.223127	2.648732	-0.905427
Bq	-2.727852	-1.240293	-0.483486	Bq	-3.092843	0.479625	0.469753

3a_NICS0

```
#p nmr=giao b3lyp/6-311+g(d,p)
```

Charge = 0, Multiplicity = 1, Point group = C1

Electronic Energy = -1016.952281 Hartree

	**Cartesian Coordinates**				**Cartesian Coordinates**		
	X	***Y***	***Z***		***X***	***Y***	***Z***
C	-0.333701	1.359686	0.022113	C	1.084739	1.287985	0.022022
C	1.426764	-0.118703	-0.000947	C	0.207615	-0.824771	-0.013844
N	-0.853122	0.066767	0.000980	C	-1.011704	2.578694	0.061386
C	-0.264211	3.747709	0.090001	C	1.140603	3.698158	0.081577
C	1.820622	2.487148	0.050546	C	2.659297	-0.846652	-0.021851
C	2.589148	-2.280157	-0.061581	C	1.319501	-2.935547	-0.083111
C	0.141958	-2.235299	-0.060757	C	3.942977	-0.243960	-0.006328
C	5.093039	-1.006435	-0.028065	C	5.021306	-2.414239	-0.066767
C	3.789898	-3.032462	-0.083019	C	-2.240818	-0.274086	-0.001445
C	-3.034455	0.028663	-1.109123	C	-4.387625	-0.300402	-1.104795
C	-4.974911	-0.944731	-0.008825	C	-4.161095	-1.249288	1.088817
C	-2.809468	-0.912126	1.101703	C	-6.446812	-1.277487	-0.001646
O	1.795708	4.909358	0.108292	H	-2.093732	2.618776	0.069253
H	-0.753807	4.713676	0.119380	H	2.902963	2.489663	0.050506
H	1.300864	-4.019366	-0.119634	H	-0.813094	-2.744981	-0.080135
H	4.031684	0.832716	0.024408	H	6.060644	-0.517141	-0.014882
H	5.930750	-3.003727	-0.083752	H	3.722324	-4.115200	-0.113120
H	-2.590658	0.512486	-1.971391	H	-4.993040	-0.062039	-1.972975
H	-4.589401	-1.751476	1.949833	H	-2.195390	-1.142788	1.964442
H	-6.811667	-1.487061	-1.009485	H	-6.655357	-2.144819	0.628090
H	-7.032560	-0.438962	0.390310	H	2.749673	4.767814	0.099592
Bq	0.306459	0.354193	0.006065	Bq	0.406058	2.526563	0.054607
Bq	1.390714	-1.540188	-0.040349	Bq	3.849277	-1.637317	-0.044602
Bq	-3.601395	-0.608662	-0.005611				

3a_NICS1

```
#p nmr=giao b3lyp/6-311+g(d,p)
```

Charge = 0, Multiplicity = 1, Point group = C1

Electronic Energy = -1016.952281 Hartree

	Cartesian Coordinates				Cartesian Coordinates		
	X	***Y***	***Z***		***X***	***Y***	***Z***
C	-0.333701	1.359686	0.022113	C	1.084739	1.287985	0.022022
C	1.426764	-0.118703	-0.000947	C	0.207615	-0.824771	-0.013844
N	-0.853122	0.066767	0.000980	C	-1.011704	2.578694	0.061386
C	-0.264211	3.747709	0.090001	C	1.140603	3.698158	0.081577
C	1.820622	2.487148	0.050546	C	2.659297	-0.846652	-0.021851
C	2.589148	-2.280157	-0.061581	C	1.319501	-2.935547	-0.083111
C	0.141958	-2.235299	-0.060757	C	3.942977	-0.243960	-0.006328
C	5.093039	-1.006435	-0.028065	C	5.021306	-2.414239	-0.066767
C	3.789898	-3.032462	-0.083019	C	-2.240818	-0.274086	-0.001445
C	-3.034455	0.028663	-1.109123	C	-4.387625	-0.300402	-1.104795
C	-4.974911	-0.944731	-0.008825	C	-4.161095	-1.249288	1.088817
C	-2.809468	-0.912126	1.101703	C	-6.446812	-1.277487	-0.001646
O	1.795708	4.909358	0.108292	H	-2.093732	2.618776	0.069253
H	-0.753807	4.713676	0.119380	H	2.902963	2.489663	0.050506
H	1.300864	-4.019366	-0.119634	H	-0.813094	-2.744981	-0.080135
H	4.031684	0.832716	0.024408	H	6.060644	-0.517141	-0.014882
H	5.930750	-3.003727	-0.083752	H	3.722324	-4.115200	-0.113120
H	-2.590658	0.512486	-1.971391	H	-4.993040	-0.062039	-1.972975
H	-4.589401	-1.751476	1.949833	H	-2.195390	-1.142788	1.964442
H	-6.811667	-1.487061	-1.009485	H	-6.655357	-2.144819	0.628090
H	-7.032560	-0.438962	0.390310	H	2.749673	4.767814	0.099592
Bq	0.307039	0.370754	-0.993798	Bq	0.305879	0.337631	1.005927
Bq	0.408656	2.499952	1.054250	Bq	0.403460	2.553175	-0.945035
Bq	1.392091	-1.569818	0.959212	Bq	1.389336	-1.510559	-1.039909
Bq	3.849955	-1.664881	0.955018	Bq	3.848600	-1.609754	-1.044222
Bq	-3.382068	-1.496921	-0.409209	Bq	-3.820723	0.279598	0.397986

13m_NICS0

```
#p nmr=giao b3lyp/6-311+g(d,p)
```

Charge = 0, Multiplicity = 1, Point group = C1

Electronic Energy = -1170.624718 Hartree

	Cartesian Coordinates				Cartesian Coordinates		

	X	Y	Z		X	Y	Z
C	-0.465042	1.803168	0.003114	C	0.949748	1.860111	0.002229
C	1.407214	0.487797	0.001649	C	0.268508	-0.330646	0.002486
N	-0.868858	0.471733	0.003149	C	-1.260124	2.950745	0.002595
C	-0.630524	4.185959	0.001784	C	0.773069	4.267840	0.001024
C	1.566350	3.128164	0.001053	C	2.726500	-0.092034	-0.000275
C	2.852632	-1.513127	-0.002433	C	1.655001	-2.355531	0.000344
C	0.350171	-1.771137	0.003910	C	1.747412	-3.765782	0.000963
C	0.628255	-4.575824	0.005616	C	-0.648433	-3.997996	0.010307
C	-0.780038	-2.623436	0.009422	C	-2.257914	0.110546	-0.000192
C	-2.936091	-0.043878	-1.208387	C	-4.291742	-0.365359	-1.205752
C	-4.996171	-0.526418	-0.006434	C	-4.299250	-0.355404	1.196643
C	-2.944255	-0.034136	1.205342	C	-6.470645	-0.846663	-0.007698
O	1.310293	5.535855	0.000031	C	3.903548	0.692506	-0.000621
C	5.159512	0.116985	-0.003972	C	5.286390	-1.279155	-0.007272
C	4.151920	-2.069048	-0.006396	H	-2.340714	2.883673	0.003047
H	-1.209522	5.101535	0.001543	H	2.641555	3.245281	-0.000360
H	2.718088	-4.241370	-0.001859	H	0.742051	-5.653756	0.005921
H	-1.533370	-4.623873	0.014870	H	-1.771177	-2.199561	0.013328
H	-2.400455	0.075585	-2.142955	H	-4.808011	-0.493239	-2.151214
H	-4.821689	-0.475827	2.139785	H	-2.414412	0.092999	2.142206
H	-6.766070	-1.357302	-0.926301	H	-6.740456	-1.478763	0.841121
H	-7.065867	0.069871	0.065674	H	2.273256	5.484197	0.000094
H	3.829921	1.768718	0.001930	H	6.042064	0.746662	-0.004160
H	6.266998	-1.740820	-0.010319	H	4.281461	-3.142471	-0.008935
Bq	0.258314	0.858433	0.002525	Bq	0.155580	3.032665	0.001966
Bq	1.543338	-0.929113	0.000947	Bq	0.492061	-3.181618	0.005094
Bq	4.013417	-0.690646	-0.003495	Bq	-3.620904	-0.202441	-0.003130

13m_NICS1

#p nmr=giao b3lyp/6-311+g(d,p)

Charge = 0, Multiplicity = 1, Point group = C1

Electronic Energy = -1170.624718 Hartree

	Cartesian Coordinates				Cartesian Coordinates		
	X	Y	Z		X	Y	Z
C	-0.465042	1.803168	0.003114	C	0.949748	1.860111	0.002229
C	1.407214	0.487797	0.001649	C	0.268508	-0.330646	0.002486

N	-0.868858	0.471733	0.003149	C	-1.260124	2.950745	0.002595
C	-0.630524	4.185959	0.001784	C	0.773069	4.267840	0.001024
C	1.566350	3.128164	0.001053	C	2.726500	-0.092034	-0.000275
C	2.852632	-1.513127	-0.002433	C	1.655001	-2.355531	0.000344
C	0.350171	-1.771137	0.003910	C	1.747412	-3.765782	0.000963
C	0.628255	-4.575824	0.005616	C	-0.648433	-3.997996	0.010307
C	-0.780038	-2.623436	0.009422	C	-2.257914	0.110546	-0.000192
C	-2.936091	-0.043878	-1.208387	C	-4.291742	-0.365359	-1.205752
C	-4.996171	-0.526418	-0.006434	C	-4.299250	-0.355404	1.196643
C	-2.944255	-0.034136	1.205342	C	-6.470645	-0.846663	-0.007698
O	1.310293	5.535855	0.000031	C	3.903548	0.692506	-0.000621
C	5.159512	0.116985	-0.003972	C	5.286390	-1.279155	-0.007272
C	4.151920	-2.069048	-0.006396	H	-2.340714	2.883673	0.003047
H	-1.209522	5.101535	0.001543	H	2.641555	3.245281	-0.000360
H	2.718088	-4.241370	-0.001859	H	0.742051	-5.653756	0.005921
H	-1.533370	-4.623873	0.014870	H	-1.771177	-2.199561	0.013328
H	-2.400455	0.075585	-2.142955	H	-4.808011	-0.493239	-2.151214
H	-4.821689	-0.475827	2.139785	H	-2.414412	0.092999	2.142206
H	-6.766070	-1.357302	-0.926301	H	-6.740456	-1.478763	0.841121
H	-7.065867	0.069871	0.065674	H	2.273256	5.484197	0.000094
H	3.829921	1.768718	0.001930	H	6.042064	0.746662	-0.004160
H	6.266998	-1.740820	-0.010319	H	4.281461	-3.142471	-0.008935
Bq	0.258966	0.858323	1.002525	Bq	0.257662	0.858542	-0.997474
Bq	0.156105	3.033231	1.001966	Bq	0.155054	3.032098	-0.998033
Bq	1.545204	-0.929348	1.000945	Bq	1.541472	-0.928878	-0.999051
Bq	0.495776	-3.180686	1.005086	Bq	0.488346	-3.182550	-0.994899
Bq	4.015341	-0.692510	0.996502	Bq	4.011493	-0.688781	-1.003491
Bq	-3.392970	-1.176091	0.004168	Bq	-3.848838	0.771208	-0.010428

13n_NICS0

```
#p nmr=giao b3lyp/6-311+g(d,p)
```

Charge = 0, Multiplicity = 1, Point group = C1

Electronic Energy = -1092.192570 Hartree

	Cartesian Coordinates				Cartesian Coordinates		
	X	*Y*	*Z*		*X*	*Y*	*Z*
C	-0.809287	1.642740	-0.029081	C	0.504206	2.155995	0.015537
C	1.405175	1.019386	0.015692	C	0.579390	-0.152849	-0.030034
N	-0.763649	0.243498	-0.061626	C	-1.916465	2.491439	-0.020816

C	-1.691234	3.863477	0.020643	C	-0.386760	4.383096	0.055907
C	0.718478	3.538354	0.055957	C	2.782172	0.919350	0.034426
C	3.382048	-0.358456	0.008294	C	2.559595	-1.539312	-0.045967
C	1.144020	-1.418599	-0.072460	C	4.797558	-0.520038	0.027976
C	5.375733	-1.764824	-0.003385	C	4.561663	-2.925031	-0.056893
C	3.192084	-2.814990	-0.078136	C	-1.918518	-0.599237	-0.022828
C	-2.731826	-0.724903	-1.153295	C	-3.868056	-1.526444	-1.106186
C	-4.215877	-2.227969	0.056348	C	-3.394393	-2.088989	1.180355
C	-2.262050	-1.275780	1.151560	C	-5.428658	-3.124531	0.084399
O	-0.269773	5.754439	0.092624	O	0.427223	-2.585164	-0.151257
H	-2.926950	2.103317	-0.046589	H	-2.526522	4.553634	0.026640
H	1.723625	3.945797	0.089521	H	3.406756	1.805063	0.064152
H	5.416403	0.370444	0.068415	H	6.455118	-1.865124	0.012387
H	5.025256	-3.904891	-0.080948	H	2.574125	-3.702645	-0.119031
H	-2.468026	-0.197178	-2.062249	H	-4.490866	-1.613875	-1.990070
H	-3.646728	-2.611211	2.096849	H	-1.648280	-1.158921	2.037486
H	-6.245289	-2.706026	-0.508050	H	-5.192098	-4.108097	-0.335278
H	-5.785408	-3.278838	1.104432	H	0.661577	6.003653	0.113678
H	-0.520735	-2.405084	-0.098981	Bq	0.183167	0.981754	-0.017902
Bq	-0.596844	3.012517	0.016358	Bq	1.975400	-0.255080	-0.015008
Bq	3.978114	-1.653775	-0.024685	Bq	-3.065120	-1.407220	0.017659

13n_NICS1

`#p nmr=giao b3lyp/6-311+g(d,p)`

Charge = 0, Multiplicity = 1, Point group = C1

Electronic Energy = -1092.192570 Hartree

	Cartesian Coordinates				Cartesian Coordinates		
	X	***Y***	***Z***		***X***	***Y***	***Z***
C	-0.809287	1.642740	-0.029081	C	0.504206	2.155995	0.015537
C	1.405175	1.019386	0.015692	C	0.579390	-0.152849	-0.030034
N	-0.763649	0.243498	-0.061626	C	-1.916465	2.491439	-0.020816
C	-1.691234	3.863477	0.020643	C	-0.386760	4.383096	0.055907
C	0.718478	3.538354	0.055957	C	2.782172	0.919350	0.034426
C	3.382048	-0.358456	0.008294	C	2.559595	-1.539312	-0.045967
C	1.144020	-1.418599	-0.072460	C	4.797558	-0.520038	0.027976
C	5.375733	-1.764824	-0.003385	C	4.561663	-2.925031	-0.056893
C	3.192084	-2.814990	-0.078136	C	-1.918518	-0.599237	-0.022828
C	-2.731826	-0.724903	-1.153295	C	-3.868056	-1.526444	-1.106186

C	-4.215877	-2.227969	0.056348	C	-3.394393	-2.088989	1.180355
C	-2.262050	-1.275780	1.151560	C	-5.428658	-3.124531	0.084399
O	-0.269773	5.754439	0.092624	O	0.427223	-2.585164	-0.151257
H	-2.926950	2.103317	-0.046589	H	-2.526522	4.553634	0.026640
H	1.723625	3.945797	0.089521	H	3.406756	1.805063	0.064152
H	5.416403	0.370444	0.068415	H	6.455118	-1.865124	0.012387
H	5.025256	-3.904891	-0.080948	H	2.574125	-3.702645	-0.119031
H	-2.468026	-0.197178	-2.062249	H	-4.490866	-1.613875	-1.990070
H	-3.646728	-2.611211	2.096849	H	-1.648280	-1.158921	2.037486
H	-6.245289	-2.706026	-0.508050	H	-5.192098	-4.108097	-0.335278
H	-5.785408	-3.278838	1.104432	H	0.661577	6.003653	0.113678
H	-0.520735	-2.405084	-0.098981	Bq	0.210420	1.003316	-1.017298
Bq	0.155914	0.960192	0.981494	Bq	-0.578232	3.040177	-0.983086
Bq	-0.615456	2.984857	1.015802	Bq	1.992388	-0.222370	-1.014329
Bq	1.958412	-0.287790	0.984312	Bq	3.995968	-1.620128	-1.023959
Bq	3.960259	-1.687422	0.974589	Bq	-2.520098	-2.189461	-0.284089
Bq	-3.610142	-0.624980	0.319407				

10c_NICS0

`#p nmr=giao b3lyp/6-311+g(d,p)`

Charge = 0, Multiplicity = 1, Point group = C1

Electronic Energy = -1056.261364 Hartree

	Cartesian Coordinates				Cartesian Coordinates		
	X	***Y***	***Z***		***X***	***Y***	***Z***
C	-0.303196	1.194281	0.012889	C	1.085115	0.906828	0.011359
C	1.207067	-0.536057	-0.006345	C	-0.106253	-1.046929	-0.014243
N	-1.016660	-0.002164	-0.001926	C	-0.784603	2.505424	0.048899
C	0.134946	3.540360	0.072416	C	1.521380	3.279827	0.062593
C	2.004467	1.975926	0.034847	C	2.312418	-1.446071	-0.026380
C	2.021915	-2.851942	-0.058977	C	0.666511	-3.304287	-0.075559
C	-0.388725	-2.430520	-0.054993	C	3.673934	-1.049096	-0.016946
C	4.692679	-1.979785	-0.037351	C	4.404411	-3.359781	-0.068424
C	3.092283	-3.780444	-0.078944	C	-2.440179	-0.124699	-0.001286
C	-3.180794	0.302271	-1.104882	C	-4.568450	0.185330	-1.097632
C	-5.245245	-0.366828	-0.002894	C	-4.485093	-0.798577	1.090511
C	-3.097653	-0.673289	1.100623	C	-6.750832	-0.469132	0.007505
O	2.318652	4.396538	0.084980	C	3.734190	4.220938	0.081063
H	-1.847220	2.713601	0.058391	H	-0.196655	4.571431	0.099359

H	3.067008	1.791248	0.033621	H	0.481091	-4.372490	-0.107075
H	-1.411060	-2.786725	-0.070987	H	3.927710	0.001088	0.007794
H	5.724193	-1.645471	-0.029037	H	5.211841	-4.082784	-0.084259
H	2.858367	-4.839894	-0.103484	H	-2.670151	0.716329	-1.966479
H	-5.131985	0.518798	-1.962688	H	-4.983225	-1.233097	1.950770
H	-2.524424	-0.999734	1.960520	H	-7.145679	-0.623481	-0.999046
H	-7.089157	-1.292012	0.640508	H	-7.199907	0.450858	0.397121
H	4.158803	5.222912	0.100753	H	4.068336	3.667237	0.964148
H	4.067762	3.702540	-0.823391	Bq	0.173215	0.103192	0.000347
Bq	0.609685	2.233774	0.040500	Bq	0.952155	-1.935968	-0.039416
Bq	3.366273	-2.411186	-0.047837	Bq	-3.836236	-0.245965	-0.002593

10c_NICS1

`#p nmr=giao b3lyp/6-311+g(d,p)`

Charge = 0, Multiplicity = 1, Point group = C1

Electronic Energy = -1056.261364 Hartree

	Cartesian Coordinates				Cartesian Coordinates		
	X	***Y***	***Z***		***X***	***Y***	***Z***
C	-0.303196	1.194281	0.012889	C	1.085115	0.906828	0.011359
C	1.207067	-0.536057	-0.006345	C	-0.106253	-1.046929	-0.014243
N	-1.016660	-0.002164	-0.001926	C	-0.784603	2.505424	0.048899
C	0.134946	3.540360	0.072416	C	1.521380	3.279827	0.062593
C	2.004467	1.975926	0.034847	C	2.312418	-1.446071	-0.026380
C	2.021915	-2.851942	-0.058977	C	0.666511	-3.304287	-0.075559
C	-0.388725	-2.430520	-0.054993	C	3.673934	-1.049096	-0.016946
C	4.692679	-1.979785	-0.037351	C	4.404411	-3.359781	-0.068424
C	3.092283	-3.780444	-0.078944	C	-2.440179	-0.124699	-0.001286
C	-3.180794	0.302271	-1.104882	C	-4.568450	0.185330	-1.097632
C	-5.245245	-0.366828	-0.002894	C	-4.485093	-0.798577	1.090511
C	-3.097653	-0.673289	1.100623	C	-6.750832	-0.469132	0.007505
O	2.318652	4.396538	0.084980	C	3.734190	4.220938	0.081063
H	-1.847220	2.713601	0.058391	H	-0.196655	4.571431	0.099359
H	3.067008	1.791248	0.033621	H	0.481091	-4.372490	-0.107075
H	-1.411060	-2.786725	-0.070987	H	3.927710	0.001088	0.007794
H	5.724193	-1.645471	-0.029037	H	5.211841	-4.082784	-0.084259
H	2.858367	-4.839894	-0.103484	H	-2.670151	0.716329	-1.966479
H	-5.131985	0.518798	-1.962688	H	-4.983225	-1.233097	1.950770
H	-2.524424	-0.999734	1.960520	H	-7.145679	-0.623481	-0.999046

H	-7.089157	-1.292012	0.640508	H	-7.199907	0.450858	0.397121
H	4.158803	5.222912	0.100753	H	4.068336	3.667237	0.964148
H	4.067762	3.702540	-0.823391	Bq	0.174303	0.115439	-0.999578
Bq	0.172126	0.090945	1.000271	Bq	0.609886	2.210280	1.040224
Bq	0.609483	2.257268	-0.959223	Bq	0.953122	-1.910751	-1.039098
Bq	0.951189	-1.961184	0.960265	Bq	3.366931	-2.388578	-1.047581
Bq	3.365616	-2.433795	0.951907	Bq	-3.757163	-1.155273	-0.411136
Bq	-3.915308	0.663342	0.405949				

13o_NICS0

`#p nmr=giao b3lyp/6-311+g(d,p)`

Charge = 0, Multiplicity = 1, Point group = C1

Electronic Energy = -592.846677 Hartree

	Cartesian Coordinates				Cartesian Coordinates		
	X	***Y***	***Z***		***X***	***Y***	***Z***
C	-0.918435	-1.176226	0.000065	C	-0.638356	0.217868	0.000033
C	0.807985	0.356889	-0.000013	C	1.334439	-0.965149	0.000151
N	0.281999	-1.860611	0.000193	C	-2.218712	-1.685381	-0.000124
C	-3.259831	-0.763291	-0.000284	C	-3.022656	0.618395	-0.000136
C	-1.721258	1.112908	0.000113	C	1.704458	1.435677	-0.000350
C	3.074061	1.188423	-0.000287	C	3.568336	-0.126031	-0.000014
C	2.706784	-1.218387	0.000175	O	-1.566758	2.474116	0.000194
H	0.374121	-2.864383	0.000216	H	-2.406598	-2.751931	-0.000152
H	-4.284314	-1.117909	-0.000450	H	-3.847890	1.320363	-0.000112
H	1.363488	2.465481	-0.000735	H	3.767567	2.021078	-0.000518
H	4.639355	-0.293559	0.000007	H	3.087413	-2.233159	0.000310
H	-0.633956	2.711199	0.002557	Bq	0.173526	-0.685446	0.000086
Bq	-1.963208	-0.279288	-0.000056	Bq	2.199344	0.111904	-0.000056

13o_NICS1

`#p nmr=giao b3lyp/6-311+g(d,p)`

Charge = 0, Multiplicity = 1, Point group = C1

Electronic Energy = -592.846677 Hartree

	Cartesian Coordinates				Cartesian Coordinates		
	X	***Y***	***Z***		***X***	***Y***	***Z***
C	-0.918435	-1.176226	0.000065	C	-0.638356	0.217868	0.000033
C	0.807985	0.356889	-0.000013	C	1.334439	-0.965149	0.000151

N	0.281999	-1.860611	0.000193	C	-2.218712	-1.685381	-0.000124
C	-3.259831	-0.763291	-0.000284	C	-3.022656	0.618395	-0.000136
C	-1.721258	1.112908	0.000113	C	1.704458	1.435677	-0.000350
C	3.074061	1.188423	-0.000287	C	3.568336	-0.126031	-0.000014
C	2.706784	-1.218387	0.000175	O	-1.566758	2.474116	0.000194
H	0.374121	-2.864383	0.000216	H	-2.406598	-2.751931	-0.000152
H	-4.284314	-1.117909	-0.000450	H	-3.847890	1.320363	-0.000112
H	1.363488	2.465481	-0.000735	H	3.767567	2.021078	-0.000518
H	4.639355	-0.293559	0.000007	H	3.087413	-2.233159	0.000310
H	-0.633956	2.711199	0.002557	Bq	0.173552	-0.685525	-0.999914
Bq	0.173501	-0.685367	1.000086	Bq	-1.963091	-0.279239	-1.000055
Bq	-1.963325	-0.279337	0.999944	Bq	2.199369	0.112100	0.999943
Bq	2.199319	0.111707	-1.000055				

13p_NICS0

```
#p nmr=giao b3lyp/6-311+g(d,p)
```

Charge = 0, Multiplicity = 1, Point group = C1

Electronic Energy = -746.519496 Hartree

	**Cartesian Coordinates**				**Cartesian Coordinates**		
	X	***Y***	***Z***		***X***	***Y***	***Z***
C	2.182608	0.987618	-0.099756	C	1.326882	-0.150405	-0.004063
C	-0.041122	0.359122	0.003269	C	0.070938	1.763763	0.002776
N	1.397463	2.119316	-0.083266	C	3.574257	0.904740	-0.183591
C	4.138365	-0.362163	-0.130246	C	3.342519	-1.502198	0.051091
C	1.958670	-1.404334	0.148356	C	-1.355447	-0.218703	-0.044923
C	-2.480400	0.665857	0.098208	C	-2.284366	2.078021	0.182737
C	-1.035936	2.633805	0.101197	O	1.281765	-2.570881	0.412279
C	-1.631466	-1.589515	-0.291239	C	-2.921994	-2.078101	-0.299636
C	-4.016628	-1.219109	-0.076480	C	-3.791971	0.128012	0.101509
H	1.744662	3.065484	-0.111402	H	4.183418	1.795589	-0.274743
H	5.214501	-0.474622	-0.193990	H	3.797074	-2.479531	0.160472
H	-3.156454	2.714702	0.282247	H	-0.891888	3.707661	0.123673
H	0.435345	-2.376926	0.830352	H	-0.825078	-2.264636	-0.542927
H	-3.093381	-3.130003	-0.498819	H	-5.026439	-1.612605	-0.075905
H	-4.627587	0.808254	0.230419	Bq	0.987354	1.015883	-0.036208
Bq	2.753884	-0.254457	-0.036368	Bq	-1.187722	1.213644	0.057211
Bq	-2.699651	-0.718593	-0.085427				

13p_NICS1

`#p nmr=giao b3lyp/6-311+g(d,p)`

Charge = 0, Multiplicity = 1, Point group = C1

Electronic Energy = -746.519496 Hartree

	Cartesian Coordinates				Cartesian Coordinates		
	X	*Y*	*Z*		*X*	*Y*	*Z*
C	2.182608	0.987618	-0.099756	C	1.326882	-0.150405	-0.004063
C	-0.041122	0.359122	0.003269	C	0.070938	1.763763	0.002776
N	1.397463	2.119316	-0.083266	C	3.574257	0.904740	-0.183591
C	4.138365	-0.362163	-0.130246	C	3.342519	-1.502198	0.051091
C	1.958670	-1.404334	0.148356	C	-1.355447	-0.218703	-0.044923
C	-2.480400	0.665857	0.098208	C	-2.284366	2.078021	0.182737
C	-1.035936	2.633805	0.101197	O	1.281765	-2.570881	0.412279
C	-1.631466	-1.589515	-0.291239	C	-2.921994	-2.078101	-0.299636
C	-4.016628	-1.219109	-0.076480	C	-3.791971	0.128012	0.101509
H	1.744662	3.065484	-0.111402	H	4.183418	1.795589	-0.274743
H	5.214501	-0.474622	-0.193990	H	3.797074	-2.479531	0.160472
H	-3.156454	2.714702	0.282247	H	-0.891888	3.707661	0.123673
H	0.435345	-2.376926	0.830352	H	-0.825078	-2.264636	-0.542927
H	-3.093381	-3.130003	-0.498819	H	-5.026439	-1.612605	-0.075905
H	-4.627587	0.808254	0.230419	Bq	1.031724	1.039337	0.962532
Bq	0.942983	0.992428	-1.034948	Bq	2.811572	-0.159970	0.957485
Bq	2.696195	-0.348944	-1.030221	Bq	-1.137427	1.163782	1.054700
Bq	-1.238017	1.263507	-0.940278	Bq	-2.649047	-0.873772	0.901163
Bq	-2.750255	-0.563415	-1.072016				

13q_NICS0

`#p nmr=giao b3lyp/6-311+g(d,p)`

Charge = 0, Multiplicity = 1, Point group = C1

Electronic Energy = -592.846828 Hartree

	Cartesian Coordinates				Cartesian Coordinates		
	X	*Y*	*Z*		*X*	*Y*	*Z*
C	0.760704	-0.772003	0.000004	C	0.297938	0.569715	0.000004
C	-1.150468	0.510682	0.000002	C	-1.499878	-0.866271	0.000002
N	-0.333194	-1.616763	0.000005	C	2.120946	-1.090953	-0.000015
C	3.024623	-0.031586	-0.000002	C	2.592043	1.308067	0.000008
C	1.236702	1.607820	0.000004	C	-2.165970	1.473747	0.000000

C	-3.494572	1.057291	-0.000003	C	-3.820155	-0.309957	-0.000003
C	-2.829551	-1.289333	0.000000	O	4.381841	-0.234112	-0.000043
H	-0.290148	-2.623909	0.000000	H	2.462578	-2.120430	-0.000038
H	3.337038	2.094445	0.000027	H	0.915293	2.643442	0.000010
H	-1.923222	2.530810	0.000001	H	-4.289172	1.794681	-0.000005
H	-4.862032	-0.610056	-0.000006	H	-3.083667	-2.343266	0.000000
H	4.576778	-1.178790	0.000307	Bq	-0.384980	-0.434928	0.000003
Bq	1.672159	0.265177	0.000000	Bq	-2.493432	0.096026	0.000000

13q_NICS1

```
#p nmr=giao b3lyp/6-311+g(d,p)
```

Charge = 0, Multiplicity = 1, Point group = C1

Electronic Energy = -592.846828 Hartree

	Cartesian Coordinates				Cartesian Coordinates		
	X	***Y***	***Z***		***X***	***Y***	***Z***
C	0.760704	-0.772003	0.000004	C	0.297938	0.569715	0.000004
C	-1.150468	0.510682	0.000002	C	-1.499878	-0.866271	0.000002
N	-0.333194	-1.616763	0.000005	C	2.120946	-1.090953	-0.000015
C	3.024623	-0.031586	-0.000002	C	2.592043	1.308067	0.000008
C	1.236702	1.607820	0.000004	C	-2.165970	1.473747	0.000000
C	-3.494572	1.057291	-0.000003	C	-3.820155	-0.309957	-0.000003
C	-2.829551	-1.289333	0.000000	O	4.381841	-0.234112	-0.000043
H	-0.290148	-2.623909	0.000000	H	2.462578	-2.120430	-0.000038
H	3.337038	2.094445	0.000027	H	0.915293	2.643442	0.000010
H	-1.923222	2.530810	0.000001	H	-4.289172	1.794681	-0.000005
H	-4.862032	-0.610056	-0.000006	H	-3.083667	-2.343266	0.000000
H	4.576778	-1.178790	0.000307	Bq	-0.384979	-0.434929	-1.000008
Bq	-0.384981	-0.434927	1.000015	Bq	1.672161	0.265171	0.999991
Bq	1.672157	0.265182	-0.999990	Bq	-2.493430	0.096026	-1.000049
Bq	-2.493434	0.096027	1.000048				

13r_NICS0

```
#p nmr=giao b3lyp/6-311+g(d,p)
```

Charge = 0, Multiplicity = 1, Point group = C1

Electronic Energy = -746.524662 Hartree

	Cartesian Coordinates				Cartesian Coordinates		
	X	***Y***	***Z***		***X***	***Y***	***Z***

C	1.883103	0.892480	-0.000002	C	1.028093	-0.247656	0.000003
C	-0.333384	0.256624	0.000005	C	-0.235390	1.660183	0.000003
N	1.097638	2.024908	-0.000001	C	3.274790	0.798340	0.000007
C	3.828881	-0.476061	-0.000001	C	3.016133	-1.625205	-0.000006
C	1.631434	-1.514862	0.000007	C	-1.627950	-0.350630	0.000002
C	-2.774642	0.515586	0.000001	C	-2.604442	1.933822	0.000000
C	-1.360182	2.511264	0.000001	O	5.198587	-0.564694	-0.000039
C	-1.849725	-1.751836	-0.000001	C	-3.125971	-2.274807	-0.000003
C	-4.250885	-1.422971	-0.000003	C	-4.071810	-0.057226	-0.000002
H	1.440783	2.972932	-0.000005	H	3.911124	1.674894	0.000023
H	3.480731	-2.605623	-0.000021	H	1.041226	-2.421305	0.000013
H	-3.490379	2.559462	-0.000001	H	-1.238754	3.588339	0.000000
H	5.467139	-1.491033	0.000248	H	-1.005981	-2.428060	-0.000002
H	-3.265600	-3.350194	-0.000005	H	-5.249682	-1.844283	-0.000005
H	-4.931088	0.605800	-0.000002	Bq	0.688012	0.917308	0.000002
Bq	2.443739	-0.362161	0.000001	Bq	-1.489332	1.087808	0.000002
Bq	-2.950164	-0.890314	-0.000001				

13r_NICS1

```
#p nmr=giao b3lyp/6-311+g(d,p)
```

Charge = 0, Multiplicity = 1, Point group = C1

Electronic Energy = -746.524662 Hartree

	Cartesian Coordinates				Cartesian Coordinates		
	X	***Y***	***Z***		***X***	***Y***	***Z***
C	1.883103	0.892480	-0.000002	C	1.028093	-0.247656	0.000003
C	-0.333384	0.256624	0.000005	C	-0.235390	1.660183	0.000003
N	1.097638	2.024908	-0.000001	C	3.274790	0.798340	0.000007
C	3.828881	-0.476061	-0.000001	C	3.016133	-1.625205	-0.000006
C	1.631434	-1.514862	0.000007	C	-1.627950	-0.350630	0.000002
C	-2.774642	0.515586	0.000001	C	-2.604442	1.933822	0.000000
C	-1.360182	2.511264	0.000001	O	5.198587	-0.564694	-0.000039
C	-1.849725	-1.751836	-0.000001	C	-3.125971	-2.274807	-0.000003
C	-4.250885	-1.422971	-0.000003	C	-4.071810	-0.057226	-0.000002
H	1.440783	2.972932	-0.000005	H	3.911124	1.674894	0.000023
H	3.480731	-2.605623	-0.000021	H	1.041226	-2.421305	0.000013
H	-3.490379	2.559462	-0.000001	H	-1.238754	3.588339	0.000000
H	5.467139	-1.491033	0.000248	H	-1.005981	-2.428060	-0.000002
H	-3.265600	-3.350194	-0.000005	H	-5.249682	-1.844283	-0.000005

H	-4.931088	0.605800	-0.000002	Bq	0.688015	0.917309	1.000017
Bq	0.688009	0.917306	-1.000013	Bq	2.443740	-0.362162	0.999798
Bq	2.443738	-0.362160	-0.999795	Bq	-1.489330	1.087807	-1.000071
Bq	-1.489333	1.087809	1.000075	Bq	-2.950162	-0.890313	-1.000090
Bq	-2.950165	-0.890315	1.000088				

13s_NICS0

```
#p nmr=giao b3lyp/6-311+g(d,p)
```

Charge = 0, Multiplicity = 1, Point group = C1

Electronic Energy = -592.847425 Hartree

	Cartesian Coordinates				Cartesian Coordinates		
	X	*Y*	*Z*		*X*	*Y*	*Z*
C	-0.968587	-0.489459	-0.000008	C	-0.371712	0.793222	-0.000010
C	1.061244	0.568089	-0.000005	C	1.256770	-0.839778	-0.000001
N	0.019087	-1.457933	-0.000005	C	-2.357996	-0.649557	-0.000006
C	-3.147386	0.493672	-0.000005	C	-2.561200	1.774448	0.000009
C	-1.182414	1.938856	0.000002	C	2.176183	1.415522	-0.000003
C	3.449877	0.857044	0.000004	C	3.623302	-0.539328	0.000008
C	2.534489	-1.405095	0.000006	O	-2.843117	-1.932137	-0.000041
H	-0.143971	-2.452208	0.000010	H	-4.228094	0.393647	-0.000010
H	-3.209125	2.643272	0.000019	H	-0.744757	2.930230	0.000009
H	2.049372	2.492588	-0.000006	H	4.321004	1.502013	0.000006
H	4.626504	-0.950668	0.000013	H	2.672892	-2.480070	0.000008
H	-3.807916	-1.921985	0.000359	Bq	0.199360	-0.285172	-0.000006
Bq	-1.764882	0.643530	-0.000003	Bq	2.350311	0.009409	0.000001

13s_NICS1

```
#p nmr=giao b3lyp/6-311+g(d,p)
```

Charge = 0, Multiplicity = 1, Point group = C1

Electronic Energy = -592.847425 Hartree

	Cartesian Coordinates				Cartesian Coordinates		
	X	*Y*	*Z*		*X*	*Y*	*Z*
C	-0.968587	-0.489459	-0.000008	C	-0.371712	0.793222	-0.000010
C	1.061244	0.568089	-0.000005	C	1.256770	-0.839778	-0.000001
N	0.019087	-1.457933	-0.000005	C	-2.357996	-0.649557	-0.000006
C	-3.147386	0.493672	-0.000005	C	-2.561200	1.774448	0.000009
C	-1.182414	1.938856	0.000002	C	2.176183	1.415522	-0.000003

C	3.449877	0.857044	0.000004	C	3.623302	-0.539328	0.000008
C	2.534489	-1.405095	0.000006	O	-2.843117	-1.932137	-0.000041
H	-0.143971	-2.452208	0.000010	H	-4.228094	0.393647	-0.000010
H	-3.209125	2.643272	0.000019	H	-0.744757	2.930230	0.000009
H	2.049372	2.492588	-0.000006	H	4.321004	1.502013	0.000006
H	4.626504	-0.950668	0.000013	H	2.672892	-2.480070	0.000008
H	-3.807916	-1.921985	0.000359	Bq	0.199363	-0.285174	-1.000005
Bq	0.199357	-0.285170	0.999993	Bq	-1.764880	0.643525	0.999946
Bq	-1.764885	0.643535	-0.999952	Bq	2.350315	0.009406	-1.000013
Bq	2.350307	0.009412	1.000016				

13t_NICS0

`#p nmr=giao b3lyp/6-311+g(d,p)`

Charge = 0, Multiplicity = 1, Point group = C1

Electronic Energy = -900.194495 Hartree

	Cartesian Coordinates				Cartesian Coordinates		
	X	*Y*	*Z*		*X*	*Y*	*Z*
C	1.200278	-1.603041	0.041962	C	0.632132	-0.315414	-0.004861
C	-0.810338	-0.527543	-0.055831	C	-0.999512	-1.925390	-0.151841
N	0.218832	-2.553236	-0.066238	C	2.583499	-1.847043	0.202653
C	3.427680	-0.774629	0.280253	C	2.934040	0.556844	0.110750
C	1.532275	0.810616	-0.085053	C	-1.989043	0.294282	0.064357
C	-3.267090	-0.328985	-0.150209	C	-3.363132	-1.738652	-0.367248
C	-2.256993	-2.541769	-0.326839	O	2.955821	-3.160713	0.278181
C	-1.991335	1.651444	0.474780	C	-3.159839	2.379925	0.570339
C	-4.401183	1.788647	0.264095	C	-4.446112	0.454205	-0.074204
C	3.845128	1.642117	0.073062	C	3.426002	2.923579	-0.213146
C	2.068763	3.159645	-0.502426	C	1.152588	2.127902	-0.441011
H	0.376352	-3.549040	-0.083643	H	4.492447	-0.927572	0.422484
H	-4.345288	-2.171489	-0.521491	H	-2.332098	-3.617546	-0.433756
H	3.913787	-3.231108	0.372429	H	-1.063738	2.120627	0.764676
H	-3.119870	3.413088	0.897165	H	-5.313283	2.370998	0.325803
H	-5.398711	-0.029355	-0.264852	H	4.896452	1.439771	0.250112
H	4.140810	3.737997	-0.245713	H	1.739629	4.152684	-0.787188
H	0.128267	2.328869	-0.715297	Bq	0.048278	-1.384925	-0.047362
Bq	2.051651	-0.528778	0.090951	Bq	2.493133	1.870117	-0.176304
Bq	-2.114351	-1.128009	-0.164602	Bq	-3.209100	1.039920	0.191526

13t_NICS1

```
#p nmr=giao b3lyp/6-311+g(d,p)
```

Charge = 0, Multiplicity = 1, Point group = C1

Electronic Energy = -900.194495 Hartree

	Cartesian Coordinates				Cartesian Coordinates		
	X	***Y***	***Z***		***X***	***Y***	***Z***
C	1.200278	-1.603041	0.041962	C	0.632132	-0.315414	-0.004861
C	-0.810338	-0.527543	-0.055831	C	-0.999512	-1.925390	-0.151841
N	0.218832	-2.553236	-0.066238	C	2.583499	-1.847043	0.202653
C	3.427680	-0.774629	0.280253	C	2.934040	0.556844	0.110750
C	1.532275	0.810616	-0.085053	C	-1.989043	0.294282	0.064357
C	-3.267090	-0.328985	-0.150209	C	-3.363132	-1.738652	-0.367248
C	-2.256993	-2.541769	-0.326839	O	2.955821	-3.160713	0.278181
C	-1.991335	1.651444	0.474780	C	-3.159839	2.379925	0.570339
C	-4.401183	1.788647	0.264095	C	-4.446112	0.454205	-0.074204
C	3.845128	1.642117	0.073062	C	3.426002	2.923579	-0.213146
C	2.068763	3.159645	-0.502426	C	1.152588	2.127902	-0.441011
H	0.376352	-3.549040	-0.083643	H	4.492447	-0.927572	0.422484
H	-4.345288	-2.171489	-0.521491	H	-2.332098	-3.617546	-0.433756
H	3.913787	-3.231108	0.372429	H	-1.063738	2.120627	0.764676
H	-3.119870	3.413088	0.897165	H	-5.313283	2.370998	0.325803
H	-5.398711	-0.029355	-0.264852	H	4.896452	1.439771	0.250112
H	4.140810	3.737997	-0.245713	H	1.739629	4.152684	-0.787188
H	0.128267	2.328869	-0.715297	Bq	0.116362	-1.357942	-1.044676
Bq	-0.019805	-1.411908	0.949953	Bq	2.150990	-0.588554	-0.902306
Bq	1.952311	-0.469001	1.084207	Bq	2.640220	1.685286	-1.148005
Bq	2.346045	2.054949	0.795397	Bq	-2.041758	-1.007009	-1.154596
Bq	-2.186945	-1.249010	0.825393	Bq	-3.124568	1.297054	-0.771145
Bq	-3.293632	0.782785	1.154198				

11. **Supplementary Note 12:** *NMR Spectra*

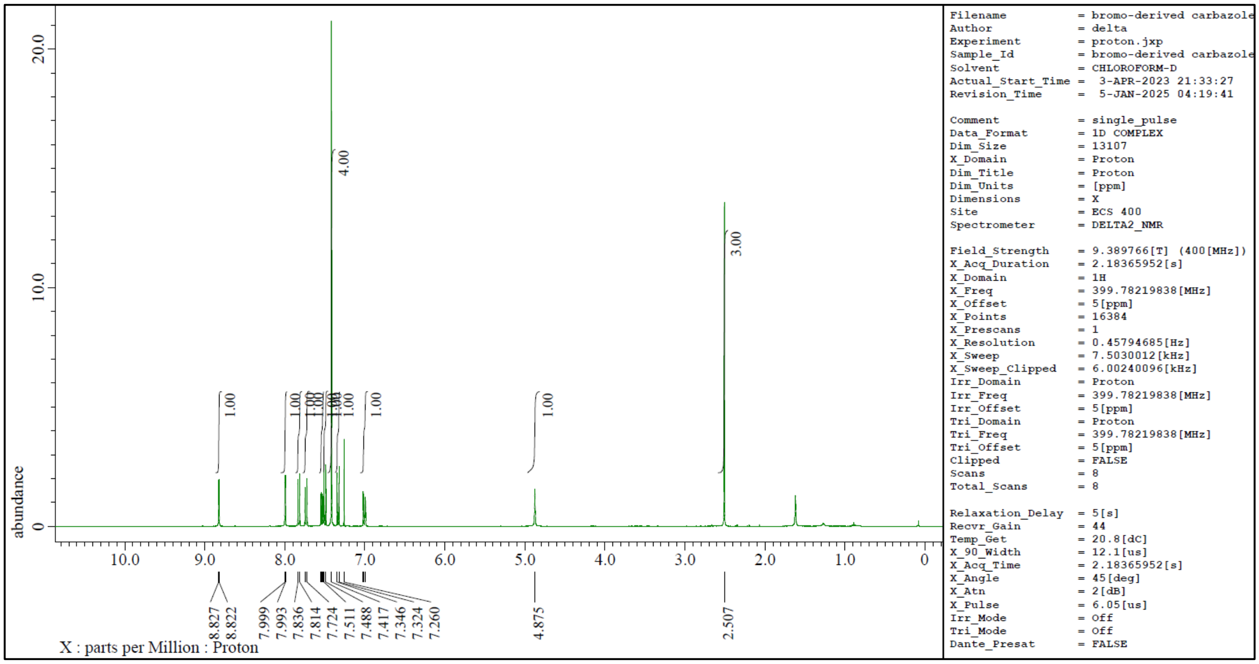

Compound **3d** (^{1}H NMR, 400 MHz, $CDCl_3$).

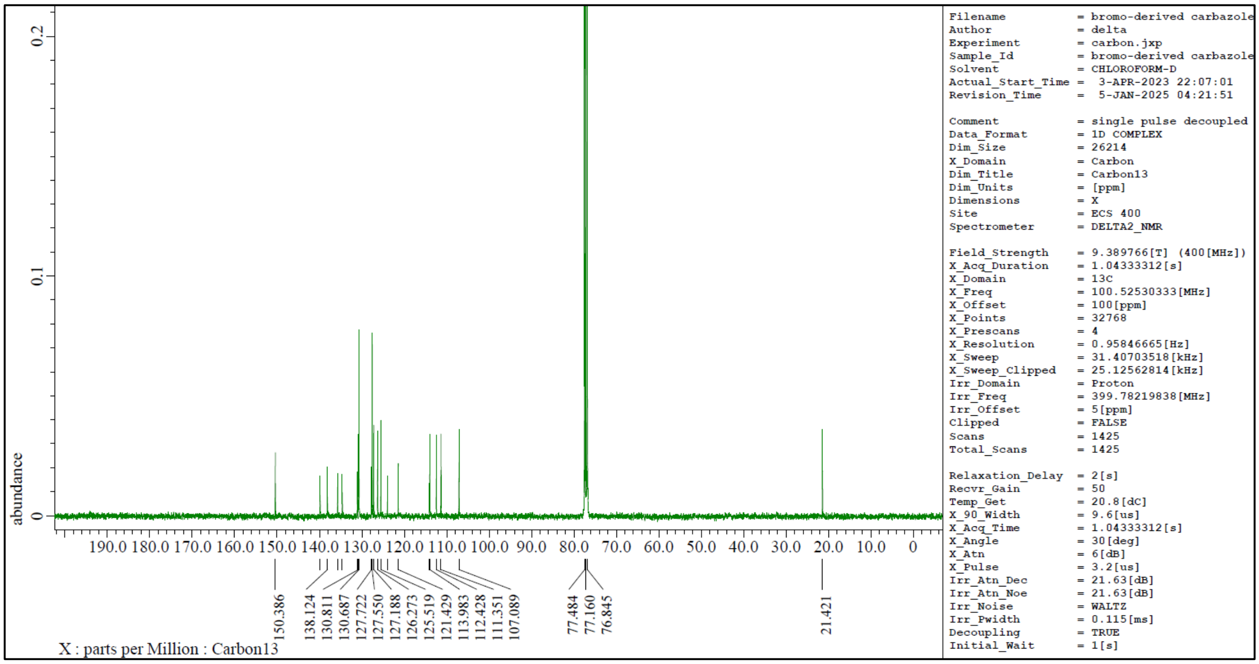

Compound **3d** (^{13}C NMR, 101 MHz, $CDCl_3$).

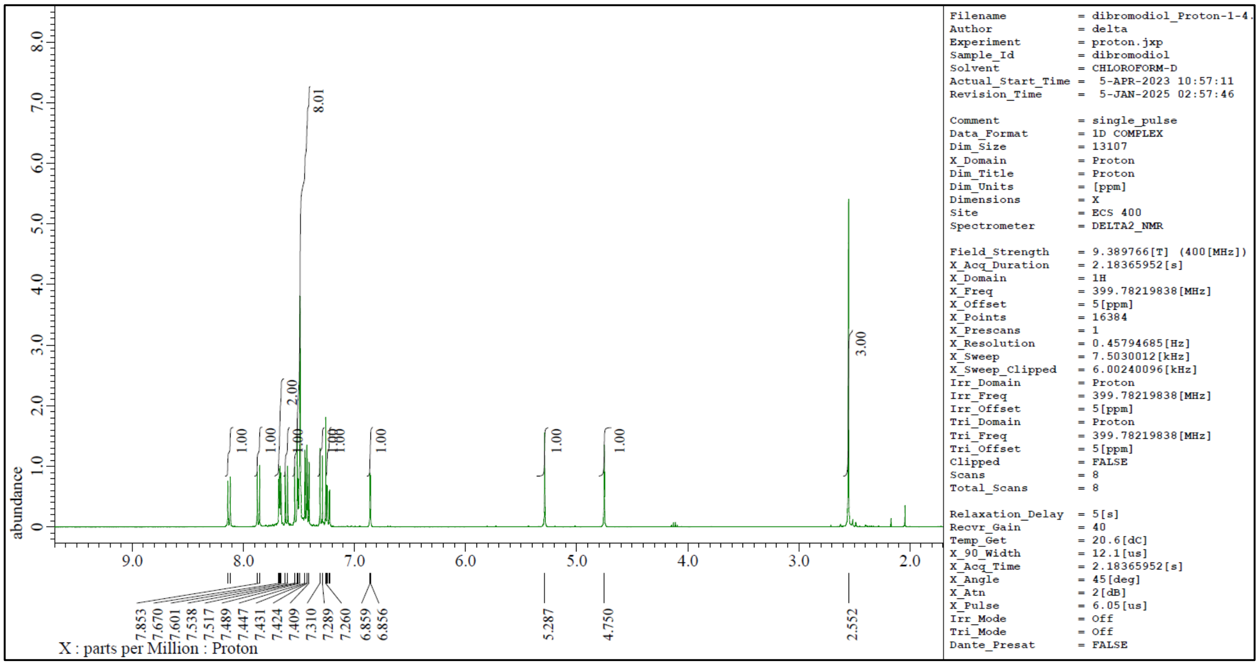

Compound **6b** (^{1}H NMR, 400 MHz, $CDCl_3$).

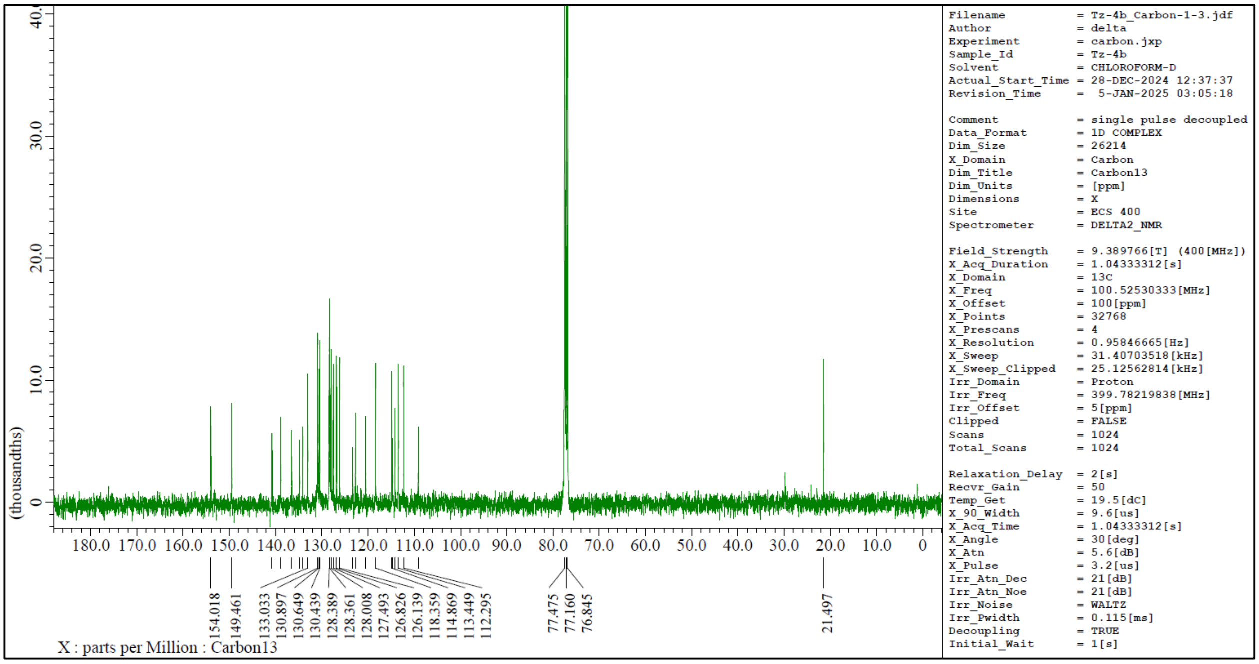

Compound **6b** (^{13}C NMR, 101 MHz, $CDCl_3$).

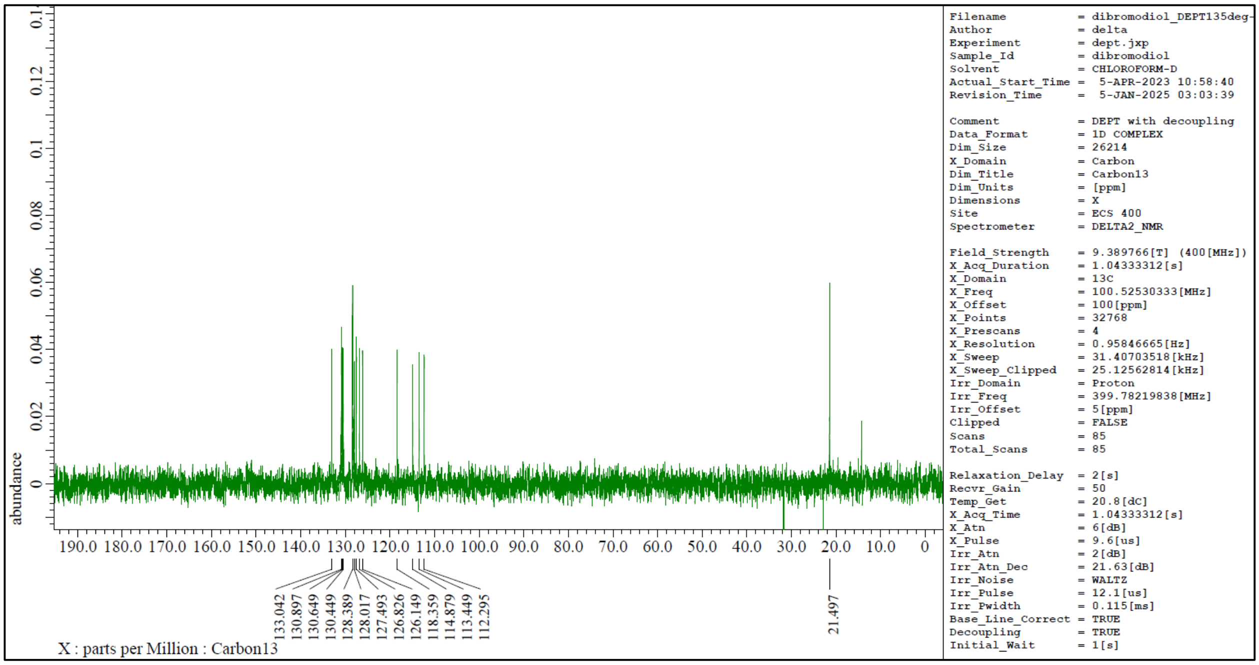

Compound **6b** (DEPT−135 NMR, 101 MHz, $CDCl_3$).

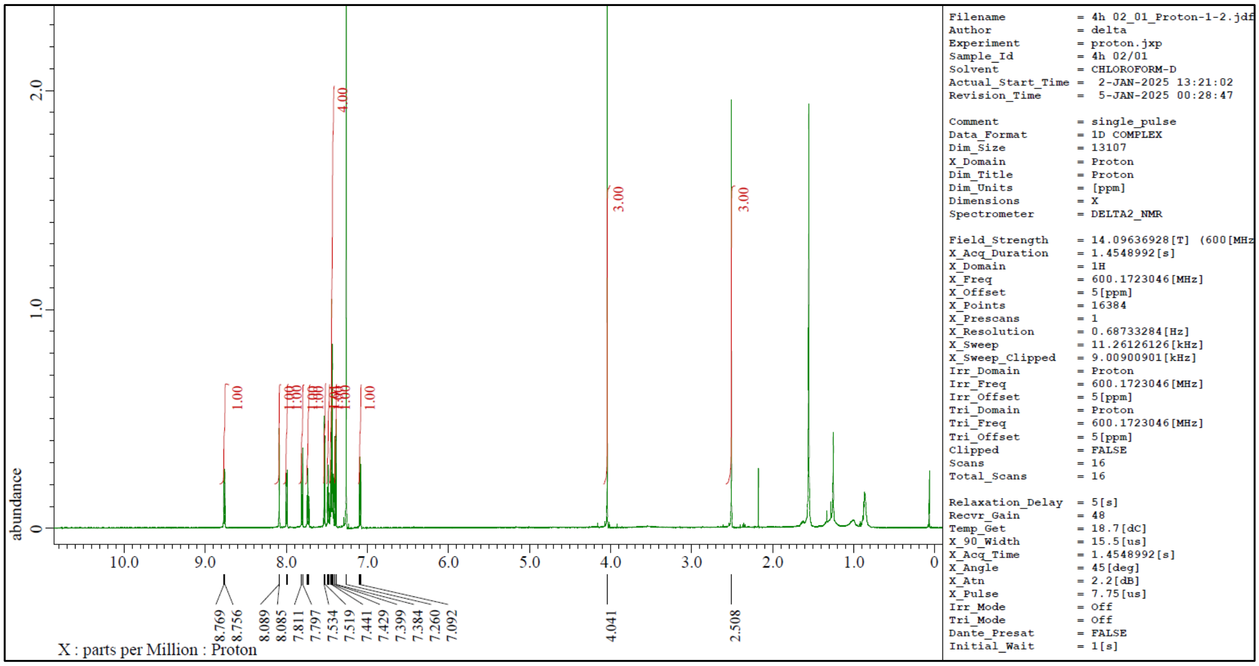

Compound **10c** (^1H NMR, 600 MHz, $CDCl_3$).

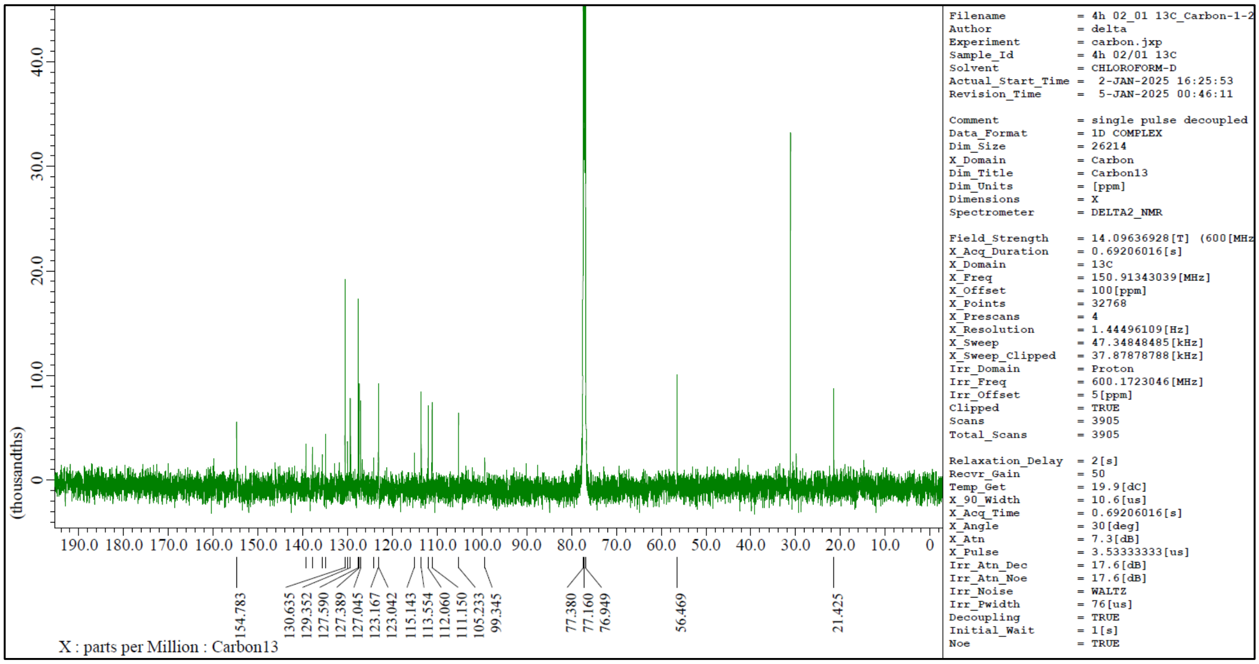

Compound **10c** (^{13}C NMR, 151 MHz, $CDCl_3$).

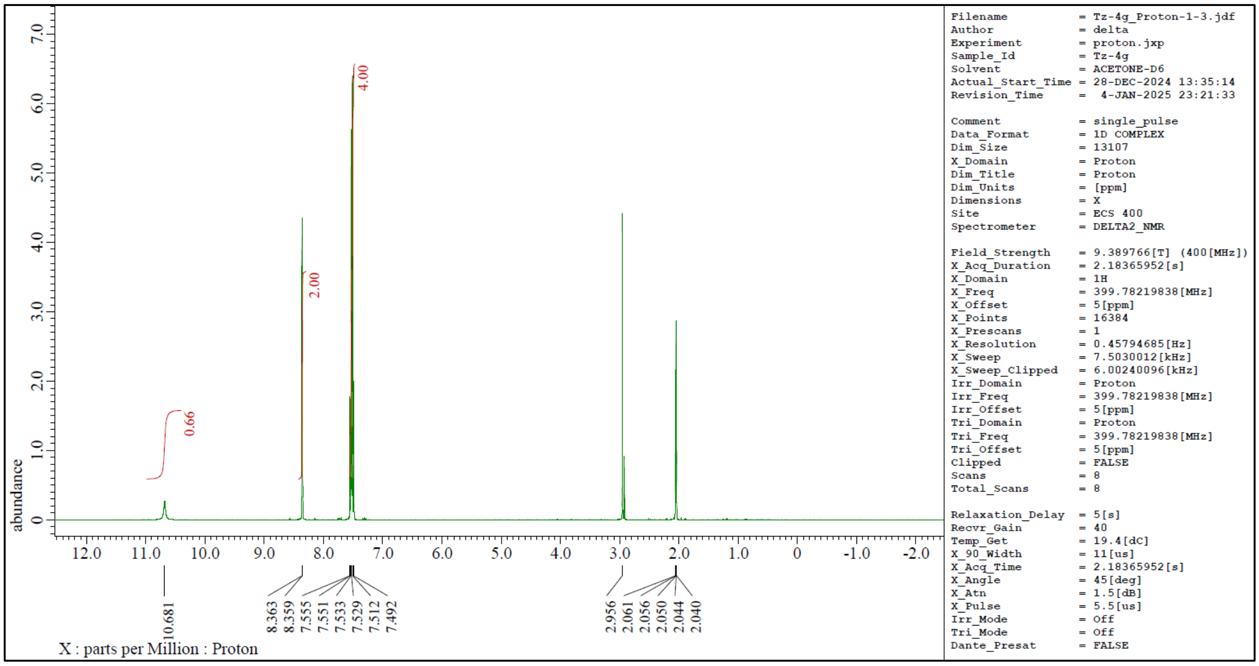

Compound **12** (^{1}H NMR, 400 MHz, $(CD_3)_2CO$).

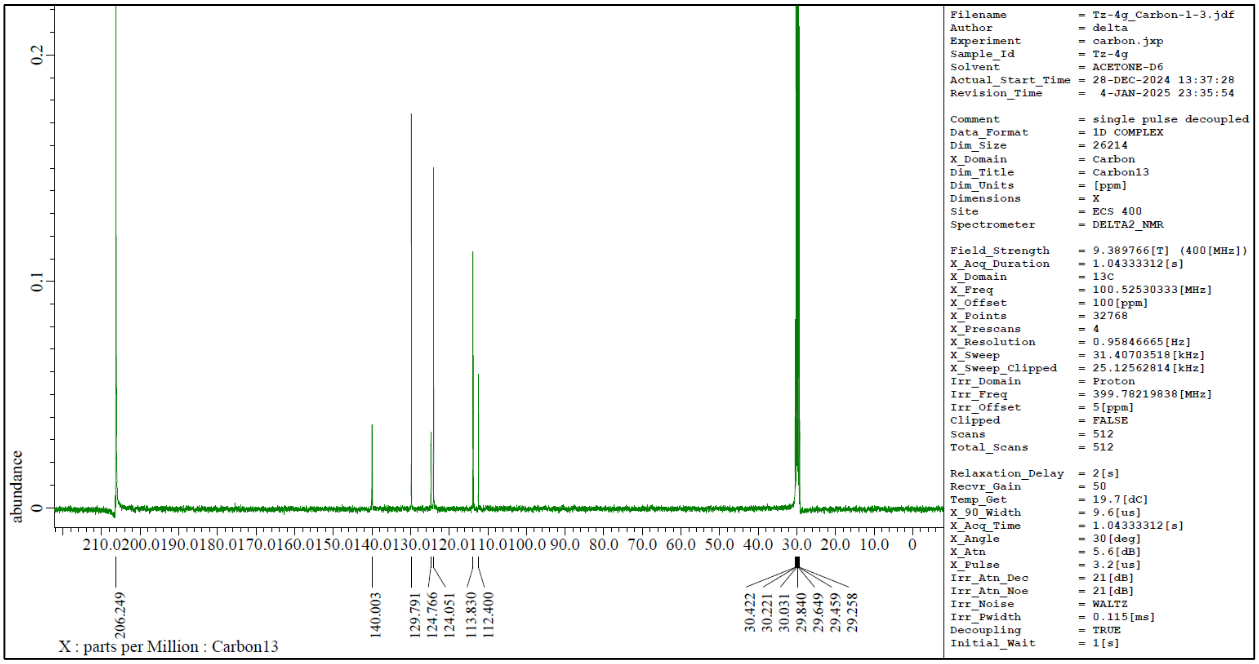

Compound **12** (^{13}C NMR, 101 MHz, $(CD_3)_2CO$).

12. Supplementary Note 13: *References*

1) Daina, A.; Michielin, O.; Zoete, V. SwissADME: a free web tool to evaluate pharmacokinetics, drug-likeness and medicinal chemistry friendliness of small molecules. *Sci. Rep.* **2017**, *7*, 42717.

2) Cheng, T.; Zhao, Y.; Li, X.; Lin, F.; Xu, Y.; Zhang, X.; Li, Y.; Wang, R.; Lai, L. Computation of octanol-water partition coefficients by guiding an additive model with knowledge. *J. Chem. Info. Model.* **2007**, *47*, 2140-2148.

3) Daina, A.; Zoete, V. A boiled-egg to predict gastrointestinal absorption and brain penetration of small molecules. *ChemMedChem* **2016**, *11*, 1117-1121.
